# Metabolomic Profiling of Citrus Grafts Challenged by *Phytophthora citrophthora*: Using the Same Samples Previously Analyzed for Epigenetic Responses

**DOI:** 10.1021/acs.jafc.5c07224

**Published:** 2025-10-28

**Authors:** Felipe Hilario, Luciano da Silva Pinto, Adielle Rodrigues da Silva, João Batista Fernandes, Abelmon da Silva Gesteira, Maria Fátima das Graças Fernandes da Silva

**Affiliations:** † Departamento de Química, 67828 Universidade Federal de São Carlos, Cp 676, São Carlos São Paulo 13565-905, Brazil; ‡ 206374 Embrapa Mandioca E Fruticultura, Cruz Das Almas, Bahia 44380-000, Brazil

**Keywords:** Phytophthora citrophthora, Citrus, metabolomics, coumarins, flavonoids, alkaloids, amino acids

## Abstract

*Phytophthora citrophthora* infection severely affects citrus crops, demanding improved diagnostic and control strategies. In a previous study, epigenetic responses, including DNA methylation patterns, were analyzed in grafted citrus plants exposed to this pathogen. Here, we report a complementary metabolomic analysis using the same biological material, stored under cryogenic conditions to preserve chemical integrity. “Pera” sweet orange and “Tahiti” acid lime scions grafted onto contrasting rootstocks were inoculated and reinoculated with *P. citrophthora*. Untargeted metabolomics via LC-MS/MS and molecular networking revealed infection-induced metabolic shifts, including increased levels of coumarins, flavonoids, and alkaloids potentially involved in plant defense. Sixty-six metabolites were annotated, and their distribution varied according to graft combinations and inoculation regimes. While the chemical profiles provided insights into differential metabolic responses, they did not directly correlate with previously observed DNA methylation patterns. Nonetheless, the results support the use of metabolomics for early detection of gummosis and highlight the value of integrating molecular approaches to guide citrus breeding for disease resistance.

## Graphical abstract

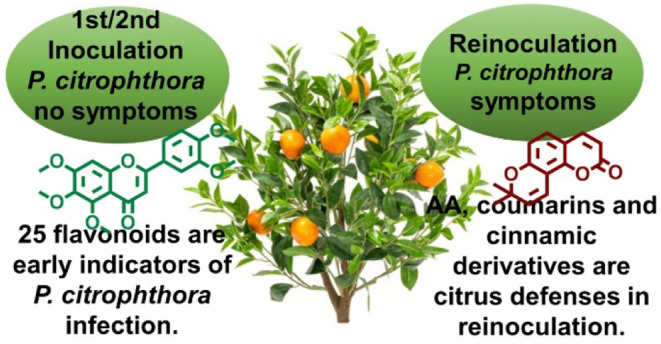

## 1 Introduction

*Citrus* production is significantly impacted by *Phytophthora* oomycete species. Among them, *P. citrophthora* (R. E. Sm. & E. H. Sm) Leonian predominantly infects the aerial parts of citrus plants, causing foot rot, commonly referred to as gummosis, root rot, and brown rot in fruits. Grafting onto rootstocks resistant to oomycete infections remains the most effective strategy for controlling *Phytophthora* gummosis, as all commercial citrus scion cultivars are susceptible to *Phytophthora* spp.^1^ Studies conducted by Gesteira and colleagues suggest that epigenetic modifications can enhance plant defense against biotic stress, offering potential strategies to improve citrus resistance to *Phytophthora* gummosis. Their findings indicate that short- and long-term biotic stress stimuli can be imprinted and transmitted through epigenetic memory, primarily via histone modifications and alterations in DNA methylation. Systemic acquired resistance (SAR) enhances the ability of infected plants to recognize biotic stress, activating defense mechanisms in distal tissues and preventing further infections. Changes in DNA (de)­methylation patterns can also modulate SAR.^2^ Gesteira and his team investigated the influence of scion-rootstock interactions on plant resistance to *P. citrophthora* infection.^1^ They assessed DNA methylation patterns in “Pera” sweet orange (*Citrus sinensis* L. Osbeck) and “Tahiti” acid lime [*C. latifolia* (Yu. Tanaka) Tanaka] grafted onto “Rangpur” lime (*C. limonia* Osbeck) and “Tropical” sunki (*C. sunki* Hayata) rootstocks, following reinoculation with *P. citrophthora*. Results revealed that reinoculated plants of “Pera” sweet orange (less susceptible)/“Rangpur” lime (highly susceptible) and “Tahiti” acid lime (highly susceptible)/“Tropical” sunki (less susceptible) exhibited smaller stem lesions and an increased frequency of full and hemimethylation patterns compared to their initially inoculated counterparts. Conversely, “Tahiti” acid lime/“Rangpur” lime (both highly susceptible) and “Pera”/“Tropical” sunki (both less susceptible) displayed a higher frequency of hemimethylation and nonmethylated sites. These findings suggest that scion-rootstock interactions and DNA methylation jointly influence citrus responses to *P. citrophthora* infection. The initial inoculation triggered biotic stress memory in reinoculated plants, reinforcing their defense responses, particularly when either the scion or rootstock was highly susceptible. Reinoculated plants frequently demonstrated a more rapid and robust immune response, limiting pathogen proliferation.

In a previous study,^1^ a controlled grafting and inoculation protocol was developed to investigate the response of citrus plants to *Phytophthora citrophthora*. In this experiment, “Pera” sweet orange and “Tahiti” acid lime scions were grafted onto “Rangpur” lime and “Tropical” sunki rootstocks following a sequence of pathogen inoculations.^3^ The design included control plants, plants inoculated only before grafting, only after grafting, and plants inoculated at both stages (Figure 1). These treatments allowed the differentiation of plant responses to infection during distinct developmental phases. Notably, symptoms such as dark lesions beneath the bark were observed in plants inoculated postgrafting and reinoculated, while control and pregrafting inoculated plants showed little to no visible symptoms. This experimental setup generated four biological groups (GA–GD, Figure 2), some of which were previously analyzed for epigenetic changes.^1^ In the present study, we focused on the metabolomic profiling of the same plant material to further explore biochemical responses associated with pathogen challenge in grafted citrus.

**Figure 1:**
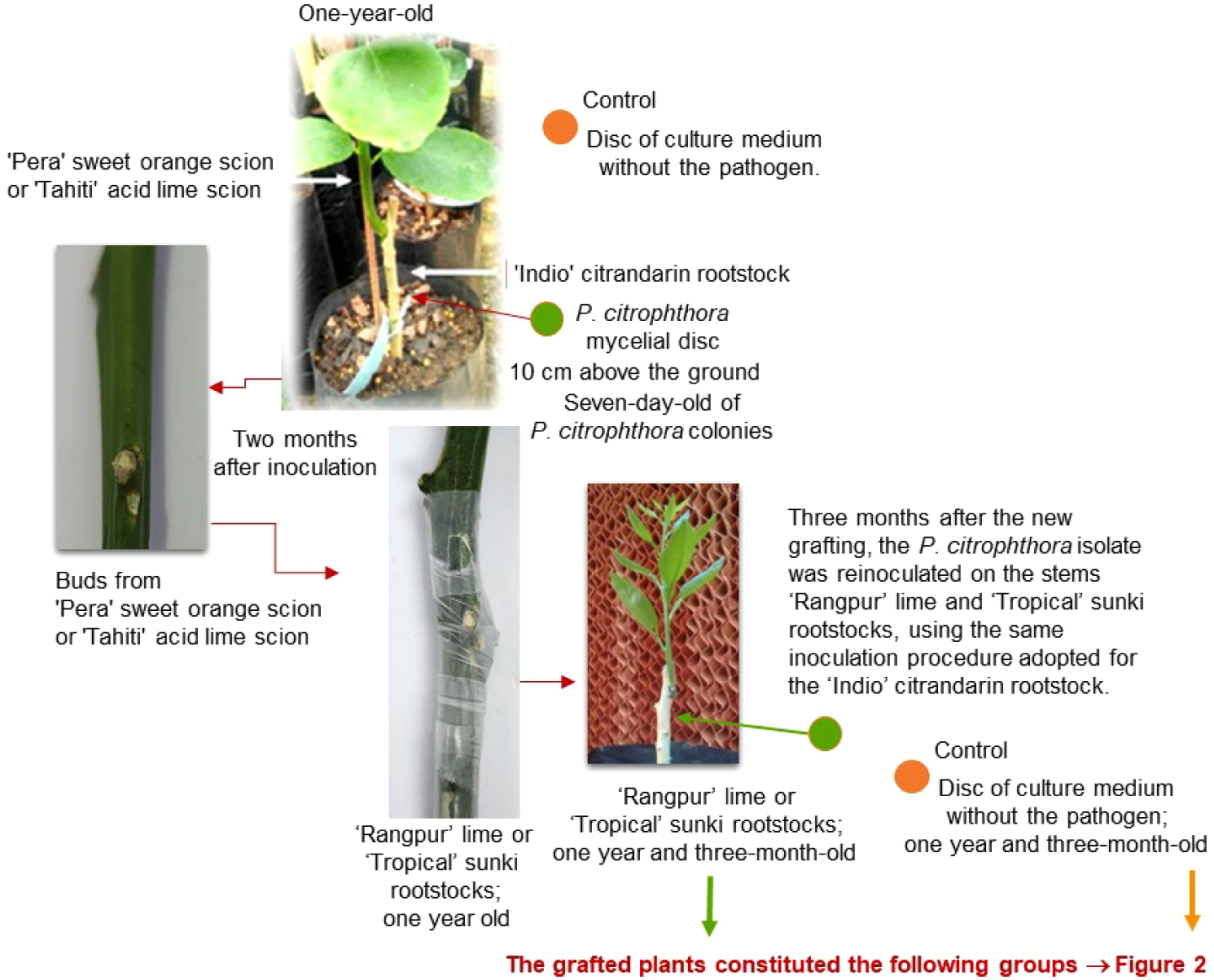
Design of the inoculation trials carried out with *Phytophthora citrophthora* mycelium discs.^1^ Green solid circle: One-year-old seedlings of “Pera” sweet orange and “Tahiti” acid lime scions grafted onto “Indio” citrandarin rootstock were inoculated with *P. citrophthora* mycelial discs on rootstock stems, following the mycelium disc under-bark inoculation method.^3^ Two months postinoculation, buds from control and infected “Pera” sweet orange and “Tahiti” acid lime scions were grafted onto one-year-old “Rangpur” lime and “Tropical” sunki rootstocks. Orange solid circle: Control plants underwent the same procedure using culture medium discs without the pathogen.

**Figure 2:**
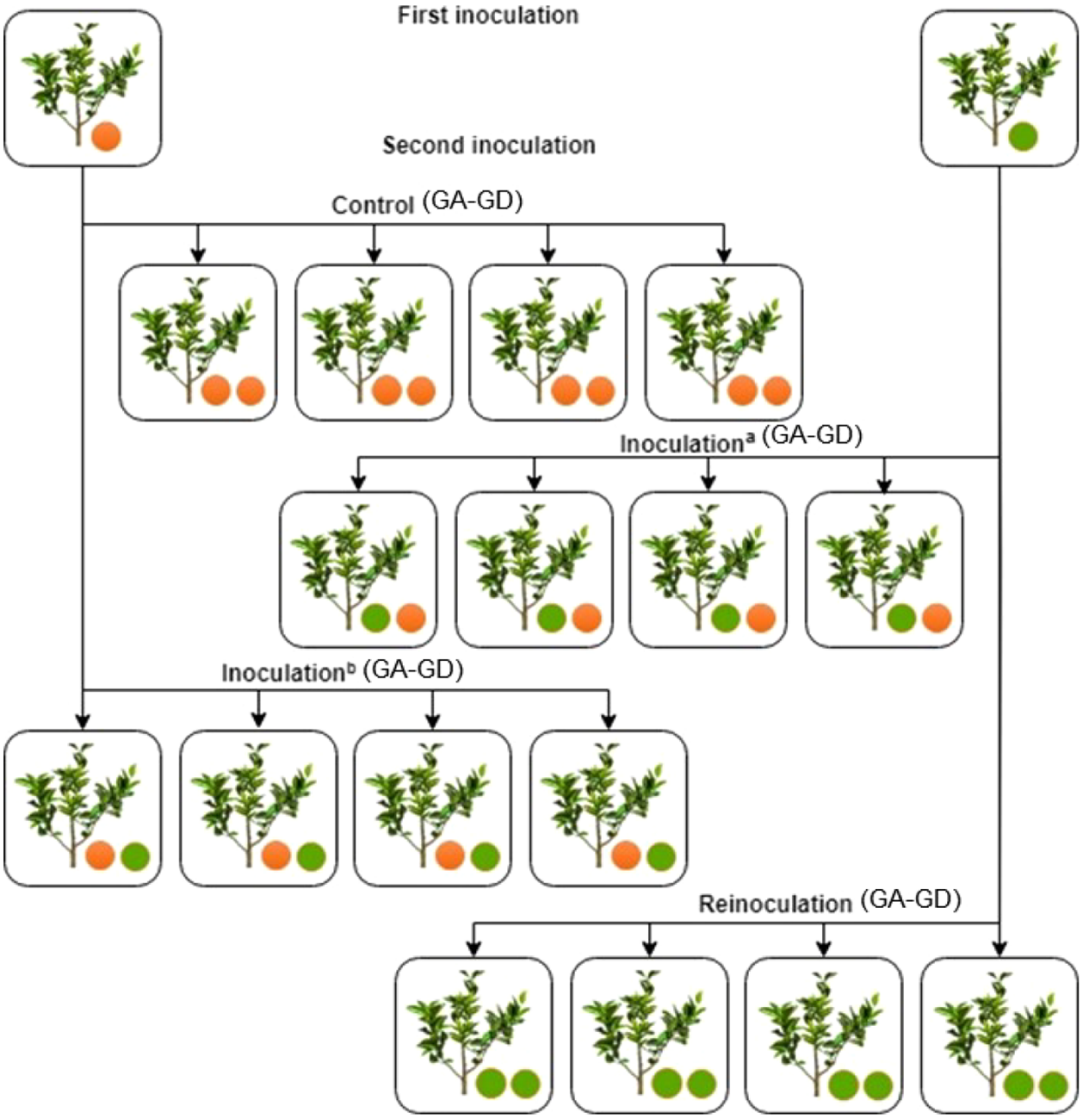
Design of the inoculation trials carried out with *Phytophthora citrophthora* mycelium discs: the grafted plants constituted the following groups I–IV; the grafted plants controls [scion “Pera” sweet orange/“Rangpur” lime rootstock (GA), scion “Pera” sweet orange/“Tropical” sunki rootstock (GB), scion “Tahiti” acid lime/“Rangpur” lime rootstock (GC), scion “Tahiti” acid lime/“Tropical” sunki rootstock (GD)], inoculated^(a)^ (GA-GD), inoculated^(b)^ (GA-GD), and reinoculated (GA-GD); and the plants were assessed 90 days after inoculation.

*Citrus* species are prolific sources of bioactive secondary metabolites with commercial significance.^4^ Their genetic and metabolic diversity reflects their evolutionary adaptation to phytopathogens. However, the complexity of plant-pathogen interactions has hindered comprehensive chemical profiling studies. This study aims to analyze the samples examined by the Gesteira group to assess the production of phytoanticipins and phytoalexins in response to *P. citrophthora* infection. Enhanced biosynthesis of endogenous compounds, such as flavonoids and coumarins, has been correlated with increased resistance to fungi and bacteria.^4^

Recent metabolomics approaches leverage advanced analytical techniques, particularly LC-MS, to analyze diverse metabolic classes.^5^ The vast MS/MS fragmentation data sets generated by automated analysis require web-based services for data simplification and pattern recognition. Among these approaches, molecular networking is particularly effective in processing tandem MS/MS fragmentation data, clustering structurally related compounds based on spectral similarity. Several online platforms facilitate efficient analysis and knowledge dissemination through molecular networking. The Global Natural Products Social Molecular Networking (GNPS) platform has been instrumental in clustering plant and microbial extracts, leading to the correlation of biological functions such as species grouping and bioactive compound identification.^6^ Given that reinoculated plants frequently exhibit a faster and stronger immune response, reducing pathogen spread, we propose integrating molecular networking with the analysis of the samples from the Gesteira group.^1^ This strategy will enable rapid and reliable clustering and characterization of bioactive compounds involved in *P. citrophthora* resistance.

## 2 Materials and Methods

### 2.1 Materials

For extraction, HPLC-grade methanol was sourced from Sigma-Aldrich (St. Louis, MO, USA). LC-MS-grade methanol, acetonitrile, and water were obtained from J.T. Baker (Phillipsburg, NJ, USA), while LC-MS-grade isopropyl alcohol was supplied by Merck (Darmstadt, Germany). Ultrapure water was generated using a Milli-Q purification system (Millipore, Merck, Germany). LC-MS-grade formic acid from Fluka (Missouri, USA) was added to the mobile phase for chromatographic analysis.

### 2.2 Plants

The plant samples analyzed in this study were obtained from greenhouse experiments conducted at Embrapa Mandioca e Fruticultura, located in Cruz das Almas, Bahia, Brazil (12°40′39“S, 39°06′23”W, at 225 m altitude). All inoculated plants were maintained at 22 ± 2 °C, relative humidity of approximately 80 ± 10%, and a 12/12 h light/dark photoperiod. These samples were partly used in the study carried out by the Gesteira group and summarized in the introduction (Figures 1 and 2), the remainder was frozen at −80 °C, preserving the biochemical characteristics of the samples exactly as analyzed in the work of this group.^1^ Cryogenic storage dewar was used to send the samples to São Carlos Federal University for chemical analyses. Four biological replicas of the roots and leaves from each group, scion “Pera” sweet orange/“Rangpur” lime rootstock (GA), scion “Pera” sweet orange/“Tropical” sunki rootstock (GB), scion “Tahiti” acid lime/“Rangpur” lime rootstock (GC), scion “Tahiti” acid lime/“Tropical” sunki rootstock (GD), based on treatments control, inoculated^a^, inoculated^b^, and reinoculated were analyzed. A total of 128 samples (roots and leaves) were collected from four graft combinations (GA–GD) subjected to four treatments (control, inoculated^a^, inoculated^b^, and reinoculated). Each treatment comprised *n* = 16 independent samples (four biological replicates per graft combination), totaling 64 root and 64 leaf samples. All samples were stored at – 80 °C until laboratory analysis.

### 2.3 Metabolite Extraction

Frozen leaf and root tissues were finely ground using an analytical mill (A11 Basic, IKA, Germany). Approximately 500 mg of each homogenized sample was transferred into a 50 mL centrifuge tube, followed by the addition of 20 mL methanol. Samples were vortexed for 20 s and subjected to ultrasonic extraction at 25 °C for 30 min using an ultrasonic bath (USC 1400 Unique, São Paulo, Brazil). These conditions were optimized to enhance metabolite solubility while minimizing thermal degradation. After extraction, samples were centrifuged at 7500 rpm for 5 min, and supernatants were collected into glass vials. The extraction was repeated twice to maximize metabolite recovery. The combined supernatants were purified using solid-phase extraction (SPE) with XTRATA C18 cartridges to remove lipophilic contaminants. Purified extracts were centrifuged at 3200 × g for 1 min at 10 °C, filtered through a 0.22 μm PTFE membrane, and concentrated using a rotary evaporator. Residues were redissolved in methanol at 1 mg/mL and stored at −20 °C for metabolomic analysis.

### 2.4 UHPLC-HRMS/MS Analysis of Metabolite Profiles

Metabolite profiling was performed using an Ultra-High-Performance Liquid Chromatography Quadrupole Time-Of-Flight Tandem Mass Spectrometry. The analysis was conducted using an Agilent 1290 Infinity II UHPLC system (Agilent Technologies Inc., Santa Clara, CA, USA) equipped with a ZORBAX Eclipse XDB-C18 analytical column (100 mm × 2.1 mm, 1.8 μm particle size; Agilent Technologies). The system was interfaced with an Agilent G6545B quadrupole time-of-flight (Q-TOF) mass spectrometer equipped with an electrospray ionization (ESI) source operating in positive ionization mode. Data acquisition and processing were performed using Agilent MassHunter software (version B.10.00).

Chromatographic separation was carried out at a constant temperature of 33 °C using a mobile phase composed of H_2_O with 0.1% formic acid (Phase A) and acetonitrile with 0.1% formic acid (Phase B). Gradient elution began at 10% Phase B, increasing linearly to 100% over 20 min, followed by a return to initial conditions and re-equilibration for 5 min. The flow rate was maintained at 0.3 mL/min, and sample injections (5 μL, 100 ppm) were performed with a needle wash step between injections to prevent carryover. Using an electrospray ionization (ESI) source operated in positive ionization mode, mass spectra were recorded over an *m*/*z* range of 100–1700, applying a capillary voltage of 2.4 kV, a skimmer voltage of 65 V, and a fragmentor voltage of 110 V. Nebulizer gas pressure was set to 28 psig, with drying and sheath gases flowing at 10 L/min and temperatures maintained at 300 and 350 °C, respectively. Data were collected at a rate of 3 spectra per second. Automatic MS/MS fragmentation was conducted using collision-induced dissociation (CID) with collision energies ranging between 25 and 60 eV to enhance structural identification. For quality control (QC), 70 μL aliquots from 16 representative extracts were pooled into a single vial to ensure analytical accuracy and reproducibility. QC samples were crucial for maintaining precision in untargeted metabolomics.^7^

### 2.5 Quality Control and Data Processing

Pooled quality control (QC) samples were injected in triplicate across the analytical run. For each LC–MS feature detected prior to metabolite annotation, the within-QC variation was determined as relative standard deviation (RSD%). Only features with RSD% ≤ 20 among all QC replicates were retained to ensure data set reliability. The raw LC–MS/MS data set comprised 6,078 features for leaves and 7,890 for roots. Following peak alignment, blank subtraction, and QC-driven filtering, 2,072 features in leaves and 3,643 in roots remained. Features containing missing or zero values were excluded from subsequent statistical evaluations. Final statistical analyses were conducted solely on the 66 metabolites confidently annotated (30 in roots and 36 in leaves, Table S1, S2 and Figure 3), as described in the results section.

**Figure 3:**
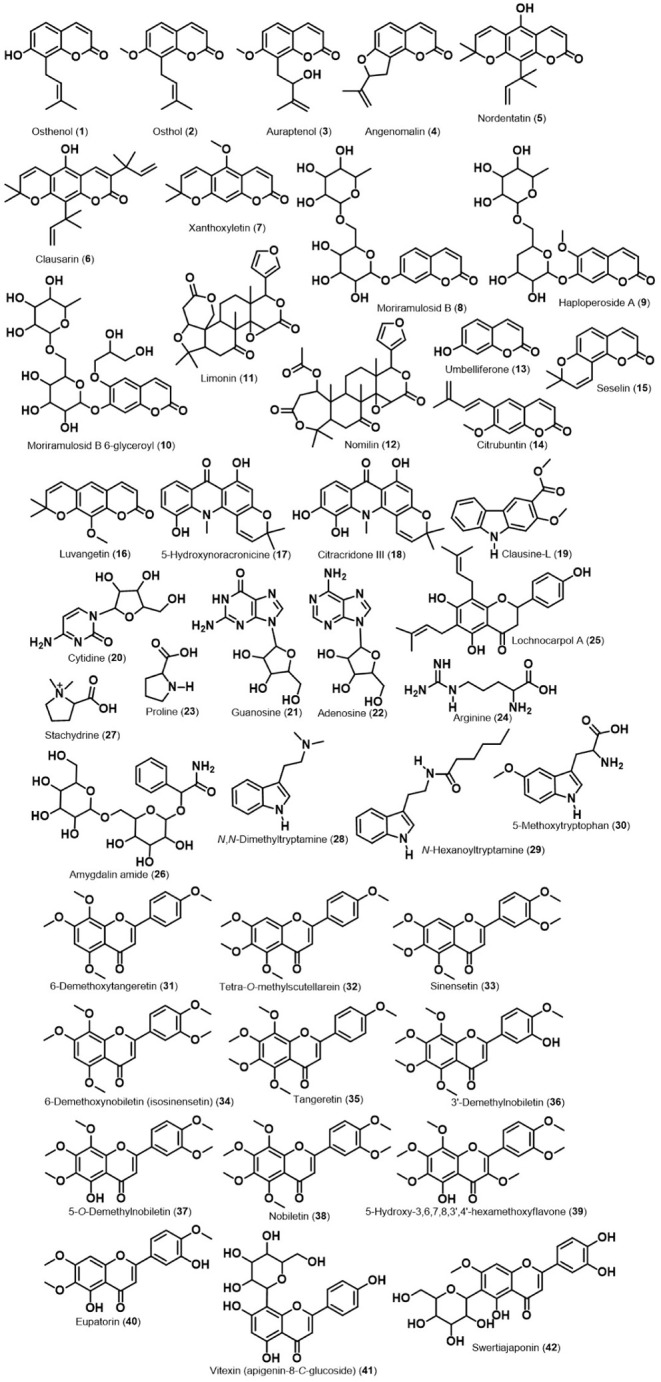

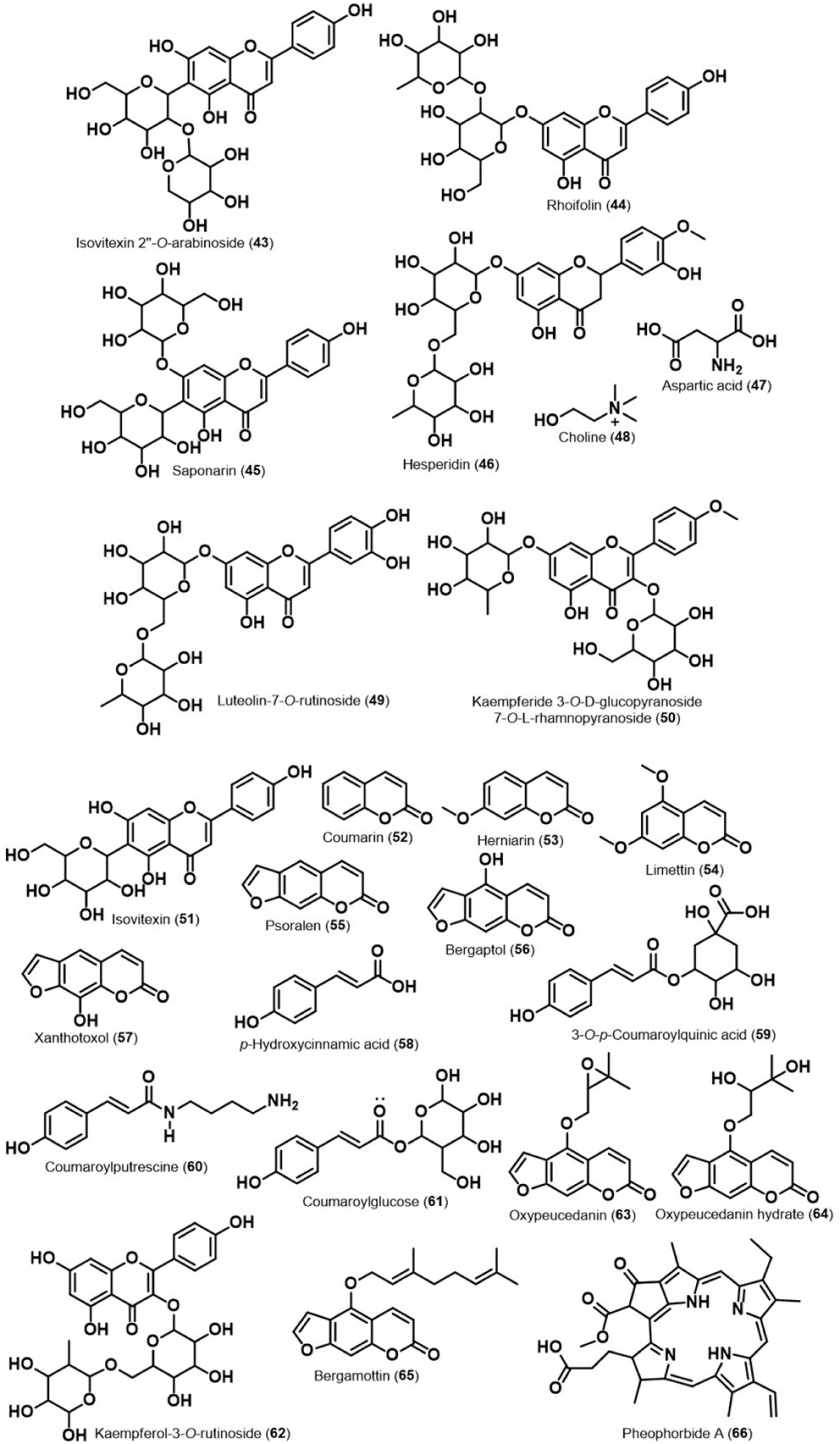
Structures of compounds detected in roots and leaves from scion “Pera” sweet orange/“Rangpur” lime rootstock (GA), scion “Pera” sweet orange/“Tropical” sunki rootstock (GB), scion “Tahiti” acid lime/“Rangpur” lime rootstock (GC), scion “Tahiti” acid lime/“Tropical” sunki rootstock (GD).

Raw mass spectrometry data were converted to mzML format using MSConvert (ProteoWizard, version 3.0). Peak detection, spectral deconvolution, and retention time alignment were performed using MS-DIAL (version 4.70) with the following processing parameters: precursor ion tolerance set at 0.02 Da, fragment ion tolerance at 0.06 Da, a minimum peak height threshold of 10.000 counts, retention time alignment tolerance values set at 0.05 min (initial) and 0.02 min (final), mass range from 50 to 1500 Da, smoothing level of 3 scans, minimum peak width of 5 scans, and maximum charge state set to 2. Mass spectral deconvolution parameters included a sigma window value of 0.5 and a mass slice width of 30 Da. A comprehensive analysis of the LC–MS/MS data was performed, employing MS^2^ fragment information and a blank-subtraction approach within MS-DIAL to accurately identify and quantify the target metabolites. For both leaves and roots, raw LC-MS/MS data (positive ion mode) were processed in MS-DIAL with the “MS2 acquired” and “Blank filter” options enabled. The initial data sets comprised 6,078 detected features in leaves and 7,890 in roots. After peak alignment, blank subtraction, and filtering, 2,072 and 3,643 features, respectively, were retained for subsequent analyses. Features with zero or missing values were excluded from downstream statistical modeling. Quality control (QC) samples were used as references, and blank samples were removed prior to data analysis. Postprocessing results, including mass spectra in.mgf format and feature quantification tables in.csv format, were exported and subsequently uploaded to the Global Natural Products Social Molecular Networking (GNPS) platform (https://gnps.ucsd.edu/) to perform molecular networking analysis and metabolite annotation.

### 2.6 Molecular Networking

Feature-based molecular networking (FBMN) analysis was performed using the GNPS online platform, with precursor ion and fragment ion mass tolerances set at 0.02 and 0.06 Da, respectively. Molecular networks were constructed by linking metabolite features that shared at least four matching fragment ions. Connections within the network were established based on a cosine similarity score exceeding 0.7 and a minimum of four matched spectral peaks. Similarly, parameters for the library search used to compare experimental spectra with reference data were set with the same criteria: a cosine similarity score above 0.7 and at least four matched peaks. These parameters facilitated metabolite annotation at level three, following the guidelines of the Metabolomics Standards Initiative.^8^

For compound identification, spectral data were matched against public databases such as METLIN and MassBank, facilitated by MassHunter PCDL Manager B.08.00 software (Agilent Technologies). The molecular formula prediction,^9^ and structural annotation based on in silico fragmentation patterns was conducted using MS-FINDER software (version 3.61; https://systemsomicslab.github.io/compms/msfinder/main.html).

### 2.7 Univariate Statistical Analysis

To complement the multivariate results and to follow best practices for robust reporting in metabolomics studies, we conducted univariate statistical analyses for each of the 66 detected metabolites (30 from roots and 36 from leaves). For each tissue, data were grouped into four treatments: control, inoculated^a^, inoculated^b^ and reinoculated, with *n* = 16 independent samples per group (four graft combinations, GA–GD × four biological replicates). Normalized peak intensities were used to calculate the mean ± standard deviation (SD) for each group. Fold change (FC) and log_2_FC values were calculated for each comparison as Control versus treatment. Statistical significance was evaluated using ANOVA, with p-values, F-statistics, and false discovery rate (FDR)-adjusted p-values (q-values) reported. Biological relevance was defined as |log_2_FC| ≥ log_2_(1.5).^10–12^ Complete univariate results, including all descriptive statistics and significance indicators, are provided in Table S3.

### 2.8 Multivariate Statistical Analysis

Multivariate analyses were performed to evaluate chemical relationships among graft combinations and treatments. Hierarchical cluster analysis (HCA) was conducted in MetaboAnalyst (version 6.0)^13^ using relative metabolite abundances to group samples (GA–GD; inoculated^a^, inoculated^b^ and reinoculated). HCA revealed distinct clusters based on compound abundance. A heatmap complemented the analysis, providing an overview of metabolite distribution between roots and leaves and highlighting treatment- and graft-specific differences.

## 3 Results and Discussion

### 3.1 Metabolic Profiling of GA-GD Grafts

A comprehensive metabolic analysis was performed on the leaves and roots of grafted plants from the Gesteira group (Figures 1 and 2). Methanolic extracts from four biological replicates of each tissue type (leaves and roots from GA-GD grafts I–IV) were subjected to nontargeted liquid chromatography–tandem mass spectrometry (LC-MS/MS) analysis. Leaf and root samples were analyzed independently to assess differences in their chemical profiles. To facilitate data interpretation, molecular networking was employed using the Global Natural Products Social Molecular Networking (GNPS) platform for extracts derived from GA-GD grafts (Figure 2).^6^ Given the possibility of false positives arising from automated spectral matching, all library matches were manually validated against spectral data available in the referenced literature (see Schemes S1–S66). Key spectral features are summarized in Table S1.

The global molecular network revealed multiple shared nodes across all leaf and root fractions of the GA-GD grafts. To investigate graft-specific metabolic variation potentially linked to scion-rootstock interactions in response to *Phytophthora citrophthora* infection, additional networks were generated for each graft type individually. A total of 66 metabolites were identified (Figure 3), and their roles in the plant–oomycete interaction are discussed in subsequent sections (Figures 4–12 and S1–S6).

**Figure 4:**
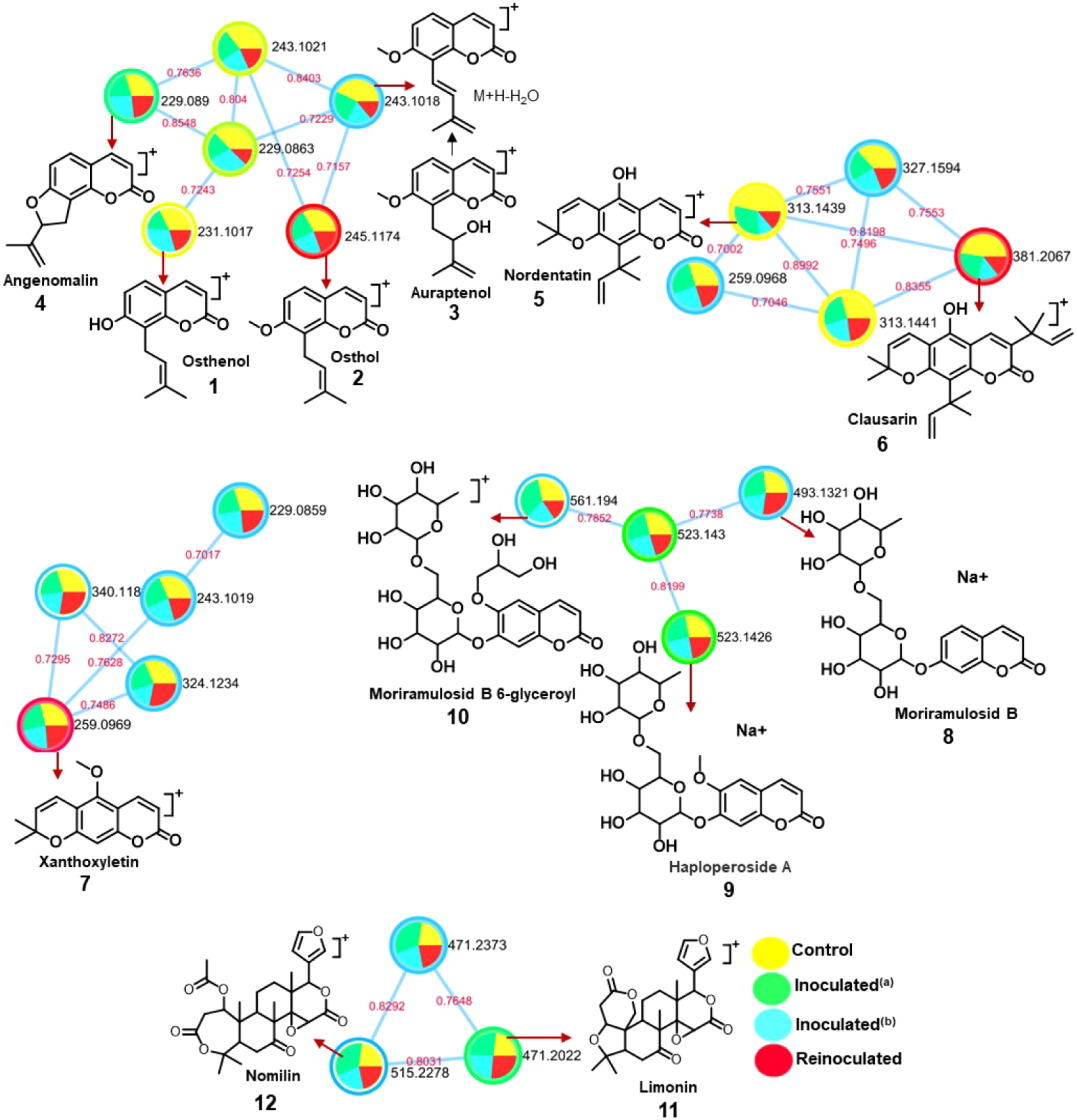
Molecular network of the analysis carried out in positive ion monitoring for roots from scion “Pera” sweet orange/“Rangpur” lime rootstock (GA), control (yellow), inoculated^(a)^ (green), inoculated^(b)^ (blue), and reinoculated (red), and selected clusters with nodes showing the compounds annotated by GNPS library search.

**Figure 5:**
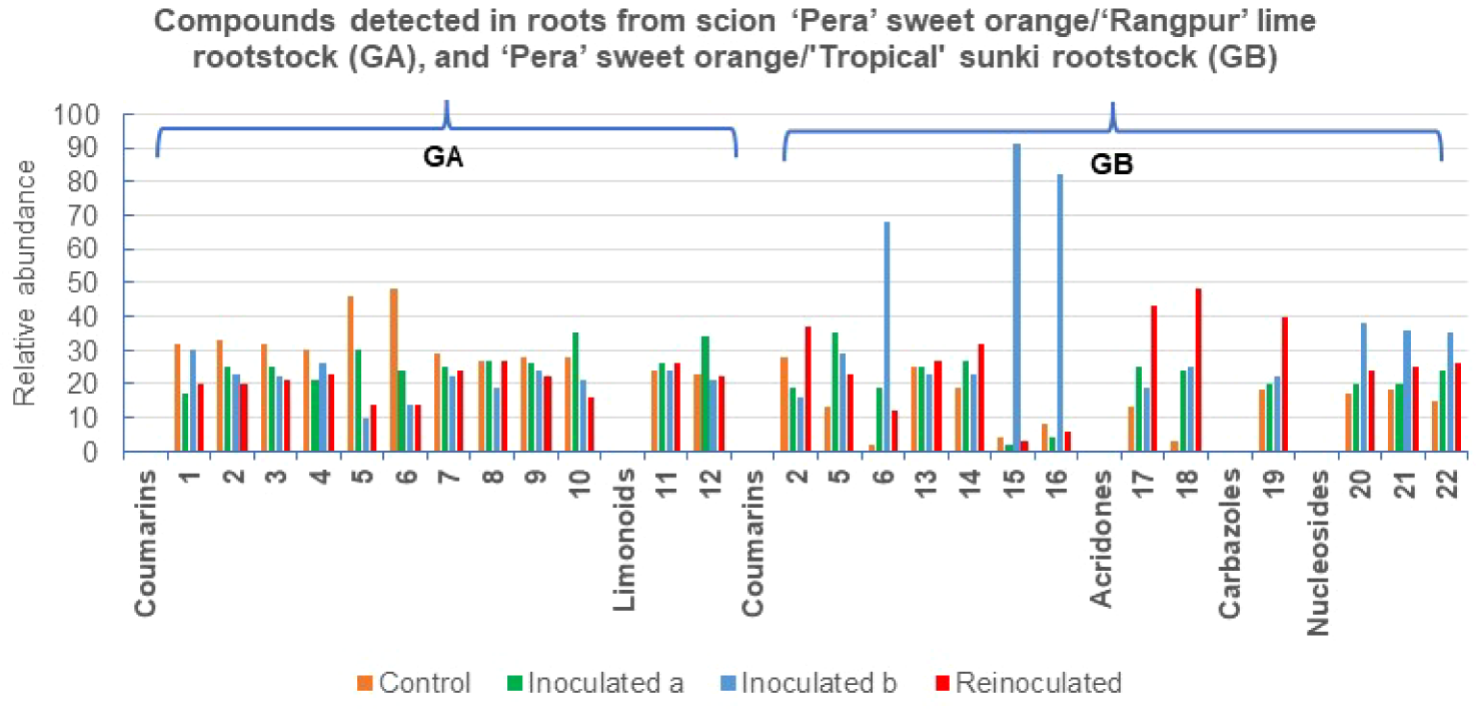
Metabolites annotated in the fractions from roots of the grafts in GA and GB, by UHPLC-ESI-MS/MS; and the relative abundance of the compounds across the samples in %.

**Figure 6:**
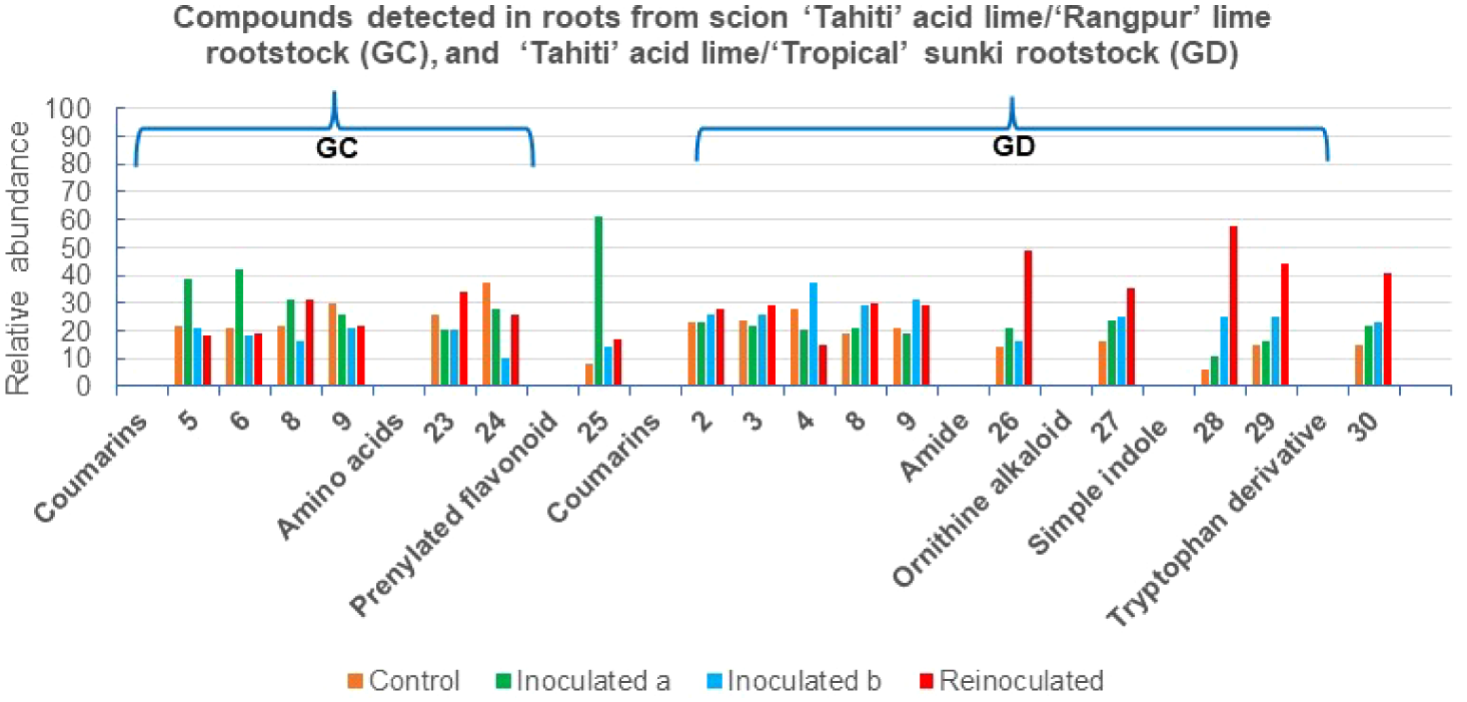
Metabolites annotated in the fractions from roots of the grafts in GC and GD, by UHPLC-ESI-MS/MS; and the relative abundance of the compounds across the samples in %.

**Figure 7:**
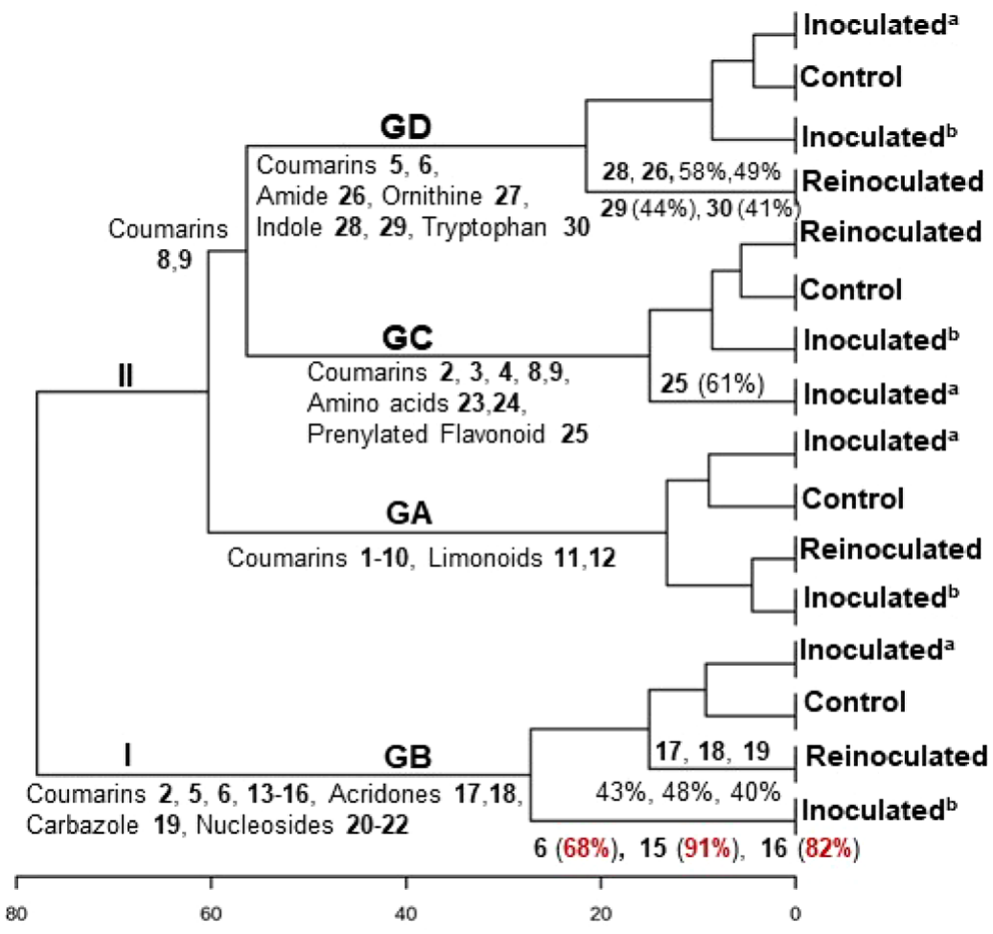
Hierarchical cluster analysis of the fractions from roots of the grafts in GA-GD, and the relative abundance of the compounds across the samples in %.

**Figure 8:**
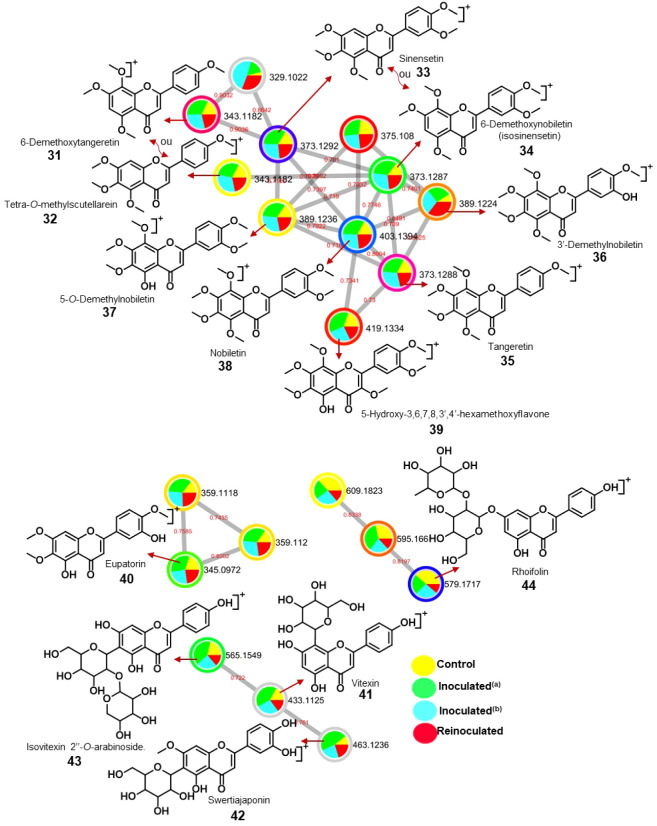
Molecular network of the analysis carried out in positive ion monitoring for leaves from scion “Pera” sweet orange/“Rangpur” lime rootstock (GA), control (yellow), inoculated^(a)^ (green), inoculated^(b)^ (blue), and reinoculated (red), and selected clusters with nodes showing the compounds annotated by GNPS library search.

**Figure 9:**
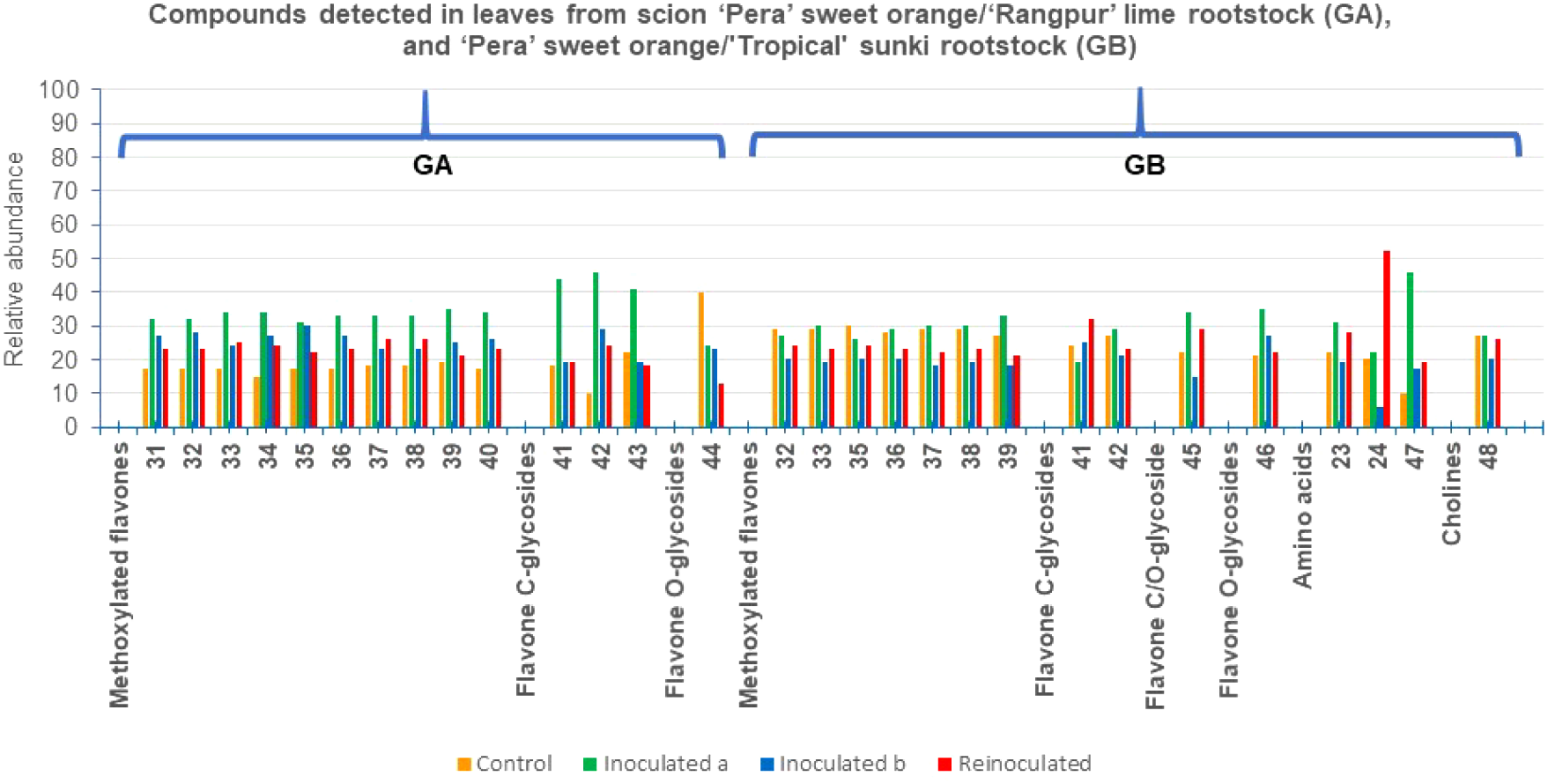
Metabolites annotated in the fractions from leaves of the grafts in GA and GB, by UHPLC-ESI-MS/MS; and the relative abundance of the compounds across the samples in %.

**Figure 10:**
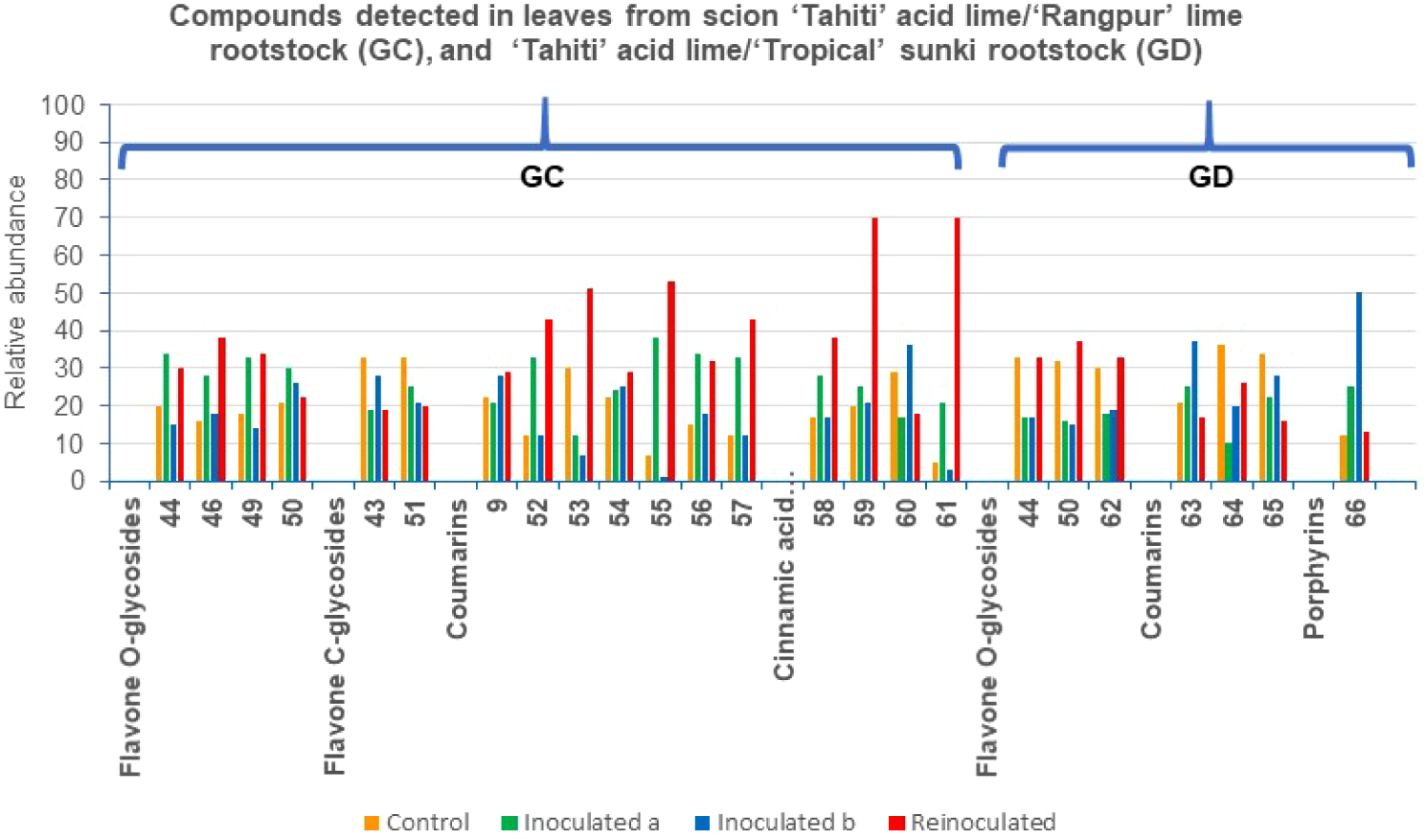
Metabolites annotated in the fractions from leaves of the grafts in GC and GD, by UHPLC-ESI-MS/MS; and the relative abundance of the compounds across the samples in %.

**Figure 11:**
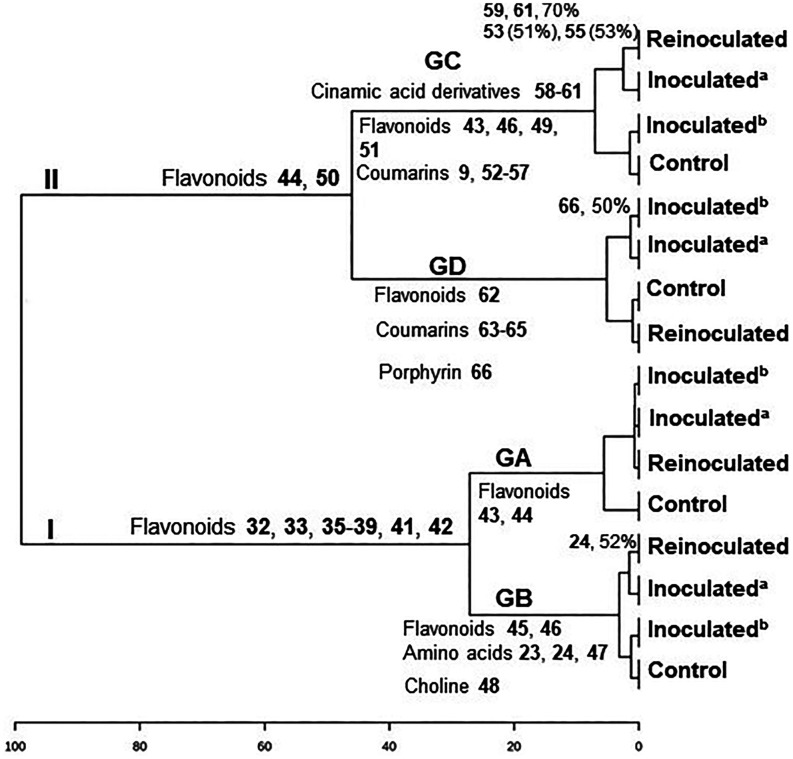
Hierarchical cluster analysis of the fractions from leaves of the grafts in GA-GD, and the relative abundance of the compounds across the samples in %.

**Figure 12:**
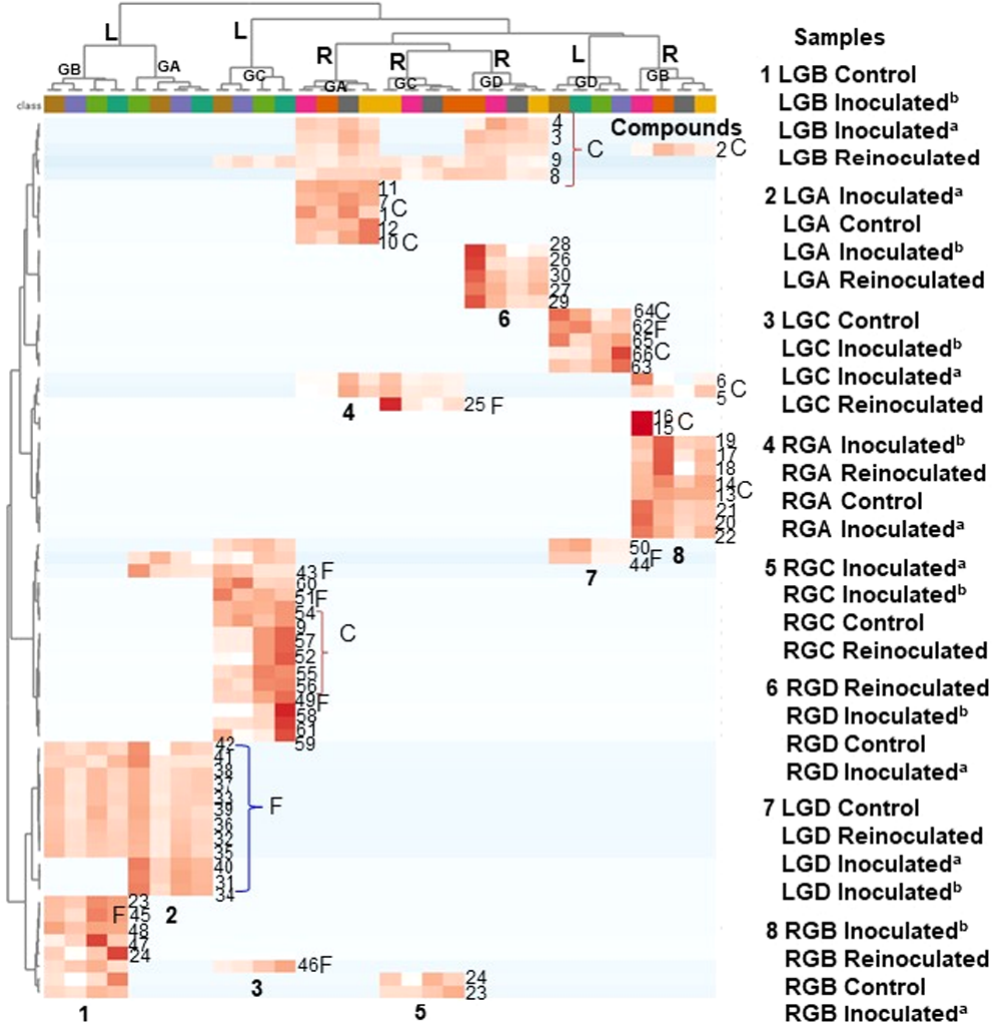
Heatmap showing the relative abundance of the identified compounds (**1**-**66**, C: Coumarins; F: Flavonoids) in each graft of GA-GD, compounds are found to be more concentrated (light red in color) in specific graft and less concentrated in others (light pink in color) with regards to their relative abundances.

Molecular networks generated under positive ionization mode for control, inoculated^(a)^, inoculated^(b)^, and reinoculated GA-GD roots and leaves (Figures 4, 8 and S1–S6) enabled the detection of infection-induced metabolites. Node sizes within these networks reflect the cumulative peak areas of detected compounds. In addition, bar plots were constructed to illustrate the relative abundance of metabolites across treatments (Figures 5, 6, 9, 10 and Table 1). To further evaluate the metabolic composition and explore chemical relationships among GA-GD grafts, hierarchical cluster analysis (HCA) was conducted using MetaboAnalyst version 6.0.^13^ The multivariate data set included the relative abundances of metabolites (Table 1), with GA-GD grafts categorized according to treatment condition: control, inoculated^(a)^, inoculated^(b)^, and reinoculated (Figures 7 and 11). For enhanced visualization, a heatmap was also generated using the same software. This heatmap (Figure 12) displays compound distribution across the different treatment groups, with higher and lower abundances indicated by light red and light pink, respectively.

**Table 1:** Metabolites Annotated from the Fractions of the grafts GA-GD, by UHPLC-ESI-MS/MS; and the Relative Abundance of the Compounds across the Samples in %

Compounds detected in roots from scion “Pera” sweet orange/“Rangpur” lime rootstock (GA)				
Coumarins	Control	Inoculated^(a)^	Inoculated^(b)^	Reinoculated
osthenol (**1**)	32	17	30	20
osthol (**2**)	33	25	23	20
auraptenol (**3)**	32	25	22	21
angenomalin (**4**)	30	21	26	23
nordentatin (**5**)	46	30	10	14
clausarin (**6**)	48	24	14	14
xanthoxyletin (**7**)	29	25	22	24
moriramulosid B (**8**)	27	27	19	27
haploperoside A (**9**)	28	26	24	22
moriramulosid B 6-glyceroyl (**10**)	28	35	21	16
Limonoids	Control	Inoculated^(a)^	Inoculated^(b)^	Reinoculated
limonin (**11**)	24	26	24	26
nomilin (**12**)	23	34	21	22
Compounds detected in roots from scion “Pera” sweet orange/“Tropical” sunki rootstock (GB)				
Coumarins	Control	Inoculated^(a)^	Inoculated^(b)^	Reinoculated
osthol (**2**)	28	19	16	37
nordentatin (**5**)	13	35	29	23
clausarin (**6**)	1	19	68	12
umbelliferone (**13**)	25	25	23	27
citrubuntin (**14**)	19	27	23	32
seselin (**15**)	4	2	91	3
luvangetin (**16**)	8	4	82	6
Acridone alkaloids	Control	Inoculated^(a)^	Inoculated^(b)^	Reinoculated
5-hydroxynoracronicine (**17**)	13	25	19	43
citracridone III (**18**)	3	24	25	48
Carbazole alkaloid	Control	Inoculated^(a)^	Inoculated^(b)^	Reinoculated
clausine-L (**19**)	18	20	22	40
Nucleosides	Control	Inoculated^(a)^	Inoculated^(b)^	Reinoculated
cytidine (**20**)	17	20	38	24
guanosine (**21**)	18	20	36	25
adenosine (**22**)	15	24	35	26
Compounds detected in roots from scion “Tahiti” acid lime/“Rangpur” lime rootstock (GC)				
Coumarins	Control	Inoculated^(a)^	Inoculated^(b)^	Reinoculated
nordentatin (**5**)	22	39	21	18
clausarin (**6**)	21	42	18	19
moriramulosid B (**8**)	22	31	16	31
haploperoside A (**9**)	30	26	21	22
Amino acids	Control	Inoculated^(a)^	Inoculated^(b)^	Reinoculated
proline (**23**)	26	20	20	34
arginine (**24**)	37	28	10	26
Prenylated flavonoid	Control	Inoculated^(a)^	Inoculated^(b)^	Reinoculated
lochnocarpol A (**25)**	8	61	14	17
Compounds detected in roots from scion “Tahiti” acid lime/“Tropical” sunki rootstock (GD)				
Coumarins	Control	Inoculated^(a)^	Inoculated^(b)^	Reinoculated
osthol (**2**)	23	23	26	28
Compounds detected in roots from scion “Tahiti” acid lime/“Tropical” sunki rootstock (GD)				
Coumarins	Control	Inoculated^(a)^	Inoculated^(b)^	Reinoculated
auraptenol (**3)**	24	22	26	29
angenomalin (**4**)	28	20	37	15
moriramulosid B (**8**)	19	21	29	30
haploperoside A (**9**)	21	19	31	29
Amide	Control	Inoculated^(a)^	Inoculated^(b)^	Reinoculated
amygdalin amide (**26**)	14	21	16	49
Ornithine alkaloid	Control	Inoculated^(a)^	Inoculated^(b)^	Reinoculated
stachydrine (**27**)	16	24	25	35
Simple indole derivatives	Control	Inoculated^(a)^	Inoculated^(b)^	Reinoculated
*N*,*N*-dimethyltryptamine (**28**)	6	11	25	58
*N*-hexanoyltryptamine (**29**)	15	16	25	44
Tryptophan derivative	Control	Inoculated^(a)^	Inoculated^(b)^	Reinoculated
5-methoxytryptophan (**30**)	15	22	23	41
Compounds detected in leaves from scion “Pera” sweet orange/“Rangpur” lime rootstock (GA)				
Polymethoxylated flavones	Control	Inoculated^(a)^	Inoculated^(b)^	Reinoculated
6-demethoxytangeretin (**31**)	17	32	27	23
tetra-*O-*methylscutellarein (**32**)	17	32	28	23
sinensetin (**33**)	17	34	24	25
6-demethoxynobiletin (isosinensetin) (**34**)	15	34	27	24
tangeretin (**35**)	17	31	30	22
3′-demethylnobiletin (**36**)	17	33	27	23
5-*O*-demethylnobiletin (**37**)	18	33	23	26
nobiletin (**38**)	18	33	23	26
5-hydroxy-3,6,7,8,3′,4’-hexamethoxyflavone (**39**)	19	35	25	21
eupatorin (**40**)	17	34	26	23
Flavone C-glycosides	Control	Inoculated^(a)^	Inoculated^(b)^	Reinoculated
vitexin (apigenin-8-C-glucoside) (**41**)	18	44	19	19
swertiajaponin (**42**)	10	46	29	24
isovitexin 2’’-*O*-arabinoside (**43**)	22	41	19	18
Flavone O-glycosides	Control	Inoculated^(a)^	Inoculated^(b)^	Reinoculated
rhoifolin (**44**)	40	24	23	13
Compounds detected in leaves from scion “Pera” sweet orange/“Tropical” sunki rootstock (GB)				
Polymethoxylated flavones	Control	Inoculated^(a)^	Inoculated^(b)^	Reinoculated
tetra-*O-*methylscutellarein (**32**)	29	27	20	24
sinensetin (**33**)	29	30	19	23
tangeretin (**35**)	30	26	20	24
3′-demethylnobiletin (**36**)	28	29	20	23
5-*O*-demethylnobiletin (**37**)	29	30	18	22
nobiletin (**38**)	29	30	19	23
5-hydroxy-3,6,7,8,3′,4’-hexamethoxyflavone (**39**)	27	33	18	21
Flavone C-glycosides	Control	Inoculated^(a)^	Inoculated^(b)^	Reinoculated
vitexin (apigenin-8-C-glucoside) (**41**)	24	19	25	32
Flavone C-glycosides	Control	Inoculated^(a)^	Inoculated^(b)^	Reinoculated
swertiajaponin (**42**)	27	29	21	23
Flavone C/O-glycoside	Control	Inoculated^(a)^	Inoculated^(b)^	Reinoculated
saponarin (**45**)	22	34	15	29
Flavone O-glycosides				
hesperidin (**46**)	21	35	27	22
Amino acids	Control	Inoculated^(a)^	Inoculated^(b)^	Reinoculated
proline (**23**)	22	31	19	28
arginine (**24**)	20	22	6	52
aspartic acid (**47**)	10	46	17	19
Cholines	Control	Inoculated^(a)^	Inoculated^(b)^	Reinoculated
choline (**48**)	27	27	20	26
Compounds detected in leaves from scion “Tahiti” acid lime/“Rangpur” lime rootstock (GC)				
Flavone O-glycosides	Control	Inoculated^(a)^	Inoculated^(b)^	Reinoculated
rhoifolin (**44**)	20	34	15	30
hesperidin (**46**)	16	28	18	38
luteolin-7-*O*-rutinoside (**49**)	18	33	14	34
kaempferide 3-*O*-β-d-glucopyranoside 7-*O*-α-L-rhamnopyranoside (**50**)	21	30	26	22
Flavone C-glycosides	Control	Inoculated^(a)^	Inoculated^(b)^	Reinoculated
isovitexin 2’’-*O*-arabinoside (**43**)	33	19	28	19
isovitexin (**51**)	33	25	21	20
Coumarins	Control	Inoculated^(a)^	Inoculated^(b)^	Reinoculated
haploperoside A (**9**)	22	21	28	29
coumarin (**52**)	12	33	12	43
herniarin (**53**)	30	12	7	51
limettin (**54**)	22	24	25	29
psoralen (**55**)	7	38	1	53
bergaptol (**56**)	15	34	18	32
xanthotoxol (**57**)	12	33	12	43
Cinnamic acid derivatives	Control	Inoculated^(a)^	Inoculated^(b)^	Reinoculated
4-hydroxycinnamic acid (**58**)	17	28	17	38
3-*O*-*p*-coumaroylquinic acid (**59**)	20	25	21	70
coumaroylputrescine (**60**)	29	17	36	18
*p*-coumaroylglucose (**61**)	5	21	3	70
Compounds detected in leaves from scion “Tahiti” acid lime/“Tropical” sunki rootstock (GD)				
Flavone O-glycosides	Control	Inoculated^(a)^	Inoculated^(b)^	Reinoculated
rhoifolin (**44**)	33	17	17	33
kaempferide 3-*O*-β-d-glucopyranoside 7-*O*-α-L-rhamnopyranoside (**50**)	32	16	15	37
kaempferol-3-O-rutinoside (**62**)	30	18	19	33
Coumarins	Control	Inoculated^(a)^	Inoculated^(b)^	Reinoculated
oxypeucedanin (**63**)	21	25	37	17
oxypeucedanin hydrate (**64**)	36	10	20	26
bergamottin (**65**)	34	22	28	16
Porphyrins	Control	Inoculated^(a)^	Inoculated^(b)^	Reinoculated
pheophorbide A (**66**)	12	25	50	13

Collectively, Table 1 and Figures 3–12 highlight metabolic distinctions between leaves and roots under various treatments, revealing metabolites likely involved in the defense response against *P. citrophthora*, as discussed in the following sections.

### 3.2 Effect of *P. citrophthora* on the Chemical Profiles of Roots from “Pera” Sweet Orange Grafted on “Rangpur” Lime (GA)

The molecular network analysis for roots from control (GA), inoculated^(a)^ (GA), inoculated^(b)^ (GA), and reinoculated (GA) (Figure 4) revealed five clusters, four of which contained compounds previously identified in *C. sinensis* (“Pera” sweet orange) grafted on *C. limonia* (“Rangpur” lime) by our research group.^4^ Clusters 1–3 comprised coumarins with increasing structural complexity, from 8-prenylated to pyranocoumarins. Osthenol (**1**), osthol (**2**), auraptenol (**3**; [*m*/*z* = M+H–H_2_O]), and angenomalin (**4**) were grouped in Cluster 1. The pyranocoumarins nordentatin (**5**) and clausarin (**6**) were found in Cluster 2, while xanthoxyletin (**7**) was detected in Cluster 3. Unusual coumarin glycosides were identified in Cluster 4, including moriramulosid B (**8**), haploperoside A (**9**), and moriramulosid B 6-glyceroyl (**10**), the latter appearing to be a novel compound. Limonoids limonin (**11**) and nomilin (12) were annotated in Cluster 5 (Figure 4).

The nomenclature of simple coumarin glycosides based on the umbelliferone nucleus (7-hydroxycoumarin) is often ambiguous. To confirm the structures correlated with haploperosides A–D, we consulted reference materials. Haploperoside A and B,^14,15^ originally isolated from *Haplophyllum perforatum* (Rutaceae), were later revised.^16^ Haploperoside A corresponds to 6-methoxy-7-[O-(α-L-rhamnopyranosyl)-(1→6)-β-d-glucopyranosyloxy]-2H-benzopyran-2-one (**9**), while haploperoside B is 7-[O-(4”-O-acetyl-α-L-rhamnopyranosyl)-(1→6)-β-d-glucopyranosyloxy]-6-methoxy-2H-benzopyran-2-one. The known coumarin glycoside moriramulosid B (**8**), originally reported from *Morus alba* L. (Moraceae),^17^ requires revision of its naming. The authors placed the glycosyl substituent at C-7, but its name suggests attachment at C-6. As all simple coumarins are based on the umbelliferone nucleus, the correct name for moriramulosid B (**8**) is 7-{[6-O-(6-deoxy-α-L-mannopyranosyl)-β-d-glucopyranosyl]­oxy}-2H-1-benzopyran-1-one, with a synonym of 7-[O-(α-L-rhamnopyranosyl)-(1→6)-β-d-glucopyranosyloxy]-2H-benzopyran-2-one (**8**).

A plausible fragmentation pathway was proposed for haploperoside A (**9**) and moriramulosid B (**8**) (Schemes S8–S9). In the positive ionization mode, both exhibited sodium cluster ions, as expected for glycosylated compounds, which tend to cluster with metal cations such as sodium or potassium.^18^ The mass spectrum fragment corresponding to sugar moiety elimination suggested two possible glycosylation sites: 6-O-(α-L-rhamnopyranosyl)-d-glucopyranose or 2-O-(α-L-rhamnopyranosyl)-d-glucopyranose. These sugars commonly glycosylate flavonoids in *Citrus*, as seen in hesperidin (**46**) and rhoifolin (**44**). Therefore, moriramulosid B (**8**) could exist as two isomers. The correct structure requires confirmation via NMR spectroscopy. Given that haploperoside A (**9**) from *Haplophyllum* (Rutaceae) contains 6-O-(α-L-rhamnopyranosyl)-d-glucopyranose, we hypothesize a similar structure for moriramulosid B (**8**), necessitating further isolation and NMR analysis. In contrast, no sodium cluster ion was observed for moriramulosid B 6-glyceroyl (**10**) (Scheme S10). MS/MS data revealed an additional 91 mass units, suggesting a glyceroyl moiety at C-6. The fragmentation pattern, which includes losses of two neutral H_2_O molecules and 1,2-dihydro-rhamnopyranosyl at *m*/*z* 379, supports the proposed structure for moriramulosid B 6-glyceroyl (**10**).

The 8-prenylated coumarin osthenol (**1**) has been reported in *C. medica*,^19^ angenomalin (**4**) in *Boenninghausenia albiflora* and *Boronella aff. verticillate*, and auraptenol (**3**) in *C. aurantium*, *C. aurantium var. amara*, *C. aurantium var. natsudaidai*, and *C. sinensis*.^20^ The limonoid nomilin (**12**) has been identified in *C. grandis*, *C. reticulata*, *C. sinensis*, *C. mitis*, and *C. jambhiri*.^21^ Previously, we analyzed *C. sinensis* and *C. limonia* seedlings to compare their chemical profiles with those of grafted plants.^4^ Using LC-UV-MS/MS, we identified major chemical components in leaves, stems, rootstock stems, and roots of *C. sinensis* grafted onto *C. limonia* cv. Pêra. These data were instrumental in confirming the metabolomic profiles in control, inoculated^(a)^, inoculated^(b)^, and reinoculated GA samples in the present study, as all detected compounds had been previously verified using authentic standards.^4,22^

### 3.3 Metabolic Response of GA Grafts to Infection and Stress

Sample type information was incorporated to color the nodes as pie charts, representing the relative abundance of the compounds across the samples. The colors correspond to the different sample groups: controls (yellow), inoculated^(a)^ (green), inoculated^(b)^ (blue), and reinoculated extracts (red), as shown in Figure 4. Two pyranocoumarins, nordentatin (**5**, 46%) and clausarin (**6**, 48%), were observed at slightly higher concentrations in the control samples. The “Rangpur” lemon rootstock represents the group most sensitive to *Phytophthora* gummosis. Reinoculated plants showed no reduction in disease symptoms,^1^ suggesting that this rootstock does not stimulate citrus enzymes to enhance bioactive compound levels.

In a study on *Phytophthora* gummosis in citrus scion/rootstock combinations, Gesteira and colleagues reported that *P. citrophthora* infections impacted the stem tissues of citrus rootstocks, impairing water and nutrient uptake necessary for plant development.^23^ Osmotic adjustment is considered one of the critical processes in plant adaptation to drought and salinity, as it maintains turgor pressure at lower water potentials, sustains metabolic activity, and facilitates regrowth upon rehydration or reduction of salinity stress. The literature indicates that aliphatic polyols such as sorbitol, pinitol, and glycerol possess valuable osmotic properties, enabling plants to withstand high osmotic stress.^24,25^

Given that *Phytophthora*-induced gummosis often impairs water and nutrient uptake by damaging root tissues, the observed accumulation of glycerol-derived compounds may represent a physiological adaptation to mitigate drought stress. Glycerol is a well-established osmoprotectant that contributes to the maintenance of turgor pressure and cellular homeostasis under dehydration conditions. Its conjugation with phenolic compounds, such as coumarins, which are particularly abundant in citrus roots, may serve a dual role: (i) facilitating the storage or compartmentalization of osmolytes and (ii) detoxifying reactive intermediates generated during infection. We therefore propose that moriramuloside B 6-glyceroyl functions as a metabolite involved in osmotic adjustment, linking abiotic stress signaling (specifically dehydration) to the secondary metabolism associated with plant defense. This response suggests an integrative metabolic strategy against infection, potentially shaped by the high susceptibility of the “Rangpur” lime rootstock, which may constrain the broader activation of enzymatic defense pathways.

Experimental evidence supports this, moriramuloside B 6-glyceroyl (**10**) increase 35% in concentration in inoculated^(a)^ samples, as shown in bar plot in Figure 5 and Table 1. In the control plants, a culture medium disc without *Phytophthora* was placed, and the same procedure was followed to protect the disc insertion site. Plants respond to injury by signaling damage and secreting protective materials to safeguard the affected area. Control plants when wounded to insert the culture medium without the fungus, may have released glycerol to regulate osmotic pressure and prevent water loss. This could explain why moriramuloside B 6-glyceroyl (**10**) was also detected in fractions obtained from the control plants.

However, compound **10** has not been previously reported in *Citrus* species, suggesting that the “Pera” sweet orange/“Rangpur” lime should be further examined to isolate and characterize this compound for confirmation. Additionally, further investigations on “Pera” sweet orange/“Rangpur” lime will be essential to isolate the compound detected for the first time in this graft and to evaluate its in vitro activity against *P. citrophthora*. However, its role in protecting plants against this oomycete in vivo remains unclear.

### 3.4 Effect of *P. citrophthora* on the Chemical Profiles of Roots from “Pera” Sweet Orange Grafted onto “Tropical” Sunki (GB)

The “Pera” sweet orange/“Tropical” sunki combination consists of two varieties that exhibit significantly lower sensitivity to *Phytophthora* gummosis. The molecular network analysis for roots from the GB group (Figure S1) were predominantly characterized by the presence of coumarins (**2**, **5**, **6**, **13**-**16**). Cluster 3 contained coumarin along with three alkaloids (**17**-**19**), while cluster 5 was an exception, featuring primary metabolites such as the nucleosides cytidine (**20**), guanosine (**21**), and adenosine (**22**). In clusters 1 and 2, the simplest biogenetic representatives of the coumarins, namely umbelliferone (**13**), osthol (**2**), and citrubuntin (**14**), were identified. Notably, cluster 2 distinguished itself by the presence of the angular pyranocoumarin seselin (**15**), whose concentration increased by 91% in the inoculated^(b)^ (GB) group (Figure 5), suggesting a potential defense response against *P. citrophthora*. The linear pyranocoumarins luvangetin (**16**) in cluster 3 and clausarin (**6**) in cluster 4 also exhibited significant increases in the inoculated^(b)^ (GB) group, rising by 82% and 68%, respectively (Figure 5). This pattern suggests that both angular and linear pyranocoumarins, in order of decreasing activity, may play a role in plant-pathogen interactions, potentially acting as phytoanticipins.

Three alkaloids detected in cluster 3 may contribute to resistance in the reinoculated (GB) group, as their concentrations increased by 40% to 48%. The acridone alkaloid citracridone III (**18**) showed a 48% increase, while 5-hydroxynoracronycine (**17**) increased by 43% (Figure 5). The latter was previously isolated from *C. sinensis* grafted onto *C. limonia* by our group.^4,22^ These acridones have also been reported in Yalaha hybrid seedlings derived from a cross between Duncan grapefruit (*C. paradisi* Macf.) and Dancy tangerine (*C. tangerina* Hort. ex Tanaka).^26^ Citracridone III (**18**) was detected for the first time in *C. sunki* rootstock, likely functioning as a phytoalexin.

The acridone skeleton is biosynthesized through the sequential chain extension of *N*-methylanthraniloyl-CoA, yielding the corresponding 1,3-dihydroxy-*N*-methylacridone alkaloid.^27^ Given its structural framework, the nomenclature of acridones should consistently reflect biosynthetic origins. The aromatic rings are numbered conventionally, whereas the pyran ring is assigned primed numbers. The ring derived from the acetate pathway, typically depicted on the right, is designated as 1,3-dioxy (numbered from the first hydroxyl group), while the left ring is numbered starting from the nitrogen heteroatom (positions 5–8). This nomenclature can be ambiguous, as seen in the case of 5-hydroxynoracronycine (**17**), which is also referred to as 11-hydroxynoracronycine in the literature.

The detection of the carbazole alkaloid (**19**) in the roots of “Pera” sweet orange grafted onto “Tropical” sunki (*C. sunki*) is not unexpected, as related compounds are also known from *Murraya*, *Clausena*, and *Glycosmis*, all classified under the Aurantioideae subfamily alongside *Citrus*. The biosynthesis of carbazole alkaloids in higher plants remains incompletely understood and lacks substantial experimental validation. However, the anthranilic acid pathway, proceeding via 3-prenylquinolone, is the most widely accepted mechanism. In contrast, carbazole alkaloids isolated from microorganisms typically lack a carbon substituent at C-3 and are derived from *L*-tryptophan.^27,28^ The low concentration of clausine-L (**19**) may explain why carbazole alkaloids have remained largely undiscovered in *Citrus* or suggest its role as a phytoalexin in response to *P. citrophthora* infection. Clausine-L (**19**) was previously isolated and characterized from the leaves of *Clausena excavata* and the roots of *C. wallichii*.^29,30^

To date, no records exist of the aforementioned nucleosides being isolated from *Citrus*. The only documented nucleotides were extracted from the fruit of “Orlando” tangelo (*Citrus reticulata* x *C. paradisi*) and the “Hamlin” and “Pineapple” sweet oranges (*C. sinensis*), where cytidine, guanosine, and adenosine were detected in mono-, di-, or triphosphate forms in juice vesicles and were found to influence flavor.^31^

### 3.5 Effect of *P. citrophthora* on the Chemical Profiles of Roots from “Tahiti” Acid Lime/“Rangpur” Lime (GC)

The “Tahiti” acid lime/“Rangpur” lime (GC) combination consists of two varieties highly susceptible to *Phytophthora* gummosis. The molecular network analysis for roots from the GC group revealed chemical families similar to those detected in the “Pera” sweet orange/“Rangpur” lime (GA), as expected, given that both share the same rootstock. Simple glycosylated coumarins (**8**, **9**) were identified in cluster 1, while pyranocoumarins **5** and **6** appeared in cluster 2 (Figure S2). Cluster 3 contained only primary metabolites, such as the amino acids proline (**23**) and arginine (**24**). Proline (**23**), due to its osmotic properties, may contribute to water retention in the plant, aiding in adaptation to stress caused by *P. citrophthora*.^24,25^

Cluster 4 lacked coumarins and was instead characterized by the prenylated flavonoid lochnocarpol A (**25**). The first report of the flavanone lochnocarpol A was from *Lonchocarpus minimiflorus* (Fabaceae);^32^ however, its occurrence in *C. sinensis* roots is mentioned only in a review that does not cite the original reference.^33^ The detection of lochnocarpol A (**25**) in the roots of “Tahiti” acid lime/“Rangpur” lime provides evidence for its role as a biosynthetic precursor of the flavonoids erythrisenegalone (**25a**) and limonianin (**25b**), which were previously isolated from *C. limonia* seedlings germinated by our group.^22^ The increased concentration of lochnocarpol A (**25**) in inoculated^(a)^ (GC) samples, as shown in bar plot at Figure 6 (Table 1) suggests that its accumulation may be induced by *P. citrophthora* infection. The subsequent decrease in its concentration in inoculated^(b)^ and reinoculated samples could be due to its conversion into flavonoids **25a** and **25b**, which were not detected, indicating the need for further investigation of these fractions.

### 3.6 Effect of *P. citrophthora* on the Chemical Profiles of Roots from “Tahiti” Acid Lime/“Tropical” Sunki (GD)

In the “Tahiti” acid lime/“Tropical” sunki (GD) combination, the scion is highly sensitive, while the rootstock exhibits significantly lower sensitivity to *Phytophthora* gummosis. The molecular network analysis for roots from the GD group revealed a relatively low presence of coumarins, with only simple glycosylated coumarins (**8** and **9**) and precursors of angular pyranocoumarins (**2**, **3**, and **4**) detected in clusters 1 and 2 (Figure S3). Chemically, the “Tropical” sunki rootstock in this graft (GD) differs from its profile in the “Pera”/“Tropical” sunki graft (GB), resembling instead the coumarin profile characteristic of the “Rangpur” lime rootstock in GA (Figure 4). These differences in chemical profiles may be attributed to scion influence within the graft. While sample misidentification could be considered, the unique presence of specific classes of substances in clusters 3 and 4 of GD, which are absent in other grafts, strongly supports the distinct identity of these samples. Furthermore, the concentration of all detected coumarins remained unchanged in the inoculated^(a)^, inoculated^(b)^, and reinoculated extracts (Figure 6), suggesting that these coumarins do not play a protective role in the plant-pathogen interaction.

The only detected metabolite in cluster 3, 2-phenyl-2-[6-O-glucopyranosyl-(1→6)-glucopyranosyloxy]-ethylacetamide (amygdalin amide **26**), exhibited a significant increase (49%) in the reinoculated fraction (Figure 6), suggesting a potential defense response against *P. citrophthora*. Amygdalin amide (**26**) has not been previously reported in *Citrus* species. However, its structural analogue, 2-phenyl-2,4’-dihydroxy-ethylamine (synephrine), is a well-documented compound in *C. aurantium*, *C. reticulata*, *C. sinensis*, *C. limon*, and *C. limonia*.^34^ The presence of amygdalin amide (**26**) has been reported in *Prunus persica* (Rosaceae) flowers,^35,36^ and other phenylethylamines have also been described in *Citrus* species.^34^ Cluster 4 is characterized by alkaloids derived from ornithine and tryptophan, including stachydrine (**27**), simple indole derivatives [*N,N*-dimethyltryptamine (**28**) and *N*-hexanoyltryptamine (**29**)], and 5-methoxytryptophan (**30**). The pyrrolidine alkaloid stachydrine (**27**) has been previously reported in several *Citrus* species.^37^ *N,N*-Dimethyltryptamine (**28**) has also been identified in multiple *Citrus* species and is known to influence insect feeding and reproduction.^38^ The presence of *N,N*-dimethyltryptamines (**28**) and their 5-hydroxylated derivatives in *Citrus* suggests the involvement of tryptophan and 5-hydroxytryptophan decarboxylases in their biosynthesis, with 5-hydroxytryptophan, or 5-methoxytryptophan (**30**) acting as an intermediate.^38^

*Phytophthora citrophthora* likely induced an increase in tryptophan-derived metabolites in reinoculated plants, with *N,N*-dimethyltryptamine (**28**) increasing by 58%, *N*-hexanoyltryptamine (**29**) by 44%, and 5-methoxytryptophan (**30**) by 41% (Figure 6).^38^ The marked increase in 5-methoxytryptophan (**30**), detected for the first time in plants, suggests its potential role as a phytoalexin, a key component of plant defense mechanisms. The alkaloid stachydrine (**27**) increased by 35% in reinoculated plants, further indicating a potential role in inducible chemical defense. The amino acid derivative 5-methoxytryptophan (**30**) appears to be a novel compound in *Citrus*. However, a metabolomic study evaluating the quality of *Pinellia ternata* tubers (Araceae), a native Chinese medicinal herb, identified several organic acids, including 5-methoxy-L-tryptophan.^39^ While *N*-hexanoyltryptamine (**29**) appears to be a new naturally occurring compound, its synthetic derivative has been documented in the literature. In the search for antipsychotic agents, a novel indole alkaloid with an *N*-propionyl side chain was isolated from the marine bacterium *Pseudoalteromonas rubra*, and the synthetic compound 6-bromo-*N*-hexanoyltryptamine exhibited significant bioactivity.^40^

### 3.7 Mechanism of Induction of Coumarins, Amines, Alkaloids, and Limonoids in GA-GD Roots

The hierarchical cluster analysis (HCA) of the chemical composition of GA-GD grafts separated them into two main groups (Figure 7). The relative abundance of compounds across samples was identified as a discriminant factor for the two groups. Group I (GB) differed from all other grafts in Group II due to the presence of angular and linear pyranocoumarins, which accounted for more than 90% of the total compound abundance. The HCA distinctly separated the GB graft, showing the highest values. Compared to the other grafts, the “Tropical sunki” rootstock exhibited an increased pyranocoumarin content. The concentration of angular pyranocoumarin seselin (**15**, 91%) and linear pyranocoumarins luvangetin (**16**, 82%) and clausarin (**6**, 68%) significantly increased in inoculated^(b)^ (GB) (Figure 7). The increased pyranocoumarin content in the rootstock may be associated with induced internal defense mechanisms. Since the “Pera” sweet orange/“Tropical sunki” graft consists of two varieties that are less sensitive to *Phytophthora* gummosis, it can be proposed that the rootstock stimulates citrus enzymes to increase the levels of bioactive compounds. This supports the hypothesis that angular and linear pyranocoumarins play a role in plant-pathogen interactions, likely acting as phytoanticipins.

These findings suggest that “Tropical sunki” is the most suitable rootstock for sweet orange, as it demonstrated the highest chemical response percentages for coumarins **6** (68%), **15** (91%), and **16** (82%), followed by alkaloids acridones **17** (43%), **18** (48%), and carbazole **19** (40%). The alkaloids detected conferred resistance in reinoculated (GB) plants: 5-hydroxynoracronicine (**17**) as a phytoanticipin, and citracridone III (**18**) and clausine-L (**19**) as phytoalexins. Moreover, this chemical profile likely inhibits oomycete development, as GB grafts exhibited smaller stem lesions compared to those in GA and GC groups. Therefore, the compounds whose concentrations increased in response to *P. citrophthora* should be isolated for further testing to elucidate their mechanisms as oomycete growth inhibitors.

Group II was further divided into three subgroups: GA, GC, and GD, with an early separation of the GA samples. This group corresponds to the “Pera” sweet orange/“Rangpur” lime rootstock. Since “Rangpur” lime is highly sensitive to *Phytophthora* gummosis,^1^ it is plausible that this rootstock does not stimulate citrus enzymes to enhance bioactive compound production. Notably, the concentration of all detected coumarins (**1**-**10**) and limonoids (**11**, **12**) did not increase in the analyzed extracts. Two pyranocoumarins, nordentatin (**5**, 46%) and clausarin (**6**, 48%), were observed at slightly higher concentrations in the control samples.

The GC and GD subgroups contained coumarins **8** and **9**. GC was characterized by relatively low concentrations of all detected compounds. This subgroup corresponds to the “Tahiti” acid lime/“Rangpur” lime rootstock, both of which are highly sensitive to *Phytophthora* gummosis. The molecular network of GC revealed chemical families similar to those found in “Pera” sweet orange/“Rangpur” lime (GA), as expected given the identical rootstocks. The increased concentration of flavanone lochnocarpol A (**25**, 61%) in inoculated^(a)^ samples suggests its induction in response to *P. citrophthora* infection, functioning as a phytoalexin (Figure 7).

GD corresponds to the “Tahiti” acid lime/“Tropical” sunki rootstock, where the scion is highly sensitive and the rootstock is significantly less sensitive to *Phytophthora* gummosis. This again demonstrates that “Tropical sunki” provides better protection against this oomycete. The rootstock confers resistance by activating phenylalanine, tryptophan, and ornithine pathway enzymes, leading to the production of simple derivatives that defend the plant against oomycetes. This activation was more pronounced in reinoculated plants. Amygdalin amide (**26**), *N*-hexanoyltryptamine (**29**), and 5-methoxytryptophan (**30**) exhibited properties characteristic of phytoalexins, while stachydrine (**27**) and *N,N*-dimethyltryptamine (**28**) acted as phytoanticipins, collectively serving as major protective agents in plants (Figure 7).

These findings suggest that “Tropical sunki” is also the optimal rootstock for “Tahiti” acid lime, as it activated alkaloid biosynthetic pathways leading to increased concentrations of compounds **26** (49%), **27** (35%), **28** (58%), **29** (44%), and **30** (41%) in reinoculated plants. These compounds will be isolated and subjected to in vivo and in vitro bioassays against *P. citrophthora*.

Comparison of the chemical profiles of the two grafts, “Pera”/“Tropical sunki” (GB) and “Tahiti” acid lime/“Tropical sunki” (GD), indicates that the scion also influences rootstock metabolism. Despite having the same rootstock, the two grafts exhibited different biosynthetic responses to oomycete presence. GB predominantly activated the biosynthetic pathways of linear and angular pyranocoumarins, particularly in inoculated^(b)^ plants, while in reinoculated plants, the pathway shifted toward alkaloids derived from anthranilic acid (**17**, **18**, and **19**). Conversely, GD activated the pyranocoumarin precursor pathway at similar levels across all plants, but demonstrated higher activation of alkaloids derived from phenylalanine (**26**), tryptophan (**28**, **29**, and **30**), and ornithine (**27**) in reinoculated plants.

Studies by Gesteira’s group indicate that the initial perception of pathogens by plants can induce an enhanced state of defense activation against subsequent pathogen challenges.^1,23^ This general defense response mechanism is associated with systemic acquired resistance (SAR).^23^ The literature presents numerous examples indicating that stress memory is a complex cellular phenomenon. Beyond epigenetic modifications, it also involves changes in the regulation of metabolic pathways and broader physiological adjustments.^41^ Although the current chemical profiles do not directly confirm these findings, they suggest that GB and GD, by triggering the coumarin biosynthetic pathway during early infections (inoculation^(a)^, inoculation^(b)^), may have primed the activation of the alkaloid pathway in response to subsequent challenges by *P. citrophthora*.

Literature reports indicate that alkaloids produced in various plant tissues, such as leaves, roots, bark, and seeds, are transported to localized tissues to counteract different predators, including fungi, bacteria, and insect larvae. Several references highlight alkaloids with potent antimicrobial activity against various pathogenic microorganisms,^42^ supporting the hypothesis that the alkaloids present in GB and GD grafts contribute to plant defense.

### 3.8 Effect of *P. citrophthora* on the Chemical Profiles of Leaves from “Pera” Sweet Orange Grafted on “Rangpur” Lime (GA)

The molecular network analysis conducted in positive ion mode for leaves from control (GA), inoculated^(a)^ (GA), inoculated^(b)^ (GA), and reinoculated (GA) samples (Figure 8) enabled the identification of induced compounds. Four clusters were selected, two of which contained exclusively polymethoxylated flavones. Extensive *O*-methylation of highly oxygenated flavones is a characteristic feature largely confined to the Rutaceae (Aurantioideae) family.^43^ Cluster 1 included the polymethoxylated flavones 6-demethoxytangeretin (**31**), tetra-*O*-methylscutellarein (**32**), sinensetin (**33**), 6-demethoxynobiletin (isosinensetin) (**34**), tangeretin (**35**), 3′-demethylnobiletin (**36**), 5-*O*-demethylnobiletin (**37**), nobiletin (**38**), and 5-hydroxy-3,6,7,8,3′,4’-hexamethoxyflavone (**39**). In contrast, cluster 2 contained only eupatorin (**40**). Flavones **32**-**35**, **37**, and **38** were previously isolated from *C. sinensis* leaves grafted on *C. limonia* by our group.^4,22^ Flavones 3′-demethylnobiletin (**36**), 5-hydroxy-3,6,7,8,3′,4’-hexamethoxyflavone (**39**), and eupatorin (**40**) were previously reported from dried ripe pericarps of *C. reticulata*, *C. sinensis* peel, and *C. aurantium* L. leaves,^44–46^ respectively. Cluster 3 was characterized by flavone *C*-glycosides (**41**-**43**), while cluster 4 contained flavone *O*-glycoside (**44**). The flavone *C*-glycosides vitexin (apigenin-8-*C*-glucoside) (**41**), swertiajaponin (**42**), and isovitexin 2’’-*O*-arabinoside (**43**) were previously identified in *C. sinensis* juice,^47^ *C. limon* fruit pulp,^48^ and *C. sinensis* flavedos,^49^ respectively. Rhoifolin (**44**) was also identified in *C. sinensis* juice.^47^

The presence of fragment ions in the mass spectrum, associated with retro-Diels–Alder cleavage of the *C*-ring, clearly indicated the number of methoxy groups in the A- and B-rings. Therefore, two possible isomers were suggested for flavones **31** and **32**, as well as for **33** and **34**. The correct structures can be confirmed using NMR spectroscopy. Similarly, mass spectrometry confirmed the hydroxyl group position as being in ring B for **36** and in ring A for **37** and **39**. However, the precise position, whether at C-5 or C-7 in ring A (**37**, **39**) or at C-3′ or C-4’ in ring B (**36**) can only be determined through biosynthetic analysis. The same principle applies to flavone *C*- or *O*-glycosides, which require isolation and NMR analysis for full structural elucidation.

*Phytophthora* spp. is a soil-borne pathogen, with symptoms progressing from the tree base upward, affecting primary and secondary branches. Leaf symptoms include rib discoloration and yellowing. Although the infection begins in the lower part of the plant, the leaves also respond to the pathogen’s presence. Notably, the concentration of all detected flavonoids increased in inoculated^(a)^ extracts, suggesting a potential defense mechanism against *P. citrophthora*, except for rhoifolin (**44**), which decreased progressively from control (40%) to inoculated^(a)^ (24%), inoculated^(b)^ (23%), and reinoculated (13%) (Figure 9). The further increase in the flavone *C*-glycoside content **41**-**43** (44%, 46%, and 41%, respectively) in inoculated^(a)^ extracts suggests that *C*-glycosylation, rather than *O*-glycosylation, might play a role in the plant’s defense against *P. citrophthora*. Although these flavonoids have not been directly tested for antimicrobial activity, they may have potential applications in controlling *P. citrophthora*.

### 3.9 Effect of *P. citrophthora* on the Chemical Profiles of Leaves from “Pera” Sweet Orange Grafted on “Tropical” Sunki (GB)

The molecular network analysis in positive ion mode for leaves from control (GB), inoculated^(a)^ (GB), inoculated^(b)^ (GB), and reinoculated (GB) samples (Figure S4) revealed four clusters. In cluster 1, polymethoxylated flavones (**31**–**39**) were detected, the same as in “Pera” sweet orange grafted on “Rangpur” lime (GA), which was expected since both share the same scion. Cluster 2 contained flavone *C*-glycosides (**41**, **42**) and the flavone *C/O*-glycoside saponarin (**45**). The first two were also found in GA leaves, while saponarin (**45**) was previously identified in red (or blood) orange (*C. sinensis*) and grapefruit (*C. paradisi*) juices,^50^ as well as in *C. hystrix* leaves.^51^ Cluster 3 was characterized solely by the flavone *O*-glycoside hesperidin (**46**), which is abundant in citrus fruits such as orange (*C. sinensis*), grapefruit (*C. paradisi*), tangerine (*C. reticulata*), lime (*C. aurantiifolia*), and lemon (*C. limon*).^52^ Cluster 4 contained three amino acids, proline (**23**), arginine (**24**), and aspartic acid (**47**), as well as choline (**48**). Proline (**23**), arginine (**24**), and aspartic acid (**47**) were previously reported in grapefruit (*C. paradisi*), Valencia orange (*C. sinensis*), and lemon (*C. limetta*) leaves.^53^

The concentration of flavonoids remained relatively stable between control and inoculated^(a)^ samples but decreased in inoculated^(b)^ and reinoculated samples, except for aspartic acid (**47**) and arginine (**24**), which were detected in higher concentrations, 47% in inoculated^(a)^ and 52% in reinoculated samples, respectively (Figure 9). Under abiotic stress, aspartic acid (**47**) levels fluctuate depending on the plant species and type of stress.^54^ Its accumulation, along with other amino acids such as proline (**23**), which increased by 31% in inoculated^(a)^, is associated with osmotic adjustment and membrane stabilization under salinity stress. Aspartic acid (**47**) and proline (**23**) might contribute to water retention, aiding in the plant’s adaptation to *P. citrophthora*-induced stress. To date, there are no reports of choline (**48**) in *Citrus*. Despite its low concentration (20–27%) in “Pera” sweet orange leaves (GB), its osmotic properties suggest a potential role in pathogen response.^55^ Arginine (**24**) serves as a precursor for nitric oxide (NO), although its production mechanism in higher plants remains unclear. However, its conversion to polyamines is well-documented, and both NO and polyamines play crucial roles in regulating development and stress responses.^56^ Thus, arginine (**24**) may contribute to pathogen defense against *P. citrophthora*.

### 3.10 Effect of *P. citrophthora* on the Chemical Profiles of “Tahiti” Acid Lime/“Rangpur” Lime Leaves (GC)

The molecular network analysis performed in positive ion monitoring for leaves from control (GC), inoculated^(a)^ (GC), inoculated^(b)^ (GC), and reinoculated (GC) samples revealed four distinct clusters (Figure S5). The three smaller clusters were predominantly composed of glycosylated compounds, including two clusters of flavonoids and one cluster of coumarin. Cluster 1 contained three flavone *O*-glycosides, rhoifolin (**44**), luteolin-7-*O*-rutinoside (**49**), and kaempferide 3-*O*-β-d-glucopyranoside 7-*O*-α-L-rhamnopyranoside (**50**), along with one flavanone *O*-glycoside, hesperidin (**46**). The last flavonoid (**46**) has previously been isolated from “Tahiti” acid lime (*C. latifolia*) leaves,^57^ while rhoifolin (**44**) was identified in *C. sinensis* juice and luteolin-7-*O*-rutinoside (**49**) in *C. limon*.^47^ This study represents the first report of kaempferide 3-*O*-β-d-glucopyranoside 7-*O*-α-L-rhamnopyranoside (**50**) in *Citrus*, as it was previously isolated from *Cleome viscosa* L. (Capparaceae) leaves.^58^ Cluster 2 included two *C*-glycosylated flavonoids, isovitexin 2″-*O*-arabinoside (**43**) and isovitexin (**51**). Isovitexin 2″-*O*-arabinoside (**43**) was also detected in “Pera” sweet orange leaves grafted onto “Rangpur” lime, while isovitexin (**51**) has been previously isolated from bergamot (*Citrus bergamia* Risso).^59^ The third cluster contained an *O*-glycosylated coumarin, haploperoside A (**9**), detected as a sodium cluster ion, which was also found in the roots of grafts GA, GC, and GD.

Cluster 4 was distinct in that it lacked flavonoids and was primarily composed of coumarins and cinnamic acid derivatives. This cluster contained three simple coumarins, coumarin (**52**), herniarin (**53**), and limettin (citropten) (**54**), as well as three linear furocoumarins, psoralen (**55**), bergaptol (**56**), and xanthotoxol (**57**). Coumarins **54** and **55** were previously identified in *C. latifolia* leaves by our group.^60^ However, this is the first report of herniarin (**53**), bergaptol (**56**), and xanthotoxol (**57**) in this species. Herniarin (**53**) was earlier detected in *C. aurantifolia* essential oil,^61^ bergaptol (**56**) in citrus peels from *C. bergamia*, *C. grandis*, and *C. paradisi*,^62^ and xanthotoxol (**57**) in *C. micrantha* Wester fruits.^63^ Coumarin (**52**) itself may be an artifact of isolation procedures; however, there is evidence suggesting that plants naturally contain glucosides of (*E*)- and (*Z*)-2-coumaric acid, which release coumarin upon enzymatic hydrolysis and lactonization due to tissue damage during harvesting and processing. The major coumarin class originate from umbelliferone (7-hydroxycoumarin).^20^

Within Cluster 4, additional cinnamic acid derivatives were identified, *p*-hydroxycinnamic acid (**58**), 3-*O*-*p*-coumaroylquinic acid (**59**), coumaroylputrescine (**60**), and *p*-coumaroylglucose (1-*O*-*p*-coumaroyl-β-d-glucopyranose) (**61**). These compounds appear to be newly reported in *C. latifolia*. *Trans*-*p*-hydroxycinnamic acid (**58**) was previously isolated from blood and blond orange juices [*C. sinensis* (L.) Osbeck],^64^ while 3-*O*-*p*-coumaroylquinic acid (**59**) was identified in *C. reticulata* Blanco fruits.^65^ Coumaroylputrescine (**60**) and *p*-coumaroylglucose (**61**) are novel for *Citrus*, though feruloylputrescine has been detected in grapefruit (*C. paradisi* Macf.) leaves and juice.^66^

Studies on Solanaceae, Brassicaceae, and Poaceae indicate that phenolamide accumulation in response to microbial infection is a common defense mechanism. In genetically modified potato plants with enhanced phenolamide biosynthesis, increased levels of *p*-coumaroylagmatine, *p*-coumaroylputrescine, and caffeoylputrescine reduced *P. infestans* spore germination.^67,68^ Coumaroylglucose (**61**) has been previously isolated from black currant (*Ribes nigrum* L., Grossulariaceae) juice,^69^ marking its first report in *Citrus*.

Both *E*- and *Z*- cinnamic acid derivatives occur in plants, with the *E*-isomer being predominant and more stable. However, mass spectrometry used in routine biomolecule analysis cannot reliably distinguish between these isomeric forms. While direct hydroxylation of the aromatic ring is common, it generally occurs first at the *para* position (4-position) and subsequently at the ortho position. In contrast, hydroxylation in coumarins may occur ortho to the side-chain. The detected cinnamic acid derivatives (**58**-**61**) were assigned hydroxylation at the *para* position.

The chemical profile of “Tahiti” acid lime/“Rangpur” lime leaves differs from that of “Pera” sweet orange grafted onto “Rangpur” lime and “Tropical” sunki, primarily due to the absence of polymethoxylated flavones and the presence of coumarins. Notably, flavonoid concentrations exhibited minimal variation in response to *P. citrophthora* between control, inoculated^(a)^, and reinoculated samples. In contrast, coumarins and cinnamic acid derivatives displayed significant concentration changes in response to the oomycete, particularly herniarin (**53**) and psoralen (**55**), which increased by 51% and 53%, respectively, in the reinoculated samples (Figure 10). Furthermore, 3-*O*-*p*-coumaroylquinic acid (**59**) and coumaroylglucose (**61**) showed a 70% increase in reinoculated samples, suggesting a potential defensive role against *P. citrophthora*.

Simple coumarins and chlorogenic acids are known to exhibit antimicrobial properties.^70–72^ Herniarin (**53**) from chamomile (*Matricaria chamomilla*, Asteraceae) disrupts mitochondrial function and energy production in *Aspergillus niger*. Similarly, bergapten (*O*-methoxybergaptol), a derivative of bergaptol (**56**) found in *Ficus carica* (Moraceae), inhibits *P. infestans* mycelial growth and spore germination through DNA damage.^72^ Based on these findings, herniarin (**53**) and bergaptol (**56**) may play similar roles in defense against *P. citrophthora*.

### 3.11 Effect Of *P. citrophthora* on the Chemical Profiles of Leaves from “Tahiti” Acid Lime/“Tropical” Sunki (GD)

The molecular network analysis, performed under positive ion monitoring, revealed three distinct clusters among leaf samples from control (GD), inoculated^(a)^ (GD), inoculated^(b)^ (GD), and reinoculated (GD) groups (Figure S6). Cluster 1 contained three flavone *O*-glycosides, rhoifolin (**44)**, kaempferide 3-*O*-β-d-glucopyranoside 7-*O*-α-L-rhamnopyranoside (**50**), and kaempferol-3-*O*-rutinoside (**62**). The latter has previously been identified in fruits of *C. sinensis*, *C. reticulata*, *C. unshiu*, and *C. paradisi*.^47^ Cluster 2 consisted of three furocoumarins, two 5-*O*-prenylated compounds, oxypeucedanin (**63**) and oxypeucedanin hydrate (**64**), and one 5-geraniloxy compound, bergamottin (**65**). The last coumarin (**65**) was previously reported in *C. latifolia* leaves by our research group,^60^ while oxypeucedanin (**63**) and oxypeucedanin hydrate (**64**) were found in *C. latifolia* and *C. limon* fruits.^73^ Cluster 3 contained a single annotated compound, pheophorbide A (**66**), a porphyrin derivative and an intermediate product of chlorophyll degradation. Previous studies have demonstrated the antifungal properties of pheophorbide A. For instance, in cherry tomato (*Solanum lycopersicum* var. *cerasiforme*), it was shown to inhibit *Botrytis cinerea* by disrupting fungal cell structures and enhancing disease resistance-related enzyme activities.^74^ In most citrus species, fruit coloration results from the accumulation of chlorophylls and carotenoids. Chlorophylls are predominant in immature fruits, imparting a green hue to the peel. As fruits mature, chlorophyll levels decline rapidly, with pheophorbide A acting as an intermediate product in the degradation process.^75^

The concentration of all detected flavonoids fluctuated in response to *P. citrophthora* infection, initially decreasing from the control to the inoculated^(a)^ and inoculated^(b)^ groups, followed by an increase in the reinoculated group to levels comparable to the control. Notably, the reinoculated samples exhibited a significant increase in rhoifolin (**44**), kaempferide 3-*O*-β-d-glucopyranoside 7-*O*-α-L-rhamnopyranoside (**50**), and kaempferol-3-*O*-rutinoside (**62**) by 33%, 37%, and 33%, respectively (Figure 10; Table 1), suggesting a potential defense mechanism against *P. citrophthora*. The role of coumarins (**63**-**65**) in plant defense against this oomycete remains unclear. They do not appear to confer a protective effect, as the concentrations of oxypeucedanin hydrate (**64**) and bergamottin (**65**) decreased across all inoculated groups compared to the control, whereas oxypeucedanin (**63**) showed an increase in the inoculated^(b)^ group (ranging from 21% to 37%) (Figure 10; Table 1). Similarly, pheophorbide A (**66**) increased progressively from the control to the inoculated^(b)^ group but declined in the reinoculated group, with concentration variations of 12%, 25%, 50%, and 13%, respectively.

These findings suggest that the metabolic response of “Tahiti” acid lime/“Tropical” sunki leaves to *P. citrophthora* infection involves dynamic fluctuations in flavonoids, coumarins, and chlorophyll degradation intermediates, which may reflect adaptive defense mechanisms and physiological stress responses.

### 3.12 Mechanism of Induction of Flavonoids, Coumarins, Amino Acids, Choline, and Porphyrin in GA-GD Leaves

Hierarchical cluster analysis (HCA) revealed that the leaves of grafts in GA-GD were divided into two main groups based on the relative abundance of compounds across the samples (Figure 11). Group I consist of GA and GB grafts, primarily characterized by flavonoids, with relative abundances mostly below 50%. The distinguishing chemical classes between GB and GA include amino acids (**23**, **24**, and **47**) and a choline derivative (**48**) in GB; however, only the amino acid arginine (**24**, 52%) exceeded 50% abundance in the reinoculation experiments in GB. GA and GB share flavonoids **32**, **33**, **35**-**39**, **41**, and **42**. Group II comprises GC and GD grafts, which share flavonoids **44** and **50**. GC contains more flavonoids (**43**, **46**, **49**, **51**) than GD (**62**), and their coumarin profiles are distinct, GC features coumarins **9** and **52**-**57**, while GD contains coumarins **63**-**65**. Additionally, GC is distinguished by the presence of cinnamic acid derivatives (**58**-**61**), whereas GD is characterized by porphyrin (**66**). Notably, only two coumarins (**53**, **55**) and two cinnamic acid derivatives (**59**, **61**) exceeded 50% relative abundance (Figure 11).

The “Pera” sweet orange/Rangpur lime (GA) scion exhibits significantly lower sensitivity, whereas the rootstock is the most sensitive to *Phytophthora* gummosis.^1^ Only flavonoids were detected in the leaves of the scion, suggesting that this class of compounds may play a role in regulating defense mechanisms against *P. citrophthora*. In contrast, coumarins and limonoids were found in the rootstock but were not translocated to the leaves. The “Pera” sweet orange/Tropical sunki (GB) graft consists of two varieties that are notably less sensitive to *Phytophthora* gummosis. The scion exhibited a chemical profile similar to GA; however, in this study, the rootstock appears to have influenced the accumulation of certain amino acids and choline. Specifically, arginine (**24**, 52%) showed the most significant increase in concentration in the reinoculated samples, while aspartic acid (**47**, 46%) was predominant in the first inoculation experiment. The flavonoid content in this graft ranged from 15 to 34%. Given that GB is less sensitive to *Phytophthora* gummosis, it is plausible that amino acids contribute more effectively to its defense compared to flavonoids in GA.

The chemical profile of “Tahiti” acid lime leaves differs significantly from that of “Pera” sweet orange (*C. sinensis*), primarily due to the presence of ten coumarins. The only known coumarins in *C. sinensis* leaves are xanthyletin and xanthoxyletin (**7**), previously isolated by our group.^22^ The ten coumarins found in *C. latifolia* (Tahiti lime) exhibit antifungal properties. Citrus black spot (CBS), caused by the fungus *Phyllosticta citricarpa*, leads to fruit blemishes and premature drop, causing severe economic losses in citrus orchards. However, susceptibility to CBS varies among citrus species, while *C. limon* (lemon) is highly susceptible, *C. latifolia* (Tahiti lime) has never shown CBS-related damage, suggesting innate resistance. Our group tested isolated coumarins and a coumarin-rich fraction against *P. citricarpa*. While individual coumarins exhibited no significant inhibition, the coumarin fraction demonstrated strong antifungal activity, indicating a possible synergistic interaction among its components.^60^

This may explain why “Tahiti” lime is highly sensitive to *Phytophthora* gummosis, whereas sweet orange exhibits greater resistance. The latter contains polymethoxylated flavonoids (**31**-**40**), which are absent in “Tahiti” lime. However, the role of flavonoids remains unclear, as their concentrations in “Pera” sweet orange leaves (*C. sinensis*) did not show substantial increases in response to *P. citrophthora*. It remains uncertain whether the low concentrations detected are sufficient for plant defense. The most pronounced concentration variations in response to the oomycete involved coumarins and cinnamic acid derivatives.

“Tahiti” acid lime grafts GC and GD both synthesize coumarins, though their profiles differ. GC synthesizes coumarins **9** and **52**-**57**, whereas GD produces coumarins **63**-**65**. In GC reinoculation experiments, only herniarin (**53**) and psoralen (**55**) surpassed 50% abundance (51% and 53%, respectively). Additionally, cinnamic acid derivatives (**58**-**61**) were exclusively present in GC grafts, with 3-*O*-*p*-coumaroylquinic acid (**59**) and coumaroylglucose (**61**) reaching 70% abundance in reinoculated samples, indicating their strong defensive role against *P. citrophthora*. In the “Tahiti” acid lime/Tropical sunki (GD) graft, the scion is highly sensitive, while the rootstock is less sensitive to *Phytophthora* gummosis. Its molecular profile closely resembles that of the “Tahiti” acid lime/Rangpur lime (GC) graft, as expected, given the shared scion. A similar trend was observed in both grafts, flavonoids in the leaves did not exhibit significant concentration increases in response to *P. citrophthora*, suggesting that the detected low levels might be sufficient for defense. The most notable concentration variation in response to the oomycete occurred in the porphyrin derivative (**66**, 50%) in the inoculated^(b)^ experiment samples.

As discussed in studies focusing on root responses, the leaf data did not support the hypothesis that early pathogen perception in plants triggers an enhanced activation of defense responses against subsequent infections,^23^ also known as systemic acquired resistance (SAR). However, the findings suggest that in GB and GC grafts, the flavonoid biosynthetic pathway was explored during the initial inoculation experiments^(a)^ and later in experiment^(b)^, leading to the activation of pathways for amino acids, coumarins, and cinnamic acid derivatives in response to subsequent *P. citrophthora* challenges. Specifically, in GB, the amino acid pathway for arginine (**24**, 52%) was activated, whereas in GC, the pathways for coumarins (**52**, 43%; **53**, 51%; **55**, 53%; **57**, 43%) and cinnamic acid derivatives (**59**, 70%; **61**, 70%) were predominantly engaged.

### 3.13 Metabolomics Data: A Path Forward for Early Diagnosis of Phytophthora Gummosis in Citrus

This study explores how metabolomics and epigenetic analysis can be used to identify early defense responses in citrus plants infected with *Phytophthora citrophthora*, the causal agent of gummosis. By examining various citrus graft combinations under different inoculation conditions, we aimed to correlate chemical profiles and DNA methylation patterns with disease resistance, offering a new path for early diagnosis and sustainable management.

Heatmap analyses were used to visualize the chemical differences in root and leaf samples from different citrus graft combinations: control, inoculated^(a)^, inoculated^(b)^, and reinoculated. The clustering patterns from these analyses revealed two primary groups (Figure 12). One cluster included leaf samples from GA and GB grafts, characterized mainly by flavonoids, while the second group showed higher levels of coumarins and some flavonoids. These groupings align with previous dendrogram analyses (Figures 7 and 11) and suggest a rootstock-dependent distribution of metabolites. Chemical similarities between leaves and roots in specific graft combinations sharing the same rootstock (e.g., “Rangpur” lime or “Tropical” sunki) support the idea that rootstock genotype significantly influences metabolite profiles.

Epigenetic insights from methylation analysis, as methylation-sensitive amplification polymorphism (MSAP) techniques, using *Eco*RI/*Hpa*II and *Eco*RI/MspI enzymes, revealed further stratification among the grafts based on DNA methylation patterns. The samples separated into three MSAP groups: Group I (high full and hemimethylation), Group II (mainly hemimethylation), and Group III (predominantly unmethylated cytosines).^1^ GC grafts (Group II), composed of “Tahiti” acid lime on “Rangpur” lime rootstock, showed unique chemical compositions rich in flavonoids, coumarins, and cinnamic acid derivatives, particularly compounds **58**–**61**. Notably, 3-*O*-p-coumaroylquinic acid (**59**) and coumaroylglucose (**61**) reached 70% higher concentrations in reinoculated samples. These metabolites are proposed to play a crucial role in defense, and their accumulation may be regulated by epigenetic changes, particularly hemimethylation.

Coumarins were mainly found in root samples, especially in those with the “Tropical” sunki rootstock, which demonstrated stronger defensive properties. Significant increases were observed in pyranocoumarins such as seselin (**15**, 91%), luvangetin (**16**, 82%), and clausarin (**6**, 68%) during the second inoculations. These compounds are believed to play a direct role in inhibiting *P. citrophthora*, which typically infects roots. The Optimal Defense Theory supports this observation by suggesting that plants prioritize chemical defense allocation to tissues most vulnerable to attack.

Citrus species, like members of the Rutaceae family, naturally synthesize pyrano- and furanocoumarins, which have known biosynthetic pathways.^76,77^ Advancements in genome editing and metabolic engineering could allow targeted enhancement of these compounds in resistant varieties.

Leaf samples from inoculated^(a)^ experiments showed increased concentrations of flavonoids in most grafts, especially GA and GC. GA grafts showed a 31–46% increase in flavonoids **31**–**43**, while GC had elevated levels of flavonoids **44**, **46**, **49**, **50**, **52**, and **55**–**58** (**33**–**38**%). GB showed modest increases, and GD had no significant flavonoid boost, though it showed elevated coumarin and porphyrin levels. These findings suggest that a specific set of flavonoids may act as early biomarkers for *Phytophthora* infection, appearing before visible symptoms. Such early detection could reduce reliance on PCR-based diagnostics by enabling faster, cheaper, and more scalable detection through mass spectrometry, using minimal plant material.

The study’s integration of metabolomic profiling and epigenetic analysis provides a novel approach for early detection and resistance breeding in citrus. By identifying key metabolites linked to defense, such as flavonoids, coumarins, alkaloids, and pyranocoumarins, we can develop biomarkers for early infection detection. These findings could also inform the design of biobased treatments, reducing dependence on chemical pesticides. Furthermore, the consistent correlation between rootstock genotype and defense metabolite production highlights the importance of rootstock selection in managing disease susceptibility.

The potential to harness epigenetic memory through controlled infection or breeding strategies opens new avenues for durable resistance. Coupled with the cost-effectiveness and scalability of metabolomics, this study outlines a sustainable, scientifically grounded path forward in combating *Phytophthora* gummosis in citrus crops.

## Supplementary Material



## Notes

Figures S1–S6 (molecular networks of roots and leaves under different scion/rootstock combinations and inoculation conditions), Schemes S1–S66 (MS fragmentation patterns of compounds **1–66**), Table S1 (metabolites detected by molecular networking and MS/MS spectra), Table S2 (features with CV% > 20% in QC samples excluded from analysis), and Table S3 (univariate analysis of metabolites **1**-**66**) (PDF)
