# Supplementary material for: Metabolomic Profiling of Citrus Grafts Challenged by Phytophthora citrophthora: Using the Same Samples Previously Analyzed for Epigenetic Responses
Source: J Agric Food Chem. 2025 Oct 28;73(45):29171–91. doi: 10.1021/acs.jafc.5c07224 (PMC12616686; doi:10.1021/acs.jafc.5c07224)
Supplement: Supplementary file 1 [file jf5c07224_si_001.pdf]

SUPPORTING INFORMATION

Metabolomic Profiling of Citrus Grafts Challenged by *Phytophthora citrophthora*: Using the Same Samples Previously Analyzed for Epigenetic Responses

Felipe Hilario,† Luciano da Silva Pinto,† Adielle Rodrigues da Silva,§ João Batista Fernandes,† Abelmon da Silva Gesteira,*§ and Maria Fátima das Graças Fernandes da Silva*†

†Departamento de Química, Universidade Federal de São Carlos, CP 676, São Carlos – SP - 13565-905, Brazil; §Embrapa Mandioca e Fruticultura, Cruz das Almas, Bahia, 44380-000, Brazil

Figures, Table and Schemes

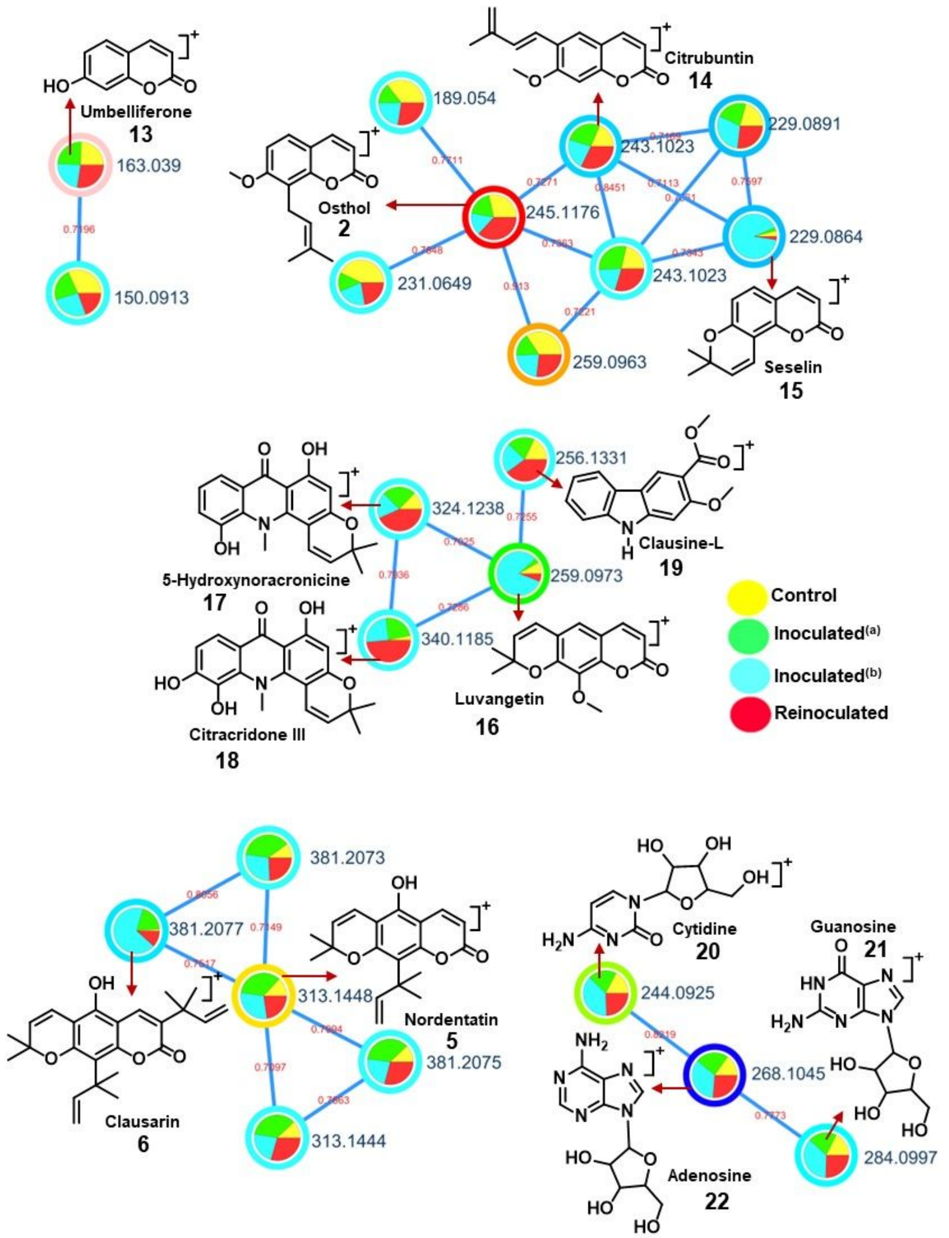

Figure S1. The molecular network of the analysis carried out in positive ion monitoring for roots from scion 'Pera' sweet orange/'Tropical' sunki rootstock (GB), control (yellow), inoculated(a) (green), inoculated(b) (blue), and reinoculated (red), and selected clusters with nodes showing the compounds annotated by GNPS library search.

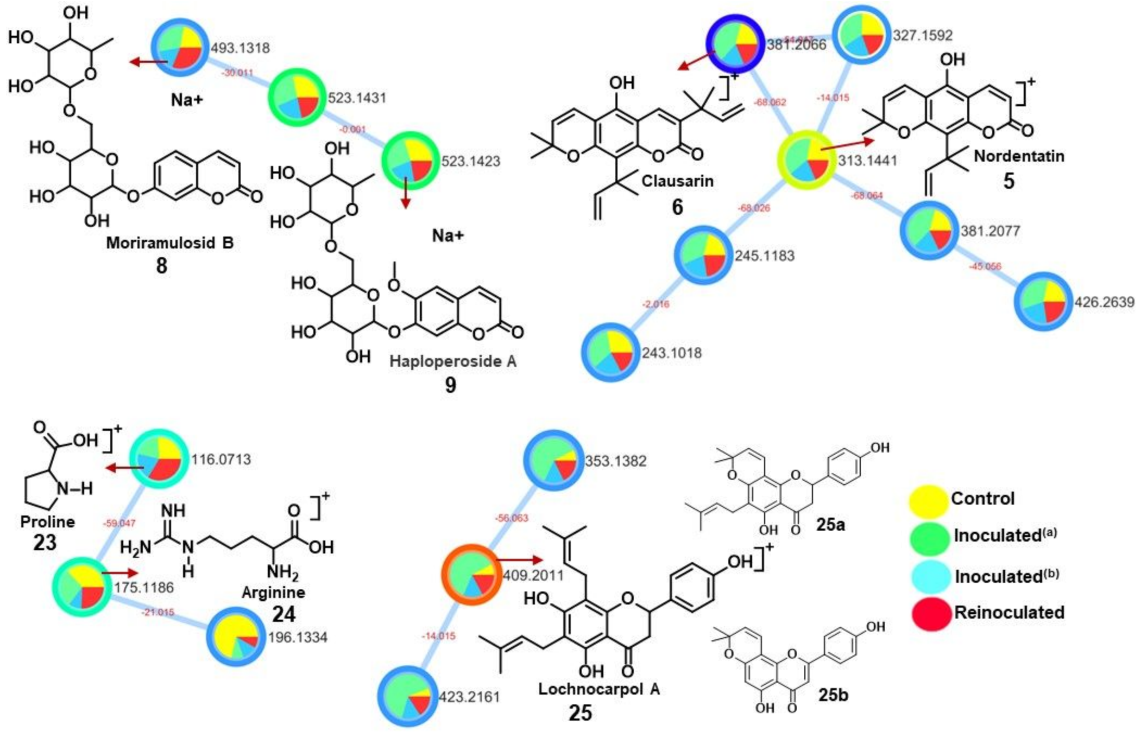

Figure S2. The molecular network of the analysis carried out in positive ion monitoring for roots from scion 'Tahiti' acid lime/'Rangpur' lime rootstock (GC), control (yellow), inoculated[(a)] (green), inoculated[(b)] (blue), and reinoculated (red), and selected clusters with nodes showing the compounds annotated by GNPS library search.

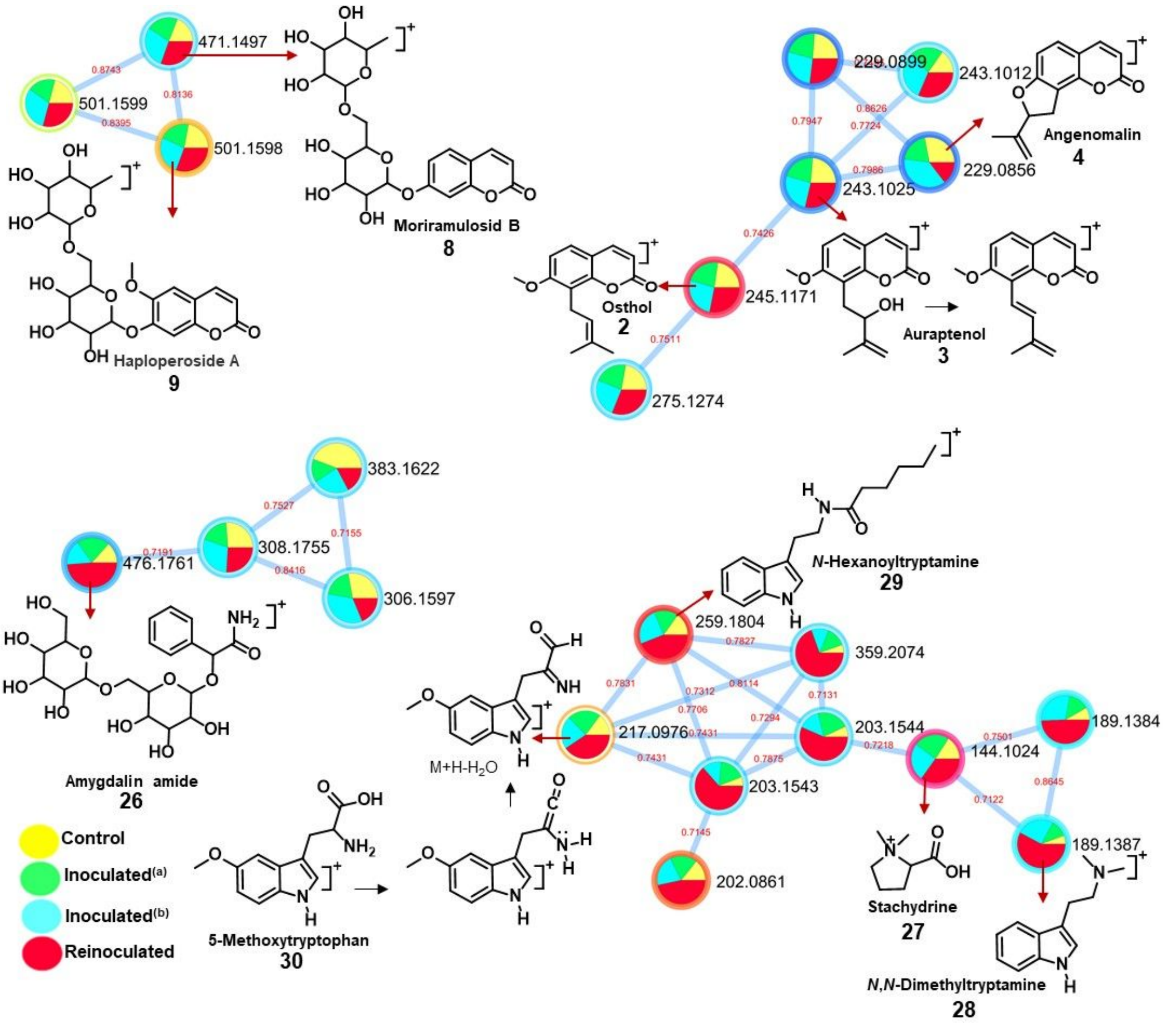

Figure S3. The molecular network of the analysis carried out in positive ion monitoring for roots from 'Tahiti' acid lime/'Tropical' sunki rootstock (GD), control (yellow), inoculated[(a)] (green), inoculated[(b)] (blue), and reinoculated (red), and selected clusters with nodes showing the compounds annotated by GNPS library search.

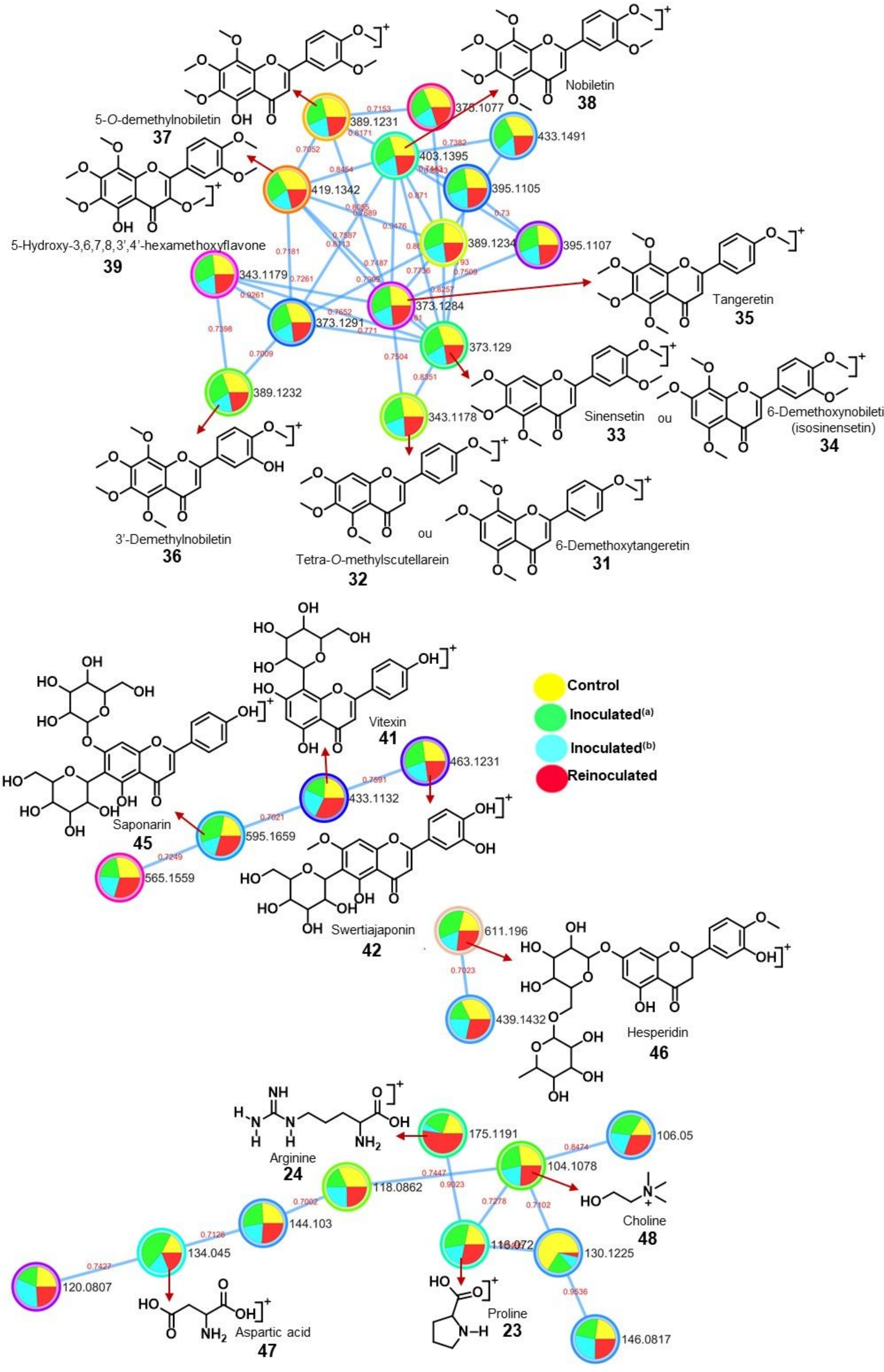

Figure S4. The molecular network of the analysis carried out in positive ion monitoring for leaves from scion 'Pera' sweet orange/'Tropical' sunki rootstock (GB), control (yellow), inoculated[(a)] (green), inoculated[(b)] (blue), and reinoculated (red), and selected clusters with nodes showing the compounds annotated by GNPS library search.

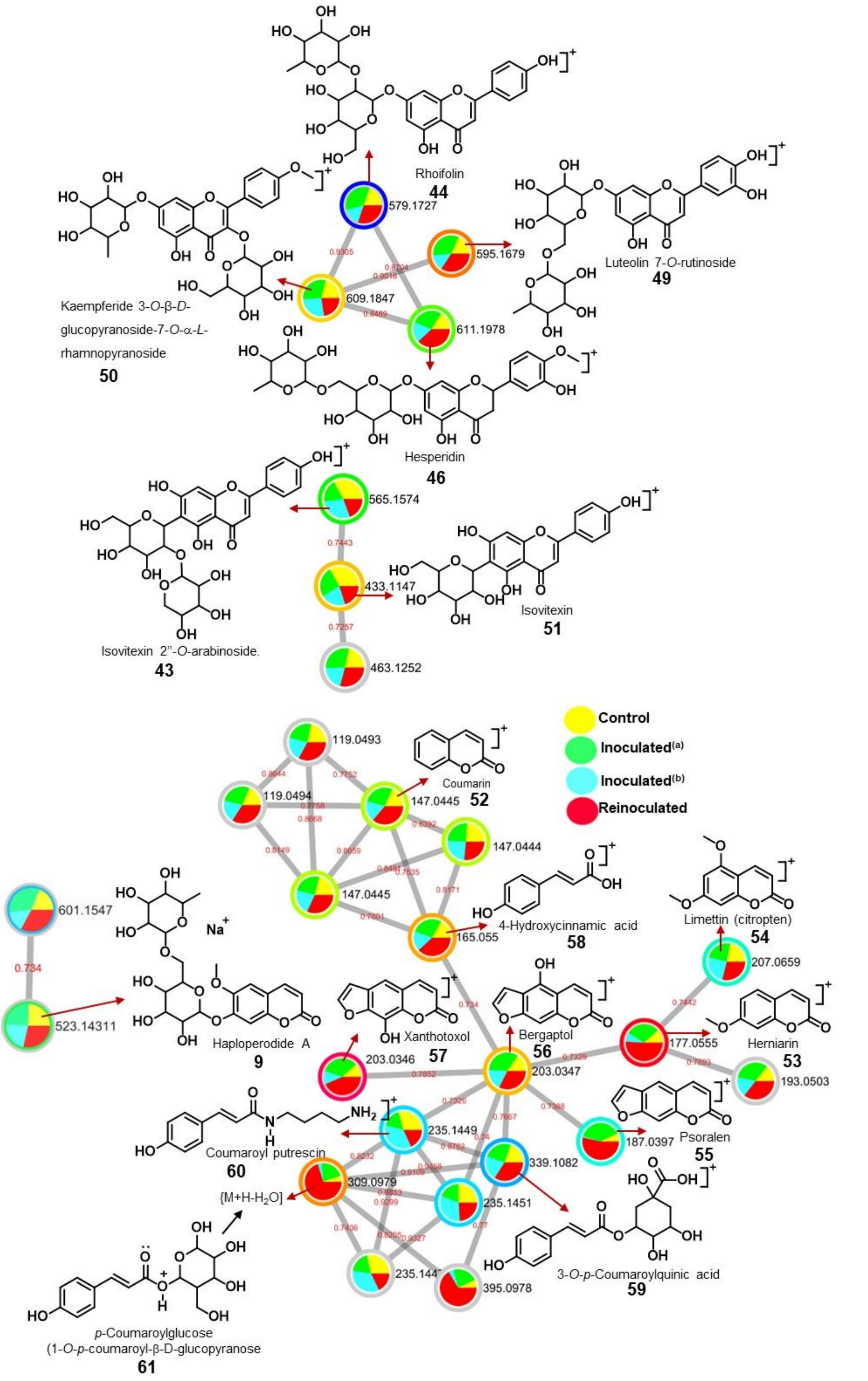

Figure S5. The molecular network of the analysis carried out in positive ion monitoring for leaves from scion 'Tahiti' acid lime/'Rangpur' lime rootstock (GC), control (yellow), inoculated[(a)] (green), inoculated[(b)] (blue), and reinoculated (red), and selected clusters with nodes showing the compounds annotated by GNPS library search.

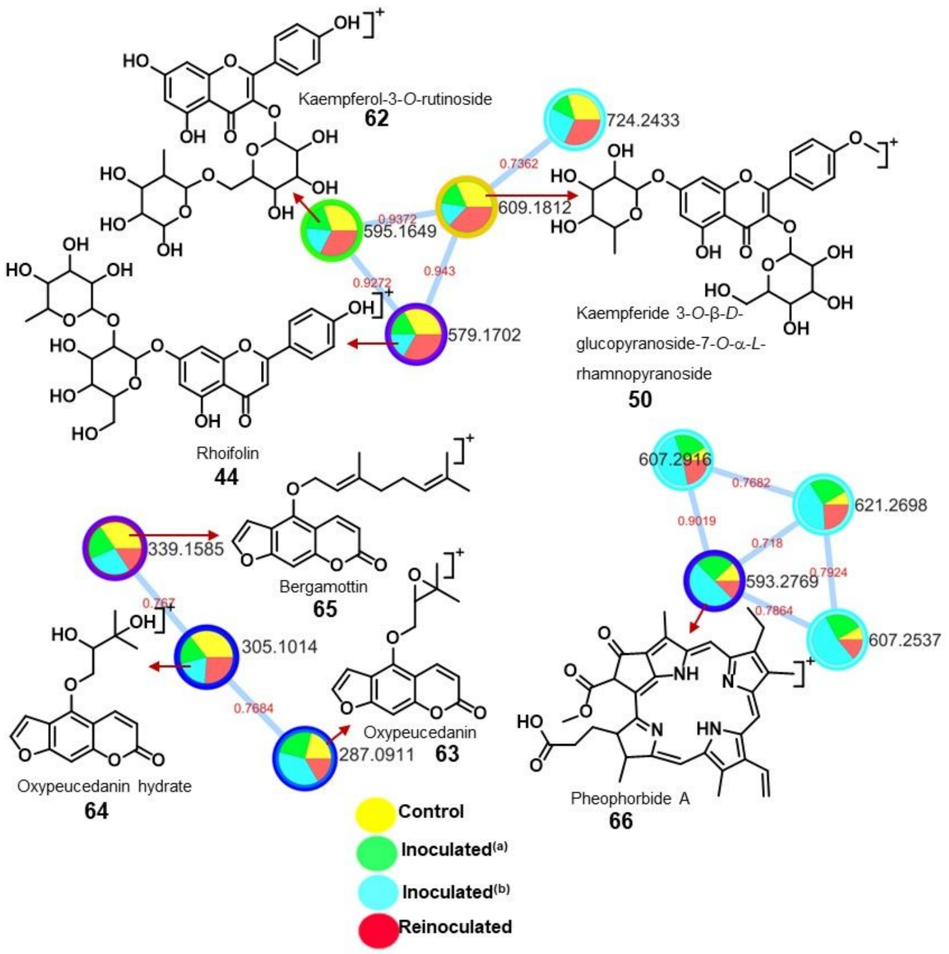

Figure S6. The molecular network of the analysis carried out in positive ion monitoring for leaves from ‘Tahiti’ acid lime/'Tropical' sunki rootstock (GD), control (yellow), inoculated[(a)] (green), inoculated[(b)] (blue), and reinoculated (red), and selected clusters with nodes showing the compounds annotated by GNPS library search.

Table S1. Metabolites detected using the molecular networking tool and MS/MS spectra.

**Compounds detected**	**RT min**	**Molecular formula**	**$[M-H]^+$ Measured**	**Mass accuracy (ppm)**	**MS^2 main fragments**
Coumarins					
osthenol (**1**)	8.9	$C_{14}H_{14}O_3$	231.1017	0.4	175; 147; 119; 91
osthol (**2**)	11.2	$C_{15}H_{16}O_3$	245.1174	0.8	215; 187; 69
auraptenol (**3**)	10.8	$C_{15}H_{16}O_4$	243.1018 $[M+H-H_2O]^+$	3.7	213; 173; 145; 131
angenomalin (**4**)	8.8	$C_{14}H_{12}O_3$	229.0863	3.5	229; 187; 186; 175; 159; 147; 131; 91
nordentatin (**5**)	11.6	$C_{19}H_{20}O_4$	313.1439	1.5	271; 257; 229; 215; 203; 187
clausarin (**6**)	15.7	$C_{24}H_{28}O_4$	381.2067	1.8	339; 325; 311; 297; 283; 269; 255; 241; 69
xanthoxyletin (**7**)	10.6	$C_{15}H_{14}O_4$	259.0969	1.5	259; 243; 229
moriramulosid B (**8**)	3.9	$C_{21}H_{26}O_{12}$	493.1324 $[M+Na]^+$	1.6	347; 331; 185
haploperoside A (**9**)	4	$C_{22}H_{28}O_{13}$	523.1429 $[M+Na]^+$	1.3	337; 331; 215
moriramulosid B 6-glyceroyl (**10**)	5.8	$C_{24}H_{30}O_{15}$	561.1948	1.3	470; 379; 361; 242
Limonoids					
limonin (**11**)	9	$C_{26}H_{30}O_9$	471.2016	0.4	213; 161; 95
nomilin (**12**)	9.5	$C_{28}H_{34}O_9$	515.2278	0.4	205; 161; 95

Table S1. Continuation.

Compounds detected	RT min	Molecular formula	[M-H]$^+$ Measured	Mass accuracy (ppm)	MS^2 main fragments
Coumarins					
umbelliferone (**13**)	5.4	$C_9H_7O_3$	163.0395	0.6	107; 91; 79
citrubuntin (**14**)	10.8	$C_{15}H_{14}O_3$	243.1023	0.8	213; 185; 175; 173; 159; 131
seselin (**15**)	10.3	$C_{14}H_{12}O_3$	229.0866	0.4	213; 175; 159; 147; 131; 91
luvangetin (**16**)	6.1	$C_{15}H_{14}O_4$	259.0971	0.4	189; 177; 161; 131; 103
Acridone alkaloids					
5-hydroxynoracronycine (**17**)	11.4	$C_{19}H_{17}NO_4$	324.1231	1.5	309; 294; 268; 267
citracridone III (**18**)	10.3	$C_{19}H_{17}NO_5$	340.1186	0.3	325; 310; 284; 283
Carbazole alkaloid					
clausine-L (**19**)	4.4	$C_{15}H_{13}NO_3$	256.1340	1.5	226; 224; 223; 144
Nucleosides					
cytidine (**20**)	0.8	$C_9H_{13}N_3O_5$	244.0932	0.4	112
guanosine (**21**)	0.9	$C_{10}H_{13}N_5O_5$	284.0999	1.4	152
adenosine (**22**)	1.0	$C_{10}H_{13}N_5O_4$	268.1050	1.5	136
Amino acids					
L-proline (**23**)	0.8	$C_5H_9NO_2$	116.0707	4.3	70
L-arginine (**24**)	0.7	$C_6H_{14}N_4O_2$	175.1189	3.4	139; 138; 92; 70
Prenylated flavonoid					
lochnocarpol A (**25**)	12.7	$C_{25}H_{28}O_5$	409.2012	0.7	297; 233; 177
Amide					
amygdalin amide (**26**)	4.6	$C_{20}H_{29}NO_{12}$	476.1758	2.1	152; 134
Ornithine alkaloid					
stachydrine (**27**)	0.8	$C_7H_{14}NO_2$	144.1025	0.4	102; 84
Simple indole derivatives					
N,*N*-dimethyltryptamine (**28**)	4.0	$C_{12}H_{16}N_2$	189.1387	2.1	144; 130
N-hexanoyl tryptamine (**29**)	9.3	$C_{16}H_{22}N_2O$	259.1815	1.5	144; 130
Tryptophan derivative					
5-methoxytryptophan (**30**)	4.0	$C_{12}H_{14}N_2O_2$	217.0984	0.4	217; 144
Polymethoxylated flavones					
6-demethoxytangeretin (**31**)	9.4	$C_{19}H_{18}O_6$	343.1172	1.2	313; 282; 211; 133
tetra-*O*-methylscutellarein (**32**)	9.4	$C_{19}H_{18}O_7$	343.1179	0.9	313; 133; 181
sinensetin (**33**)	8.8	$C_{20}H_{20}O_7$	373.1276	1.6	357; 343; 211; 163
isosinensetin (**34**)	8.1	$C_{20}H_{20}O_7$	373.1278	1.3	357; 343; 315; 181; 163
tangeritin (**35**)	10.0	$C_{20}H_{20}O_7$	373.1283	0.3	343; 297; 211, 133
3'-demethylnobiletin (**36**)	8.3	$C_{20}H_{20}O_8$	389.1224	1.8	359; 211; 149
5-*O*-demethylnobiletin (**37**)	8.3	$C_{20}H_{20}O_8$	389.1224	1.8	359; 211
nobiletin (**38**)	9.3	$C_{20}H_{20}O_{10}$	403.1387	1.5	388; 373; 163
5-hydroxy-3,6,7,8,3’,4’-hexamethoxyflavone (**39**)	10.0	$C_{21}H_{22}O_8$	419.1336	0.2	389; 374; 211
eupatorin (**40**)	8.9	$C_{18}H_{16}O_7$	345.0972	0.9	330; 329; 284; 149
Flavone C-glycosides					
vitexin (**41**)	5.1	$C_{18}H_{16}O_7$	433.1120	2.1	315; 313; 283
swertiajaponin (**42**)	5.3	$C_{21}H_{20}O_{10}$	463.1221	3.0	343; 329; 327; 313; 301
isovitexin 2''-*O*-arabinoside (**43**)	4.9	$C_{22}H_{22}O_{11}$	565.1540	2.1	447; 433; 415; 397; 313
Flavone O-glycosides					
rhoifolin (**44**)	5.4	$C_{26}H_{28}O_{14}$	579.1698	2.4	345; 271
saponarin (**45**)	4.8	$C_{27}H_{30}O_{15}$	595.1649	1.3	433; 337; 313; 283; 201
hesperidin (**46**)	5.7	$C_{28}H_{34}O_{15}$	611.1962	1.3	315; 303; 219; 153; 151
Amino acid					
L-aspartic acid (**47**)	0.8	$C_4H_7NO_4$	134.0453	3.7	74; 70

Table S1. Continuation.

Cholines					
choline (**48**)	0.7	$C_5H_{14}NO$	104.1069	1.0	60; 58
Flavone O-glycosides					
luteolin-7-*O*-rutinoside (**49**)	5.1	$C_{27}H_{30}O_{15}$	595.1669	1.0	287; 177; 107
kaempferide 3-O-*β*-D-glucopyranoside 7-O-*α*-L-rhamnopyranoside (**50**)	5.5	$C_{28}H_{32}O_{15}$	609.1832	2.1	301; 292; 286; 222
Flavone C-glycosides					
isovitexin (**51**)	5.1	$C_{21}H_{20}O_{10}$	433.1148	3.0	313; 283
Coumarins					
coumarin (**52**)	3.7	$C_9H_6O_2$	147.0443	2.0	119; 91; 65
herniarin (**53**)	7.4	$C_{10}H_8O_3$	177.0551	0.6	134; 121; 91
limettin (**54**)	8.5	$C_{11}H_{10}O_4$	207.0660	1.4	192; 164; 149; 121; 91
psoralen (**55**)	7.8	$C_{11}H_6O_3$	187.0395	0.1	159; 143;131; 115
bergaptol (**56**)	14.2	$C_{11}H_6O_4$	203.0348	1.9	175; 159; 147; 131; 119; 103; 91
xanthotoxol (**57**)	13.4	$C_{11}H_6O_4$	203.0347	1.5	159; 147; 131; 129; 119; 91
Cinnamic acid derivatives					
p-hydroxycinnamic acid (**58**)	6.2	$C_9H_8O_3$	165.0548	2.4	147; 119; 91
3-*O*-*p*-coumaroylquinic acid (**59**)	3.9	$C_{16}H_{18}O_8$	339.1083	0.9	321; 189;147; 119; 91
coumaroylputrescine (**60**)	0.9	$C_{13}H_{18}N_2O_2$	235.1446	0.4	147; 119; 91; 72
p-Coumaroylglucose (**61**)	5.6	$C_{15}H_{18}O_8$	309.0979 $[M+H-H_2O]^+$	1.6	289; 147; 119; 91
Flavone O-glycosides					
Kaempferol-3-*O*-rutinoside (**62**)	5.1	$C_{27}H_{30}O_{15}$	595.1652	1.8	412; 381; 287
Coumarins					
oxypeucedanin (**63**)	9.4	$C_{16}H_{14}O_5$	309.0724 $[M+Na^+]$ 287.0905	4.8<br><br>4.9	209;<br><br>203; 159; 147; 131; 119; 84
oxypeucedanin hydrate (**64**)	6.9	$C_{16}H_{16}O_6$	327.0833 $[M+Na^+]$ 305.1016	3.7<br><br>2.9	229; 209;<br><br>203; 159; 147; 118; 83
bergamottin (**65**)	14.2	$C_{21}H_{22}O_4$	339.1592	1.2	203; 175; 159; 147; 131; 119; 91; 81
Porphyrins					
pheophorbide A (**66**)	15.8	$C_{35}H_{36}N_4O_5$	593.2761	0.5	575; 564; 533

Mass spectra of metabolites annotated and mentioned in Table S1, and Schemes 1-66, MS fragmentation patterns for detected compounds (1-66).

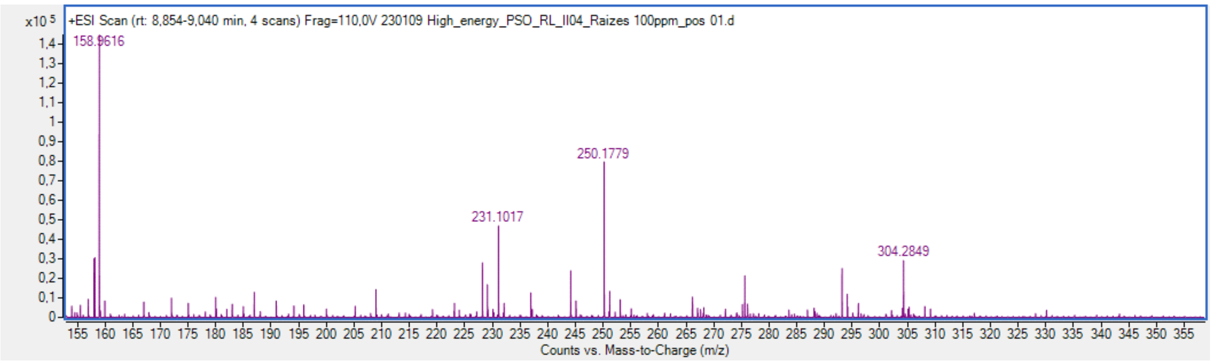

MS^1 spectrum from Osthenol (**1**) $M+H]^+$ *m/z* 231.1017, error: 0.4 ppm.

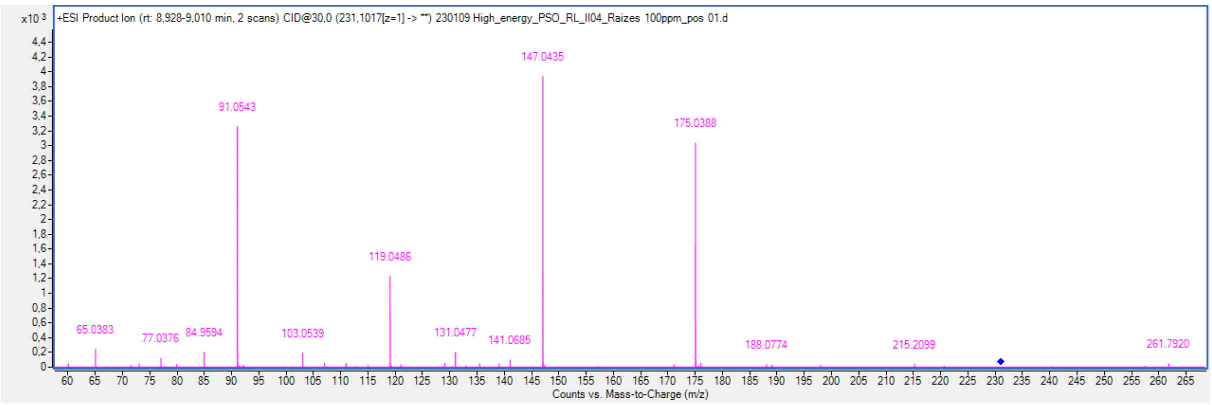

MS/MS spectrum from Osthenol (**1**).

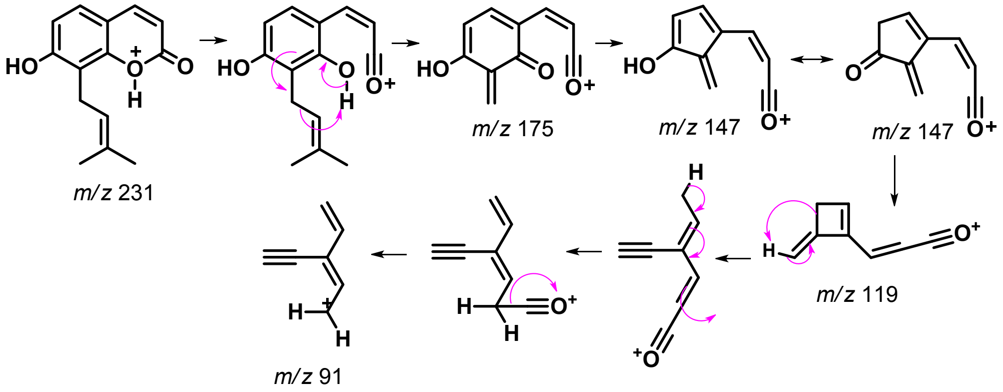

Scheme S1. MS fragmentation patterns for coumarin Osthenol (**1**).

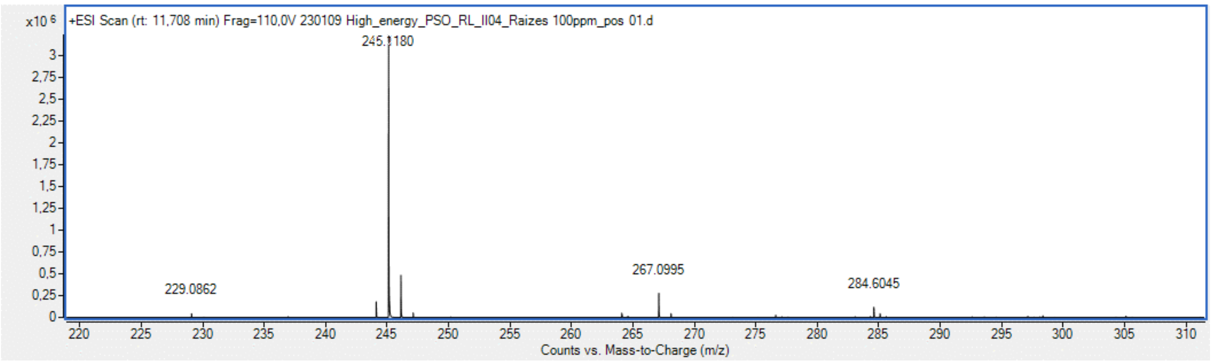

MS[1] spectrum from Osthole (**2**) M+H]$^+$ *m/z* 245.1174, error: 0.8 ppm.

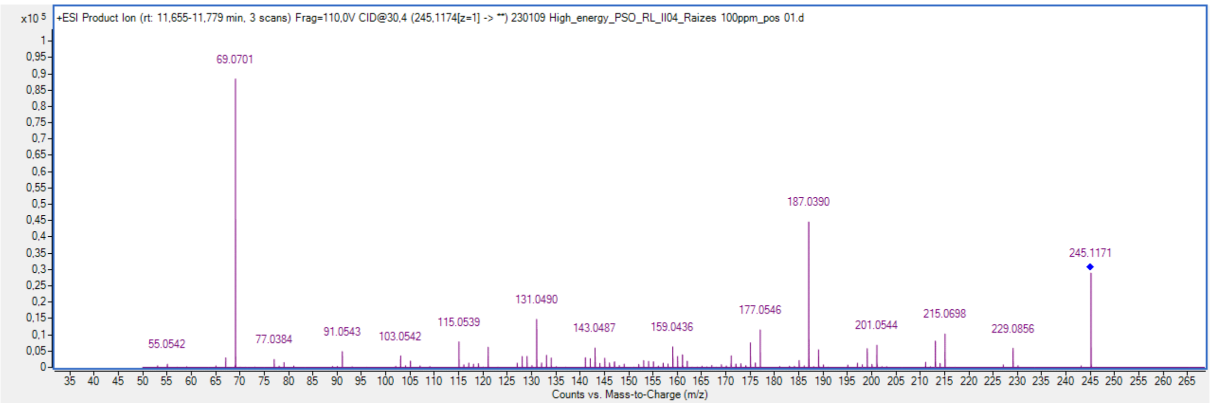

MS/MS spectrum from Osthole (**2**).

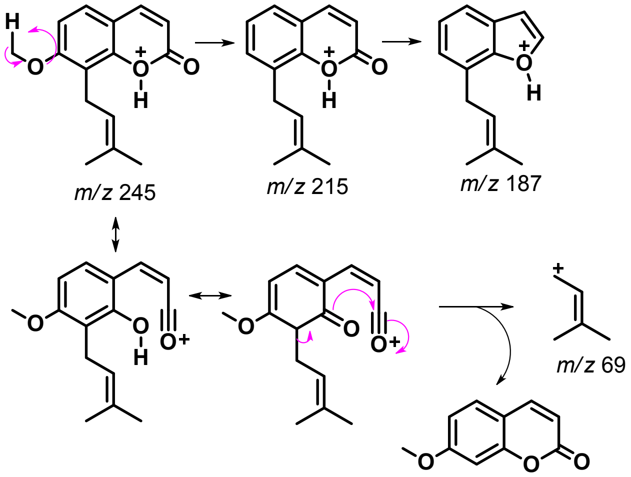

Scheme 2. MS fragmentation patterns for coumarin Osthole (**2**).

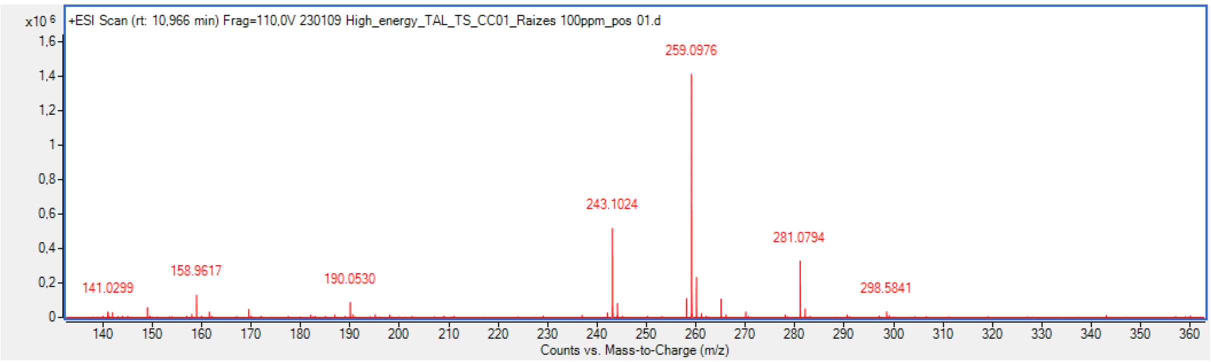

MS[1] spectrum from Auraptenol (**3**), $[M+H-H_2O]^+$ *m/z* 243.1024, error: 3.5 ppm.

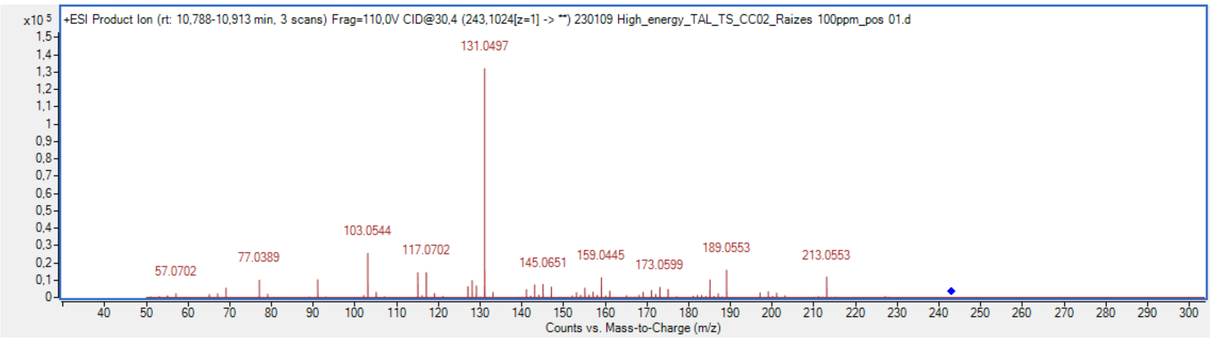

MS/MS spectrum from Auraptenol (**3**).

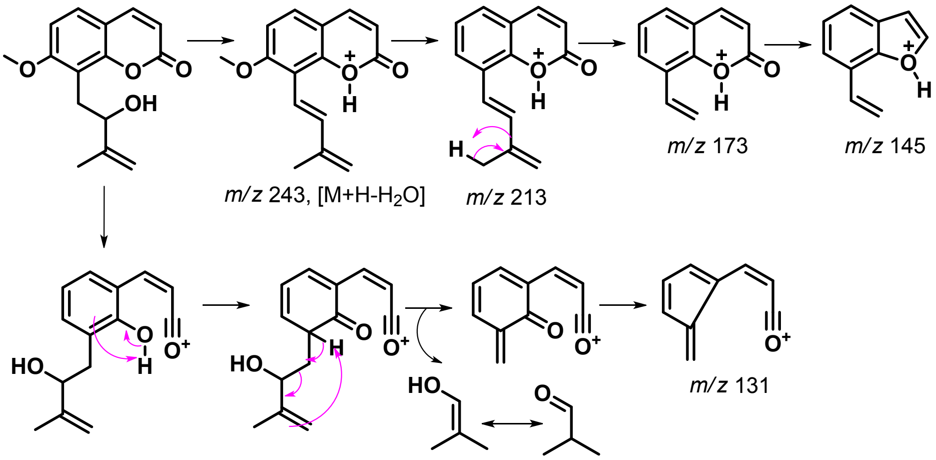

Scheme 3. MS fragmentation patterns for coumarin Auraptenol (**3**).

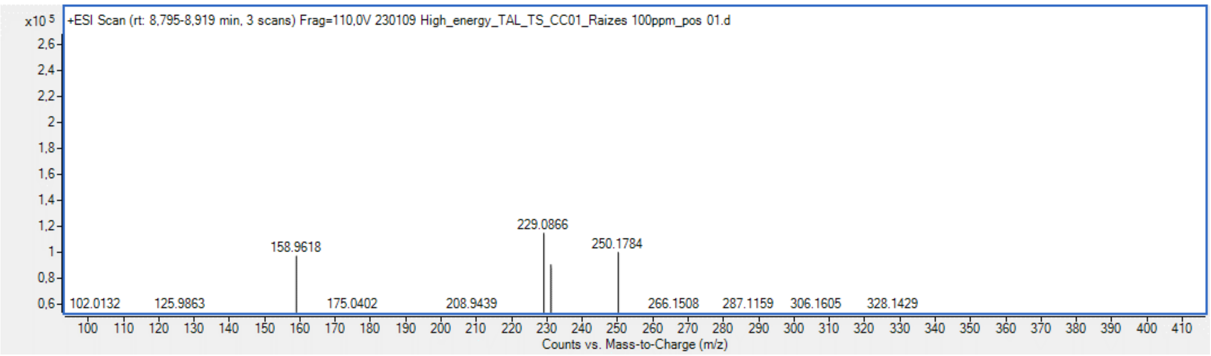

MS[1] spectrum from Angenomalin (**4**), $[M+H]^+$ *m/z* 229.0866, error: 3.5 ppm.

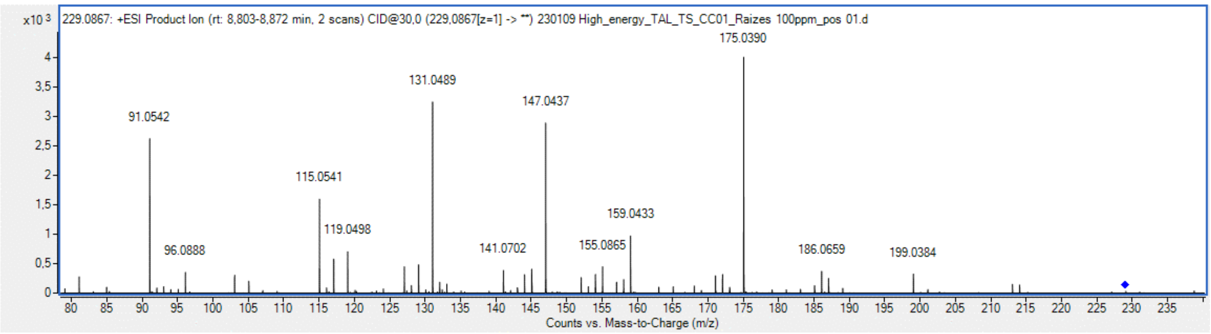

MS/MS spectrum from Angenomalin (**4**).

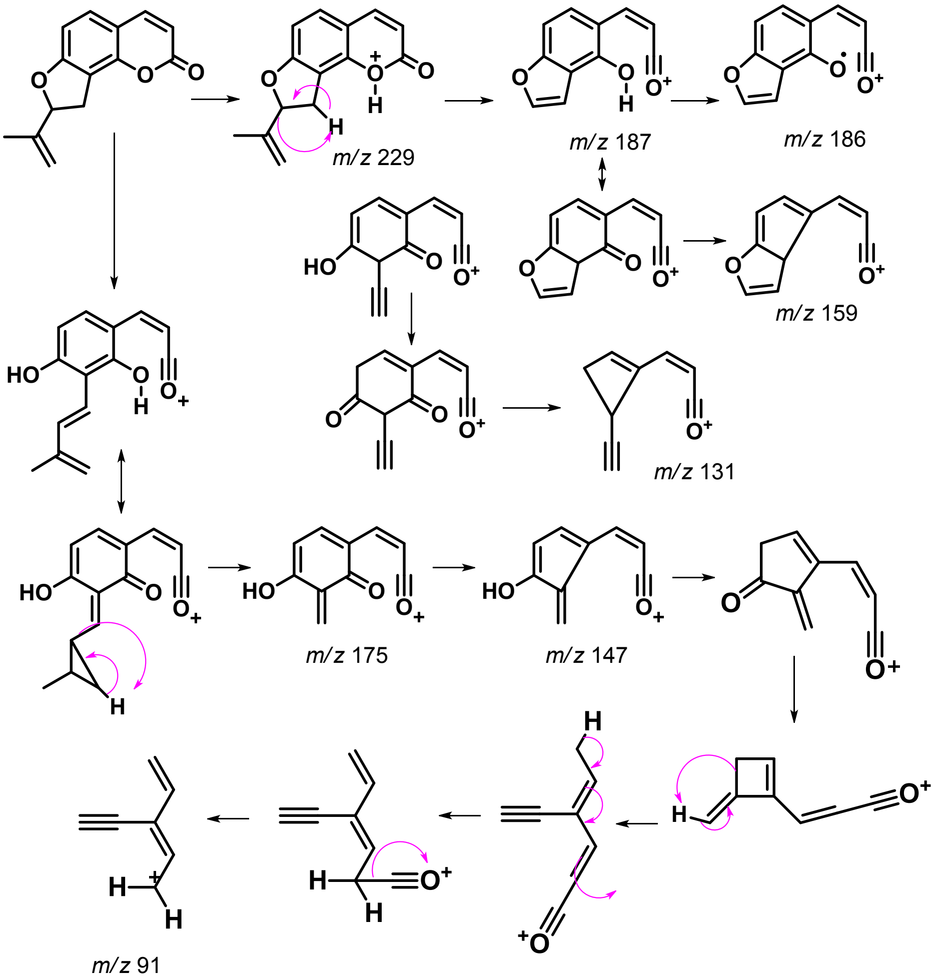

Scheme 4. MS fragmentation patterns for coumarin Angenomalin (**4**).

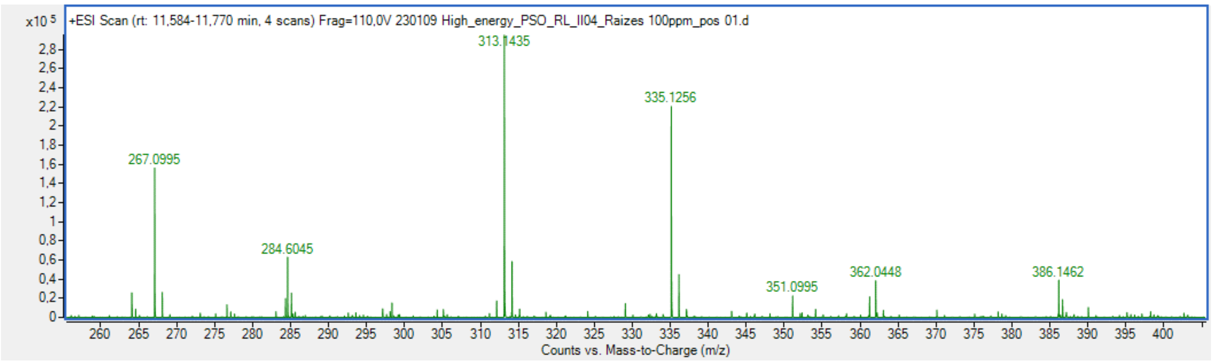

MS[1] spectrum from Nordentatin (**5**) $[M+H]^+$ *m/z* 313.1439, error: 1,5 ppm.

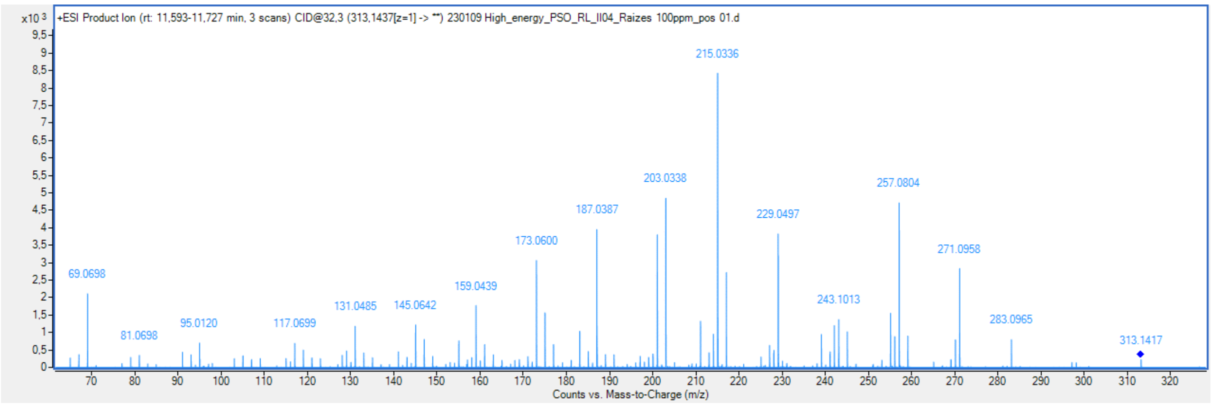

MS/MS spectrum from Nordentatin (**5**).

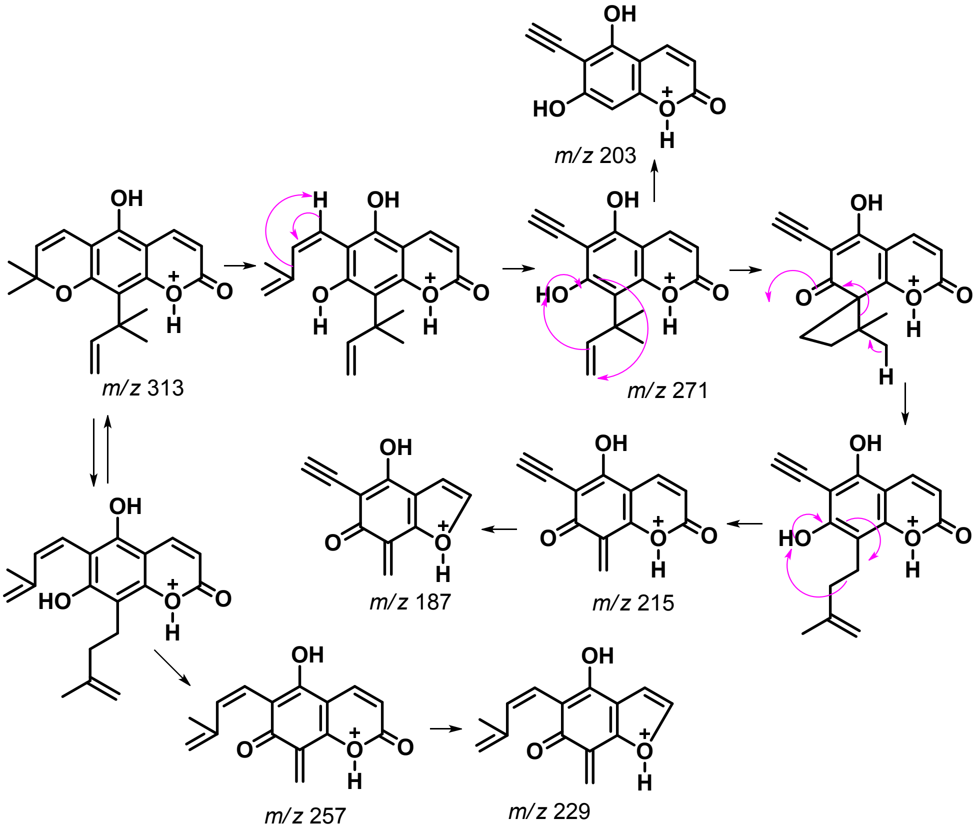

Scheme 5. MS fragmentation patterns for coumarin Nordentatin (**5**).

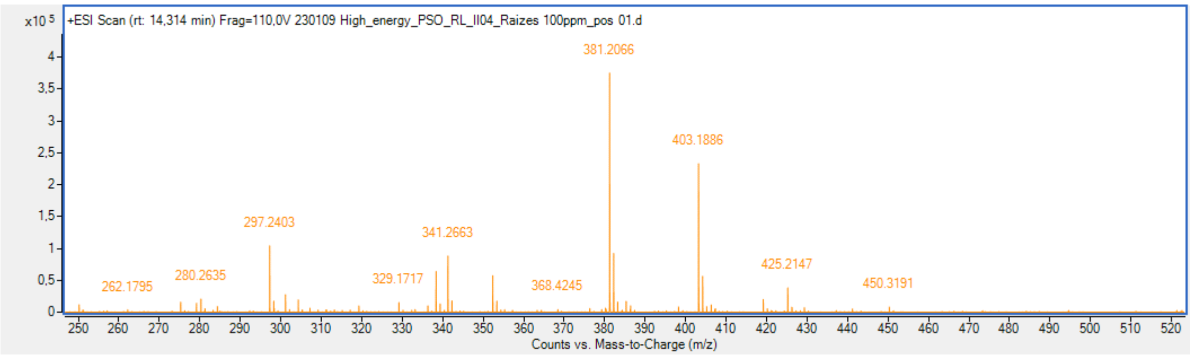

MS[1] spectrum from Clausarin (**6**) $[M+H]^+$ *m/z* 381.2067, erro: 1.8 ppm.

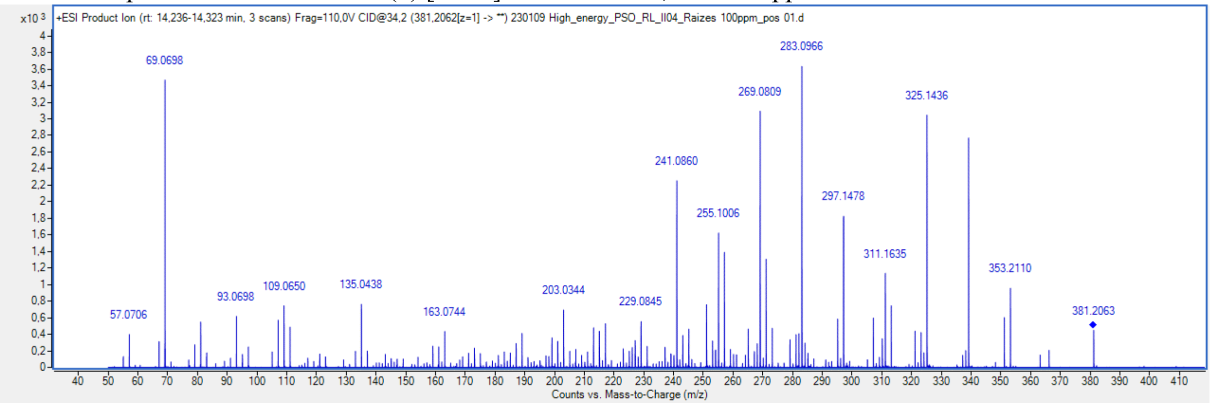

MS/MS spectrum from Clausarin (**6**).

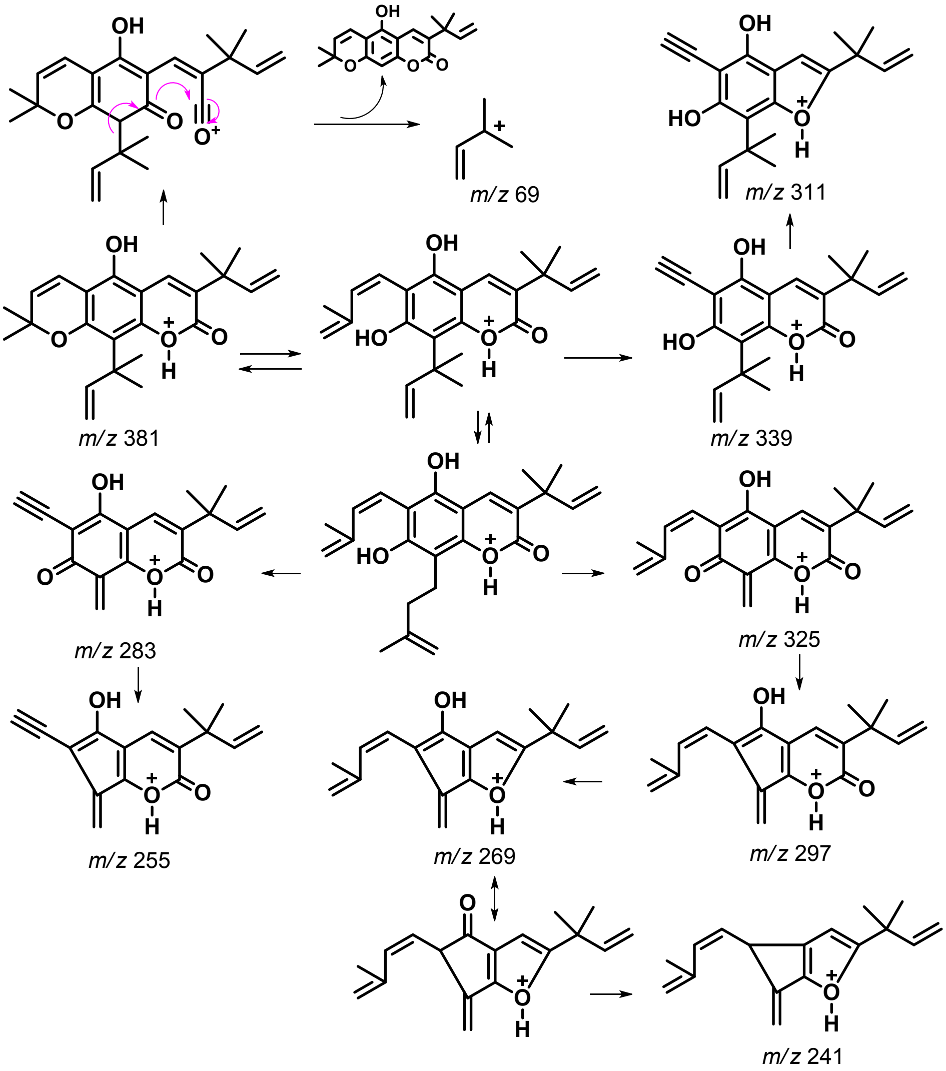

Scheme 6. MS fragmentation patterns for coumarin Clausarin (**6**).

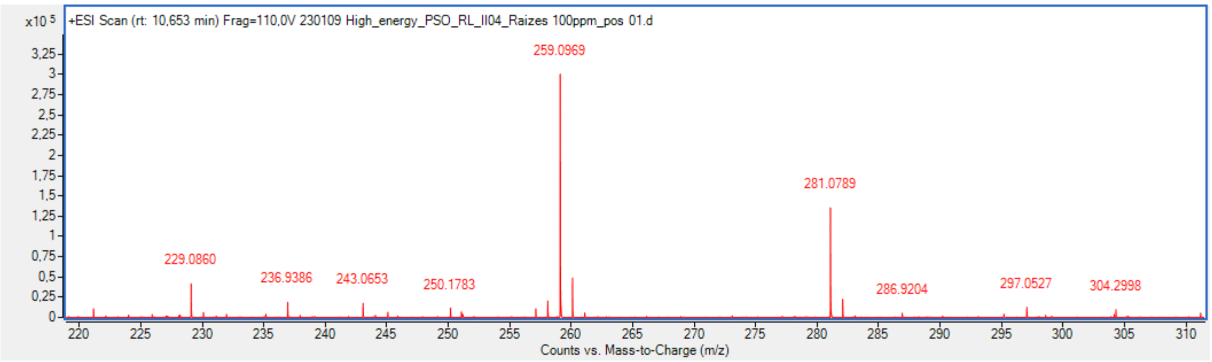

MS[1] spectrum from Xanthoxyletin (**7**) M+H]$^+$ *m/z* 259.0969, error: 1.5 ppm.

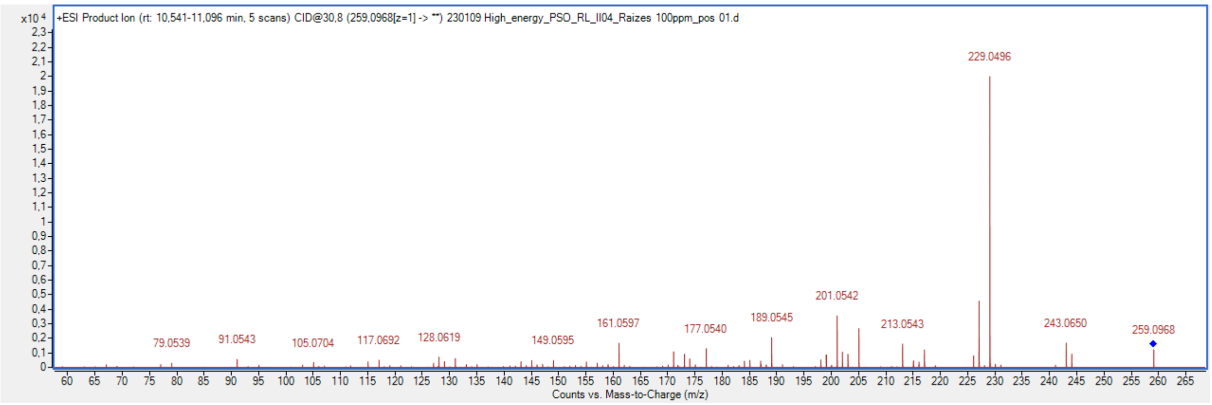

MS/MS spectrum Xanthoxyletin (**7**).

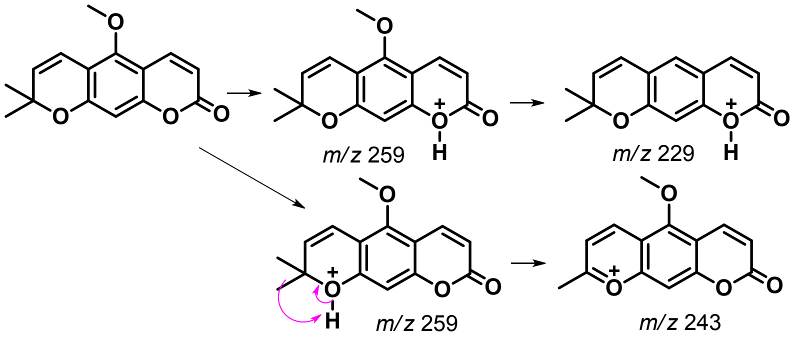

Scheme 7. MS fragmentation patterns for coumarin Xanthoxyletin (**7**).

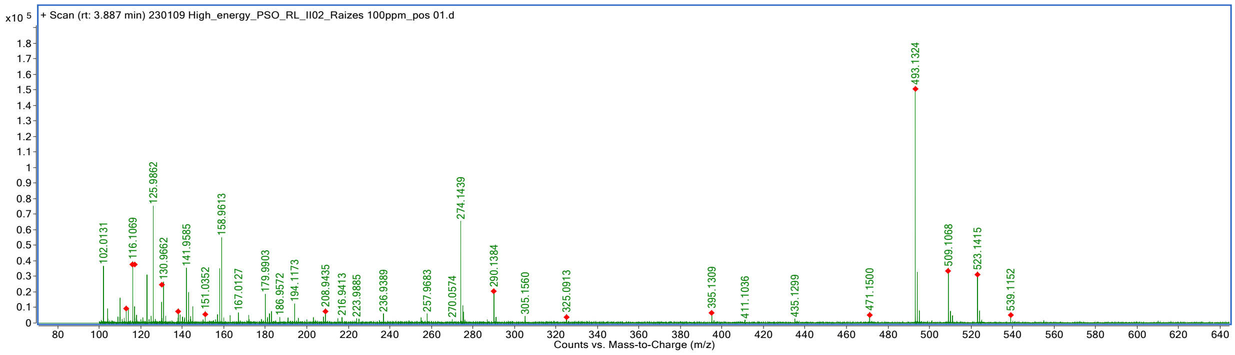

MS[1] spectrum from Moriramulosid B (**8**) M+Na]$^+$ *m/z* 493.1324, error: 1.6 ppm.

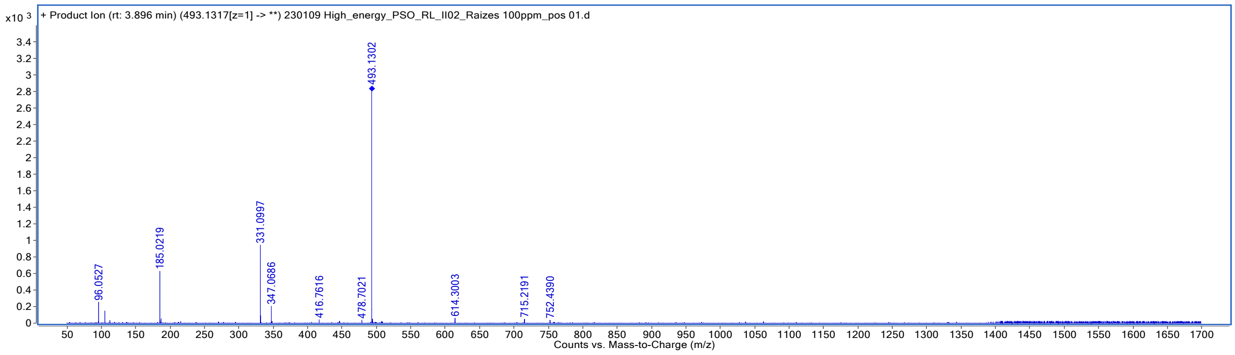

MS/MS spectrum from Moriramulosid B (**8**).

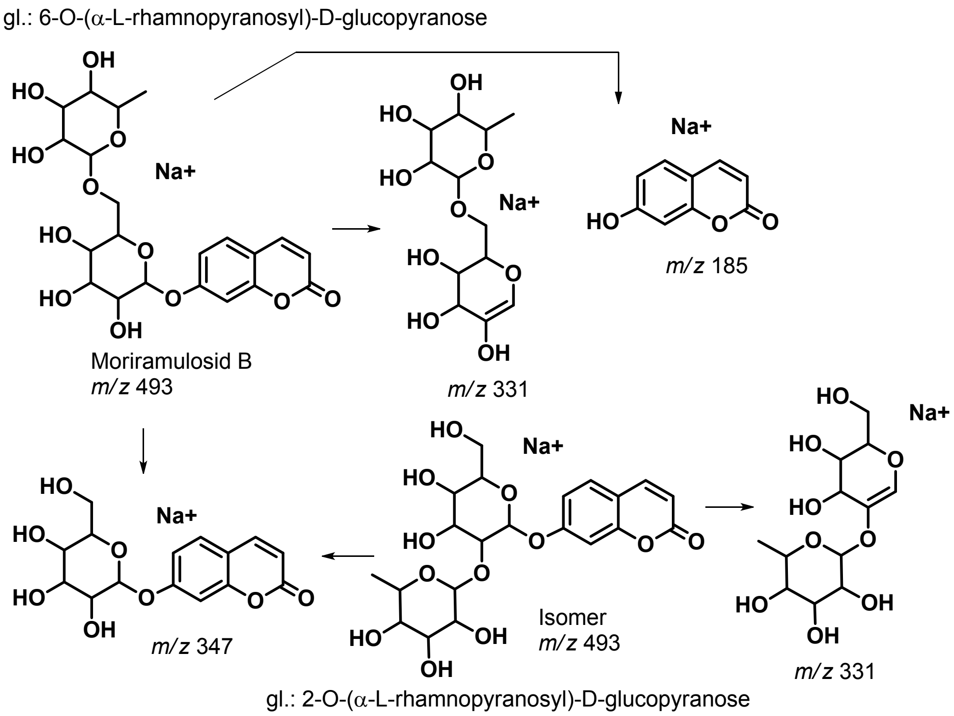

Scheme 8. MS fragmentation patterns for coumarin Moriramulosid B (**8**).

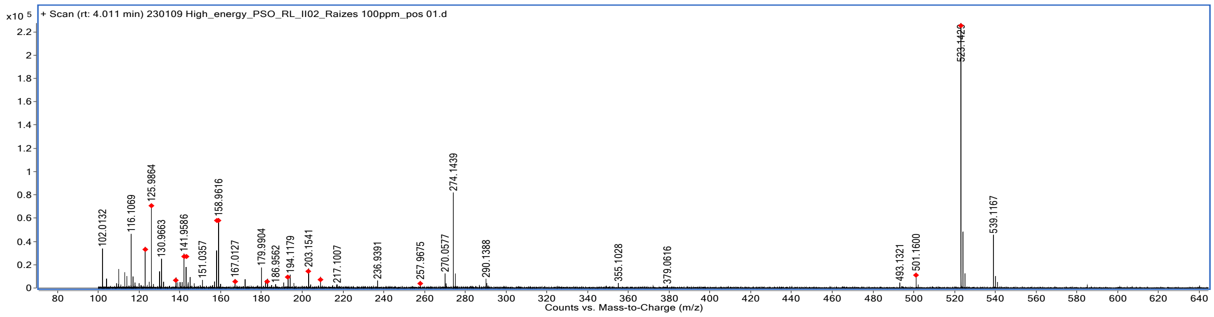

MS[1] spectrum from Haploperosides A (**9**) $[M+Na]^+$ *m/z* 523.1429, error: 1.3 ppm.

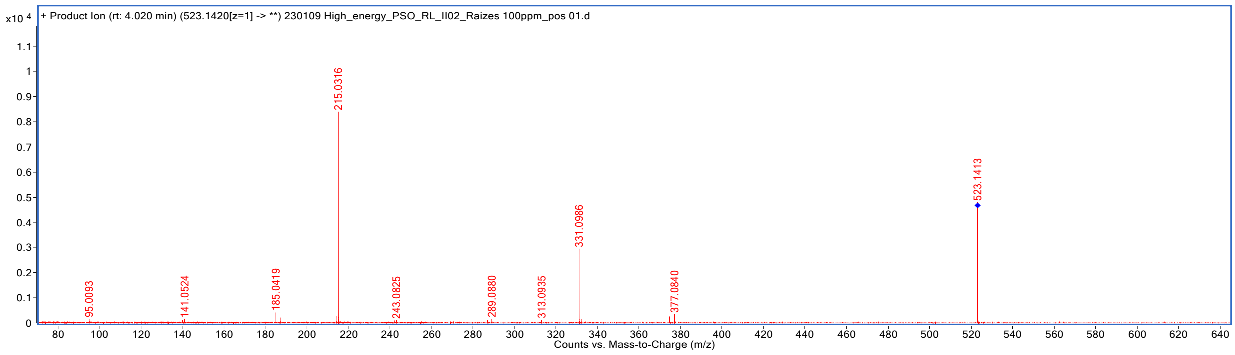

MS/MS spectrum from Haploperosides A (**9**).

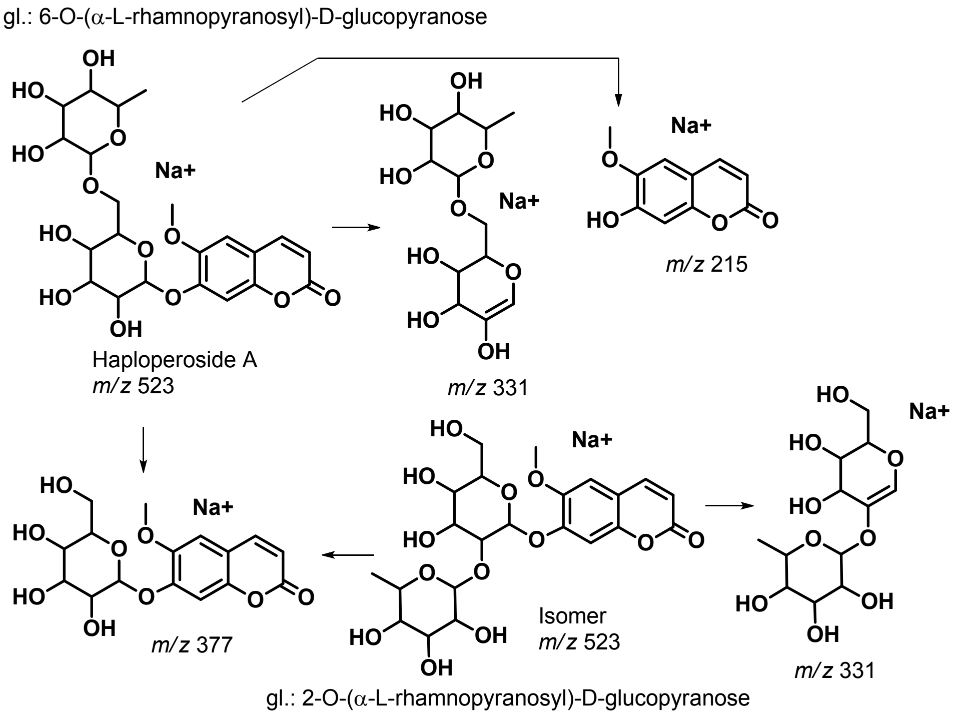

Scheme 9. MS fragmentation patterns for coumarin Haploperosides A (**9**).

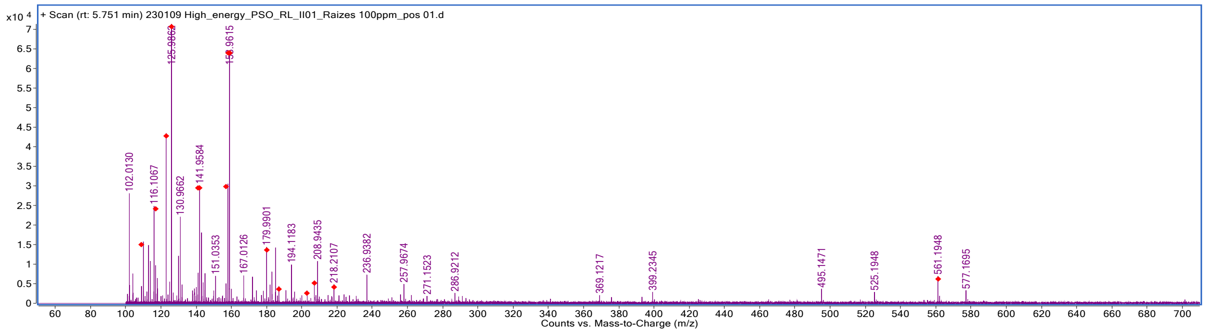

MS[1] spectrum from Moriramulosid B 6-glylceroyl (**10**) $[M+H]^+$ *m/z* 561.1948, error: 1.3 ppm.

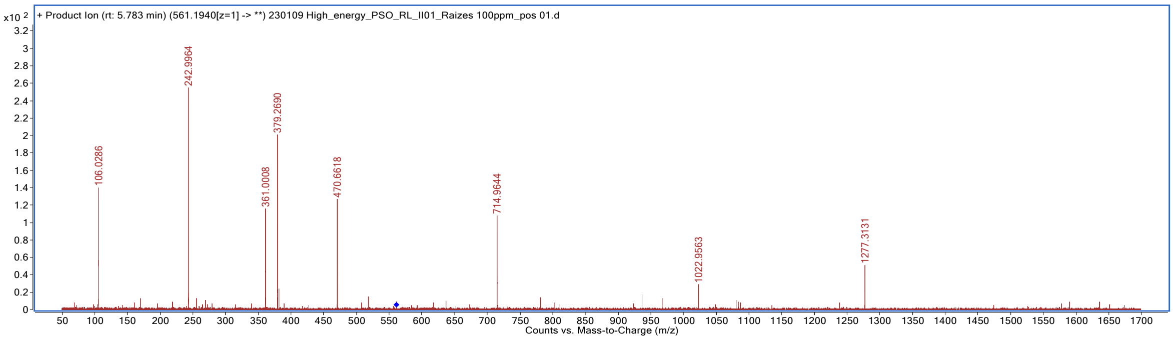

MS/MS spectrum from Moriramulosid B 6-glylceroyl (**10**).

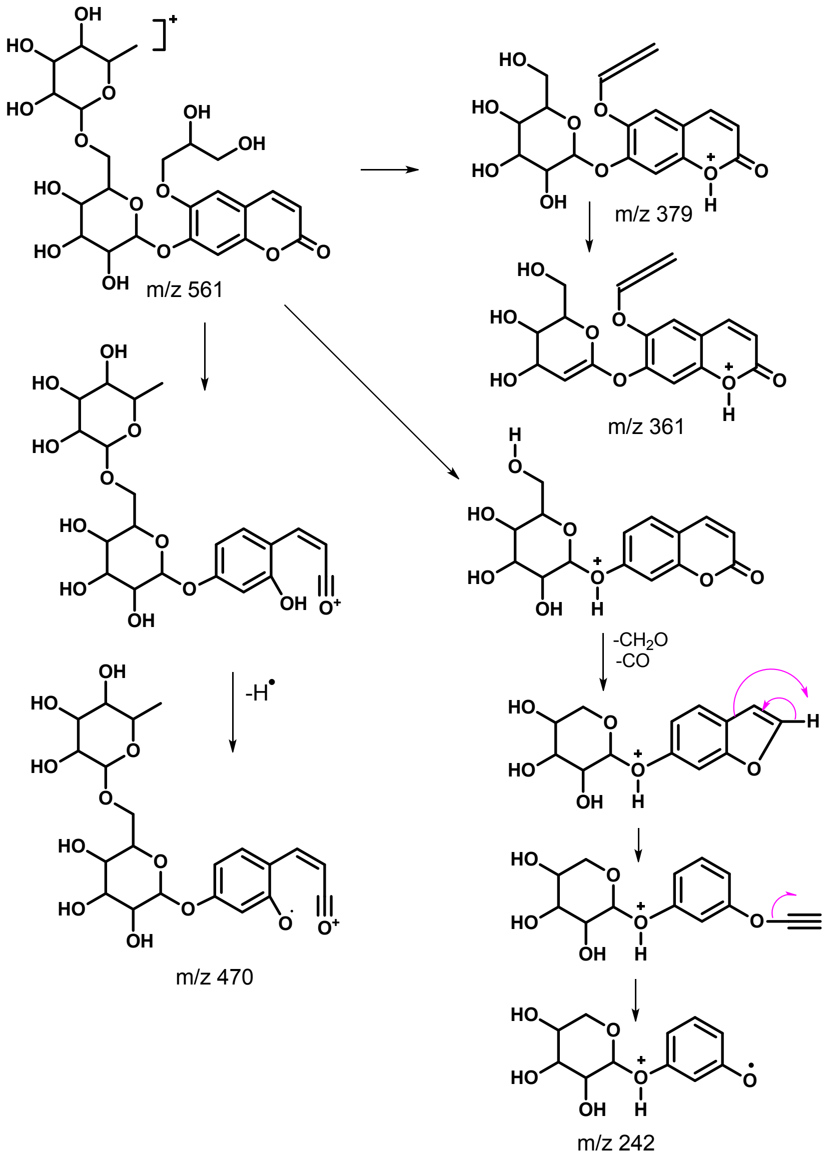

Scheme 10. MS fragmentation patterns for coumarin Moriramulosid B 6-glylceroyl (**10**).

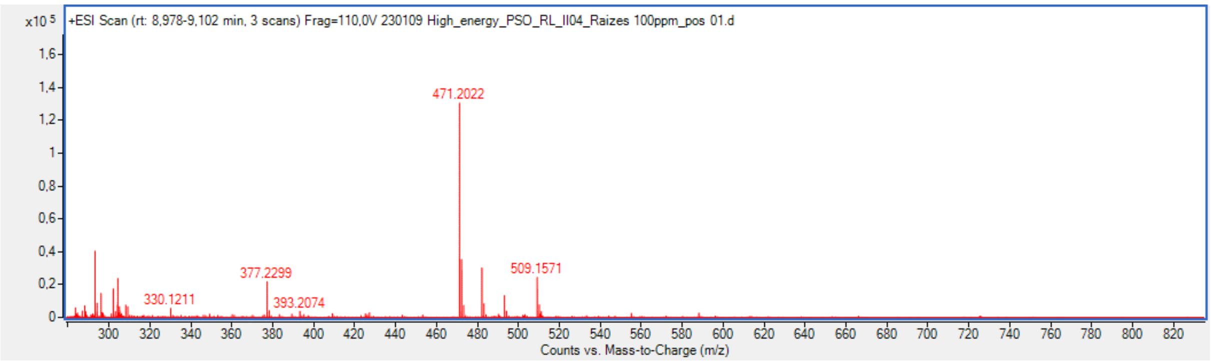

MS[1] spectrum from Limonin (**11**) $[M+H]^+$ *m/z* 471.2016, error: 0.4 ppm.

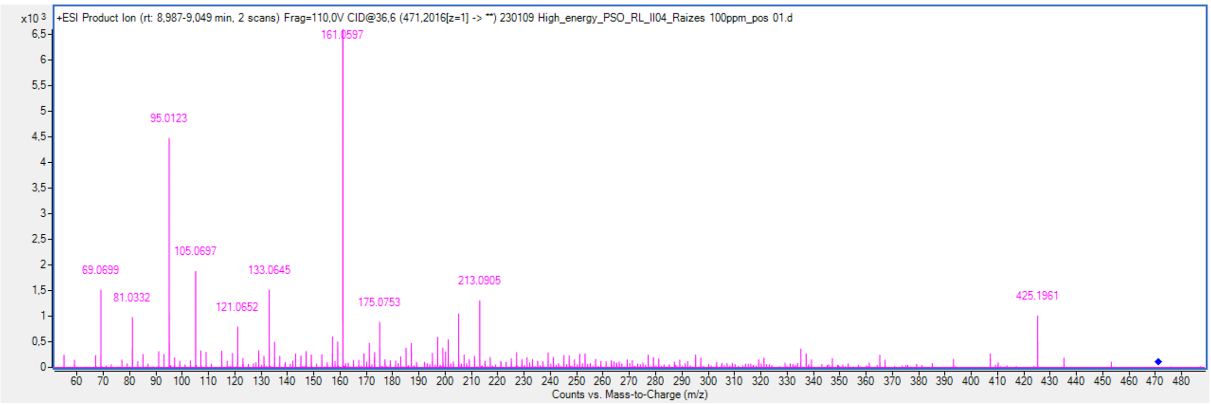

MS/MS spectrum from Limonin (**11**).

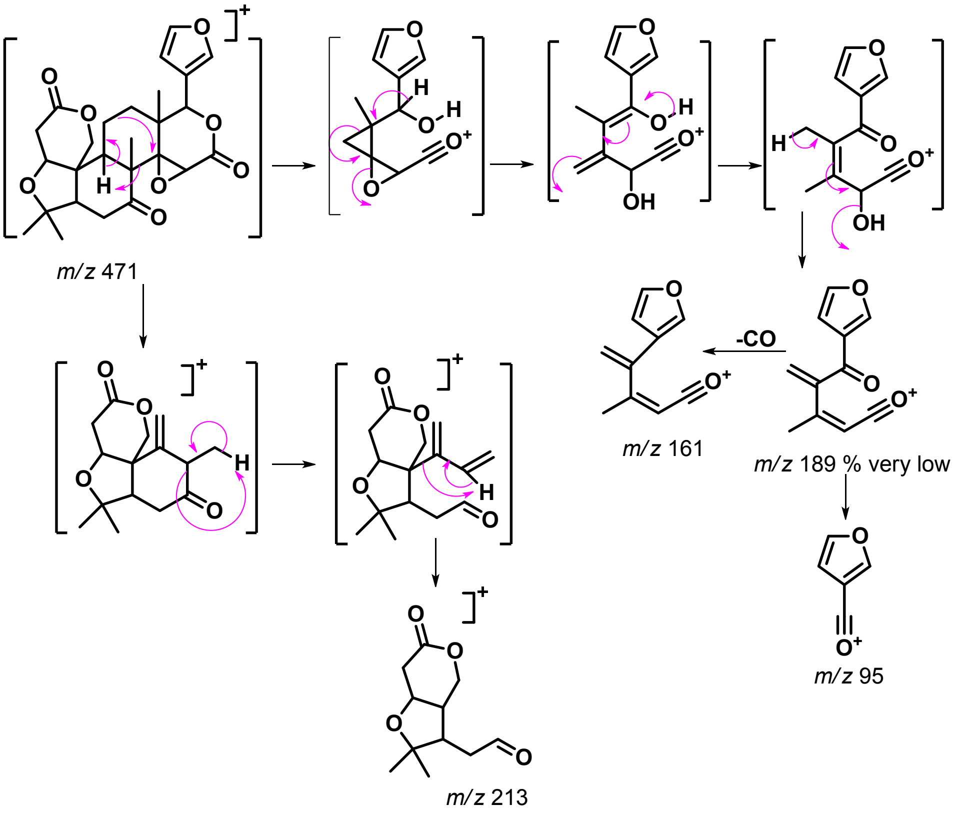

Scheme 11. MS fragmentation patterns for coumarin Limonin (**11**).

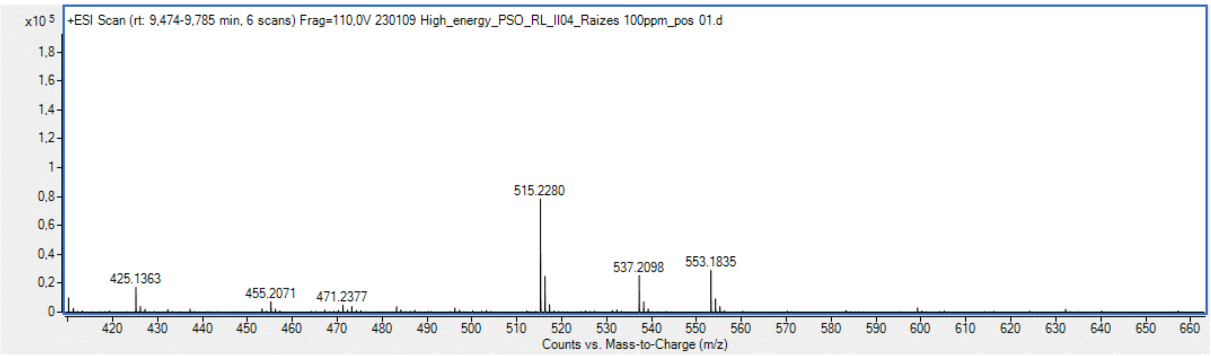

MS[1] spectrum from Nomilin (**12**)) $[M+H]^+$ *m/z* 515.2280, error: 0.4 ppm.

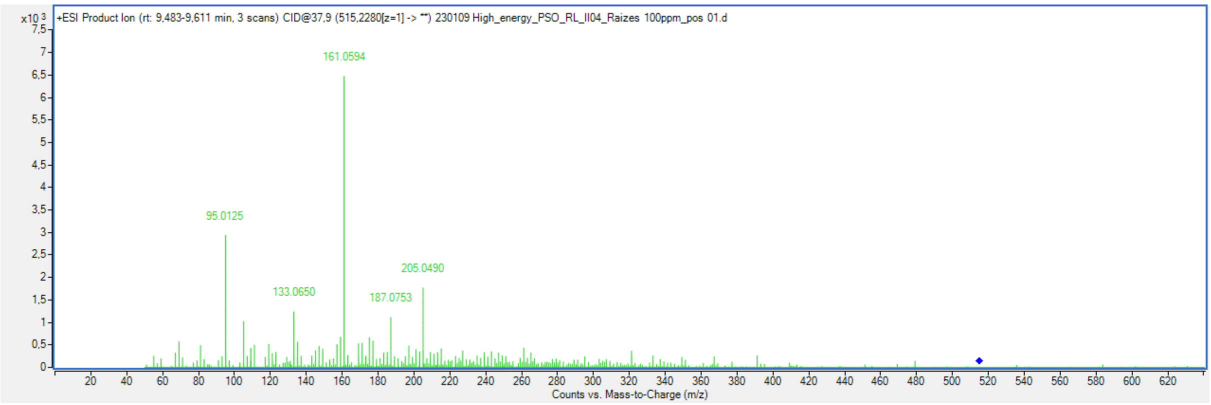

MS/MS spectrum from Nomilin (**12**).

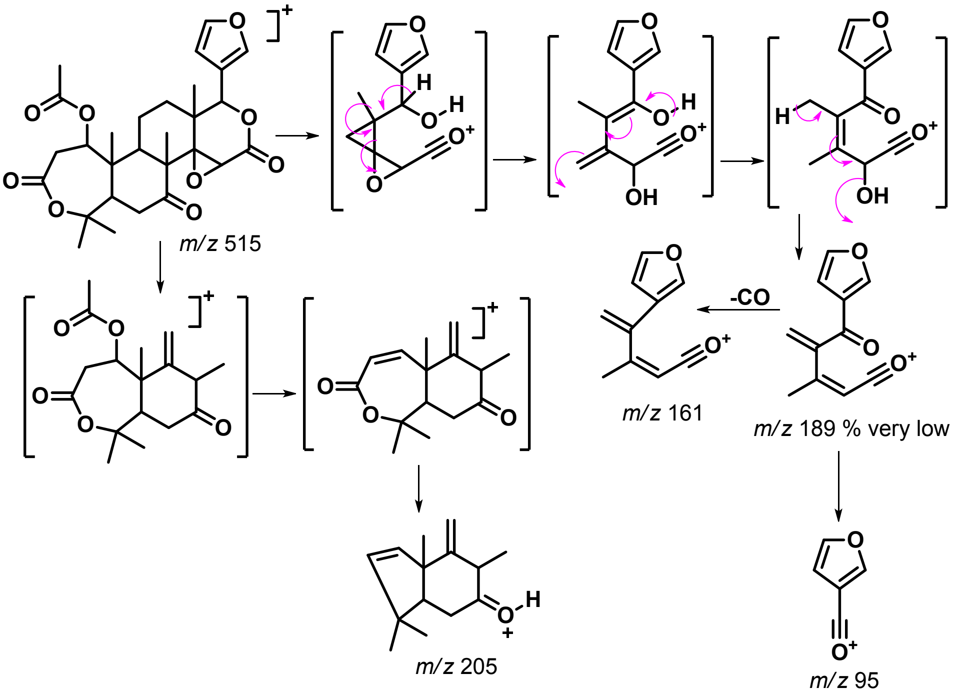

Scheme 12. MS fragmentation patterns for coumarin Nomilin (**12**).

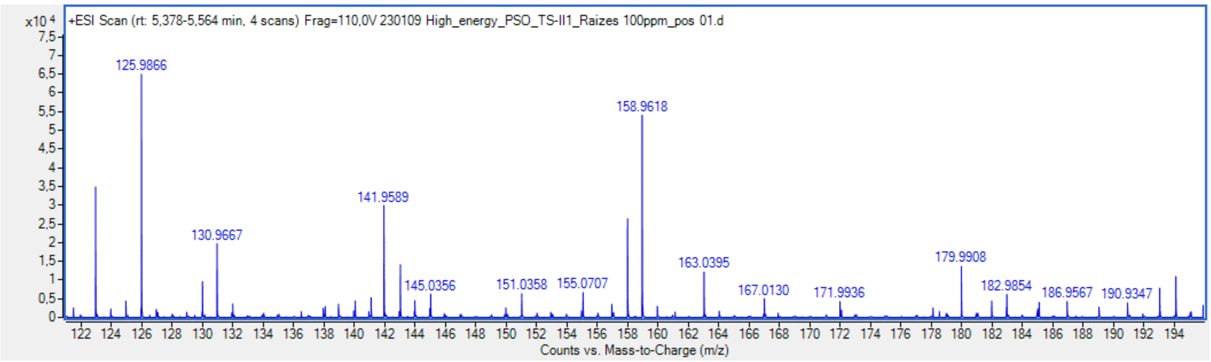

MS[1] spectrum from Umbelliferone (**13**),[M+H]$^+$ *m/z* 163.0391, error: 0.6 ppm.

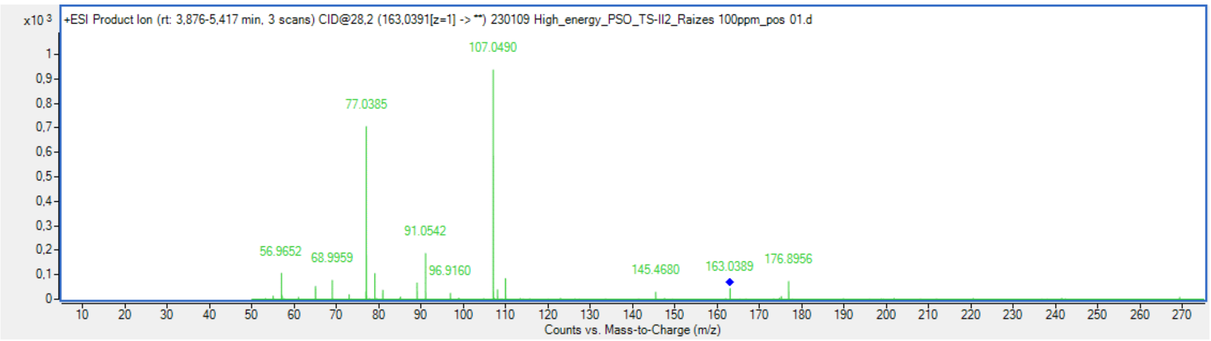

MS/MS spectrum from Umbelliferone (**13**).

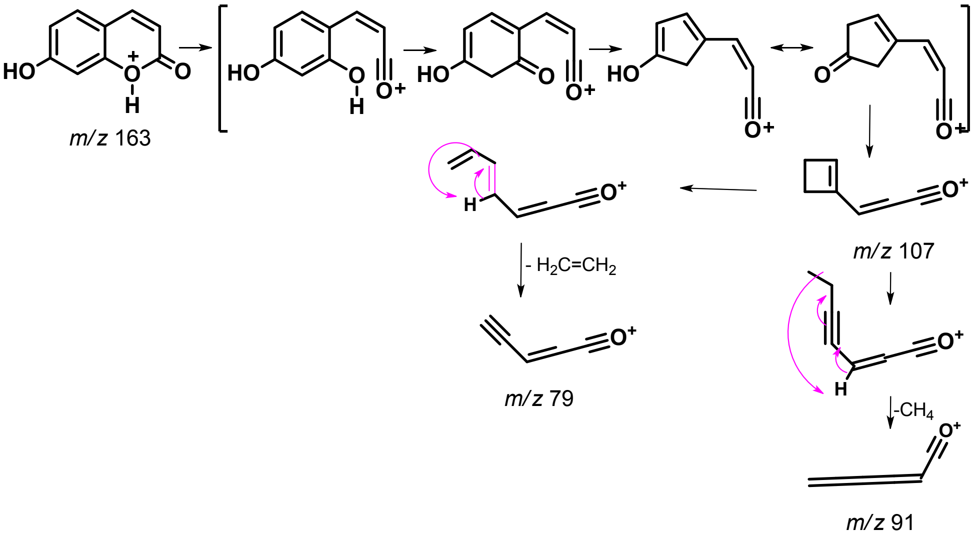

Scheme 13. MS fragmentation patterns for coumarin Umbelliferone (**13**).

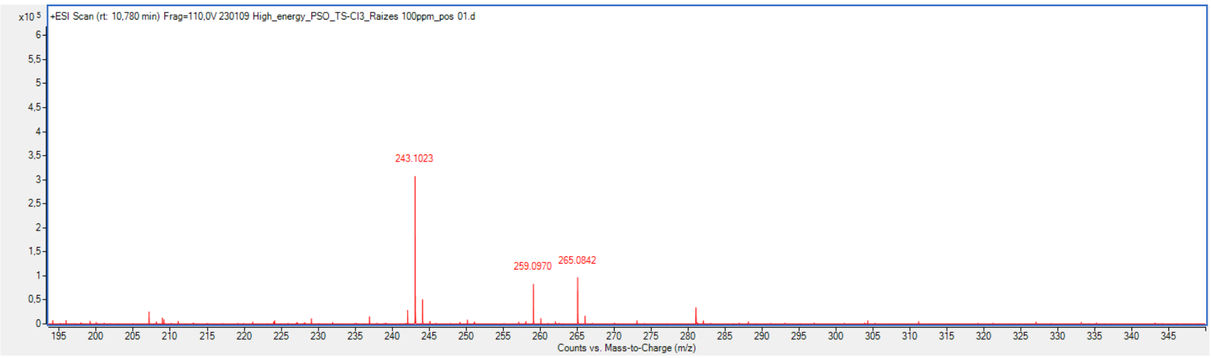

MS[1] spectrum from Citrubuntin (**14**), [M+H]$^+$ *m/z* 243.1023, error: 3.2 ppm.

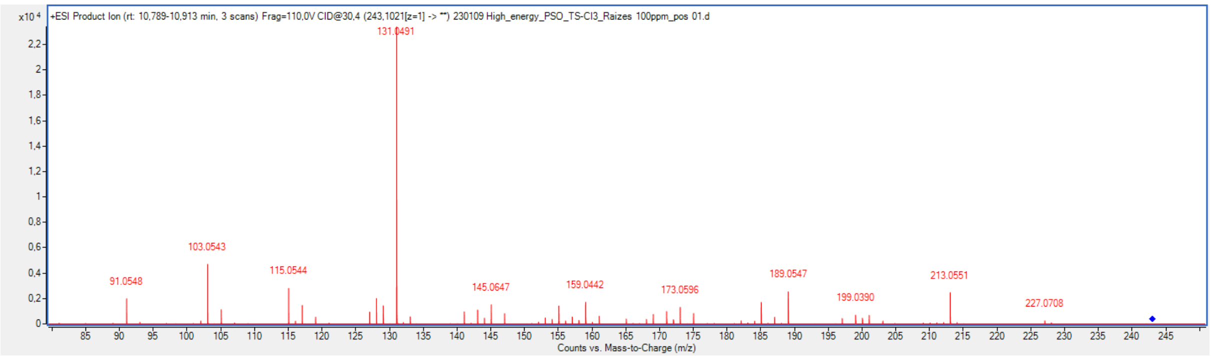

MS/MS spectrum from Citrubuntin (**14**).

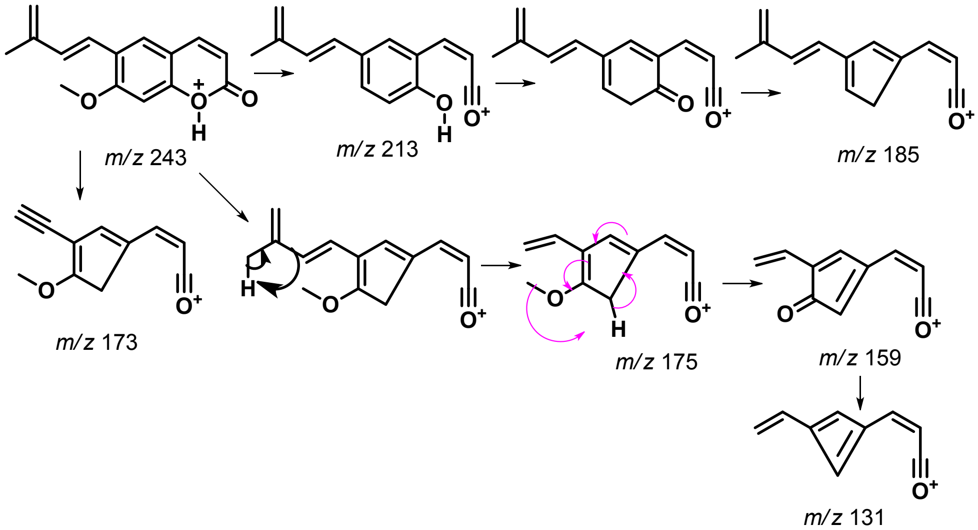

Scheme 14. MS fragmentation patterns for coumarin Citrubuntin (**14**).

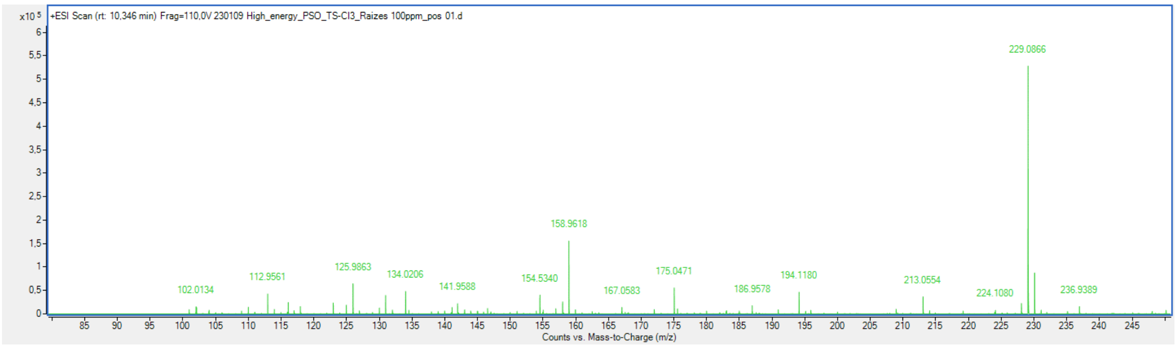

MS[1] spectrum from Seselin (**15**), $[M+H]^+$ *m/z* 229.0866, error: 2.2 ppm.

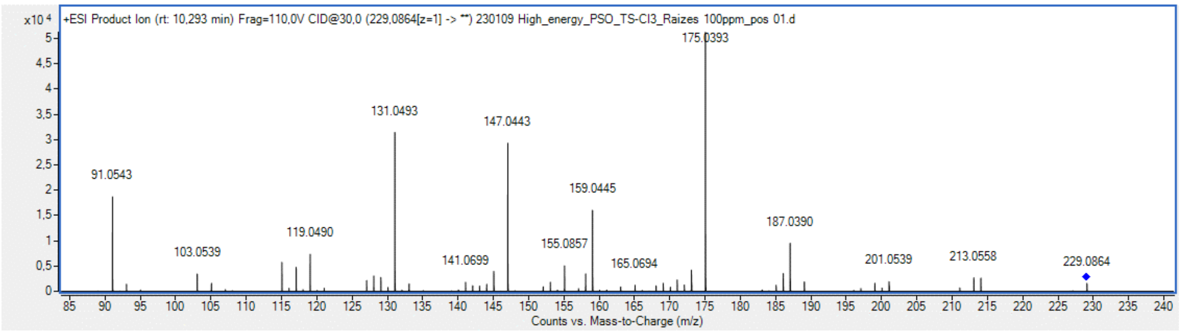

MS/MS spectrum from Seselin (**15**).

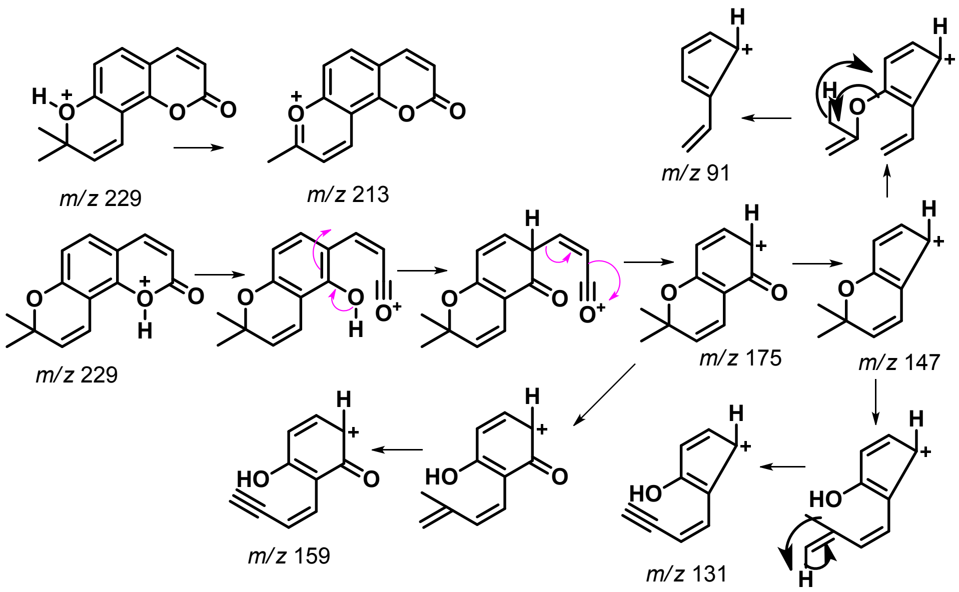

Scheme 15. MS fragmentation patterns for coumarin Seselin (**15**).

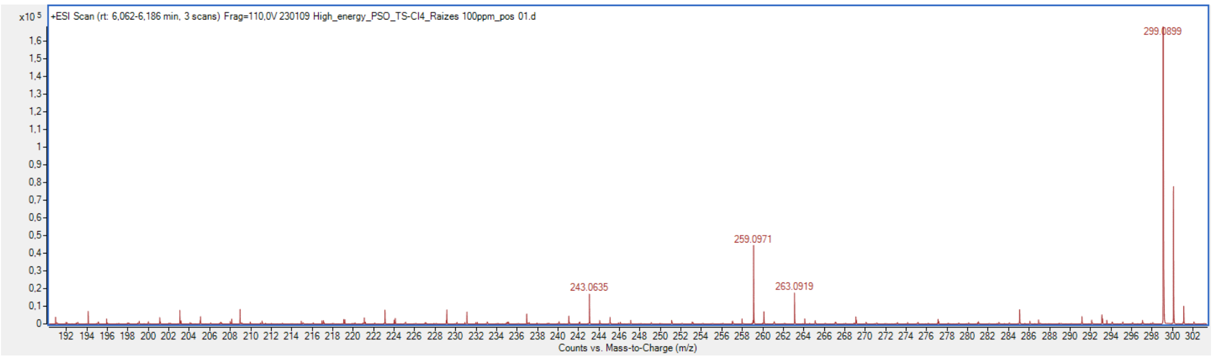

MS[1] spectrum from Luvangetin (**16**), $[M+H]^+$ *m/z* 259.0971, error: 2.7 ppm.

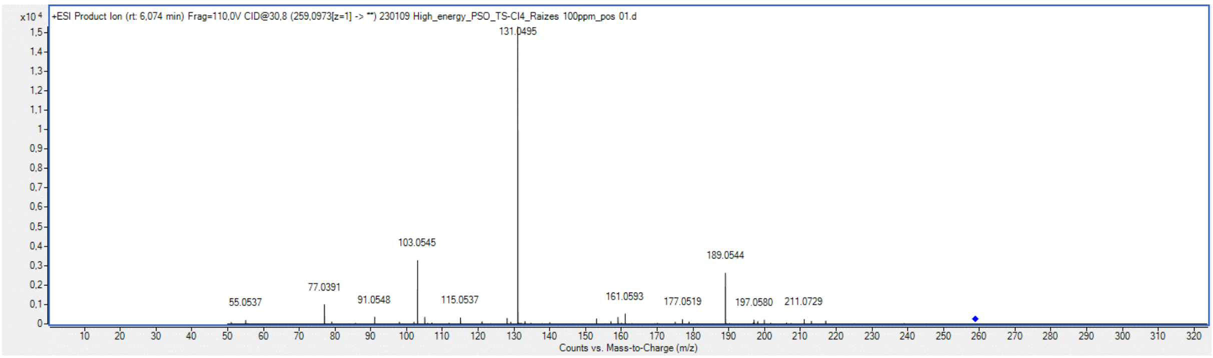

MS/MS spectrum from Luvangetin (**16**).

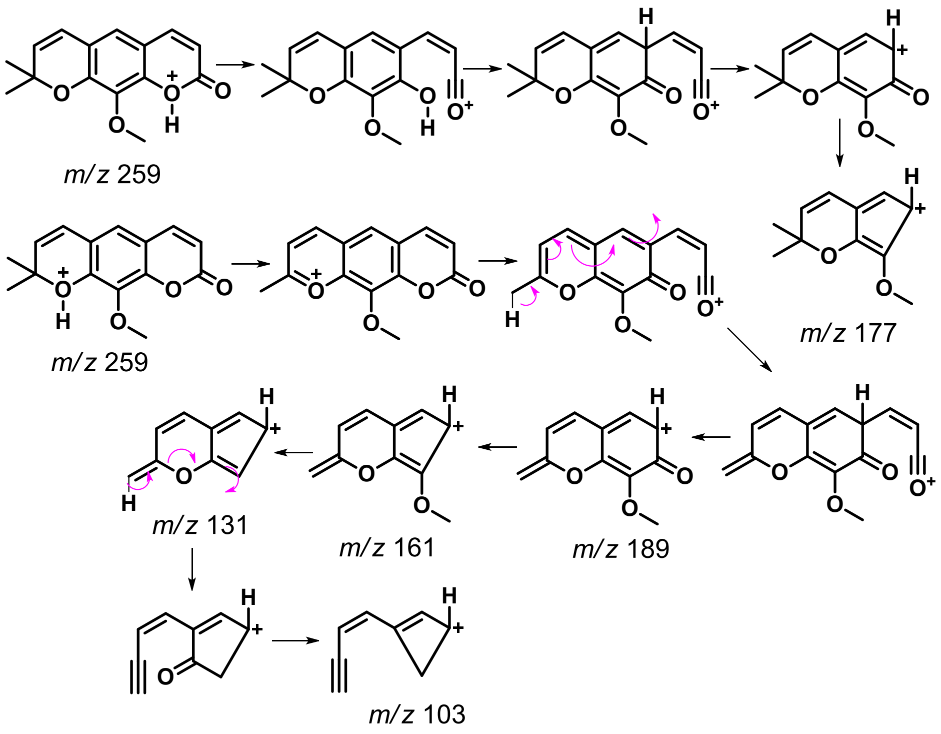

Scheme 16. MS fragmentation patterns for coumarin Luvangetin (**16**).

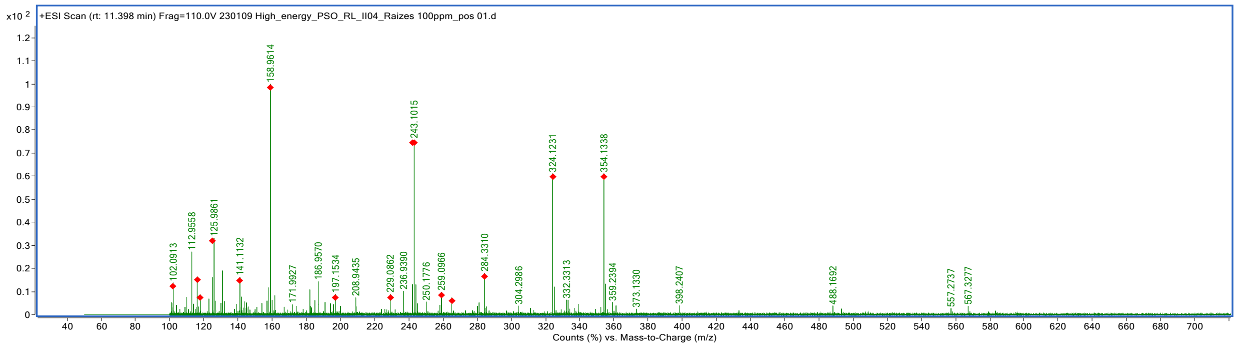

MS[1] spectrum from 5-Hydroxynoracronycine (**17**), $[M+H]^+$ *m/z* 324.1231, error: 0.3 ppm.

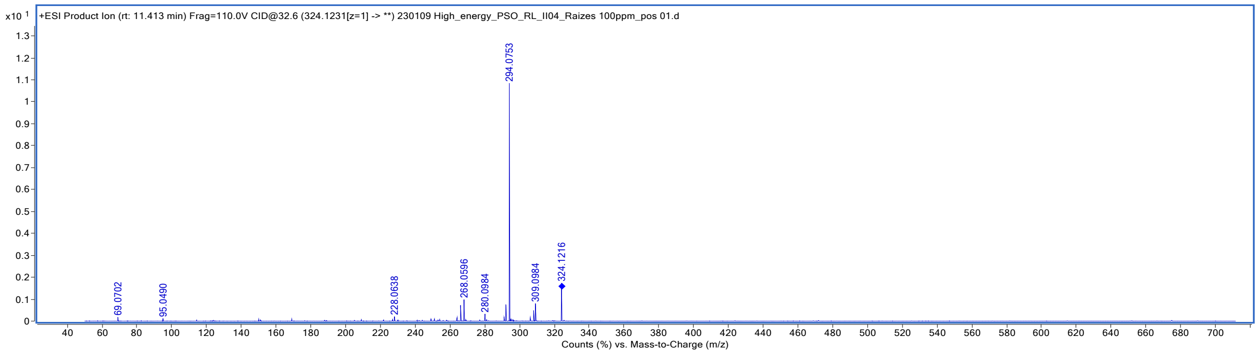

MS/MS spectrum from 5-Hydroxynoracronycine (**17**).

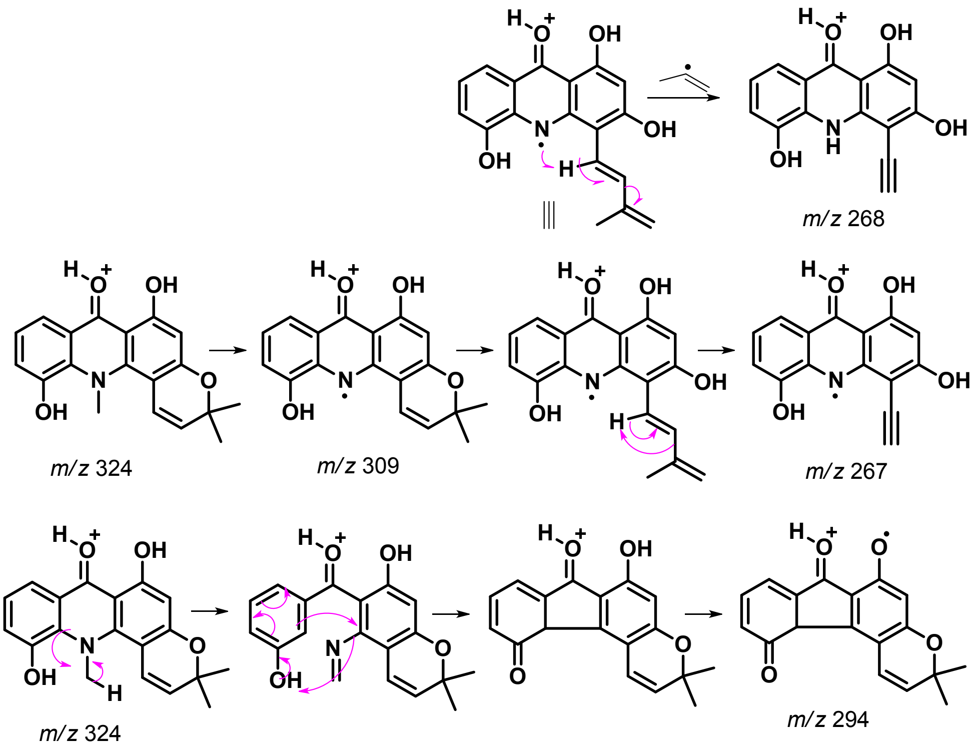

Scheme 17. MS fragmentation patterns for alkaloid 5-Hydroxynoracronycine (**17**).

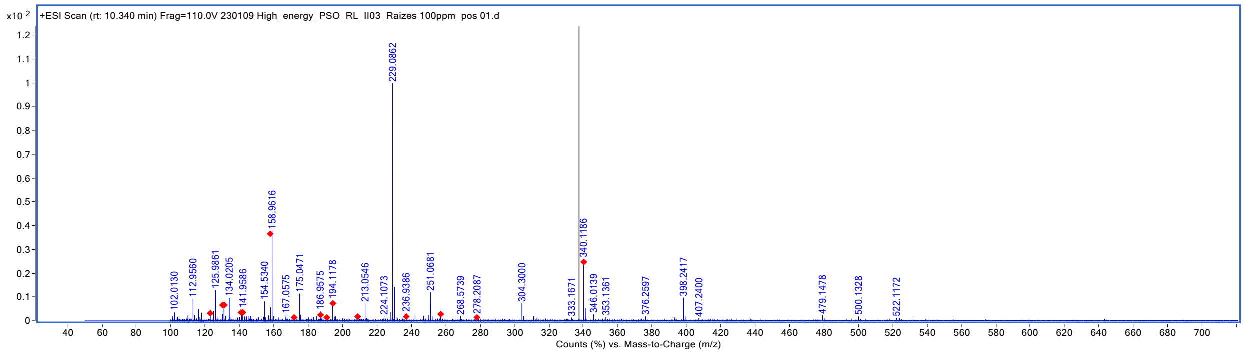

MS[1] spectrum from Citracridone III (**18**), $[M+H]^+$ *m/z* 340.1186, error: 2.0 ppm.

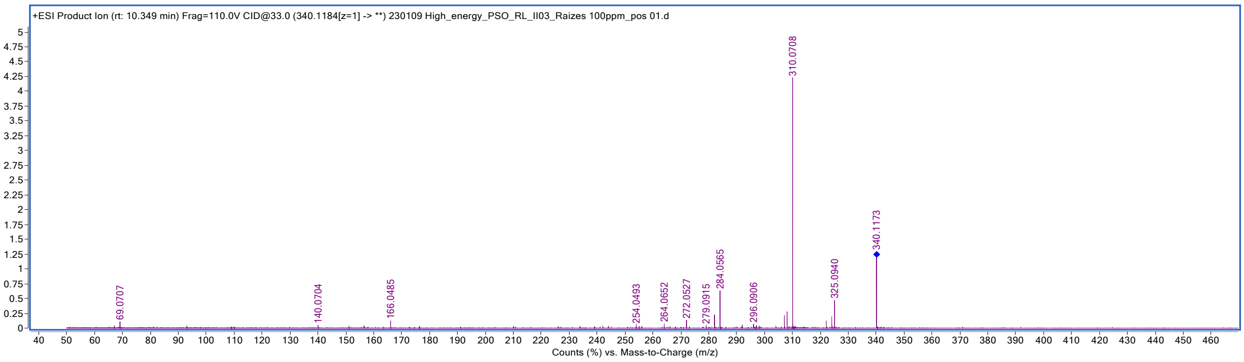

MS/MS spectrum from Citracridone III (**18**).

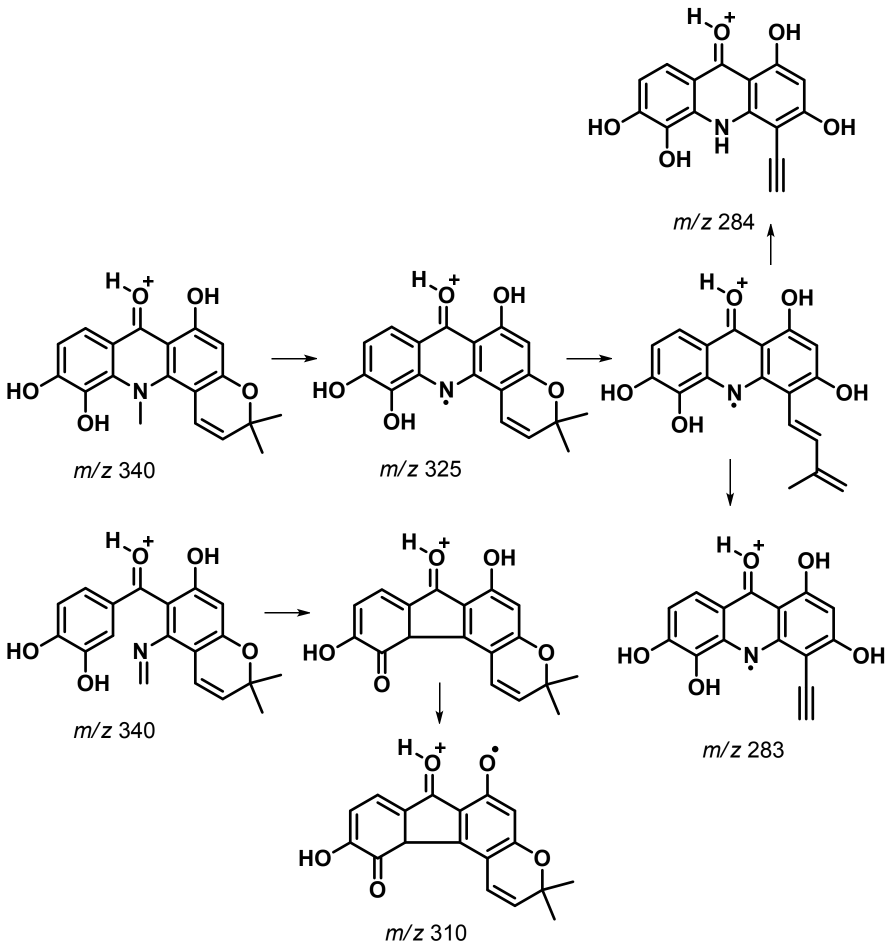

Scheme 18. MS fragmentation patterns for alkaloid Citracridone III (**18**), for the formation of *m/z* 284 see Scheme 17.

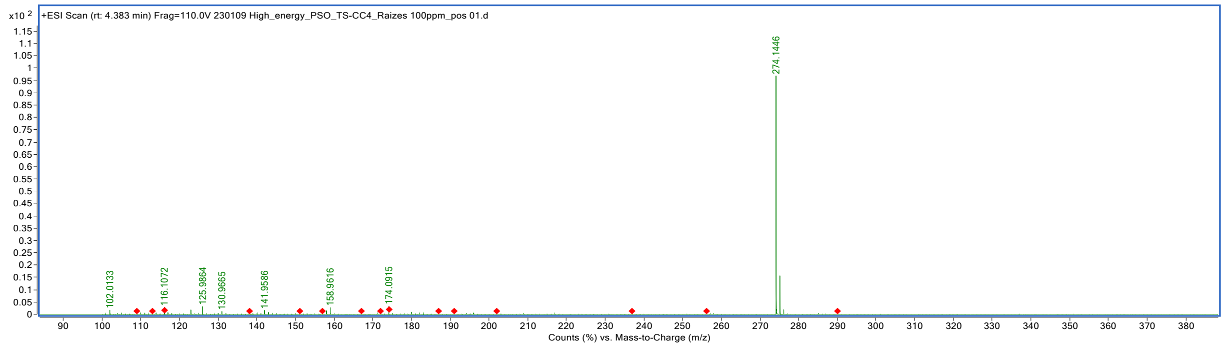

MS[1] spectrum from Clausine-L (**19**), $[M+H]^+$ *m/z* 256.1336, error: 0.3 ppm.

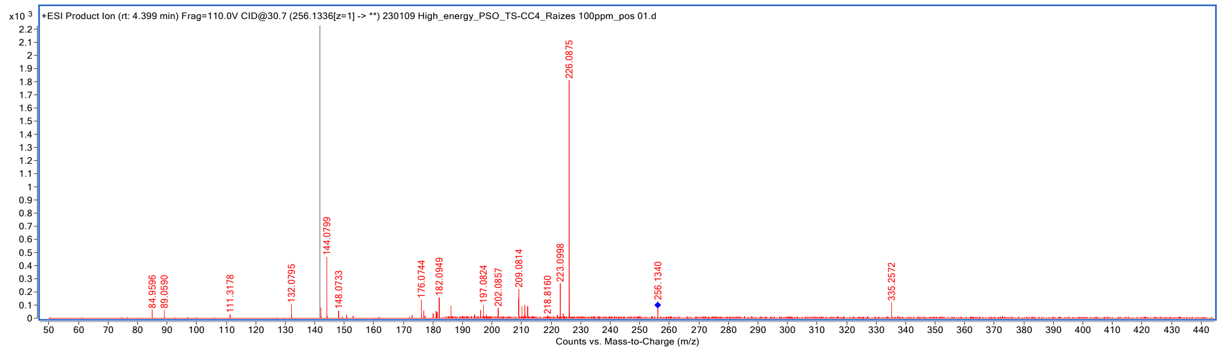

MS/MS spectrum from Clausine-L (**19**).

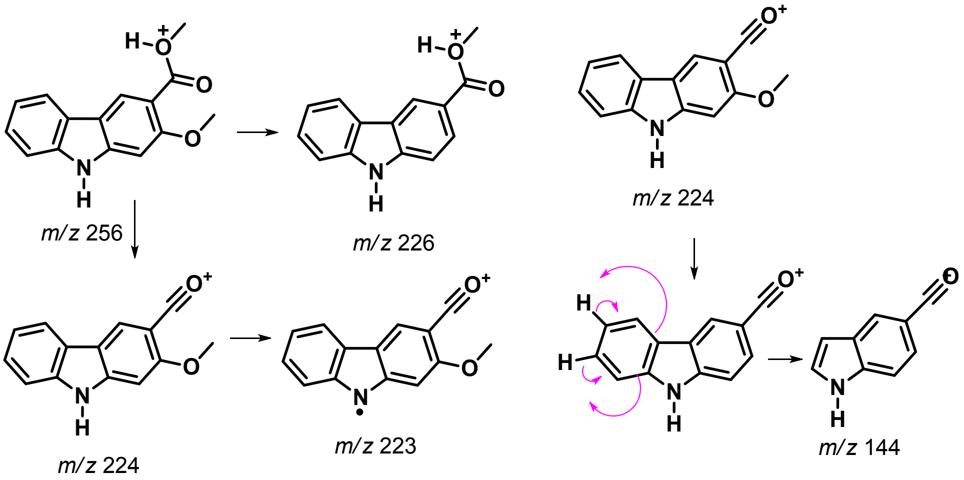

Scheme 19. MS fragmentation patterns for alkaloid Clausine-L (**19**).

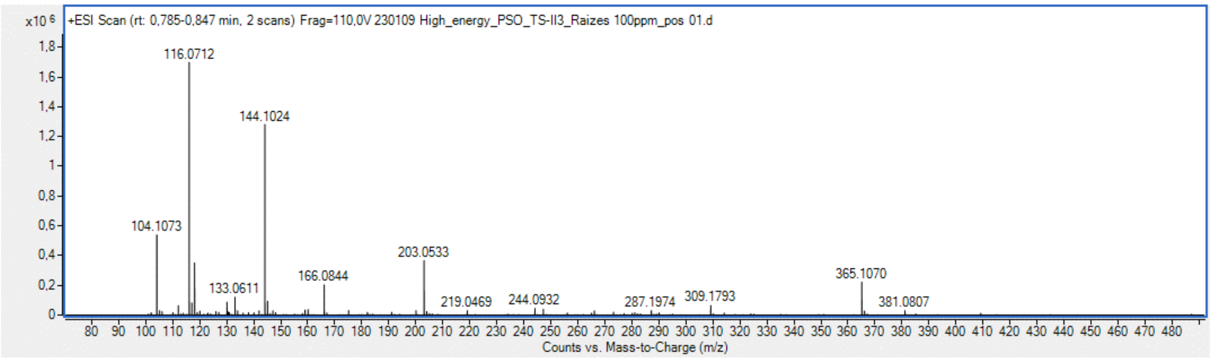

MS[1] spectrum from Cytidine (**20**), $[M+H]^+$ *m/z* 244.0932.

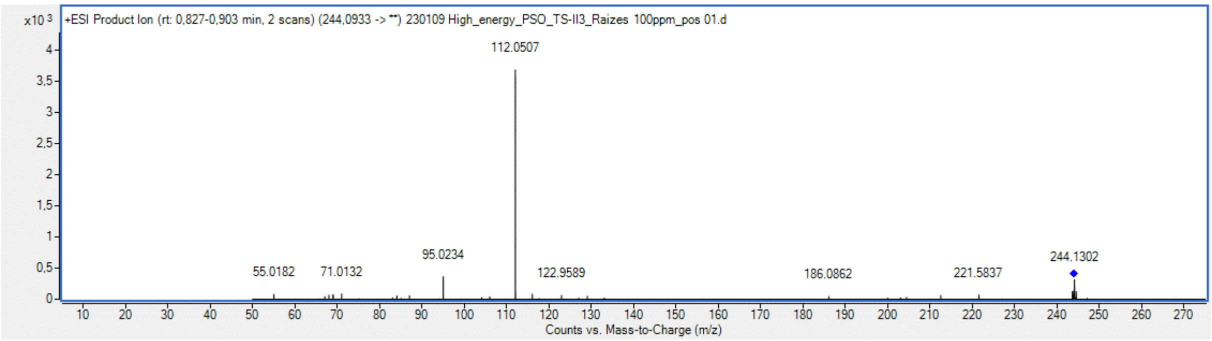

MS/MS spectrum from Cytidine (**20**).

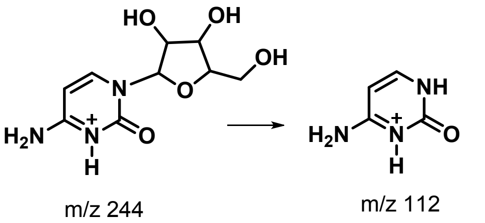

Scheme 20. MS fragmentation patterns for Nucleoside Cytidine (**20**).

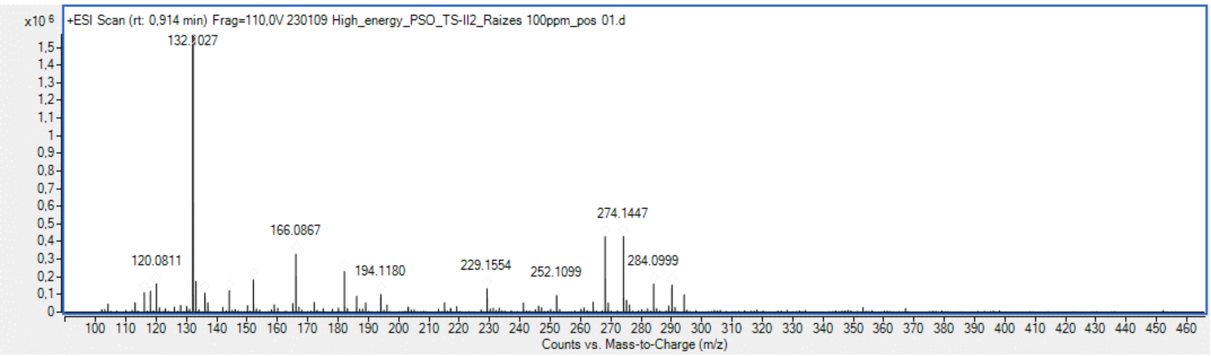

MS[1] spectrum from Guanosine (**21**), $[M+H]^+$ *m/z* 284.0999, error: 3.5 ppm.

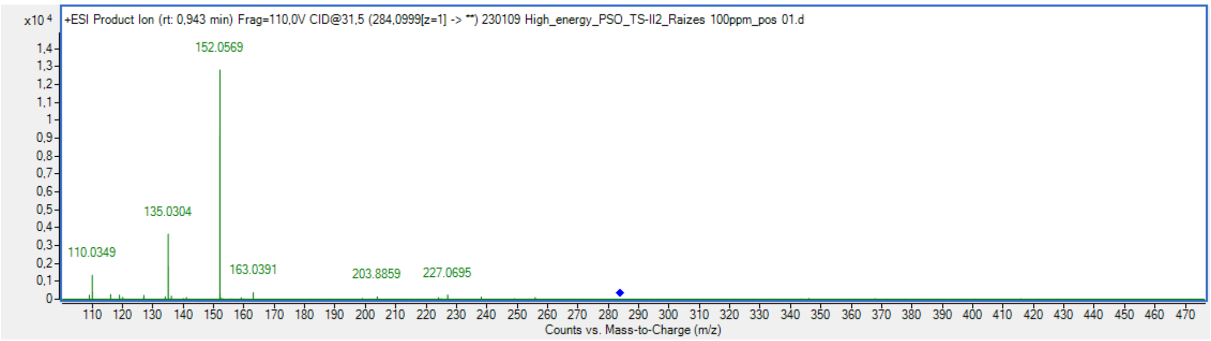

MS/MS spectrum from Guanosine (**21**).

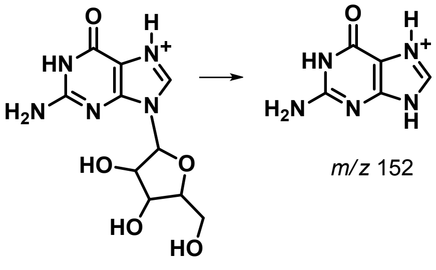

Scheme 21. MS fragmentation patterns for Nucleoside Guanosine (**21**).

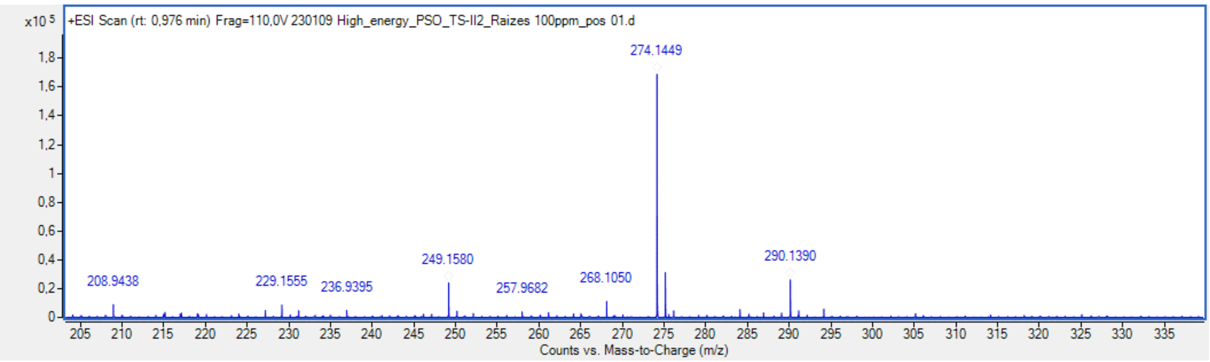

MS[1] spectrum from Adenosine (**22**), $[M+H]^+$ *m/z* 268.1050, error: 3.7 ppm.

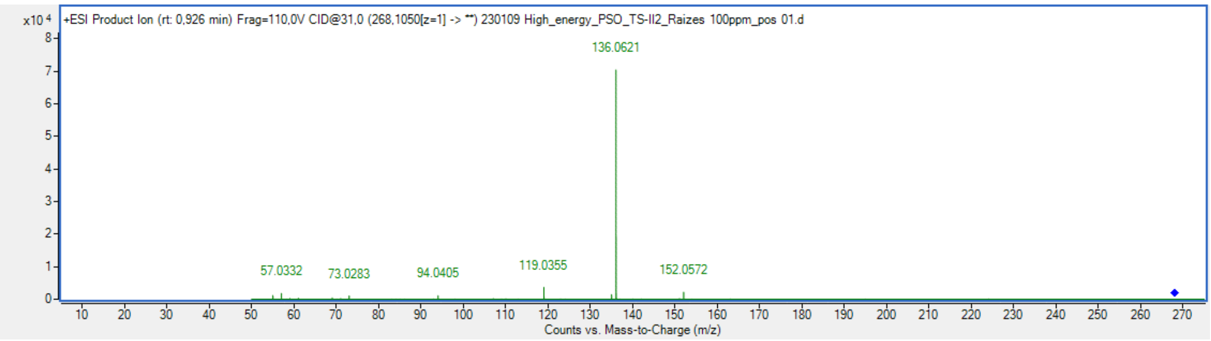

MS/MS spectrum from Adenosine (**22**).

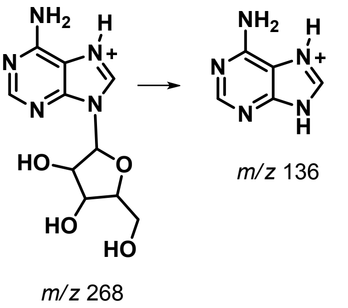

Scheme 22. MS fragmentation patterns for Nucleoside Adenosine (**22**).

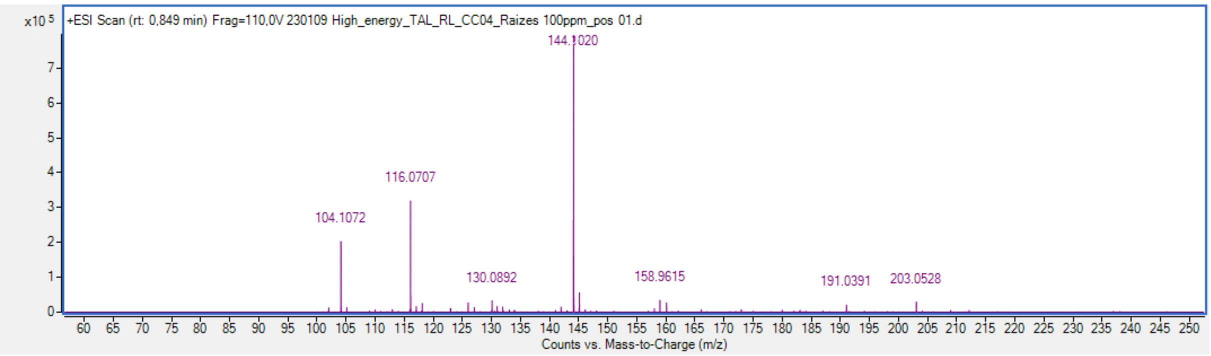

MS[1] spectrum from L-prolina (**23**) $[M+H]^+$ *m/z* 116.0713, error: 6.0 ppm.

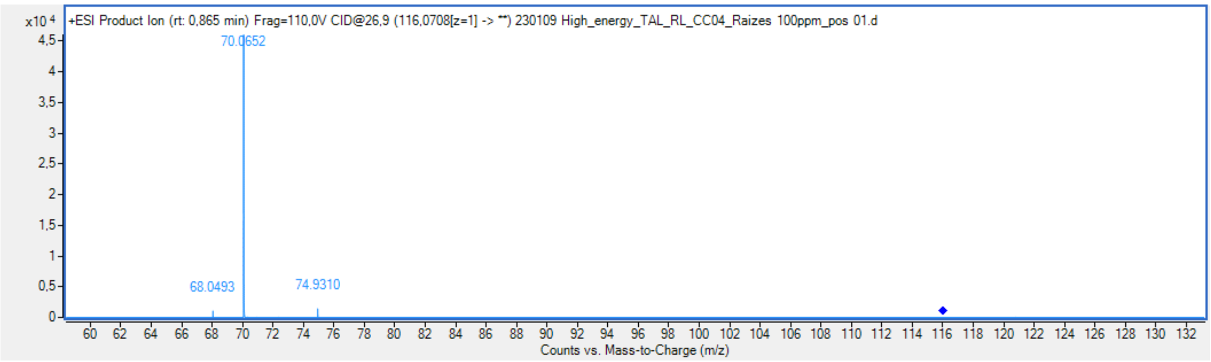

MS/MS spectrum from L-prolina (**23**).

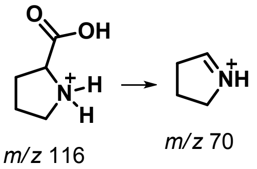

Scheme 23. MS fragmentation patterns for Amino acid L-Proline (**23**).

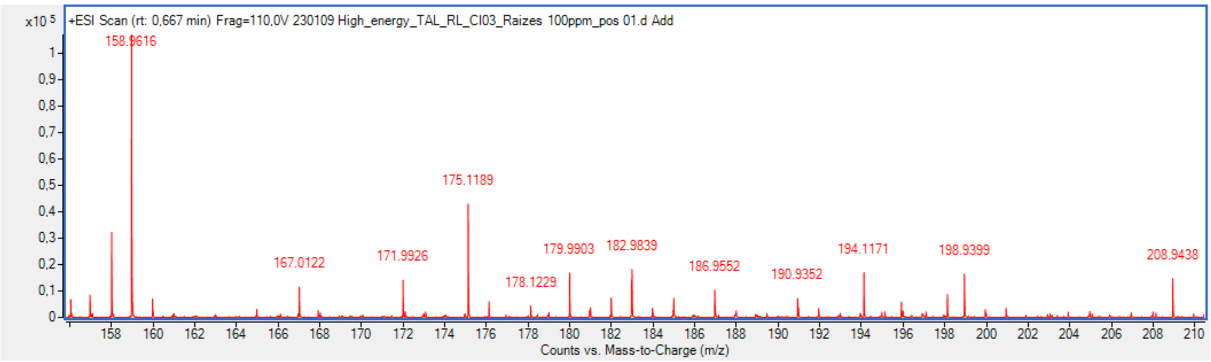

MS[1] spectrum from L-arginina (**24**) [M+H]+ *m/z* 175.1186, error: 2.2 ppm.

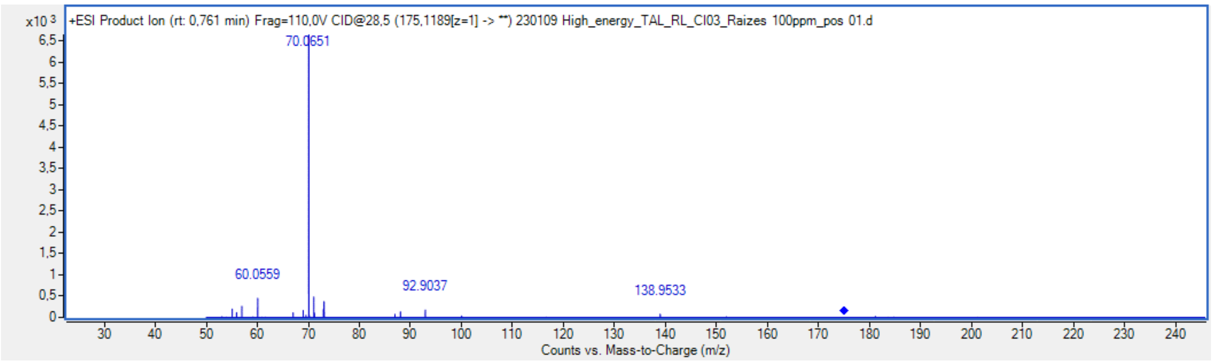

MS/MS spectrum from L-arginina (**24**).

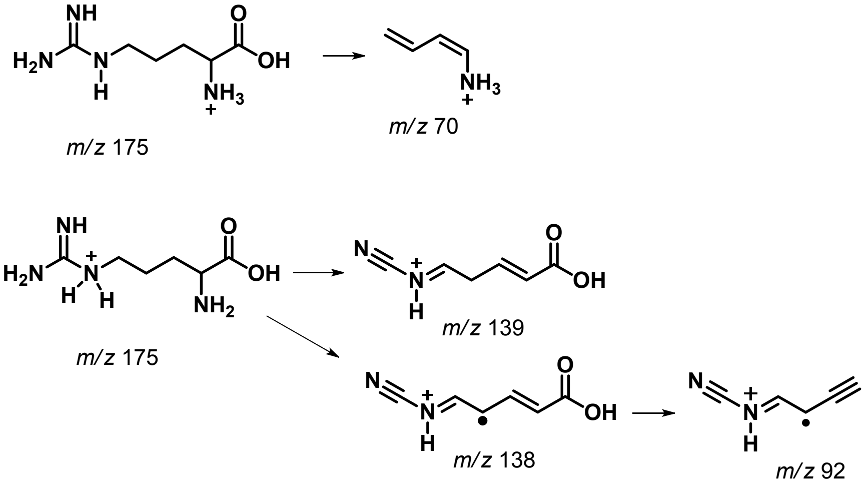

Scheme 24. MS fragmentation patterns for Amino acid L-Argenine (**24**).

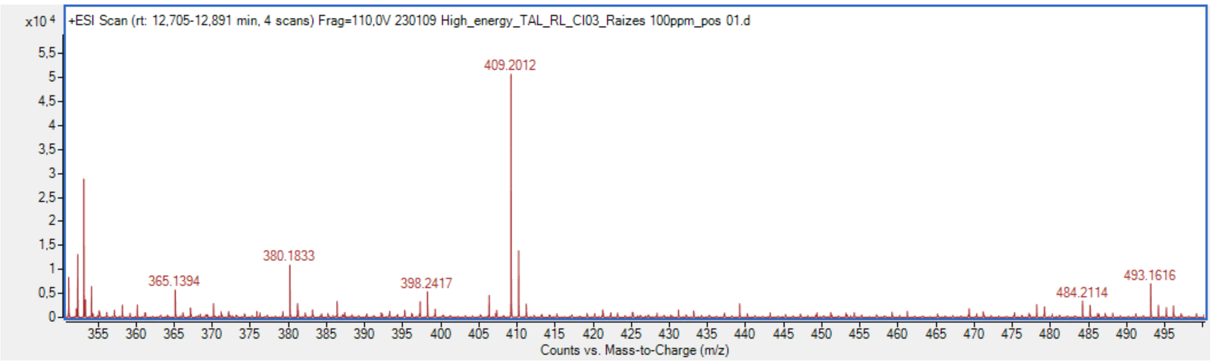

MS[1] spectrum from Lochnocarpol A (**25**) $[M+H]^+$ *m/z* 409.2011, error: 0.2 ppm.

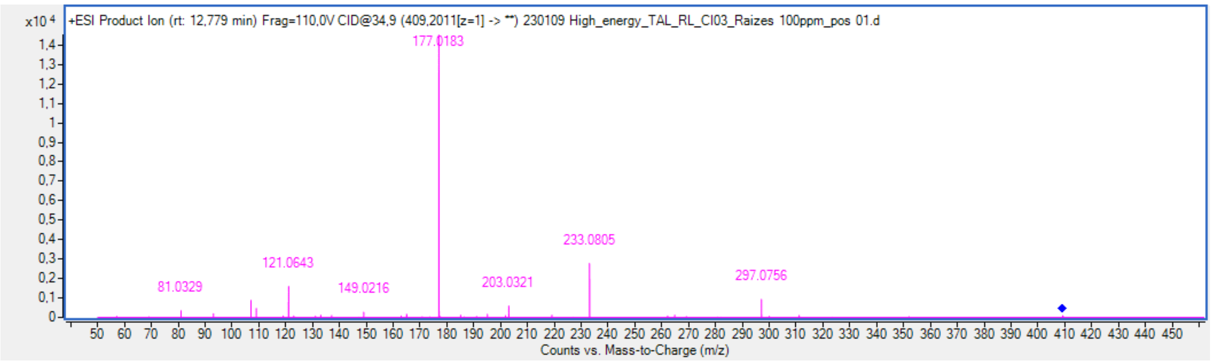

MS/MS spectrum from Lochnocarpol A (**25**).

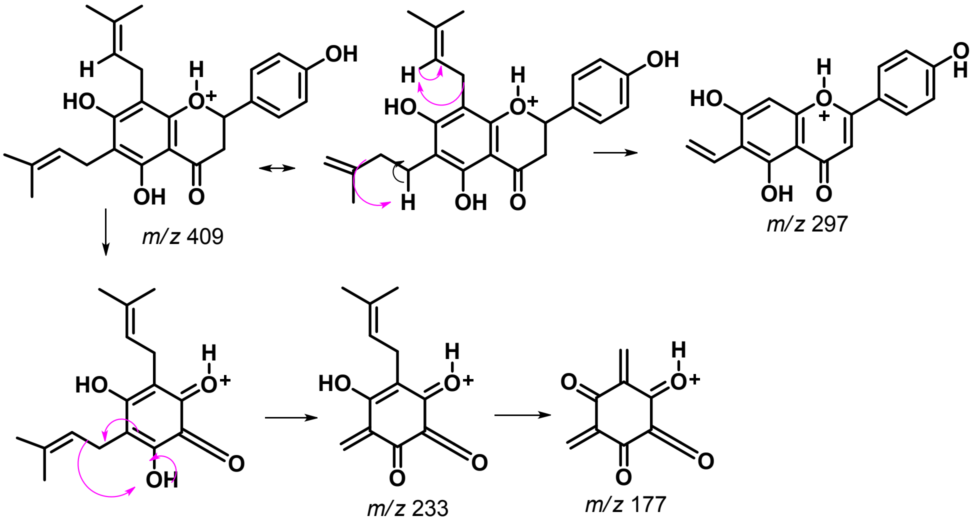

Scheme 25. MS fragmentation patterns for flavonoid Lochnocarpol A (**25**).

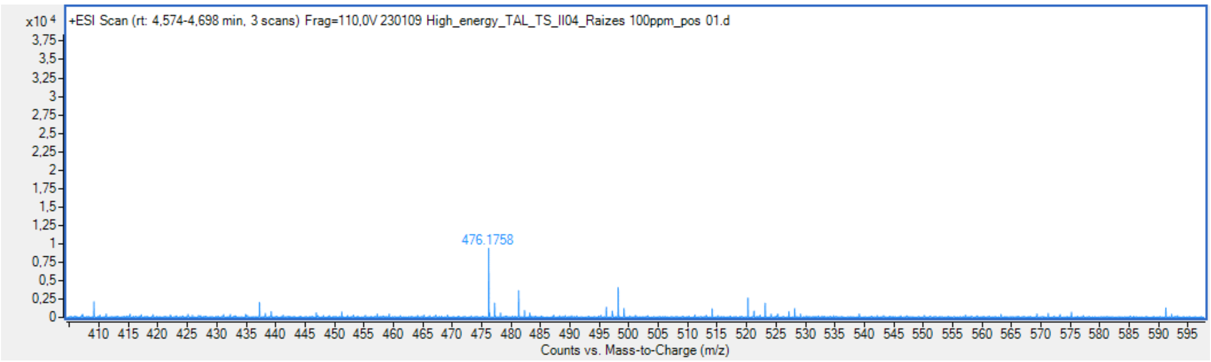

MS[1] spectrum from Amygdalin amide (**26**), $[M+H]^+$ *m/z* 476.1758, error: 0.4 ppm.

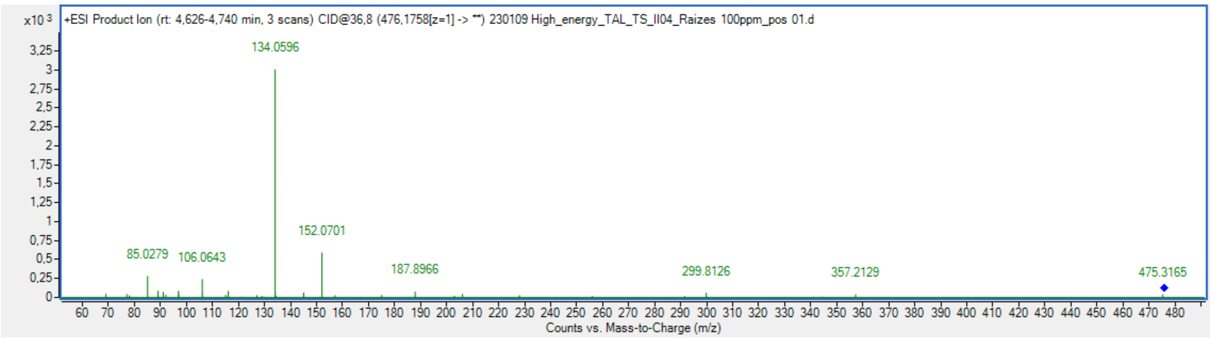

MS/MS spectrum from Amygdalin amide (**26**).

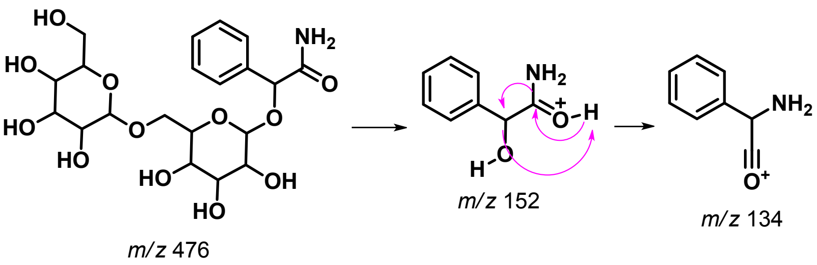

Scheme 26. MS fragmentation patterns for Amygdalin amide (**26**).

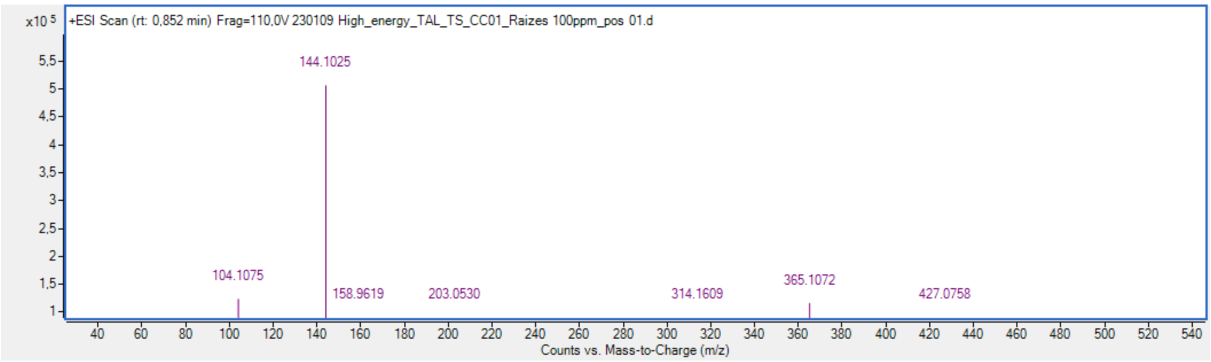

MS[1] spectrum from Stachydrine (**27**), $[M+H]^+$ *m/z* 144.1026, error: 0.4 ppm.

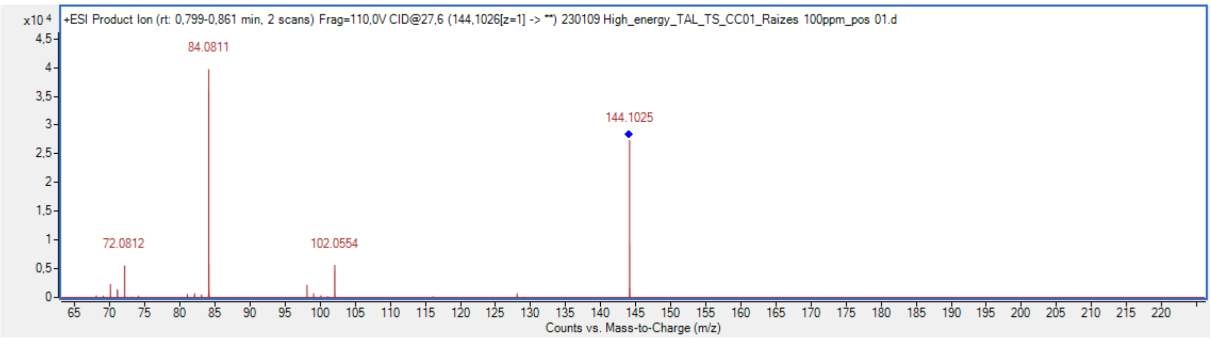

MS/MS spectrum from Stachydrine (**27**).

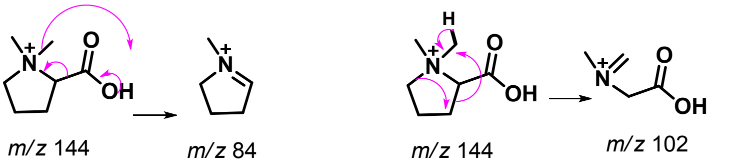

Scheme 27. MS fragmentation patterns for alkaloid Stachydrine (**27**).

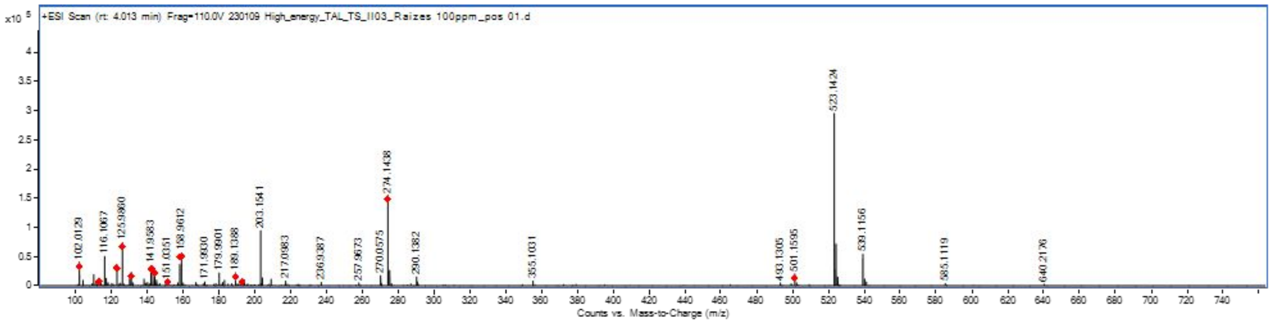

MS[1] spectrum from *N*,*N*-Dimethyltryptamine (**28**), $[M+H]^+$ *m/z* 189.1387, error: 1.1 ppm.

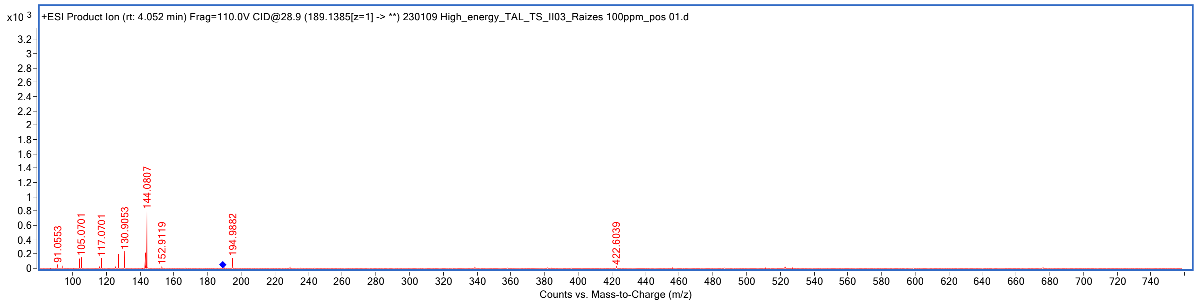

MS/MS spectrum from *N*,*N*-Dimethyltryptamine (**28**).

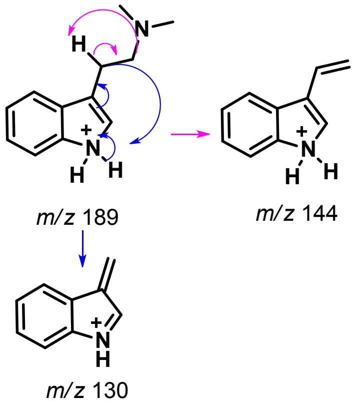

Scheme 28. MS fragmentation patterns for alkaloid *N*,*N*-Dimethyltryptamine (**28**).

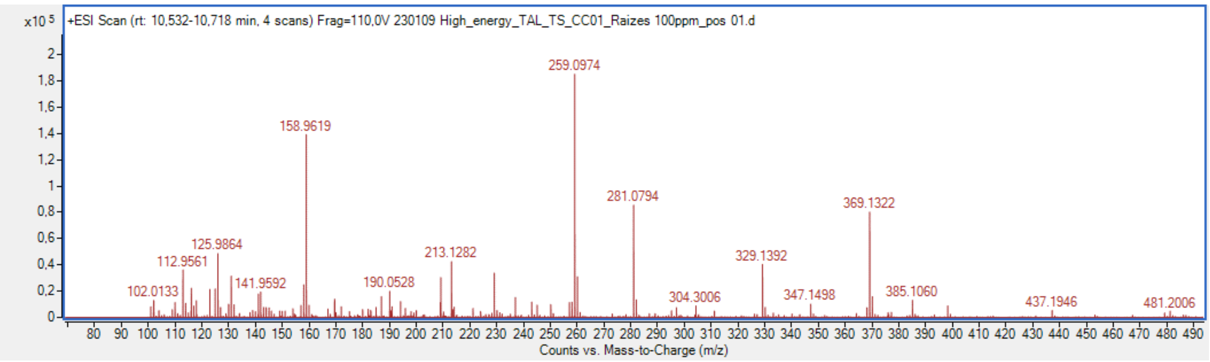

MS[1] spectrum from *N*-hexanoyl tryptamine (**29**) $[M+H]^+$ *m/z* 259.1811, error: 0.4 ppm.

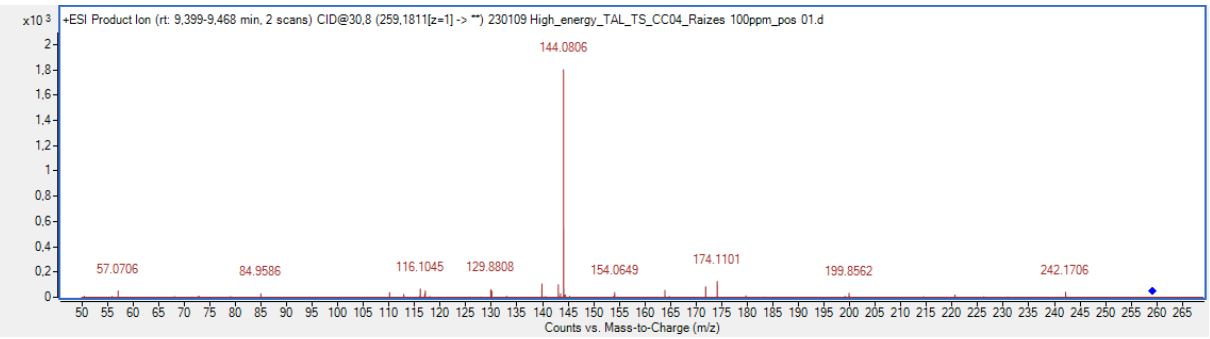

MS/MS spectrum from *N*-hexanoyl tryptamine (**29**) $[M+H]^+$ *m/z* 259.1811, error: 0.4 ppm.

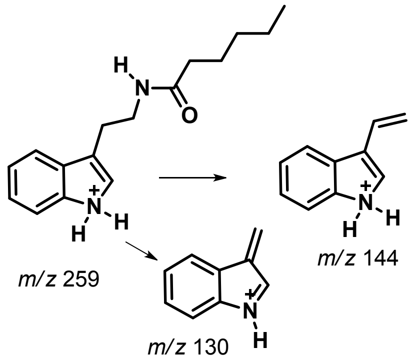

Scheme 29. MS fragmentation patterns for alkaloid *N*-Hexanoyltryptamine (**29**).

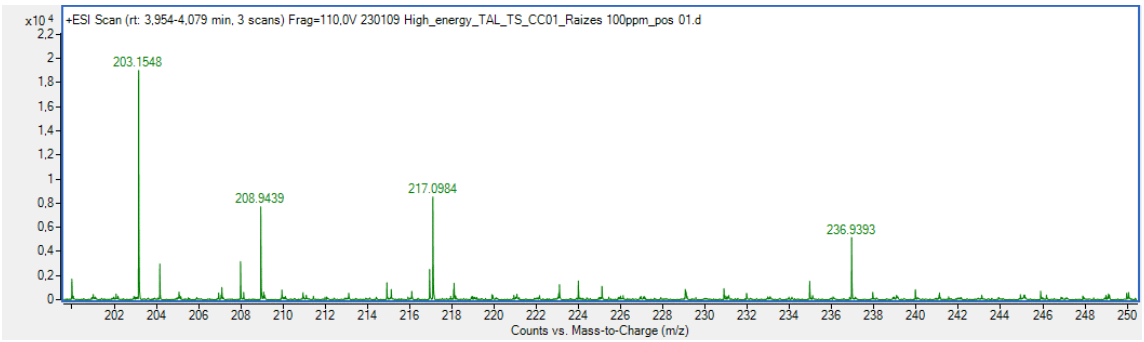

MS[1] spectrum from 5-methoxytryptophan (**30**), $[M+H]^+$ *m/z* 217.0982, error: 0.4 ppm.

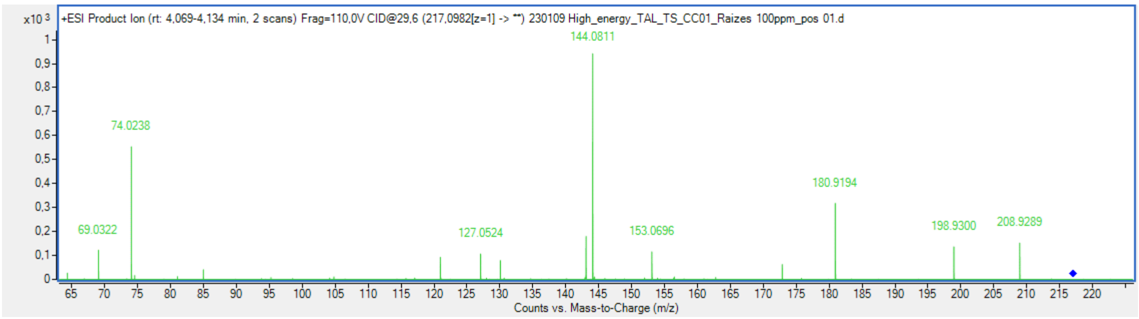

MS/MS spectrum from 5-methoxytryptophan (**30**).

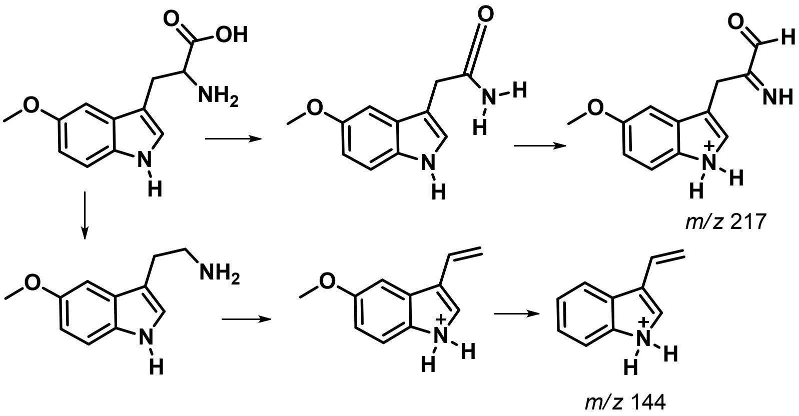

Scheme 30. MS fragmentation patterns for 5-Methoxytryptophan (**30**).

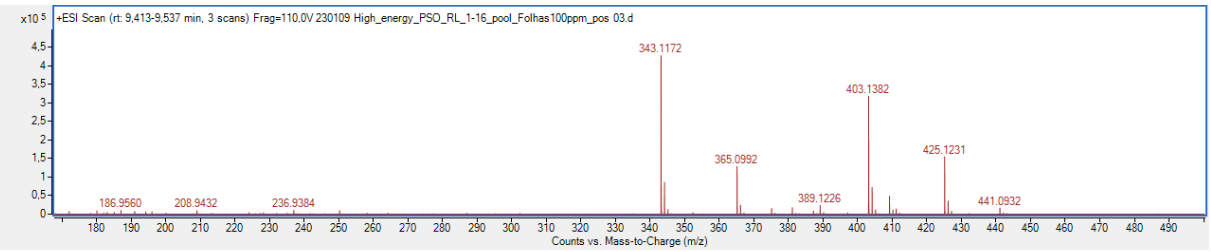

MS[1] spectrum from 6-demethoxytangeretin (**31**), $[M+H]^+$ *m/z* 343.1172, error: 1.2 ppm.

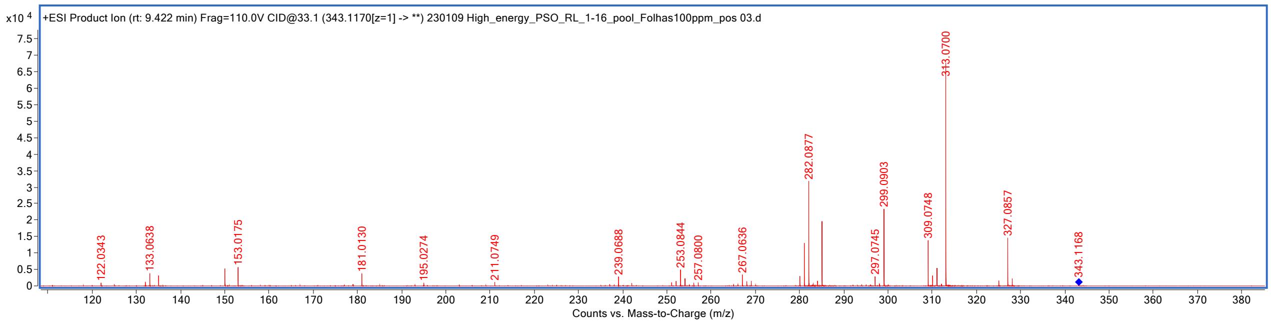

MS/MS spectrum from 6-demethoxytangeretin (**31**).

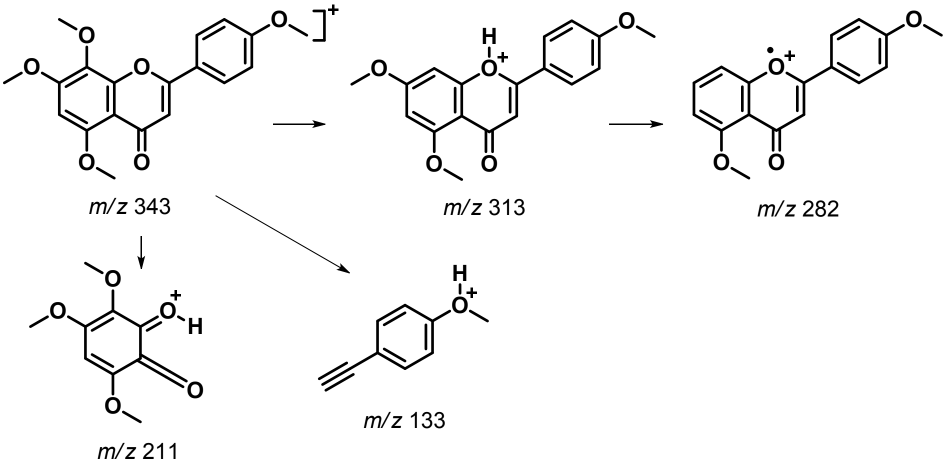

Scheme 31. MS fragmentation patterns for flavonoid 6-Demethoxytangeretin (**31**) (The loss of a methoxyl could have occurred at any position).

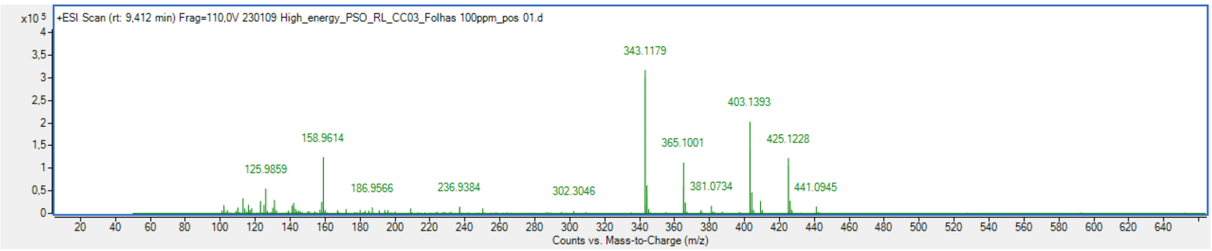

MS[1] spectrum from Tetra-*O*-methylscutellarein (**32**), $[M+H]^+$ *m/z* 343.1179, error: 0.9 ppm.

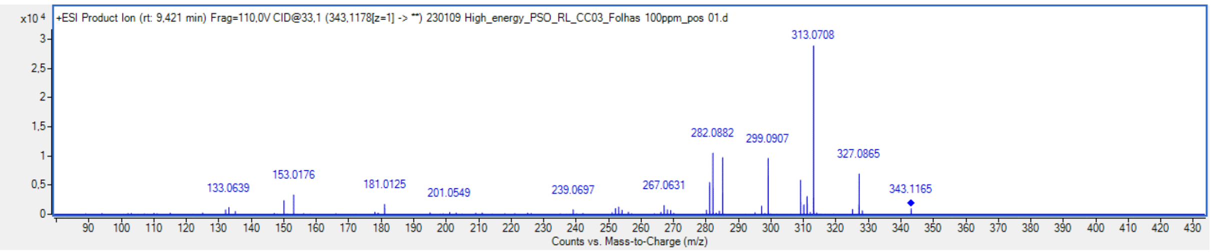

MS/MS spectrum from Tetra-*O*-methylscutellarein (**32**).

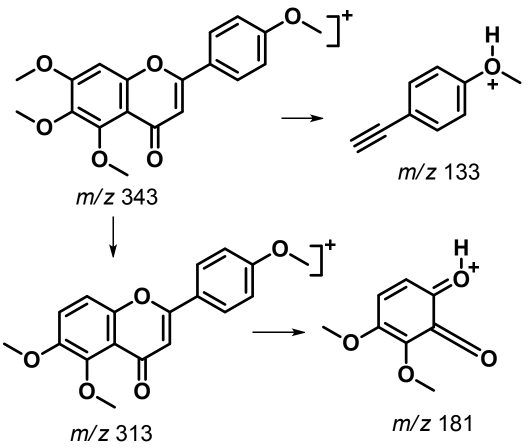

Scheme 32. MS fragmentation patterns for flavonoid Tetra-*O*-methylscutellarein (**32**) (The loss of a methoxyl could have occurred at any position).

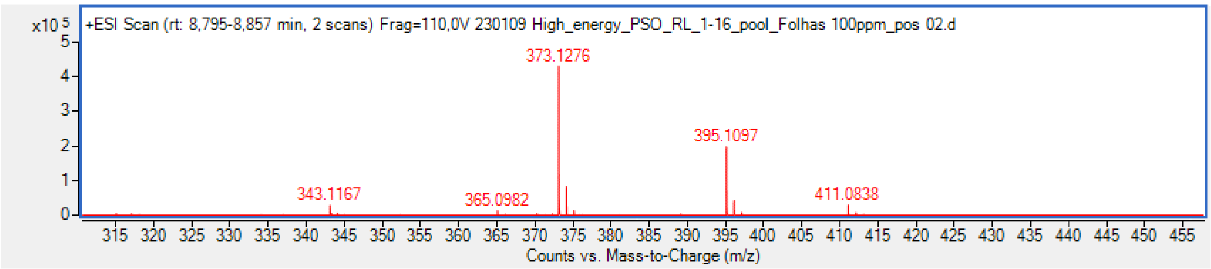

MS[1] spectrum from Sinensetin (**33**), $[M+H]^+$ *m/z* 373.1276, error: 1.6 ppm.

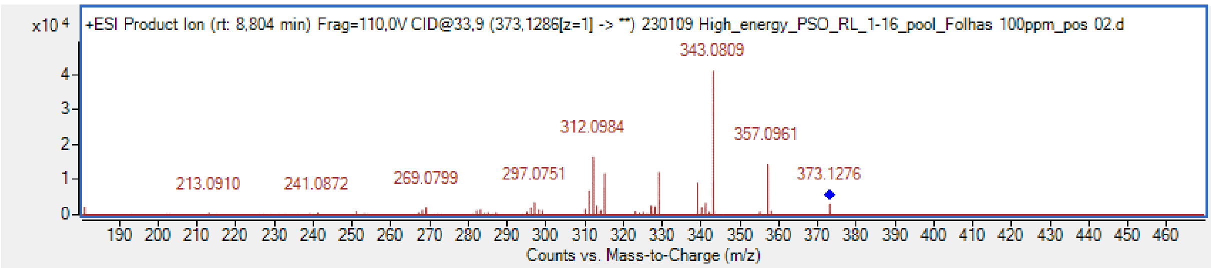

MS/MS spectrum from Sinensetin (**33**).

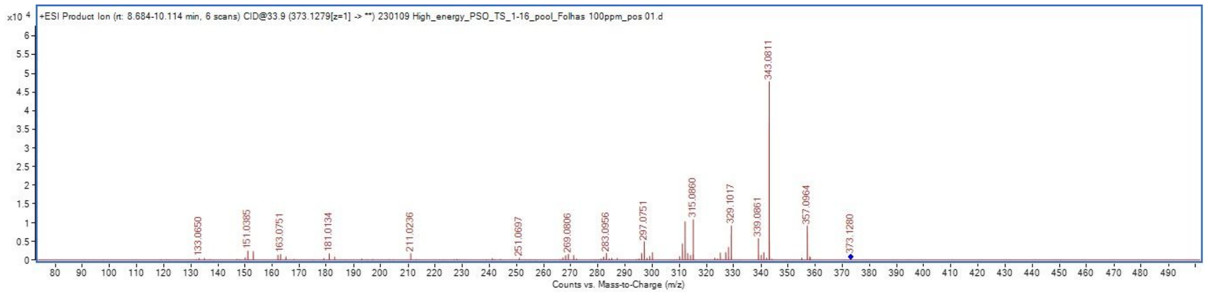

MS/MS spectrum from Sinensetin (**33**).

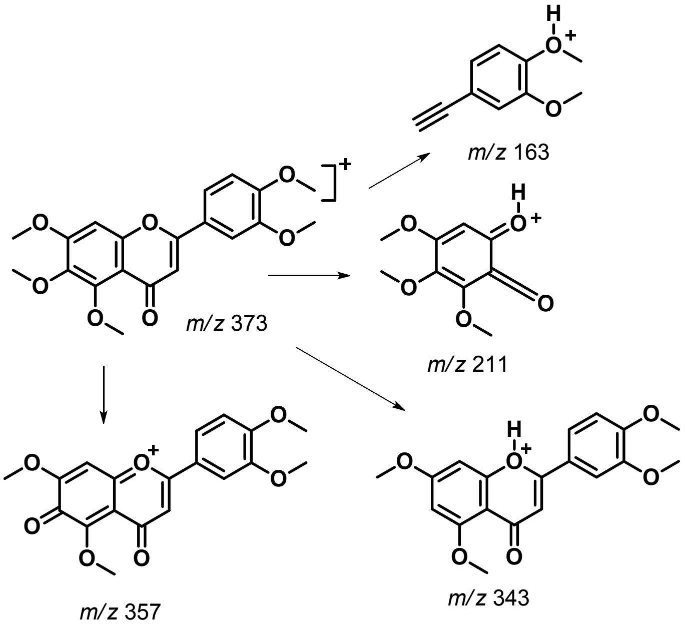

Scheme 33. MS fragmentation patterns for flavonoid Sinensetin (**33**).

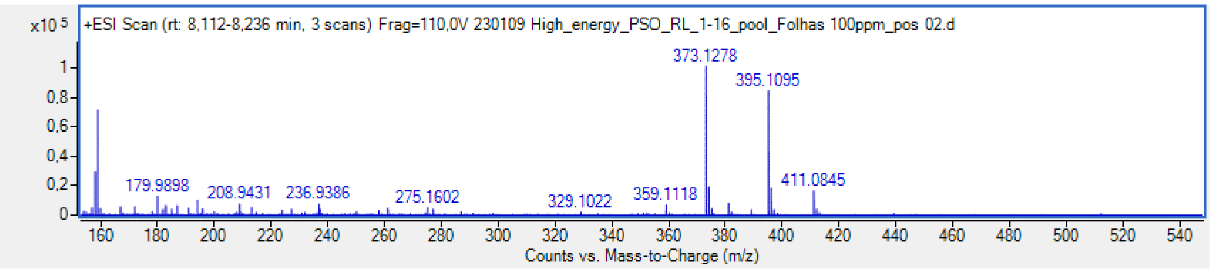

MS[1] spectrum from Isosinensetin (**34**), $[M+H]^+$ *m/z* 373.1278, error: 1.3 ppm.

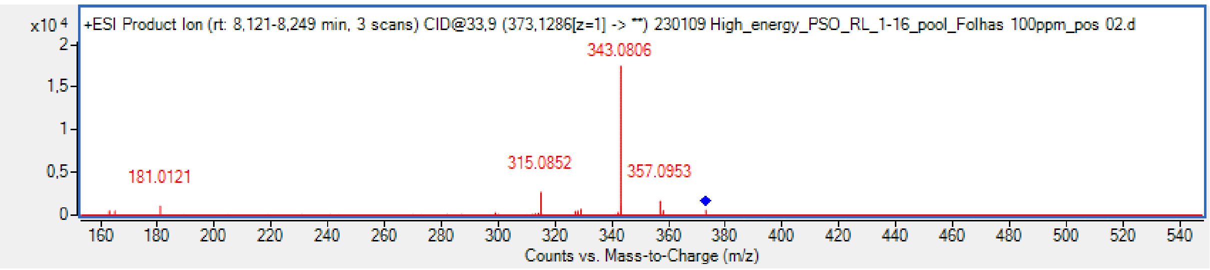

MS/MS spectrum from Isosinensetin (**34**).

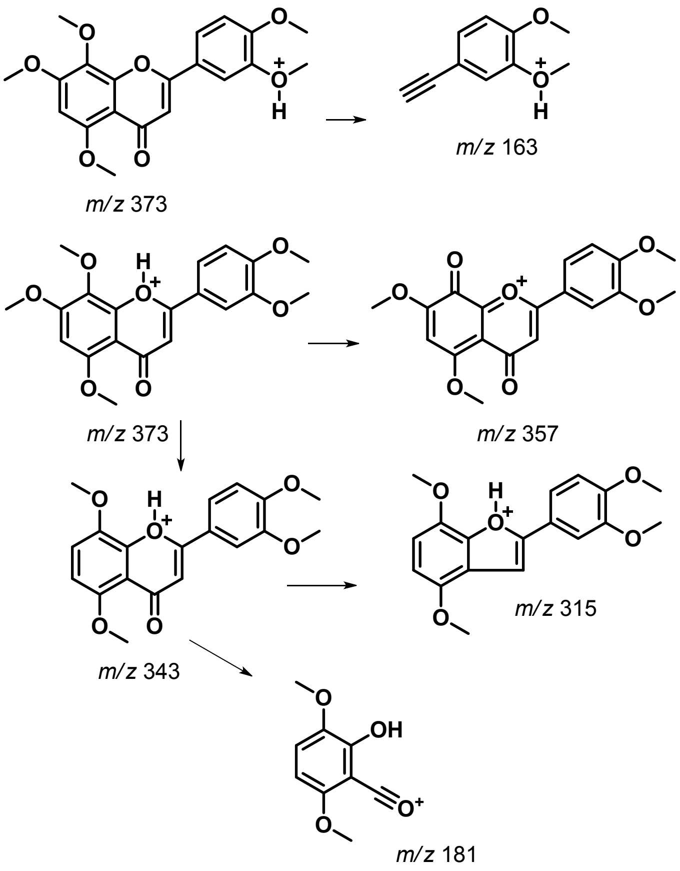

Scheme 34. MS fragmentation patterns for flavonoid Isosinensetin (**34**) (The loss of a methoxyl could have occurred at any position; the ion *m/z* 163 is present in the spectrum).

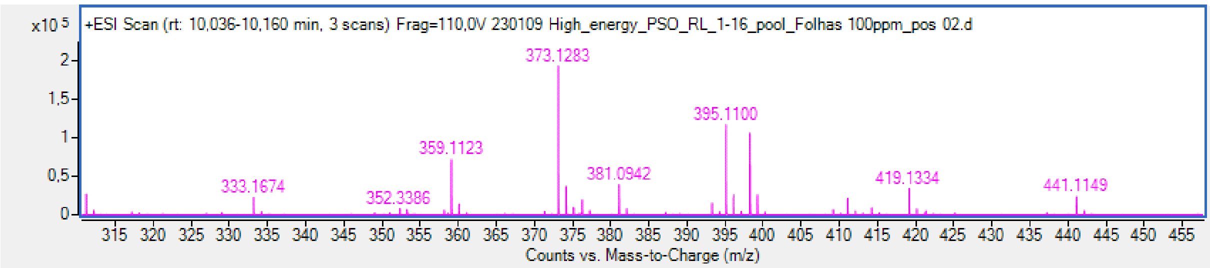

MS[1] spectrum from Tangeritin (**35**), $[M+H]^+$ *m/z* 373.1283, error: 0.3 ppm.

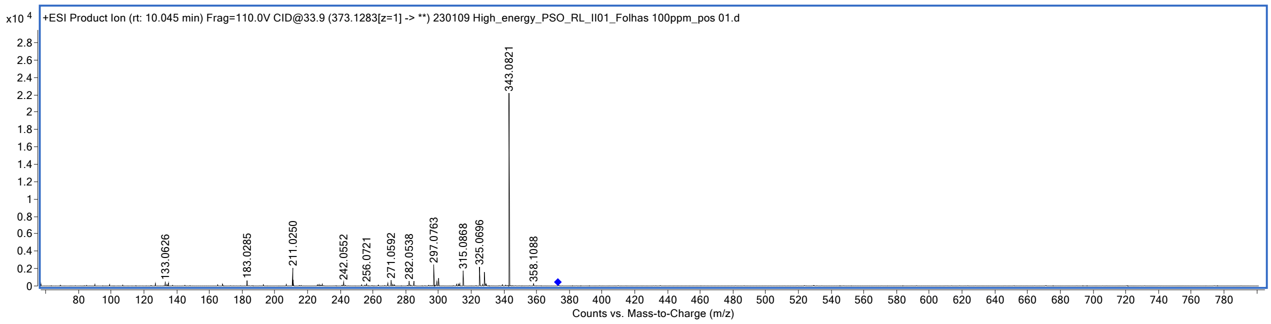

MS/MS spectrum from Tangeritin (**35**).

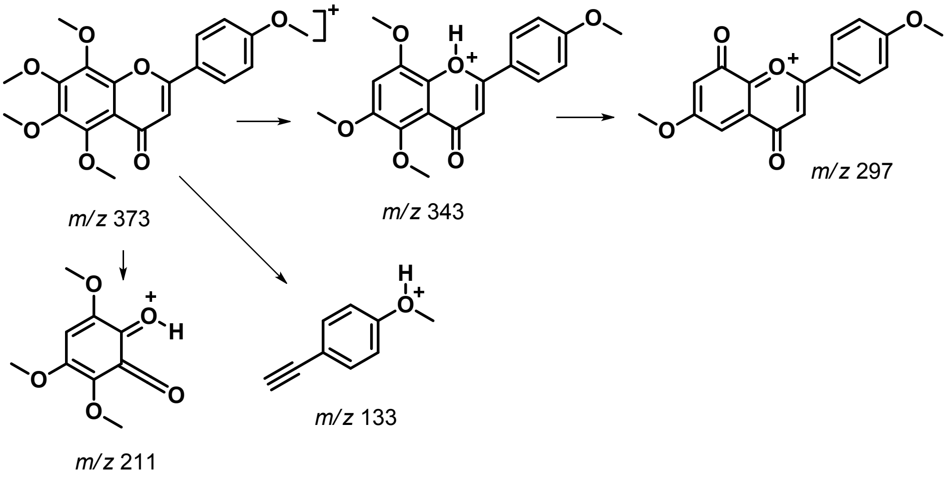

Scheme 35. MS fragmentation patterns for flavonoid Tangeretin (**35**).

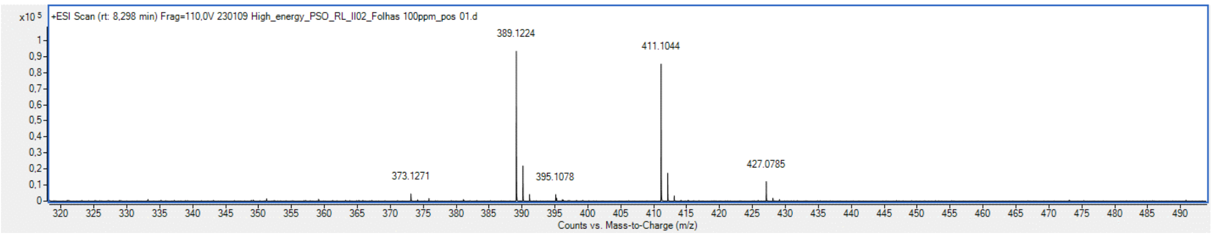

MS[1] spectrum from 3'-demethylnobiletin (**36**), $[M+H]^+$ *m/z* 389.1224, error: 1.8 ppm.

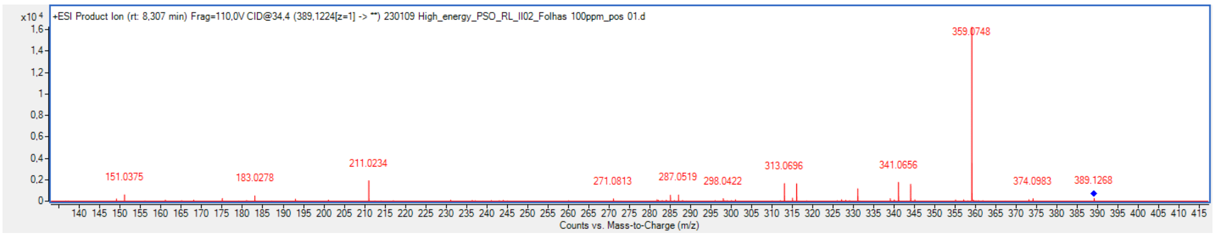

MS/MS spectrum from 3'-demethylnobiletin (**36**).

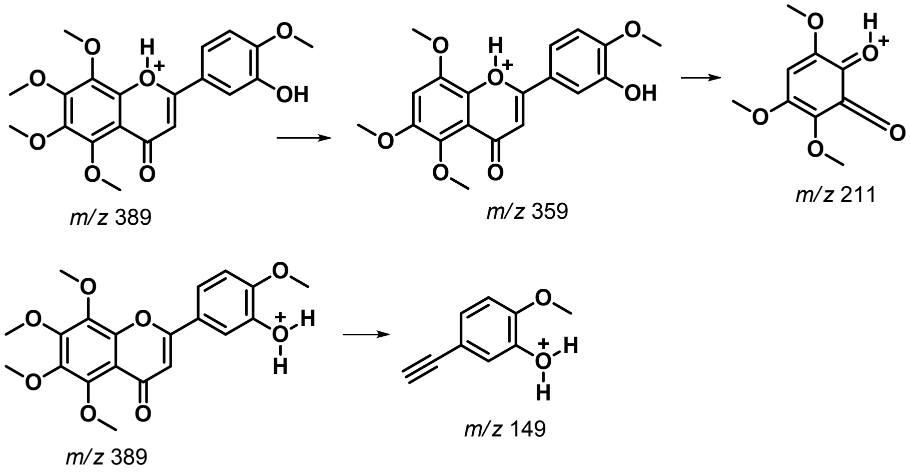

Scheme 36. MS fragmentation patterns for flavonoid 3’-Demethylnobiletin (**36**).

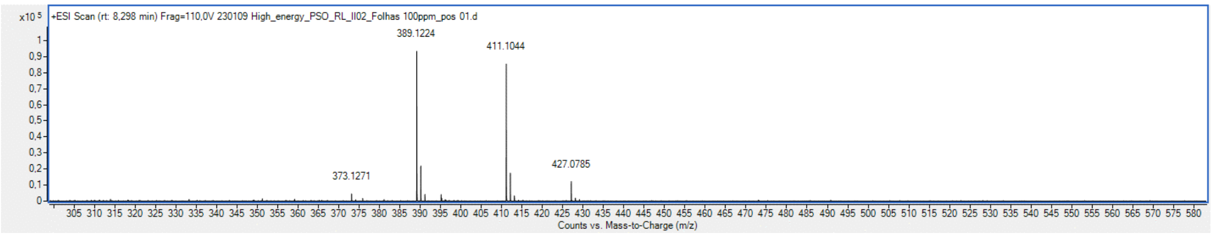

MS[1] spectrum from 5-*O*-demethylnobiletin (**37**), $[M+H]^+$ *m/z* 389.1236, error: 1.8 ppm.

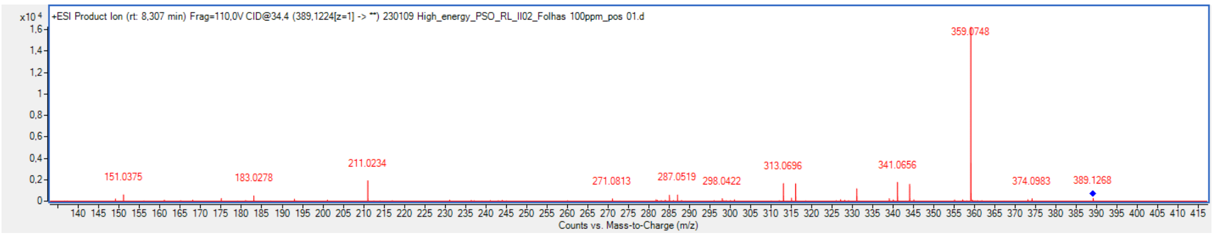

MS/MS spectrum from 5-*O*-demethylnobiletin (**37**).

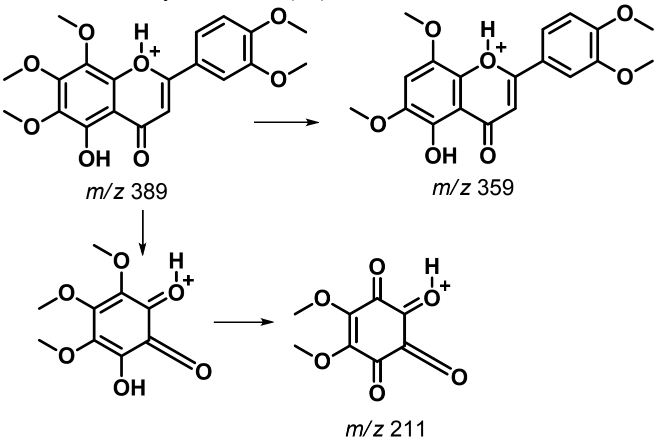

Scheme 37. MS fragmentation patterns for flavonoid 5-*O*-Demethylnobiletin (**37**); Fragment *m/z* 211 confirms the hydroxyl group on ring A.

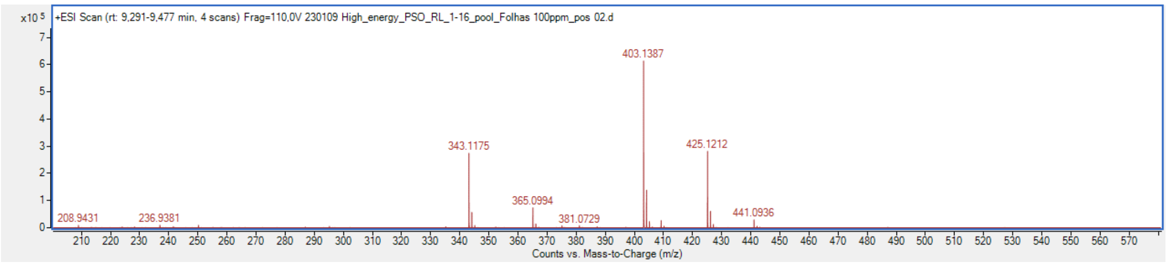

MS[1] spectrum from Nobiletin (**38**), $[M+H]^+$ *m/z* 403.1387, error: 0.0 ppm.

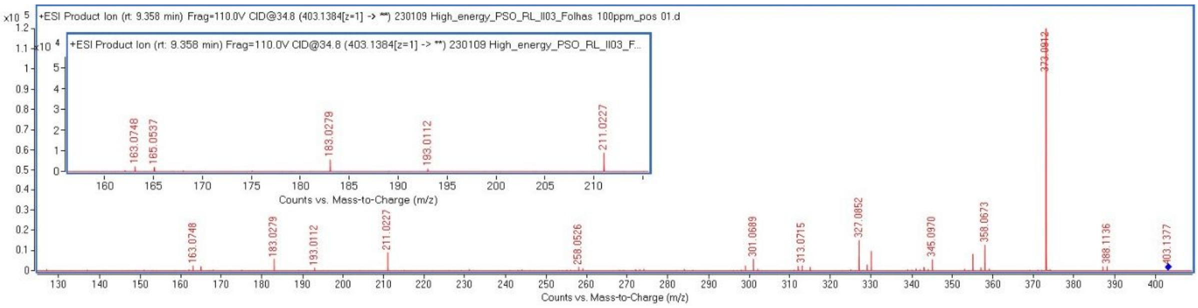

MS/MS spectrum from Nobiletin (**38**).

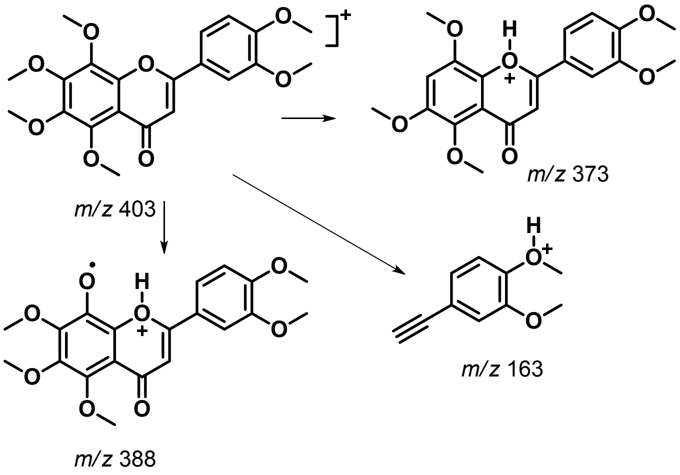

Scheme 38. MS fragmentation patterns for flavonoid Nobiletin (**38**).

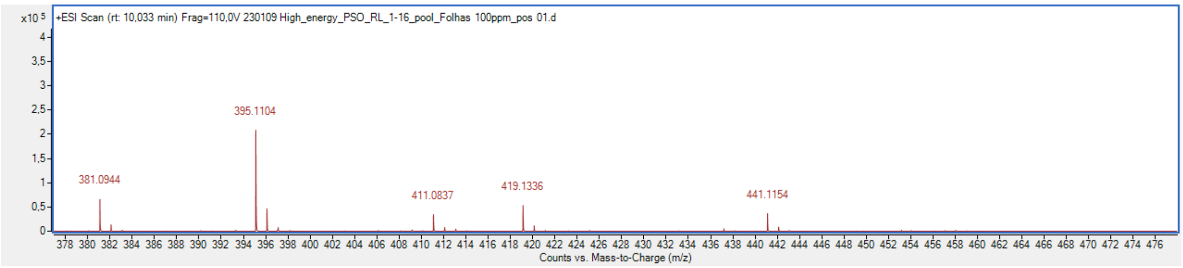

MS[1] spectrum from 5-hydroxy-3,6,7,8,3',4'-hexamethoxyflavone (**39**), $[M+H]^+$ *m/z* 419.1336, error: 0.2 ppm.

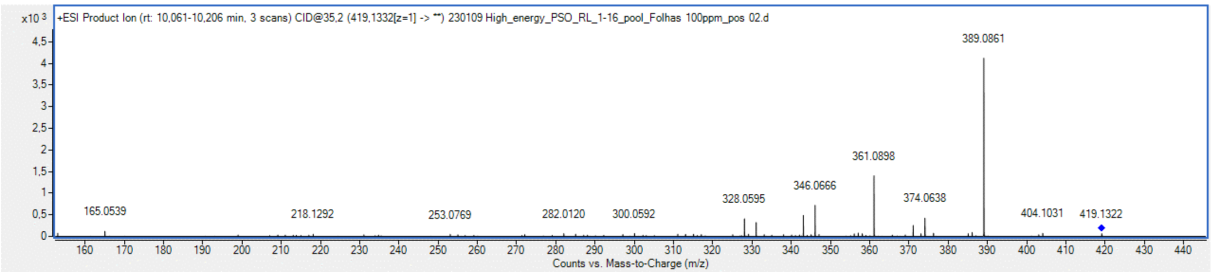

MS/MS spectrum from 5-hydroxy-3,6,7,8,3',4'-hexamethoxyflavone (**39**).

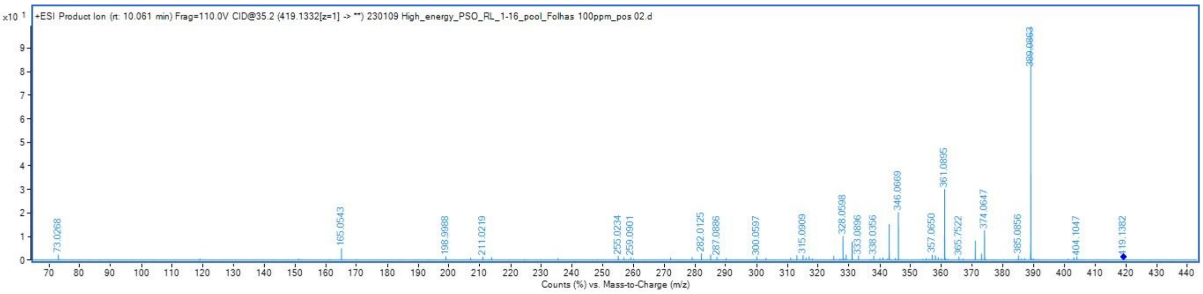

MS/MS spectrum from 5-hydroxy-3,6,7,8,3',4'-hexamethoxyflavone (**39**).

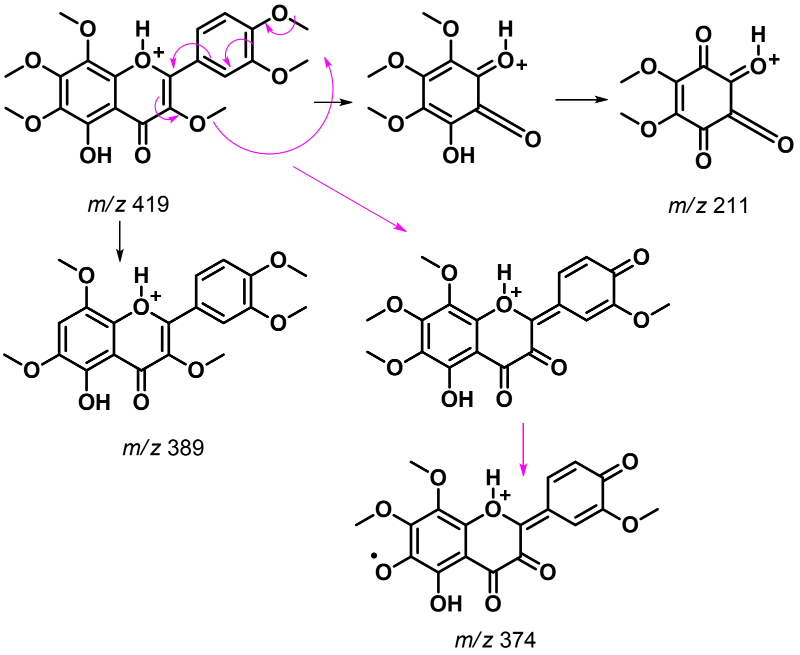

Scheme 39. MS fragmentation patterns for flavonoid 5-hydroxy-3,6,7,8,3',4'-hexamethoxyflavone (**39**), Fragment *m/z* 211 indicates the hydroxyl on ring A, and *m/z* 374 confirms having a methoxy group at C-3.

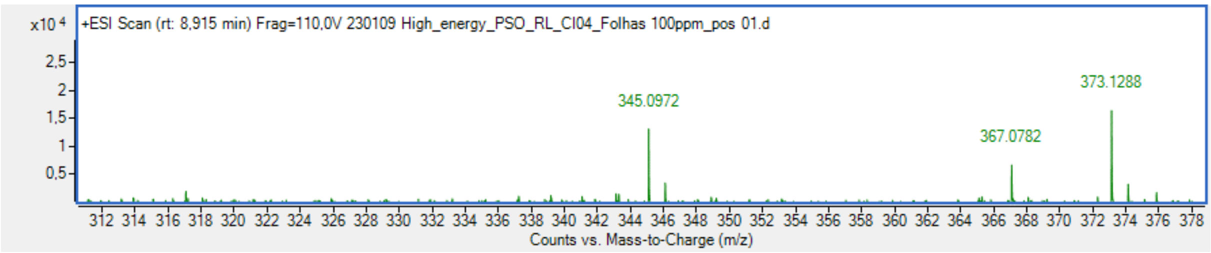

MS[1] spectrum from Eupatorin (**40**), $[M+H]^+$ *m/z* 345.0972, error: 0.9 ppm.

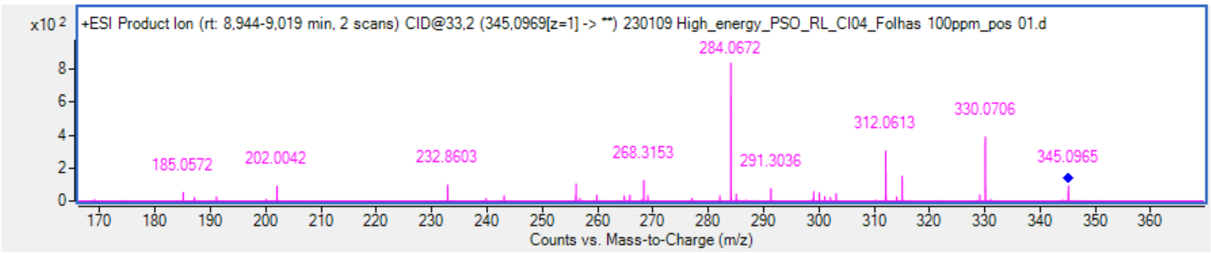

MS/MS spectrum from Eupatorin (**40**).

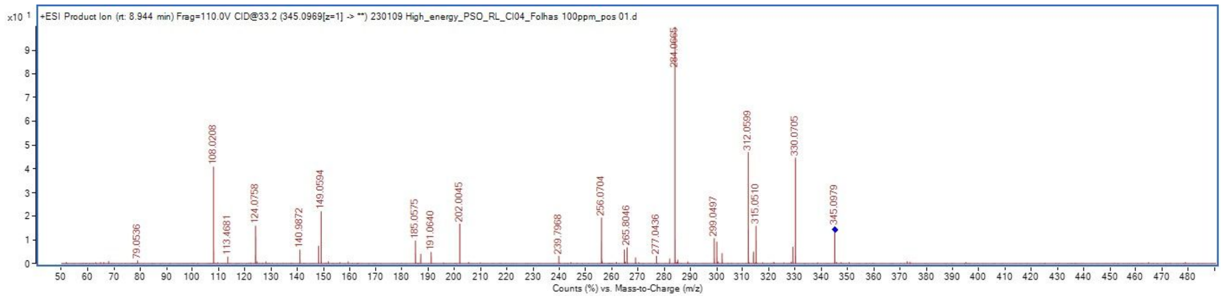

MS/MS spectrum from Eupatorin (**40**).

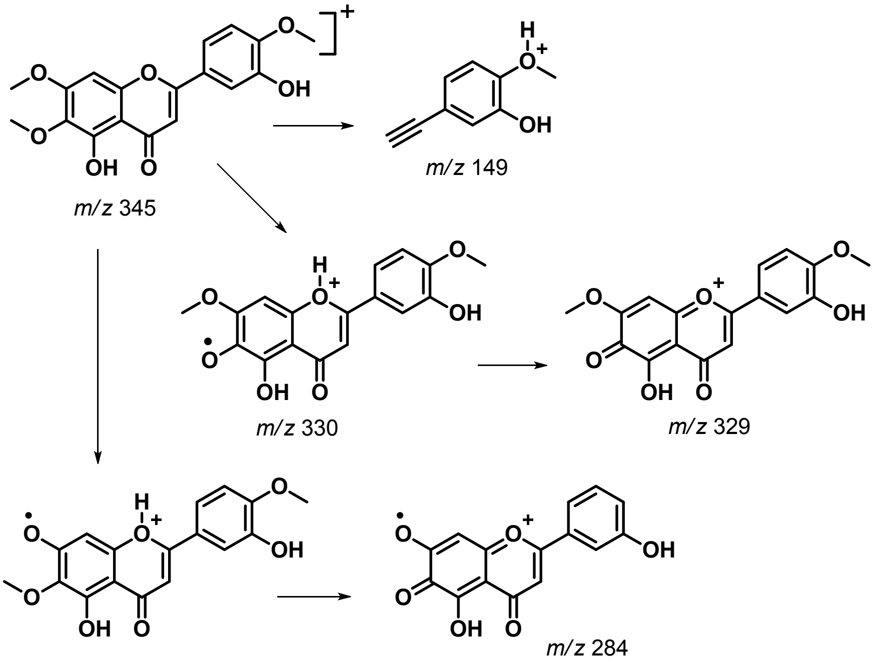

Scheme 40. MS fragmentation patterns for flavonoid Eupatorin (**40**), The fragment *m/z* 149 indicates a hydroxyl in ring B, consequently the second would be in ring A, confirmed in the other fragments.

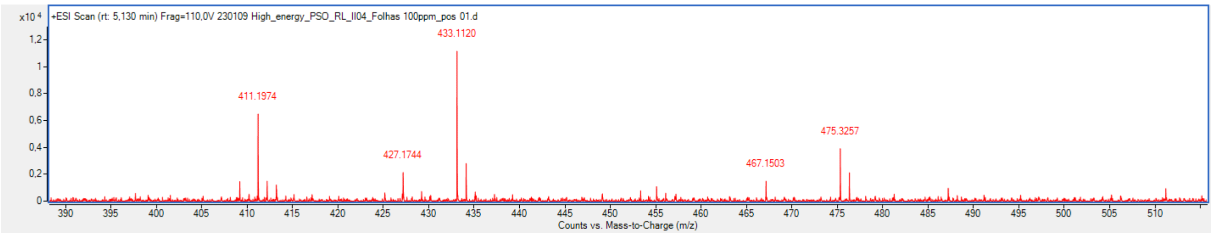

MS[1] spectrum from Vitexin (**41**), $[M+H]^+$ *m/z* 433.1120, error: 2.1 ppm.

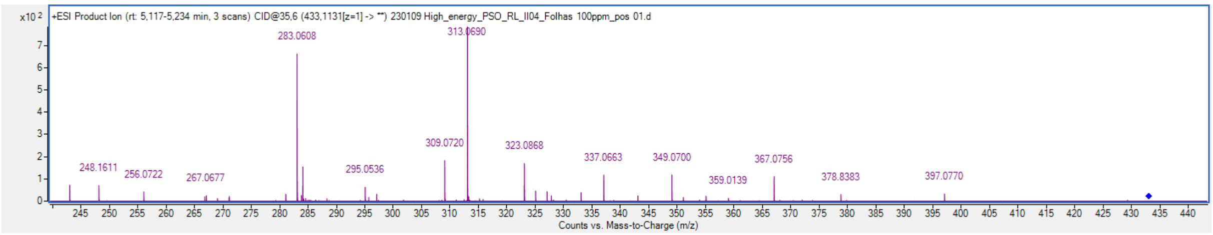

MS/MS spectrum from Vitexin (**41**).

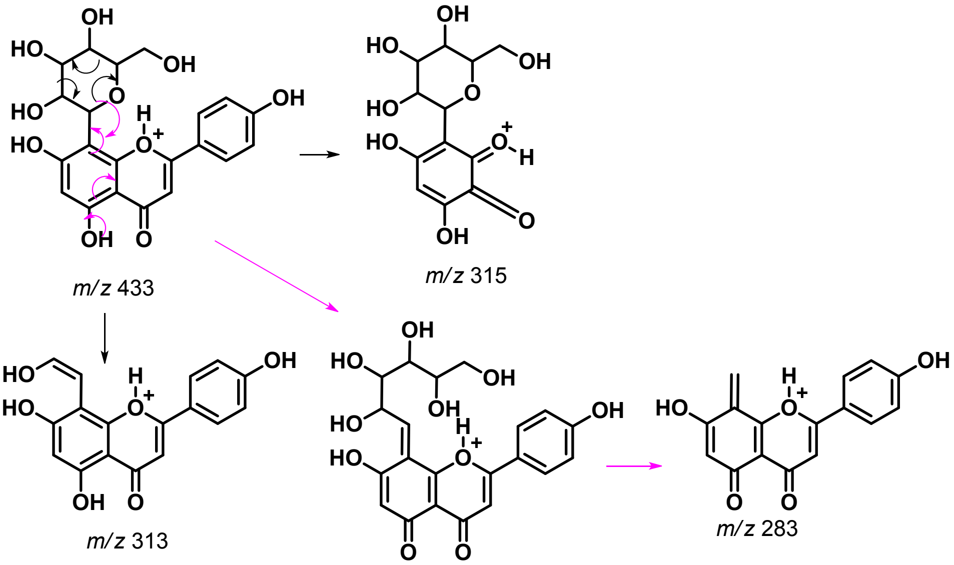

Scheme 41. MS fragmentation patterns for flavonoid Vitexin (**41**).

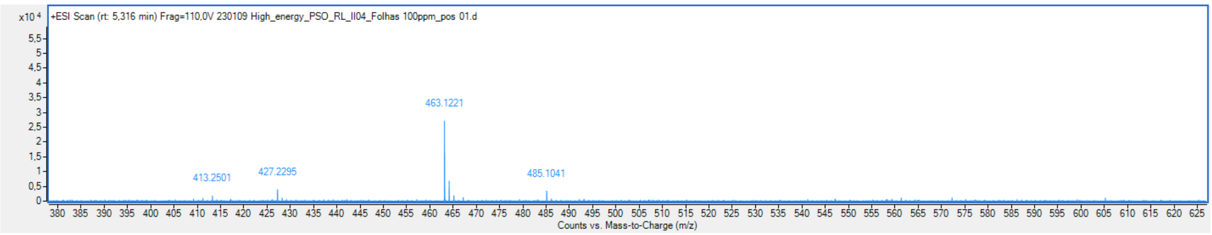

MS[1] spectrum from Swertiajaponin (**42**), $[M+H]^+$ *m/z* 463.1221, error: 3.0 ppm.

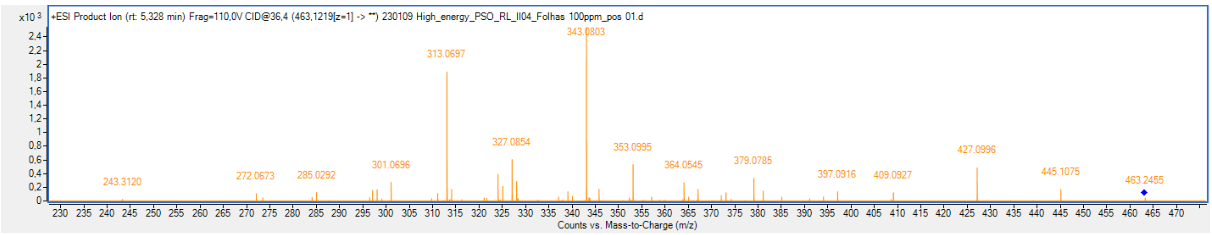

MS/MS spectrum from Swertiajaponin (**42**).

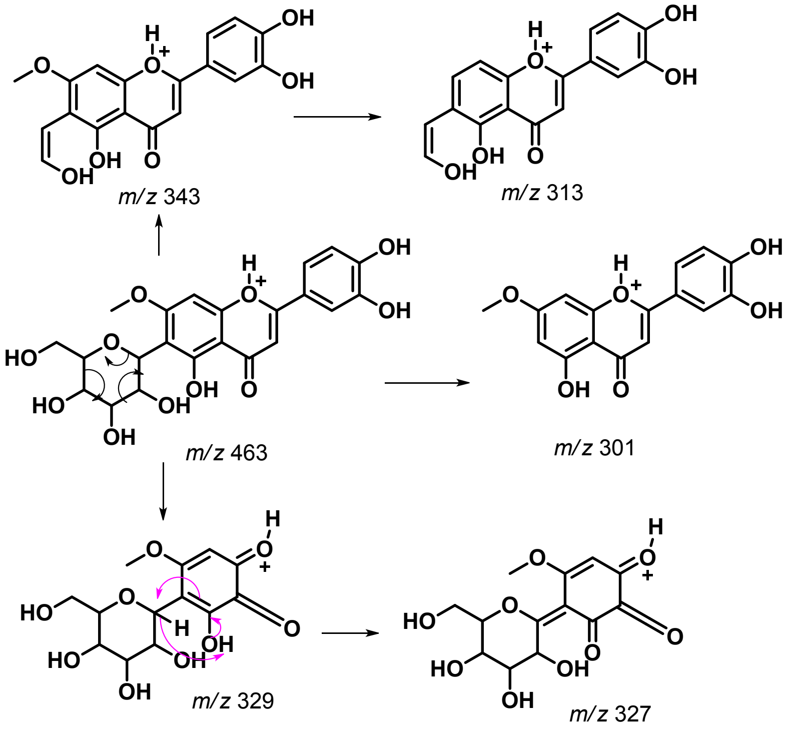

Scheme 42. MS fragmentation patterns for flavonoid Swertiajaponin (**42**).

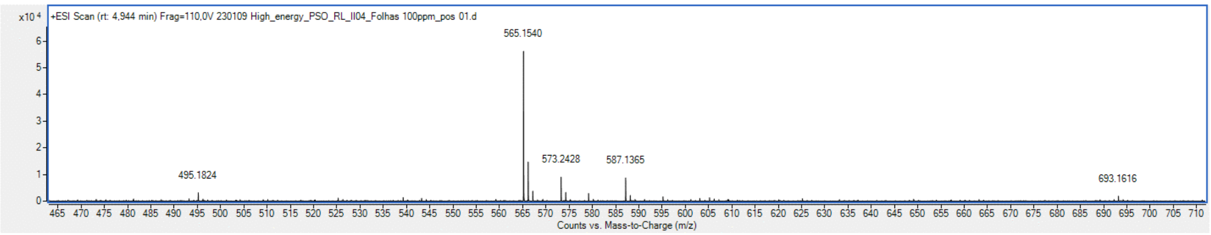

MS[1] spectrum from Isovitexin 2"-*O*-arabinoside (**43**), $[M+H]^+$ *m/z* 565.1540, error: 2.1 ppm.

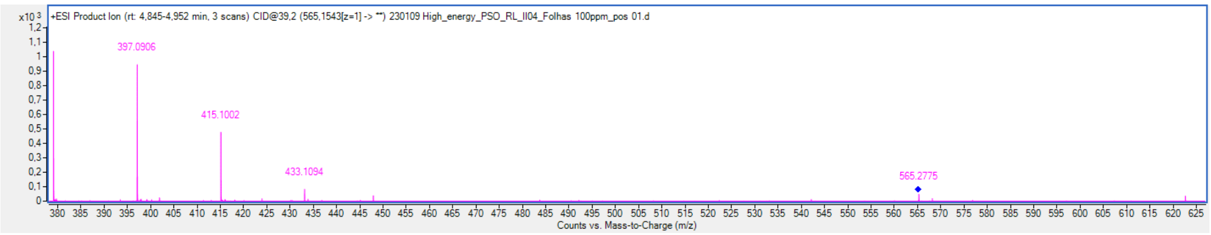

MS/MS spectrum from Isovitexin 2"-*O*-arabinoside (**43**).

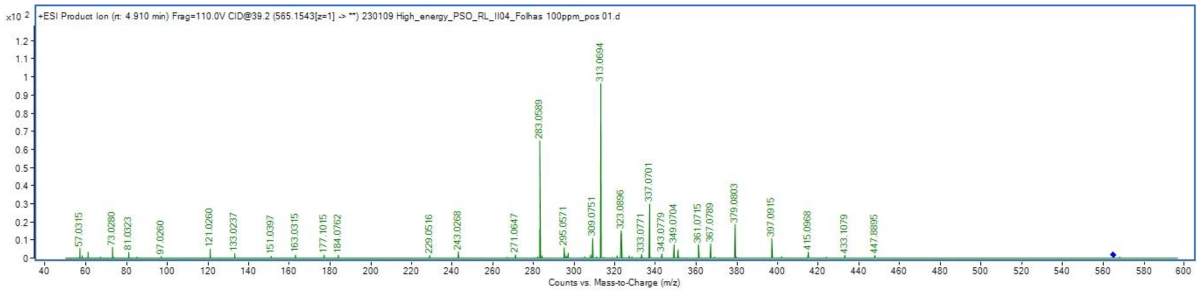

MS/MS spectrum from Isovitexin 2''-*O*-arabinoside (**43**).

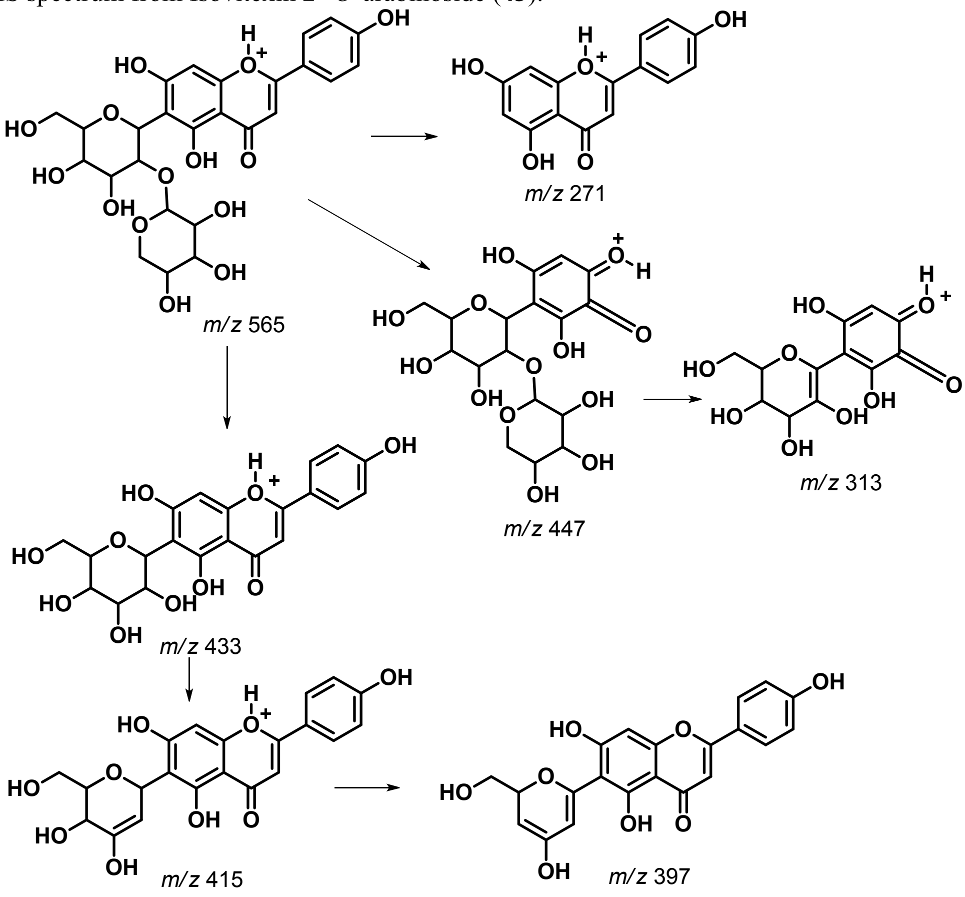

Scheme 43. MS fragmentation patterns for flavonoid Isovitexin 2''-*O*-arabinoside (**43**).

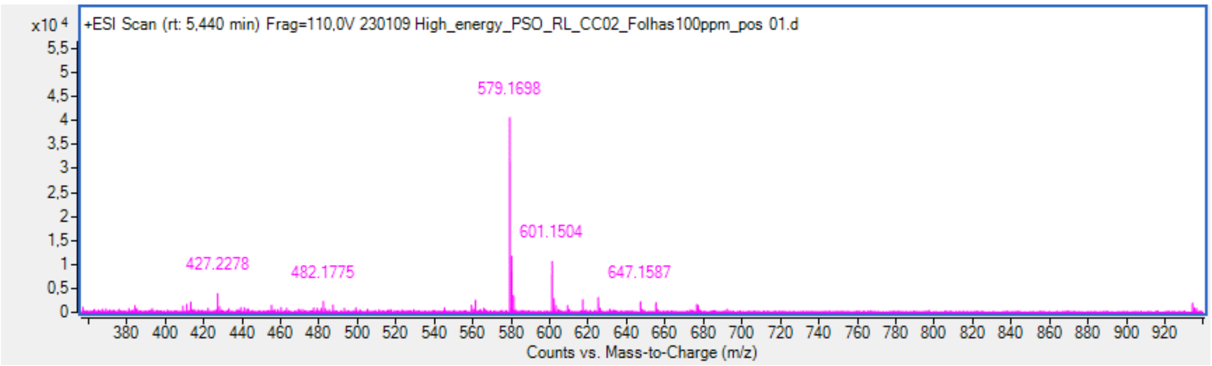

MS[1] spectrum from Rhoifolin (**44**), $[M+H]^+$ *m/z* 579.1694, error: 2.4 ppm.

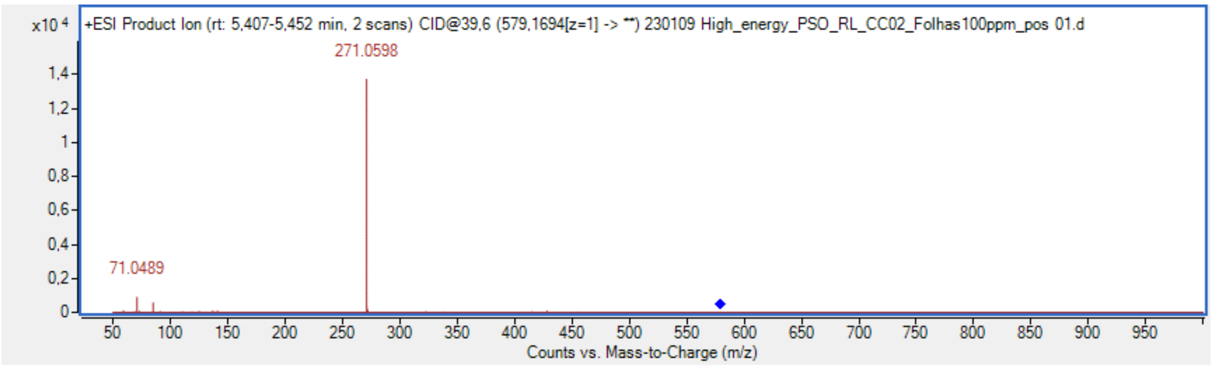

MS/MS spectrum from Rhoifolin (**44**).

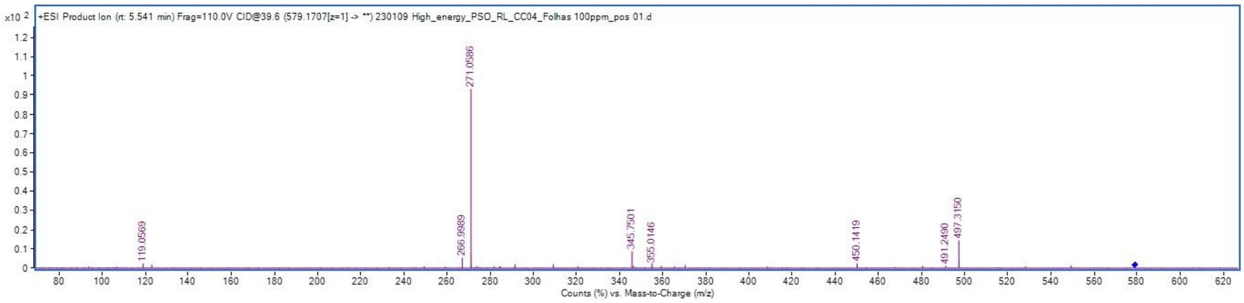

MS/MS spectrum from Rhoifolin (**44**).

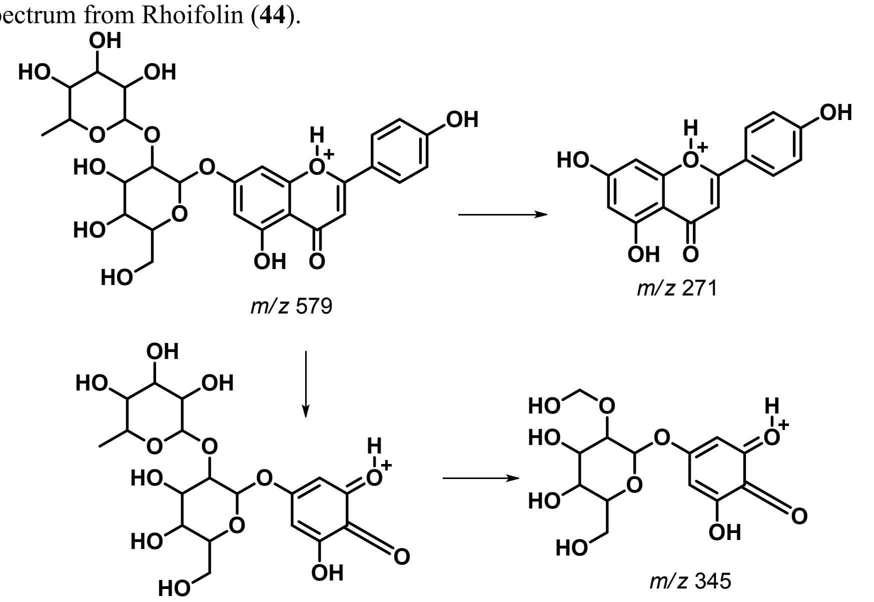

Scheme 44. MS fragmentation patterns for flavonoid Rhoifolin (**44**); The fragment *m/z* 345 indicates the glycosyl unit in ring A.

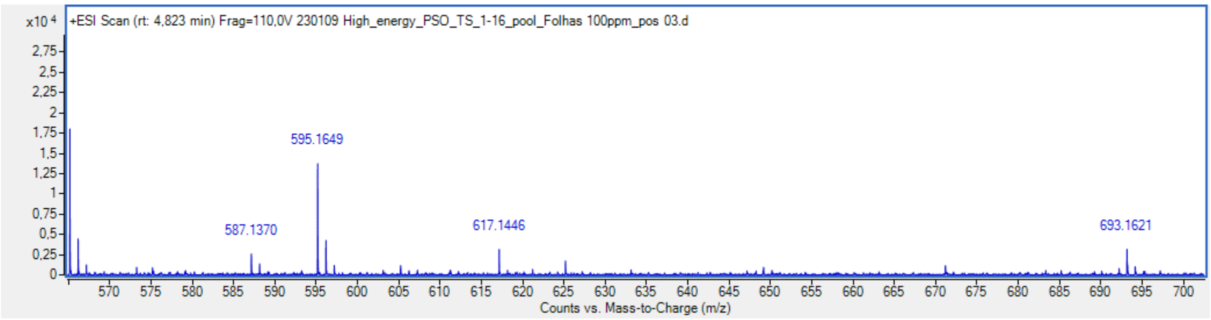

MS[1] spectrum from Saponarin (**45**), $[M+H]^+$ *m/z* 595.1649, error: 1.3 ppm.

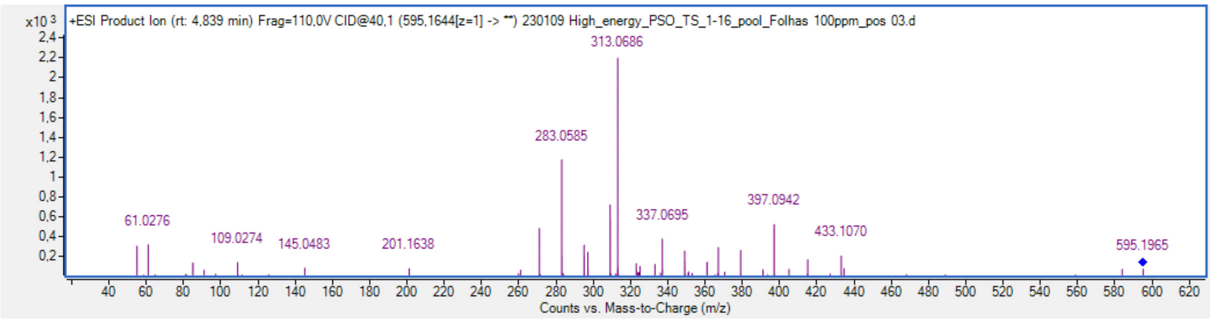

MS/MS spectrum from Saponarin (**45**).

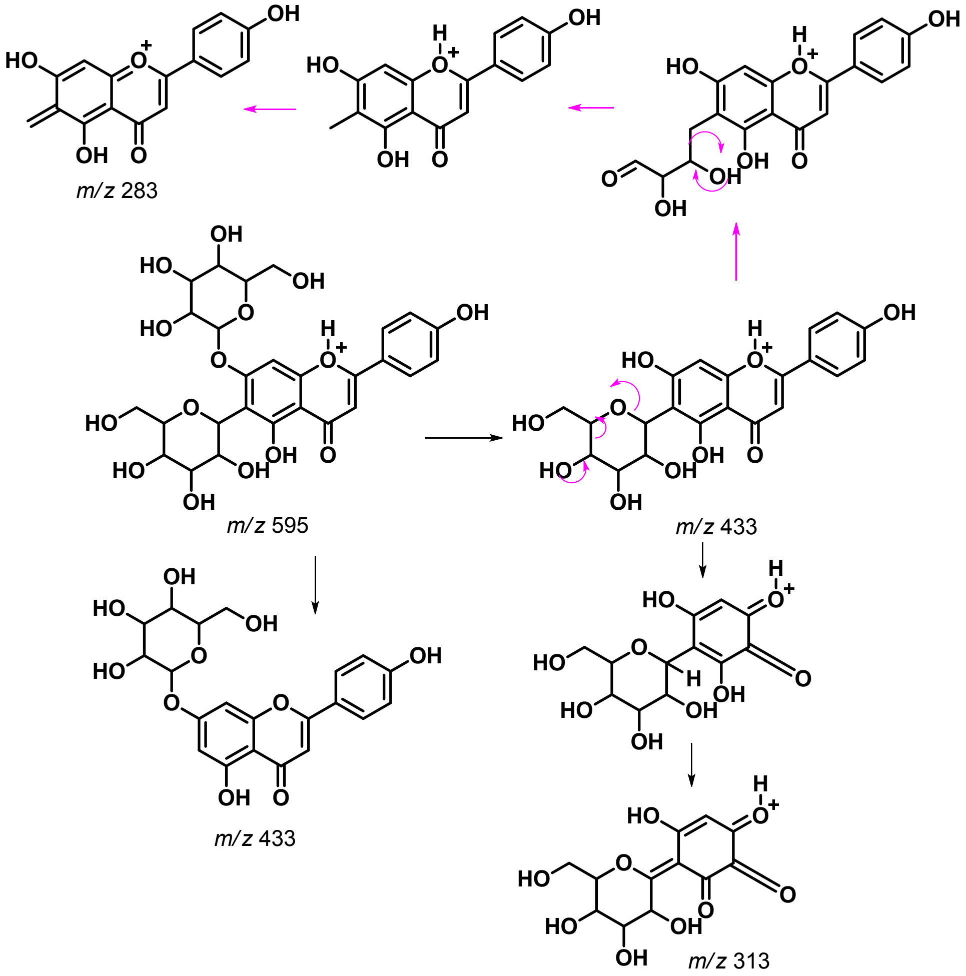

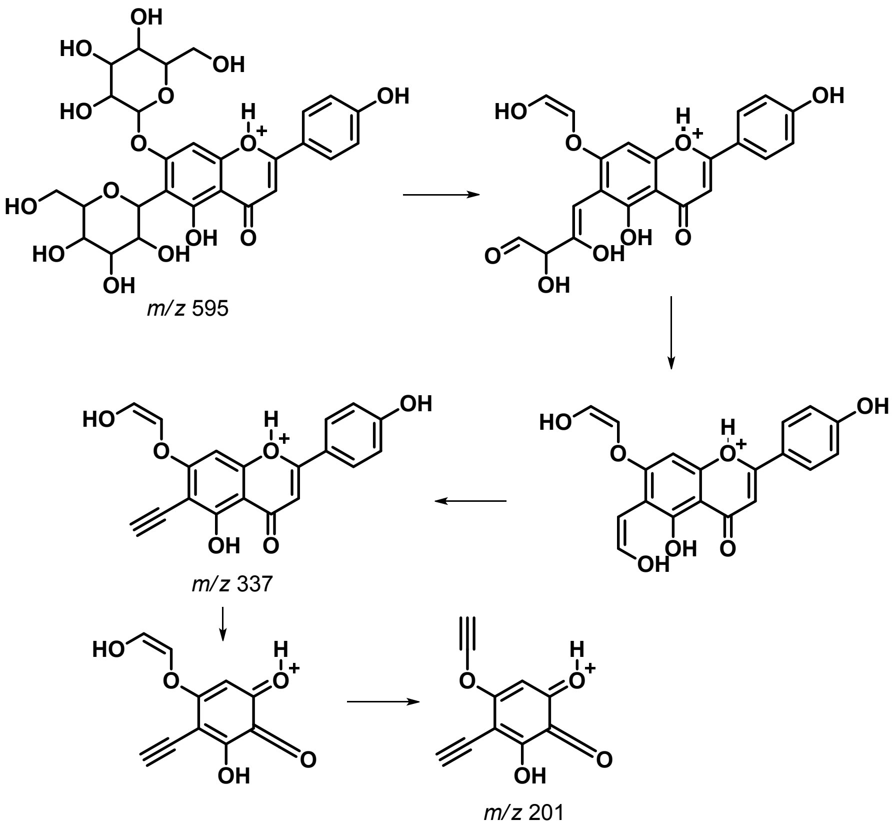

Scheme 45. MS fragmentation patterns for flavonoid Saponarin (**45**); m/z 201 indicates that both glycosyl units are in the A ring.

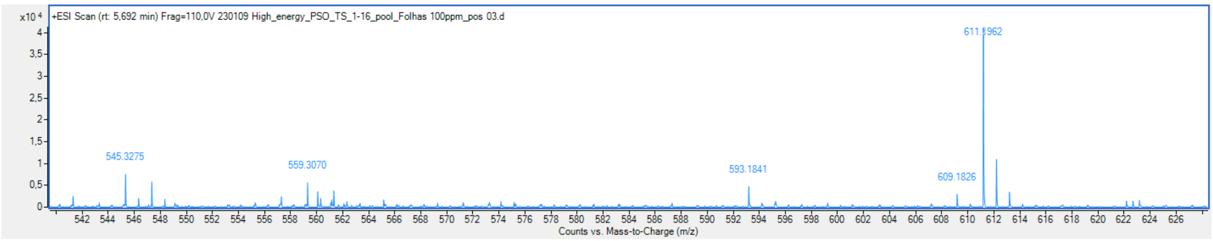

MS[1] spectrum from Hesperidin (**46**), $[M+H]^+$ *m/z* 611.1962, error: 1.3 ppm.

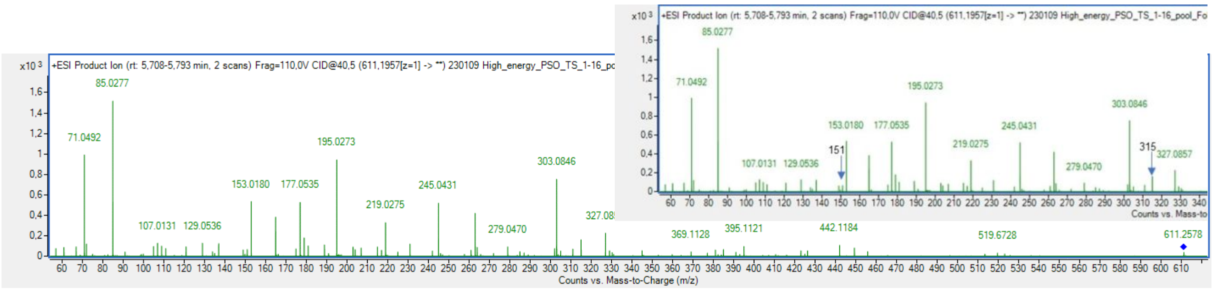

MS/MS spectrum from Hesperidin (**46**).

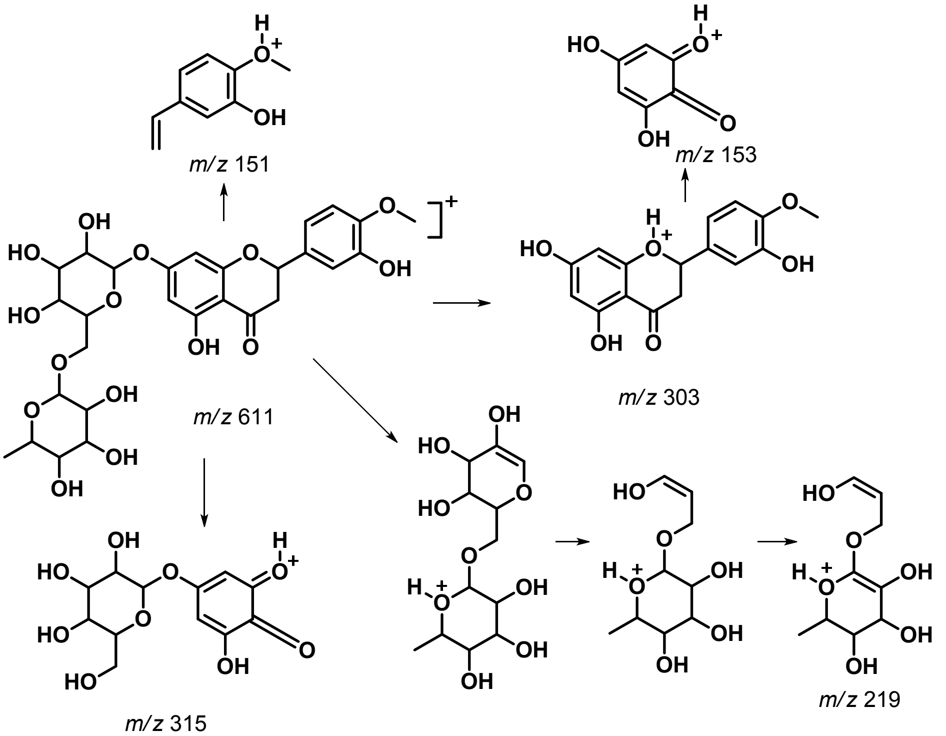

Scheme 46. MS fragmentation patterns for flavonoid Hesperidin (**46**); *m/z* 315 indicates that 6-*O*-(alpha-L-rhamnopyranosyl)-beta-D-glucopyranosyl moiety is in the A ring, and the *m/z* 219 indicates that this is a di-glucosyl and not two glycosides.

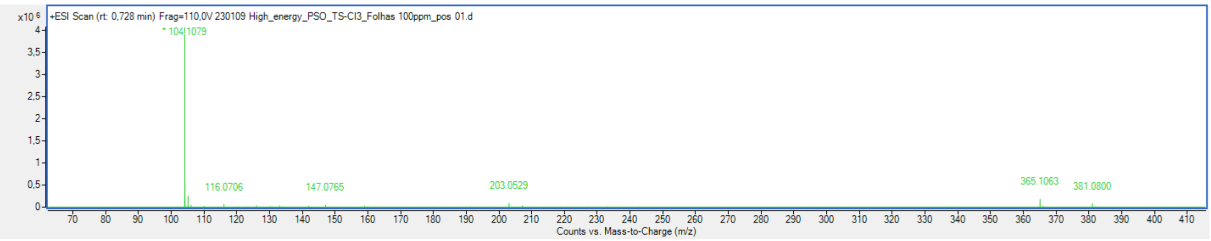

MS[1] spectrum from L-aspartic acid (**47**), $[M+H]^+$ *m/z* 134.0453, error: 3.7 ppm.

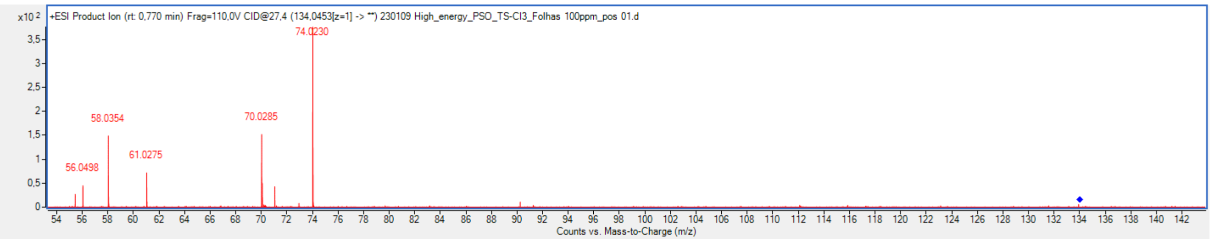

MS/MS spectrum L-aspartic acid (**47**).

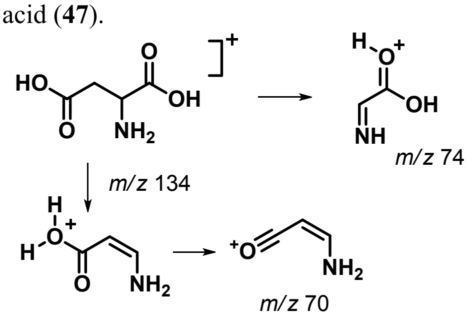

Scheme 47. MS fragmentation patterns for AA L-aspartic acid (**47**).

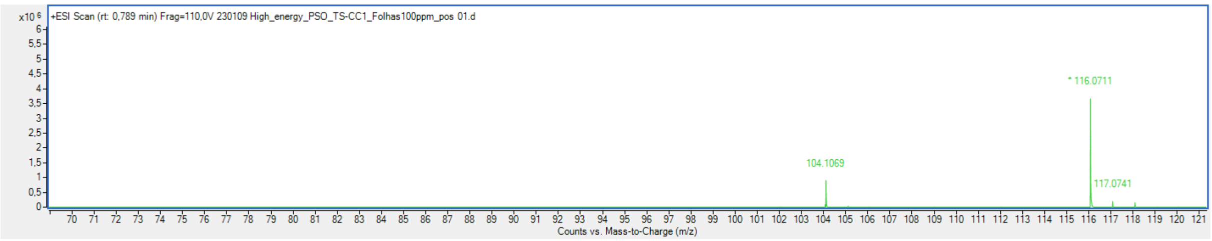

MS[1] spectrum from Choline (**48**), $[M+H]^+$ *m/z* 104.1069, error: 1.0 ppm.

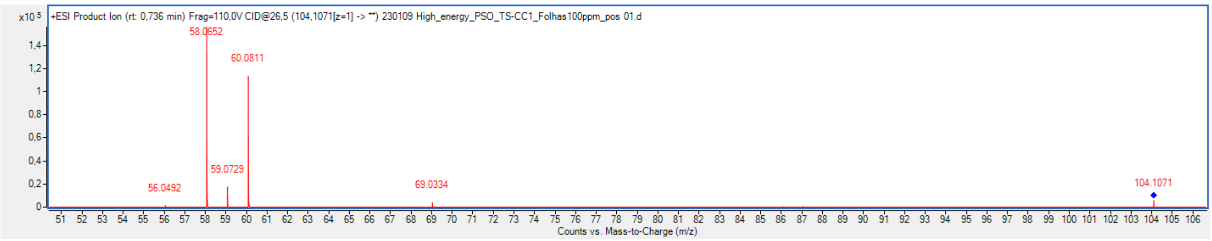

MS/MS spectrum from Choline (**48**).

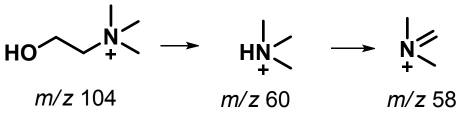

Scheme 48. MS fragmentation patterns for Choline (**48**).

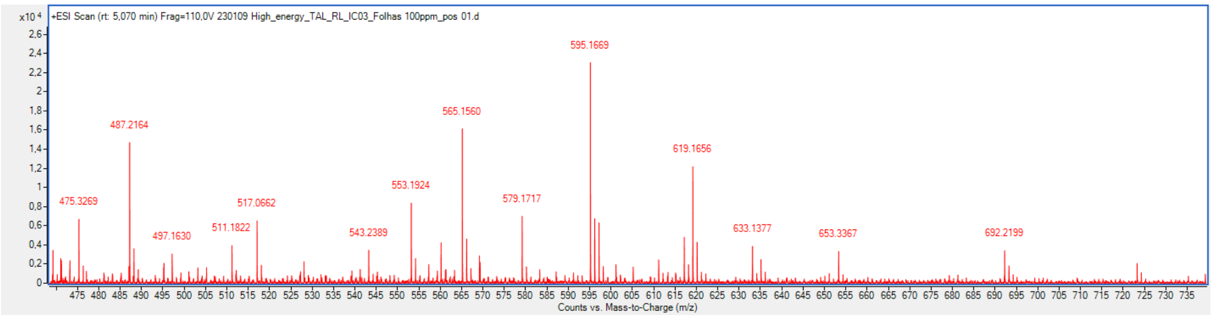

MS[1] spectrum from Luteolin-7-*O*-rutinoside (**49**), $[M+H]^+$ *m/z* 595.1669, error: 2.0 ppm.

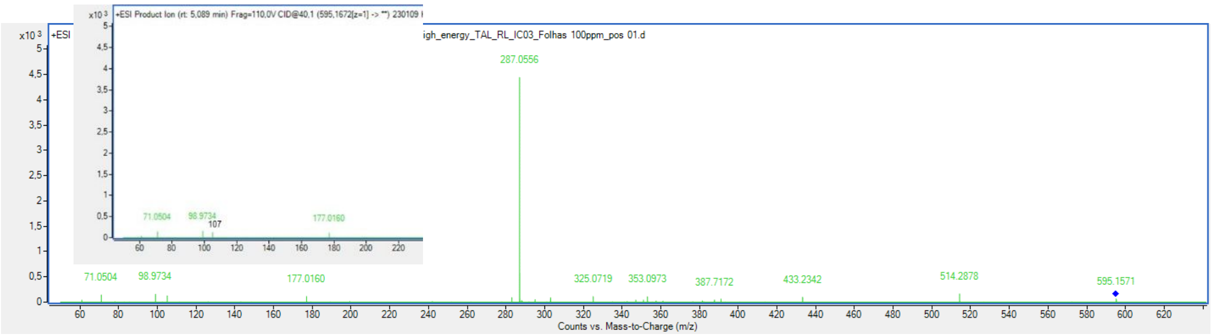

MS/MS spectrum from Luteolin-7-*O*-rutinoside (**49**).

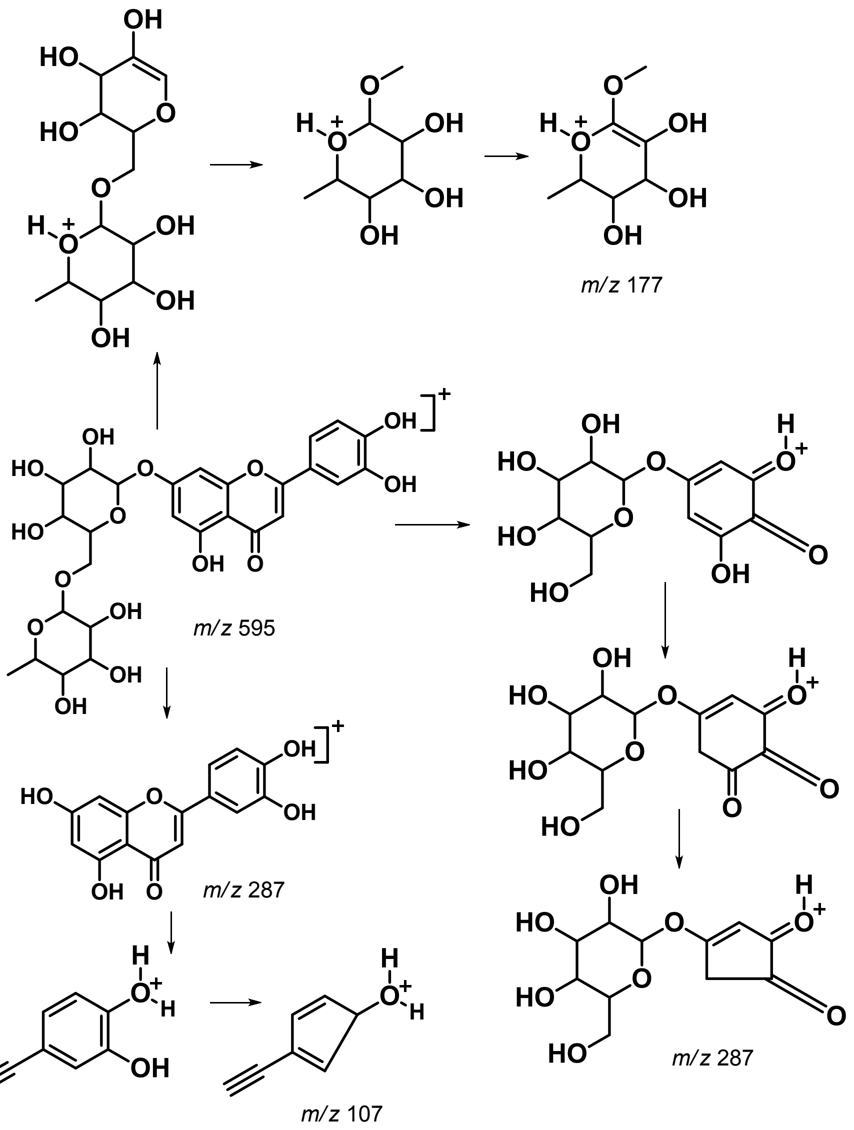

Scheme 49. MS fragmentation patterns for flavonoid Luteolin-7-*O*-rutinoside (**49**).

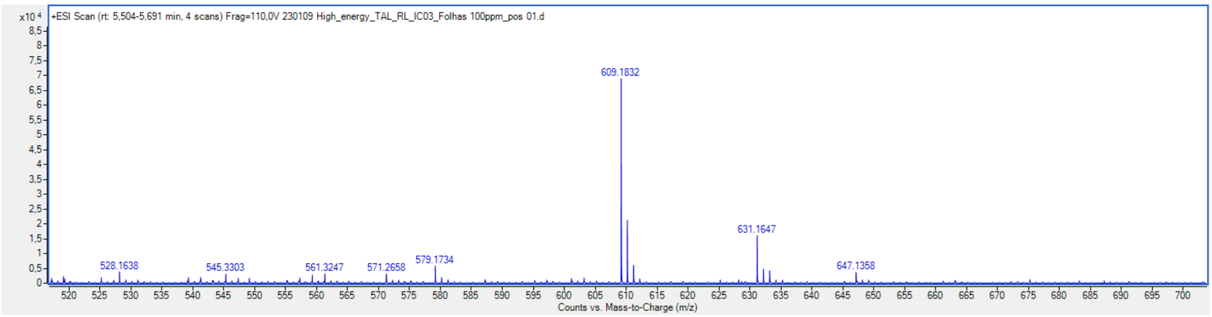

MS[1] spectrum from Kaempferide 3-*O*-β-D-glucopyranoside 7-*O*-α-L-rhamnopyranoside (**50**), $[M+H]^+$ *m/z* 609.1832, error: 3.0 ppm.

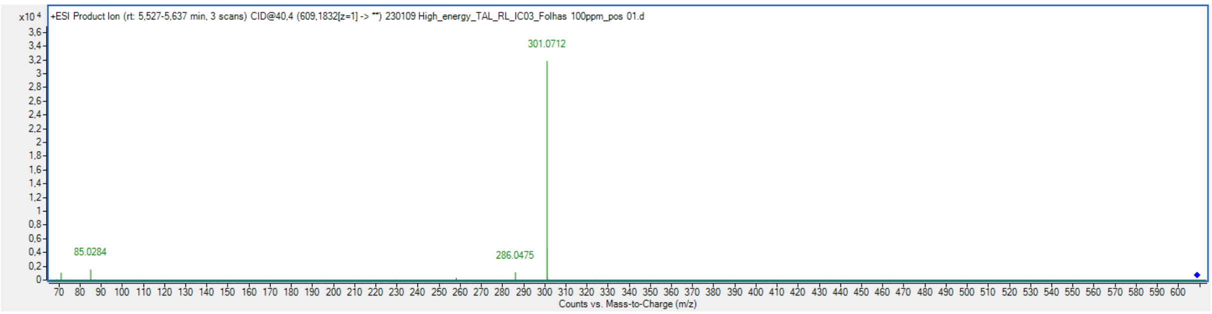

MS/MS spectrum from Kaempferide 3-*O*-β-D-glucopyranoside 7-*O*-α-L-rhamnopyranoside (**50**).

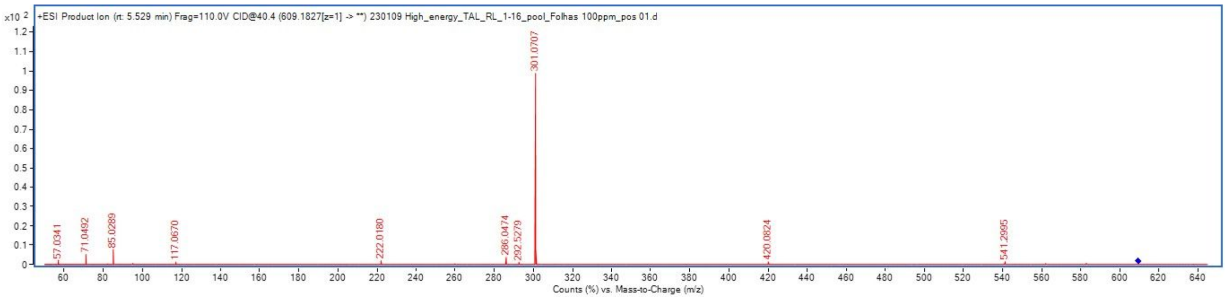

MS/MS spectrum from Kaempferide 3-*O*-β-D-glucopyranoside 7-*O*-α-L-rhamnopyranoside (**50**).

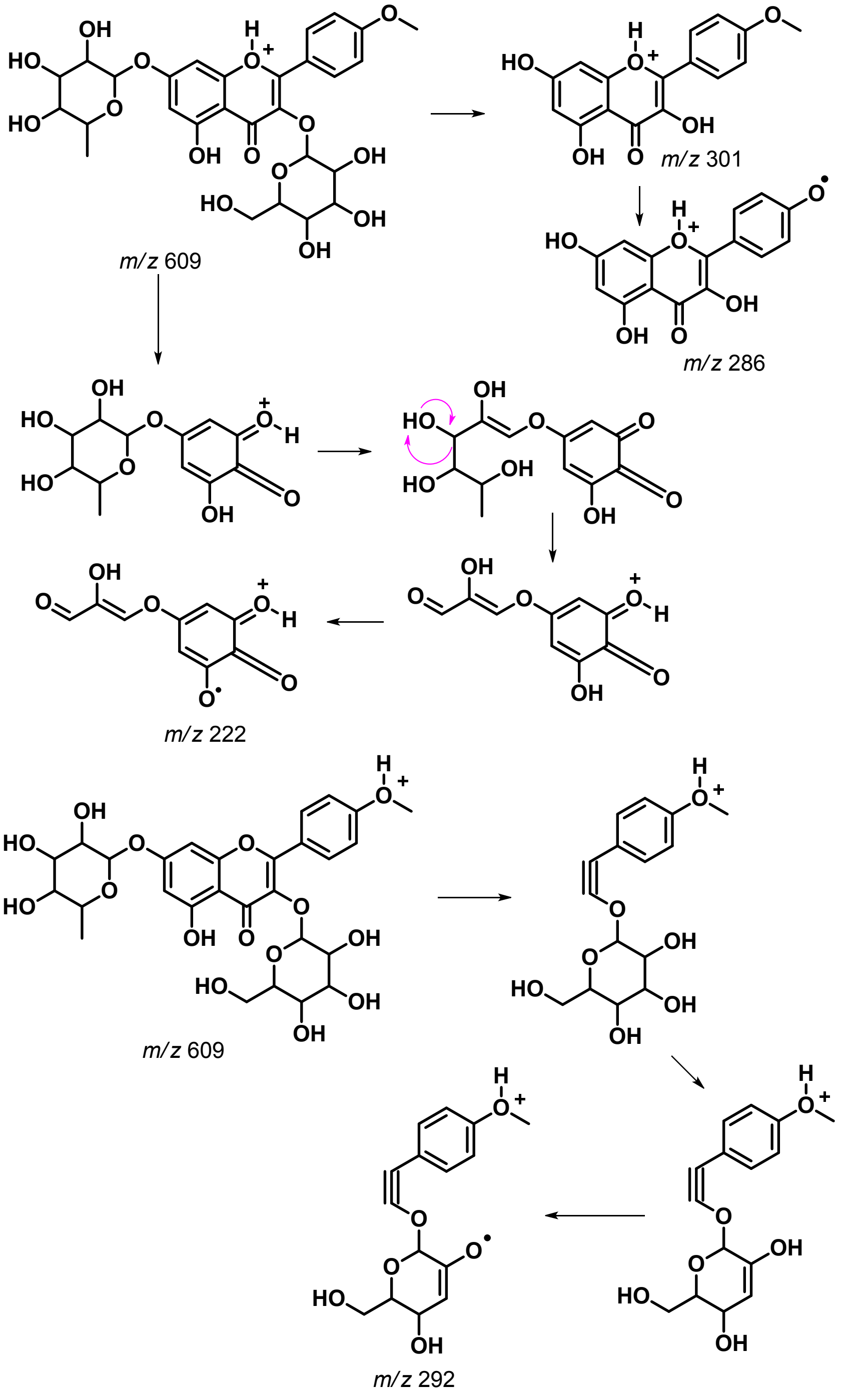

Scheme 50. MS fragmentation patterns for flavonoid Kaempferide 3-*O*-β-D-glucopyranoside 7-*O*-α-L-rhamnopyranoside (**50**).

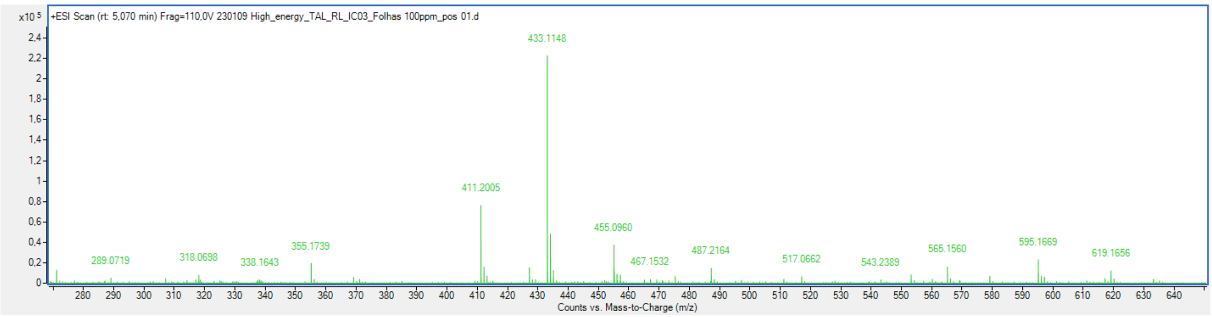

MS[1] spectrum from Isovitexin (**51**), $[M+H]^+$ *m/z* 433.1148, error: 3.98 ppm.

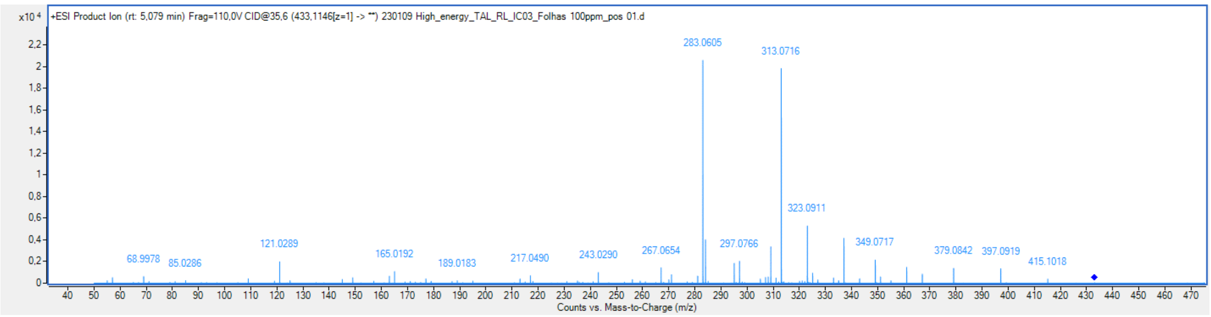

MS/MS spectrum from Isovitexin (**51**).

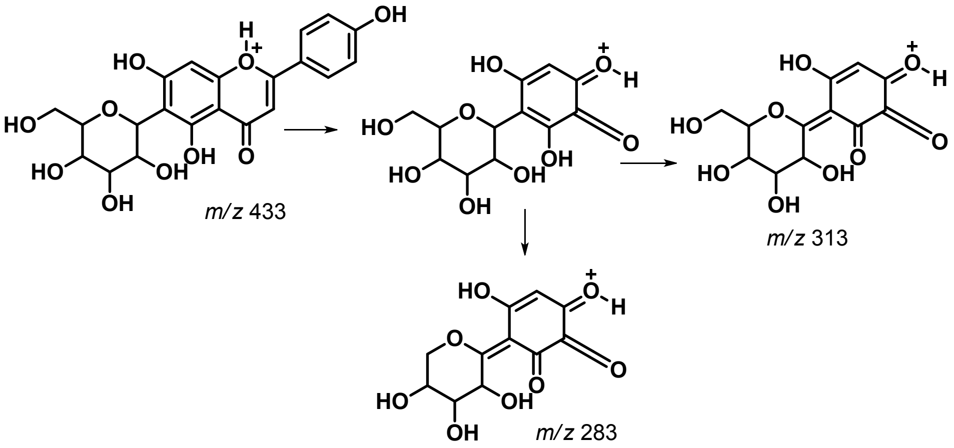

Scheme 51. MS fragmentation patterns for flavonoid Isovitexin (**51**).

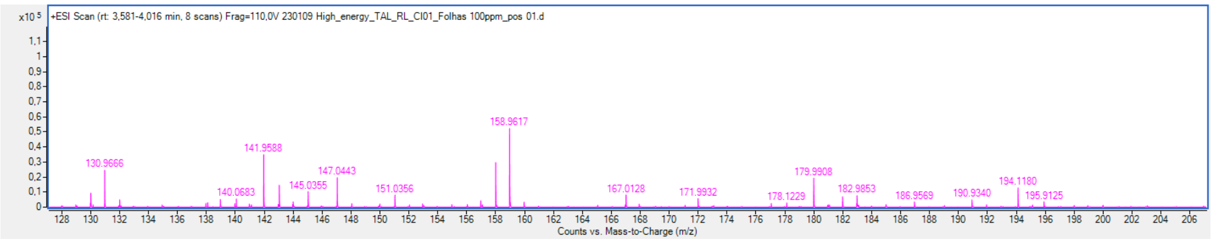

MS[1] spectrum from Coumarin (**52**), $[M+H]^+$ *m/z* 147.0443, error: 1.4 ppm.

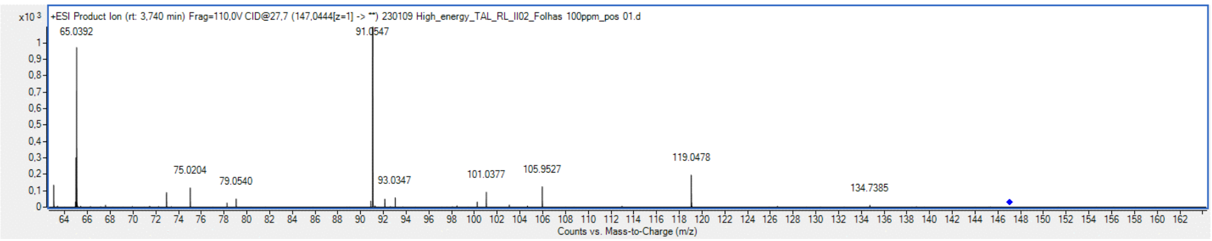

MS/MS spectrum from Coumarin (**52**).

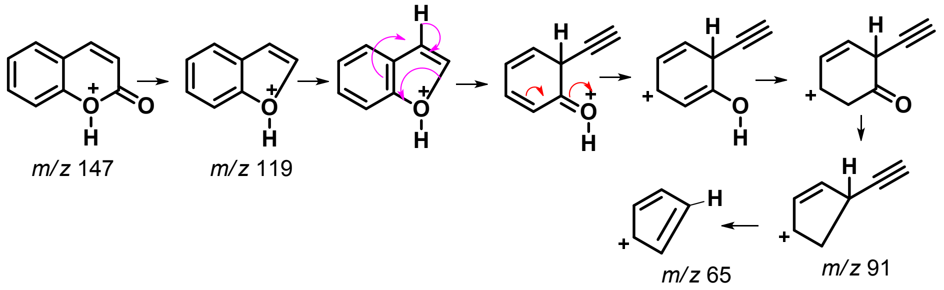

Scheme 52. MS fragmentation patterns for Coumarin (**52**).

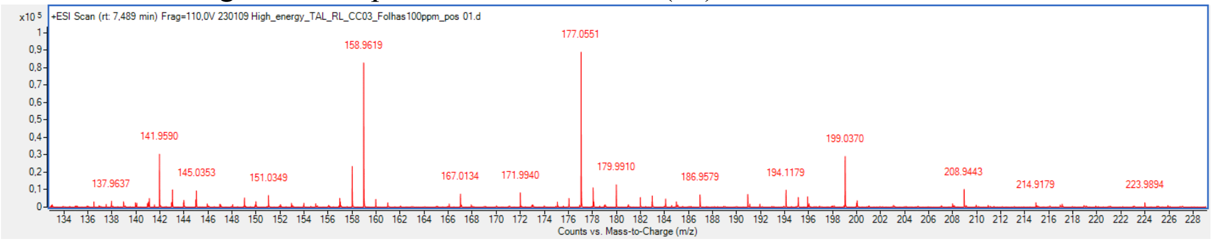

MS[1] spectrum from Herniarin (**53**),), $[M+H]^+$ *m/z* 177.0551, error: 2.8 ppm.

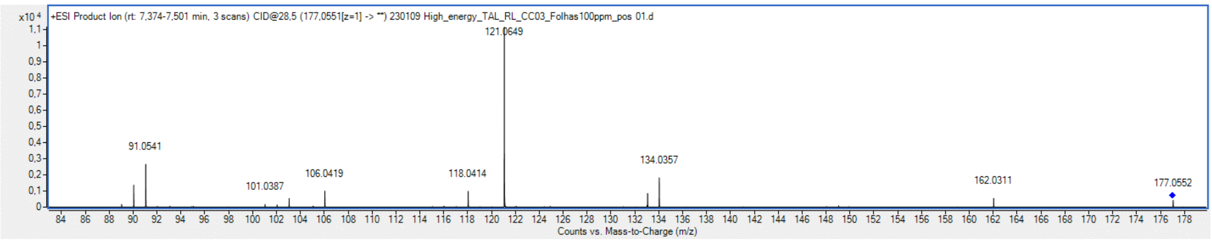

MS/MS spectrum from Herniarin (**53**).

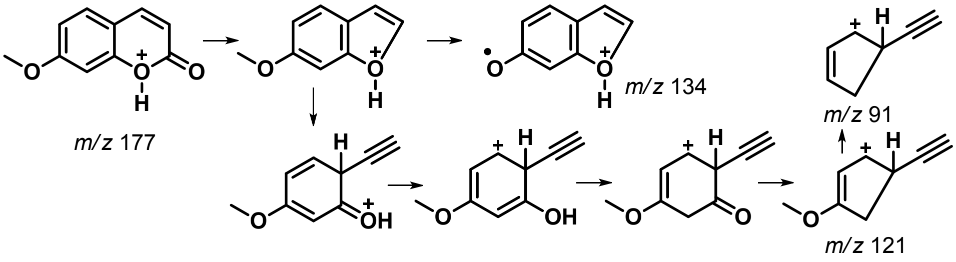

Scheme 53. MS fragmentation patterns for coumarin Herniarin (**53**).

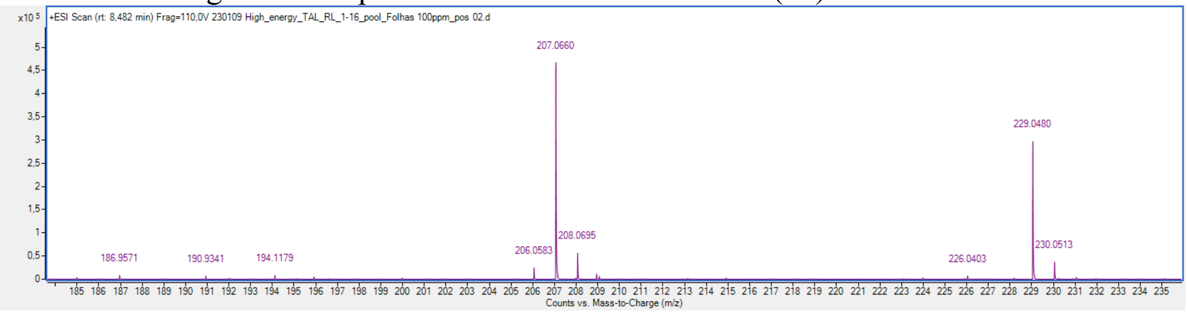

MS[1] spectrum from Limettin (**54**), $[M+H]^+$ *m/z* 207.0660, error: 3.9 ppm.

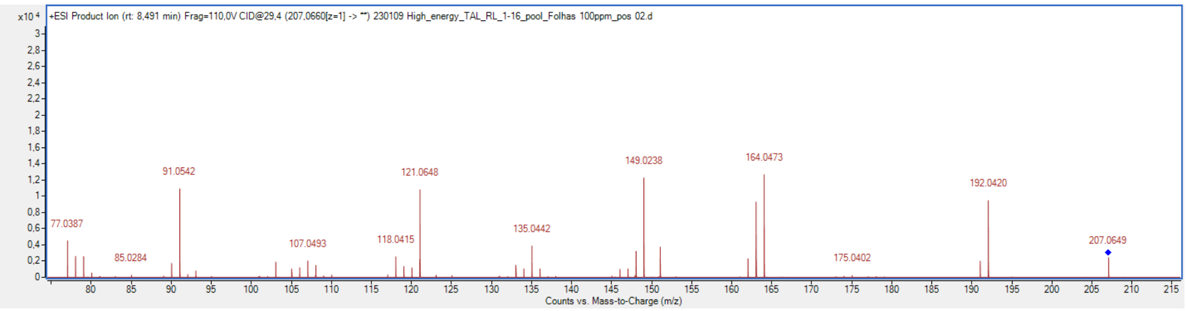

MS/MS spectrum from Limettin (**54**).

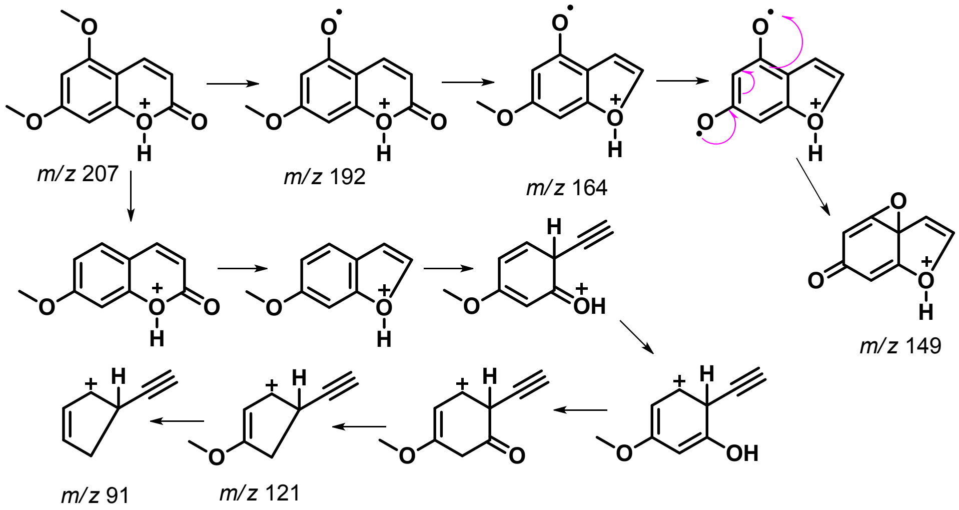

Scheme 54. MS fragmentation patterns for coumarin Limettin (**54**).

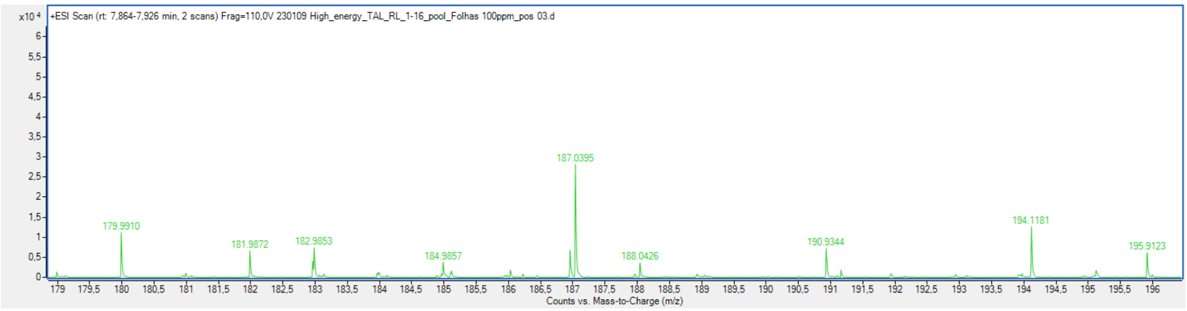

MS[1] spectrum from Psoralen (**55**), $[M+H]^+$ *m/z* 187.0395, error: 2.7 ppm.

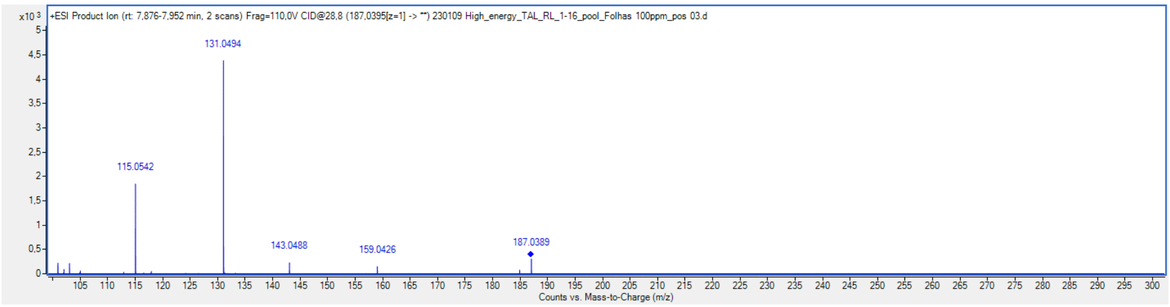

MS/MS spectrum from Psoralen (**55**).

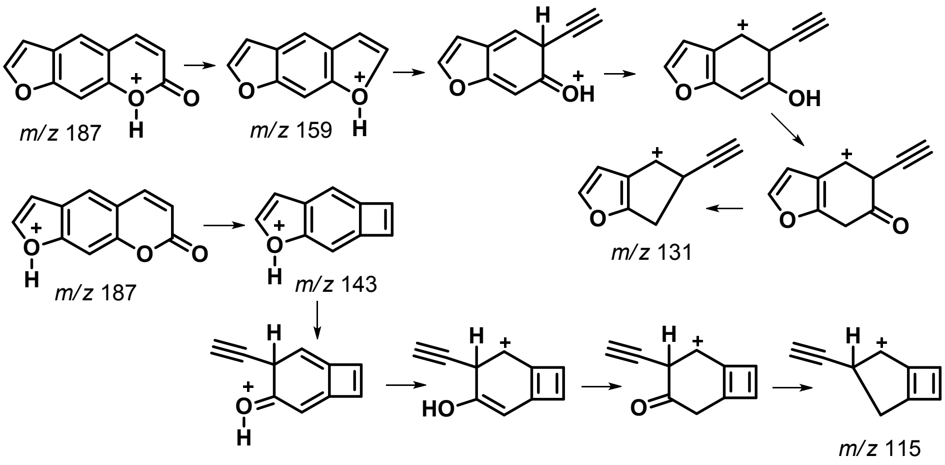

Scheme 55. MS fragmentation patterns for coumarin Psoralen (**55**).

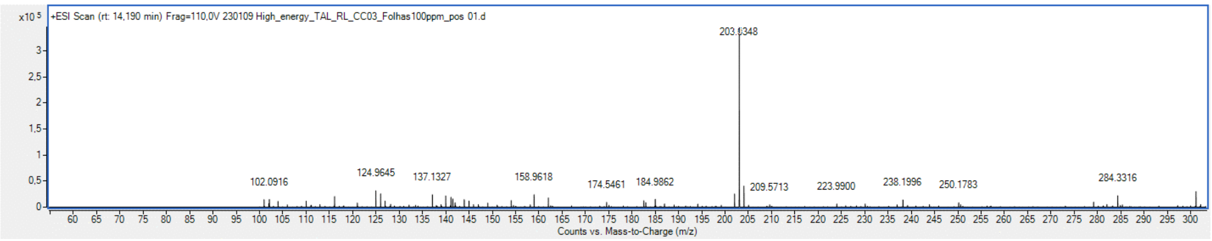

MS[1] spectrum from Bergaptol (**56**), [M+H]$^+$ *m/z* 203.0348, error: 3.9 ppm.

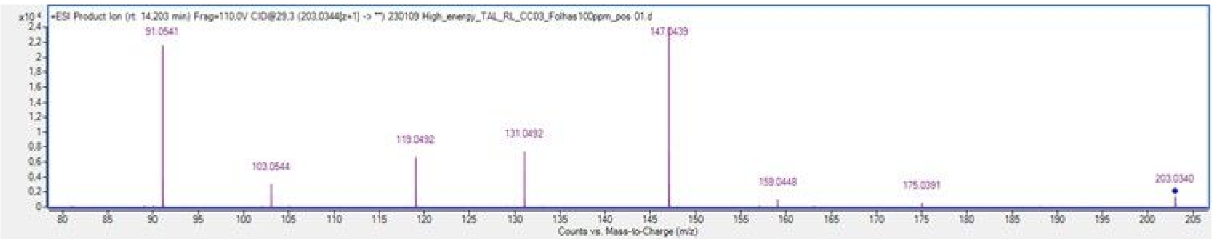

MS/MS spectrum from Bergaptol (**56**).

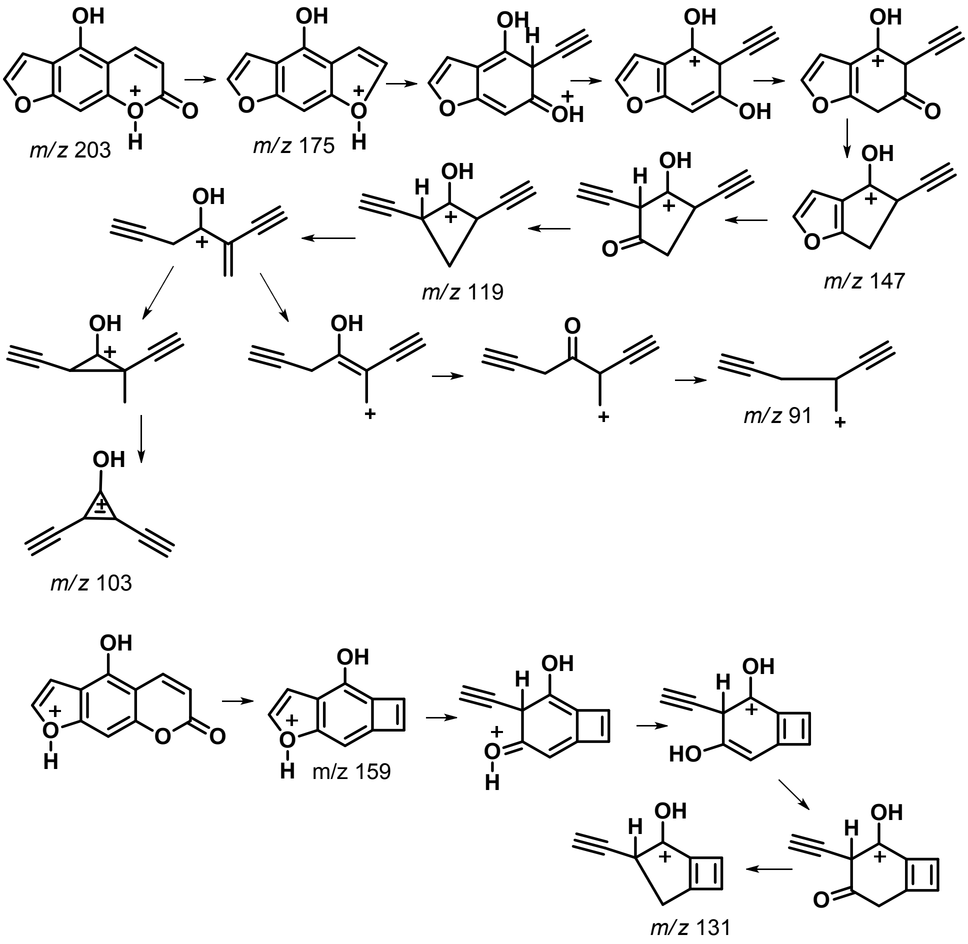

Scheme 56. MS fragmentation patterns for coumarin Bergaptol (**56**).

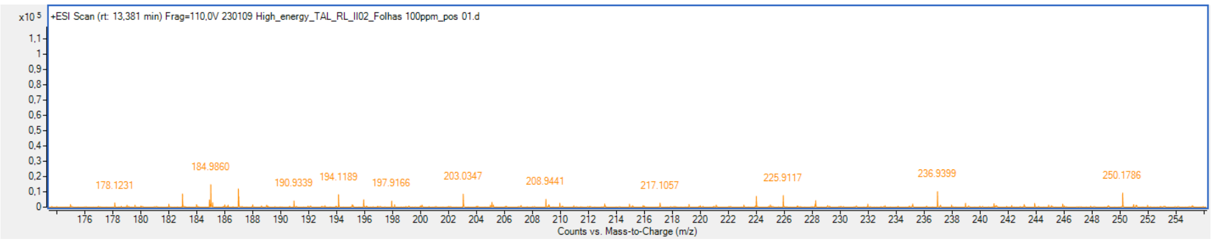

MS[1] spectrum from Xanthotoxol (**57**), [M+H]$^+$ *m/z* 203.0347, error: 3.9 ppm.

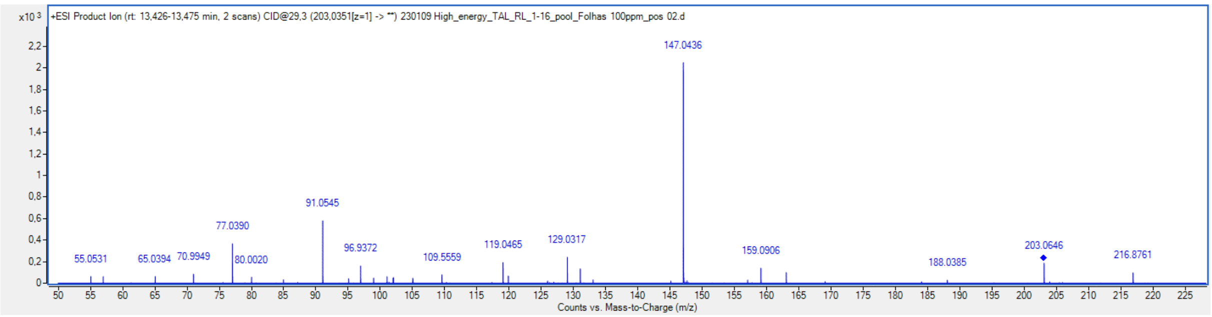

MS/MS spectrum from Xanthotoxol (**57**).

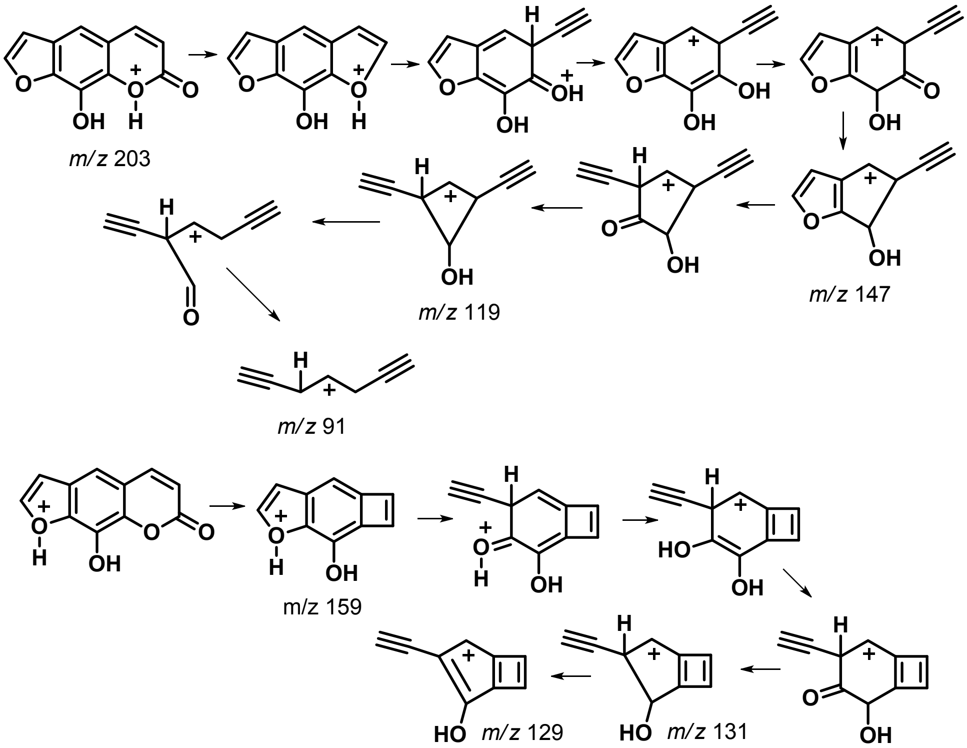

Scheme 57. MS fragmentation patterns for coumarin Xanthotoxol (**57**).

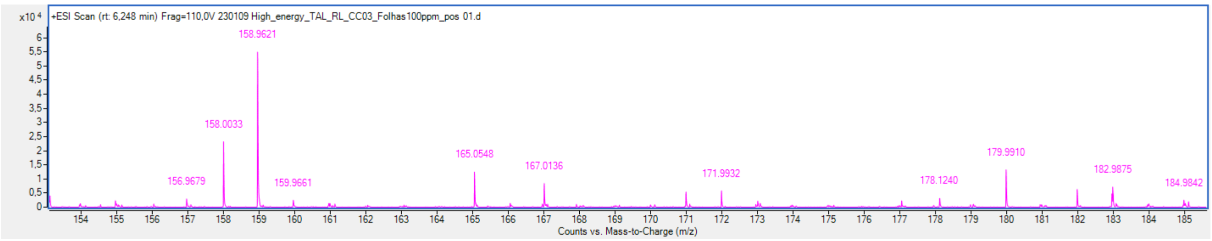

MS^1 spectrum from *p*-hydroxycinnamic acid (**58**), $[M+H]^+$ *m/z* 165.0548, error: 1.21 ppm.

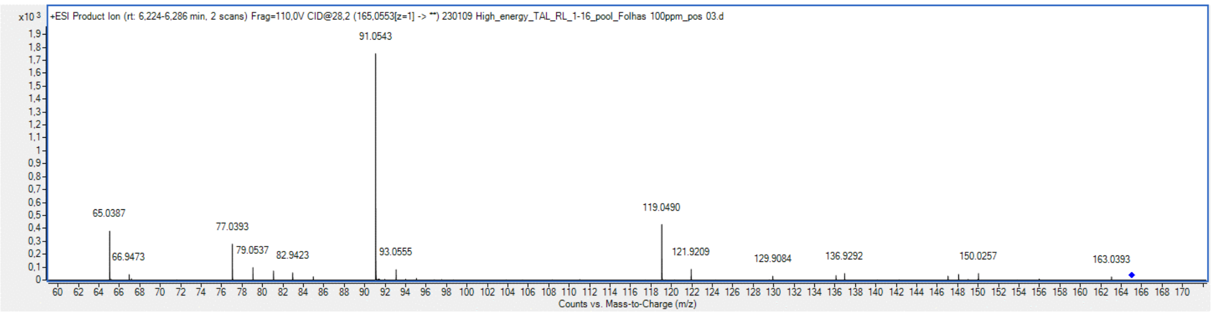

MS/MS spectrum from *p*-hydroxycinnamic acid (**58**).

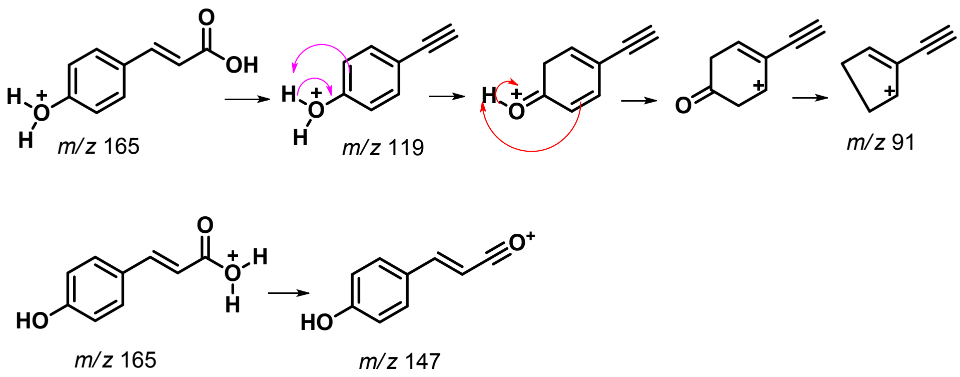

Scheme 58. MS fragmentation patterns for *p*-hydroxycinnamic acid (**58**).

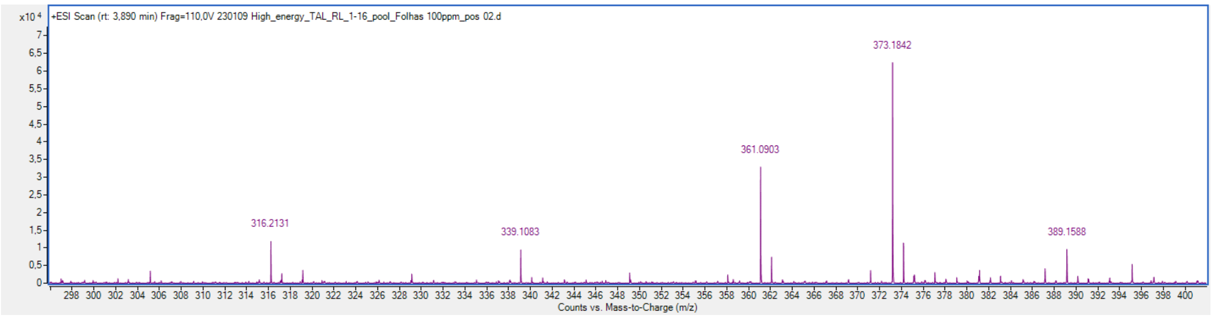

MS[1] spectrum from 3-*O*-p-coumaroylquinic acid (**59**) $[M+H]^+$ *m/z* 339.1083, error: 2.7 ppm.

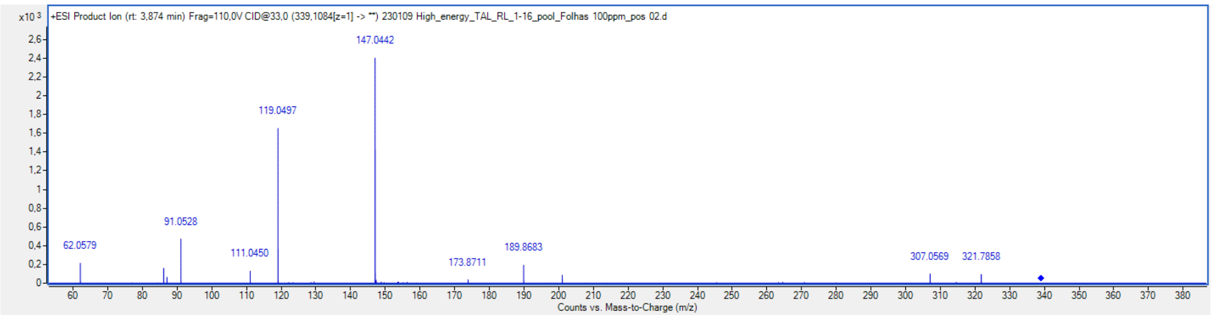

MS/MS spectrum from 3-*O*-p-coumaroylquinic acid (**59**).

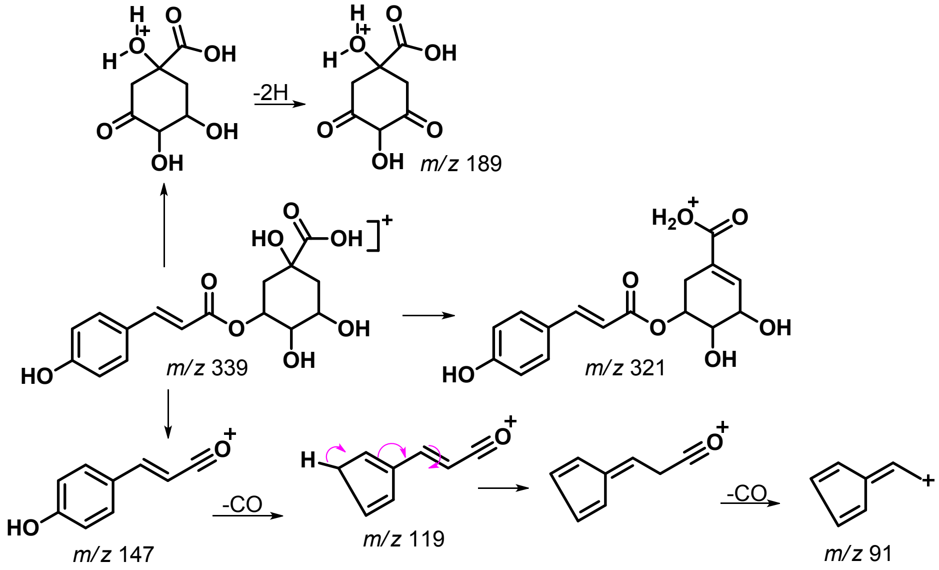

Scheme 59. MS fragmentation patterns for 3-*O*-p-coumaroylquinic acid (**59**).

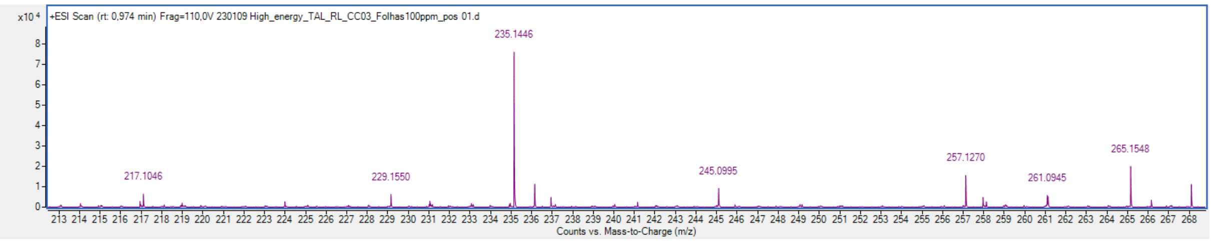

MS[1] spectrum from Coumaroylputrescine (**60**), $[M+H]^+$ *m/z* 235.1446, error: 3.98 ppm.

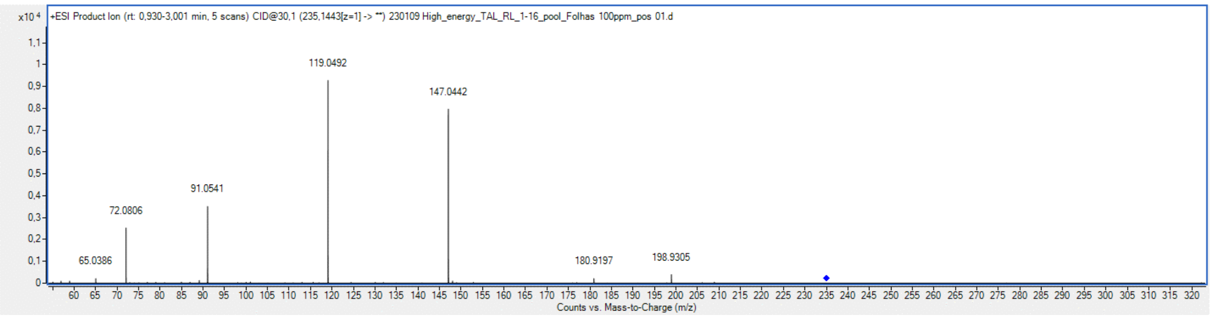

MS/MS spectrum from Coumaroylputrescine (**60**).

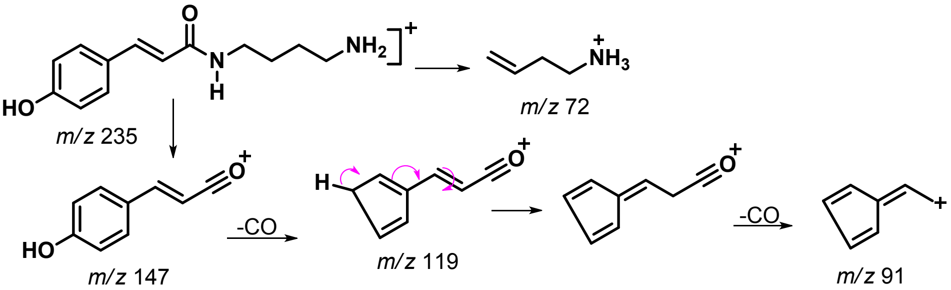

Scheme 60. MS fragmentation patterns for Coumaroylputrescine (**60**).

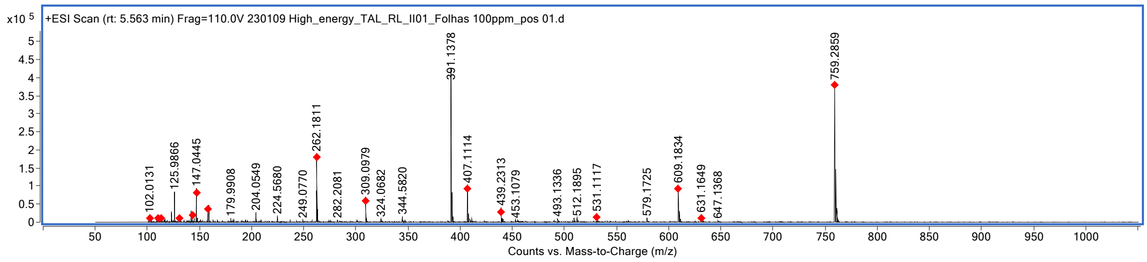

MS[1] spectrum from *p*-Coumaroylglucose (**61**), $[M+H-H_2O]^+$ *m/z* 309.0979, error: 3.98 ppm.

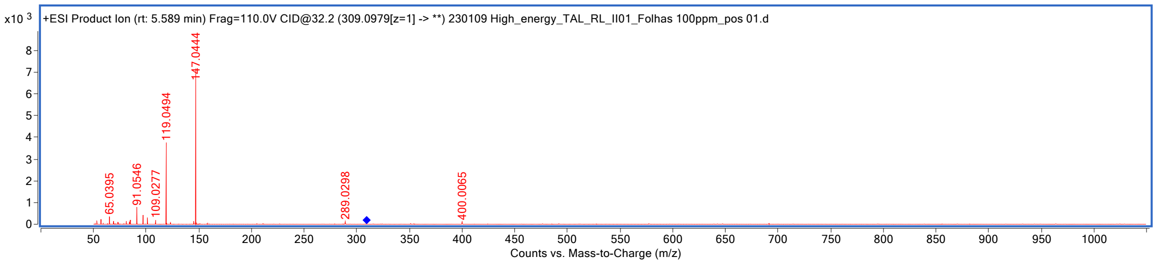

MS/MS spectrum from *p*-Coumaroylglucose (**61**).

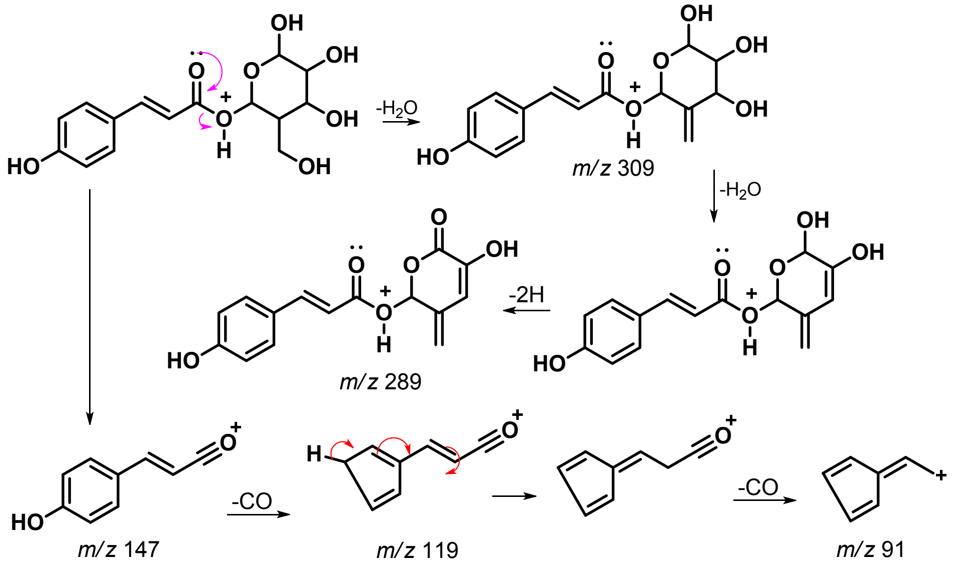

Scheme 61. MS fragmentation patterns for *p*-Coumaroylglucose (**61**).

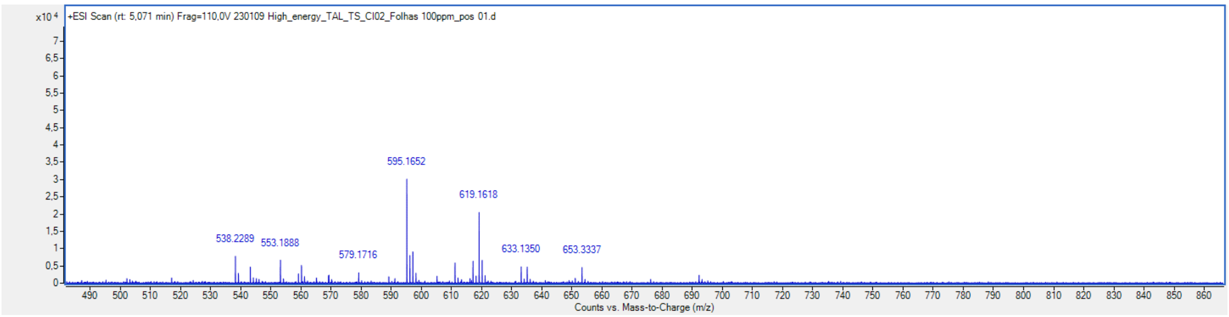

MS[1] spectrum from Kaempferol 3-*O*-rutinoside (**62**), $[M+H]^+$ *m/z* 595.1652, error: 0.8 ppm.

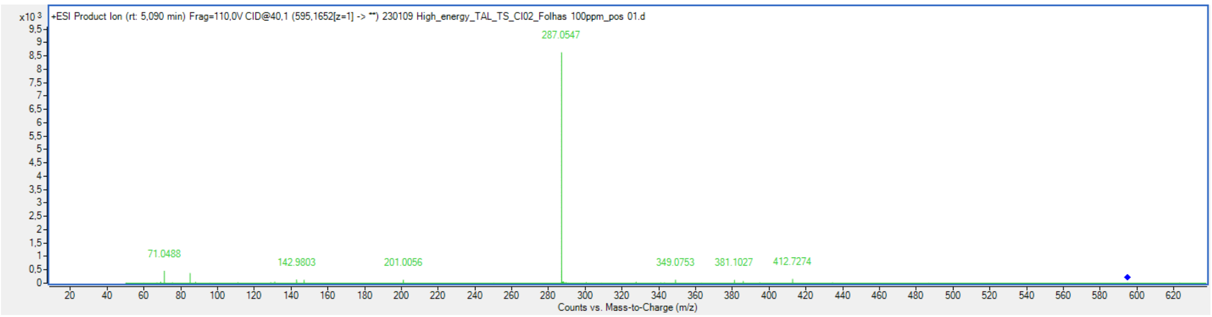

MS/MS spectrum from Kaempferol 3-*O*-rutinoside (**62**).

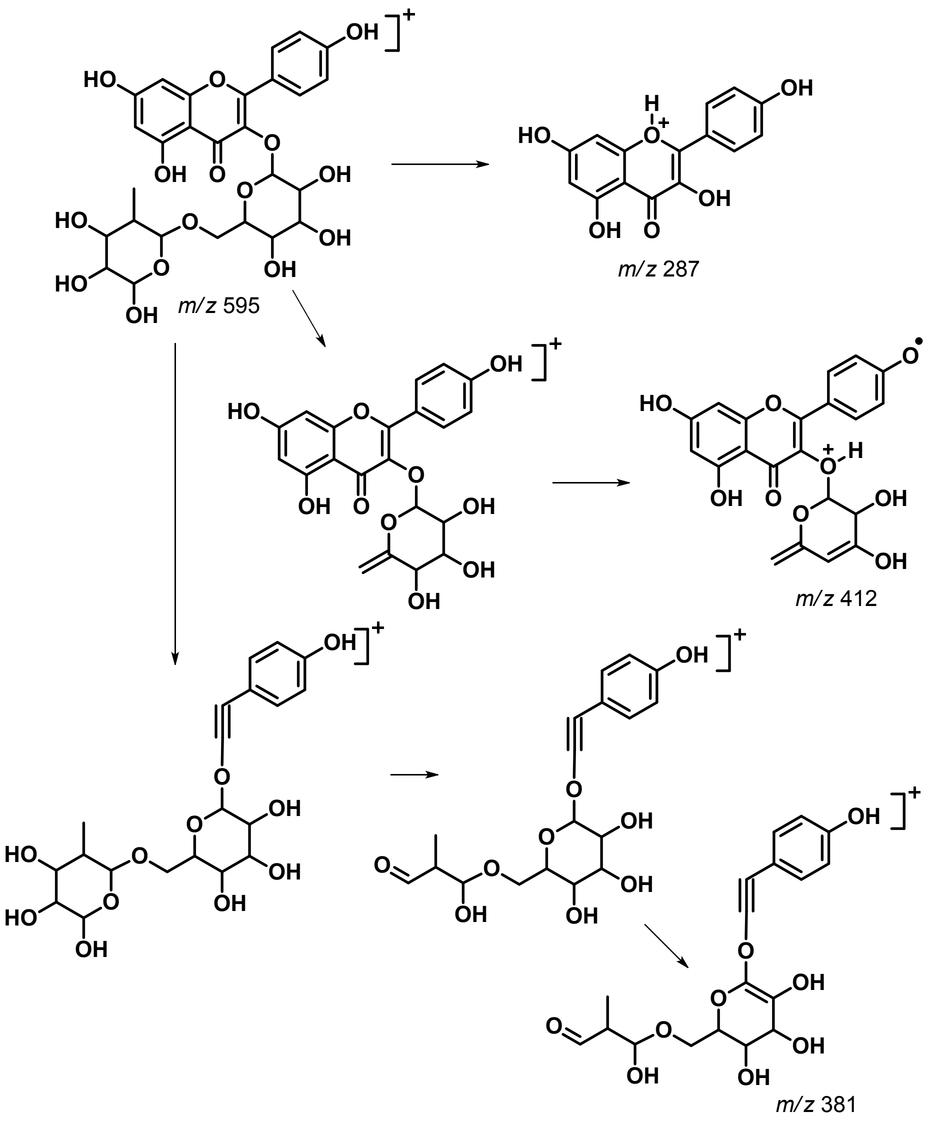

Scheme 62. MS fragmentation patterns for flavonoid Kaempferol 3-*O*-rutinoside (**62**).

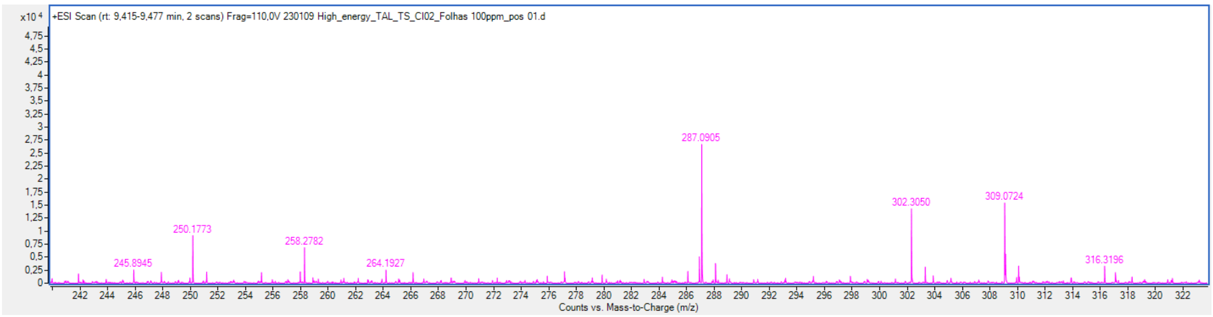

MS[1] spectrum from Oxypeucedanin (**63**), $[M+H]^+$ *m/z* 287.0905, and $[M+Na^+]$ *m/z* 309.0724, error: 3.1 ppm.

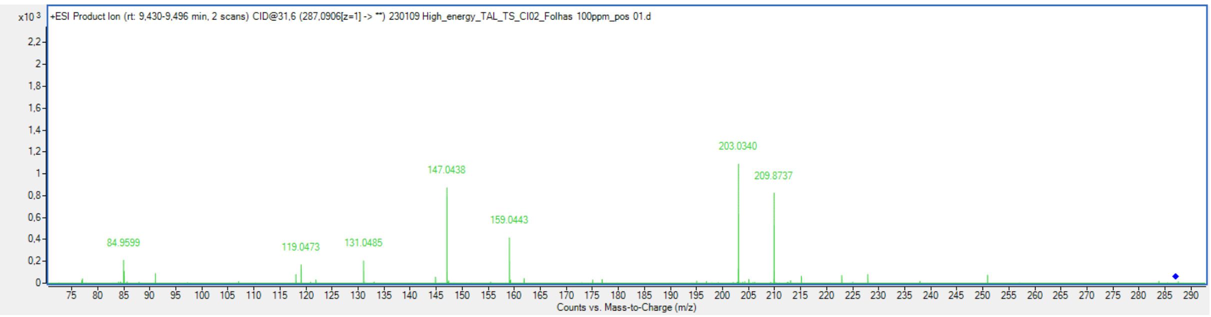

MS/MS spectrum from Oxypeucedanin (**63**).

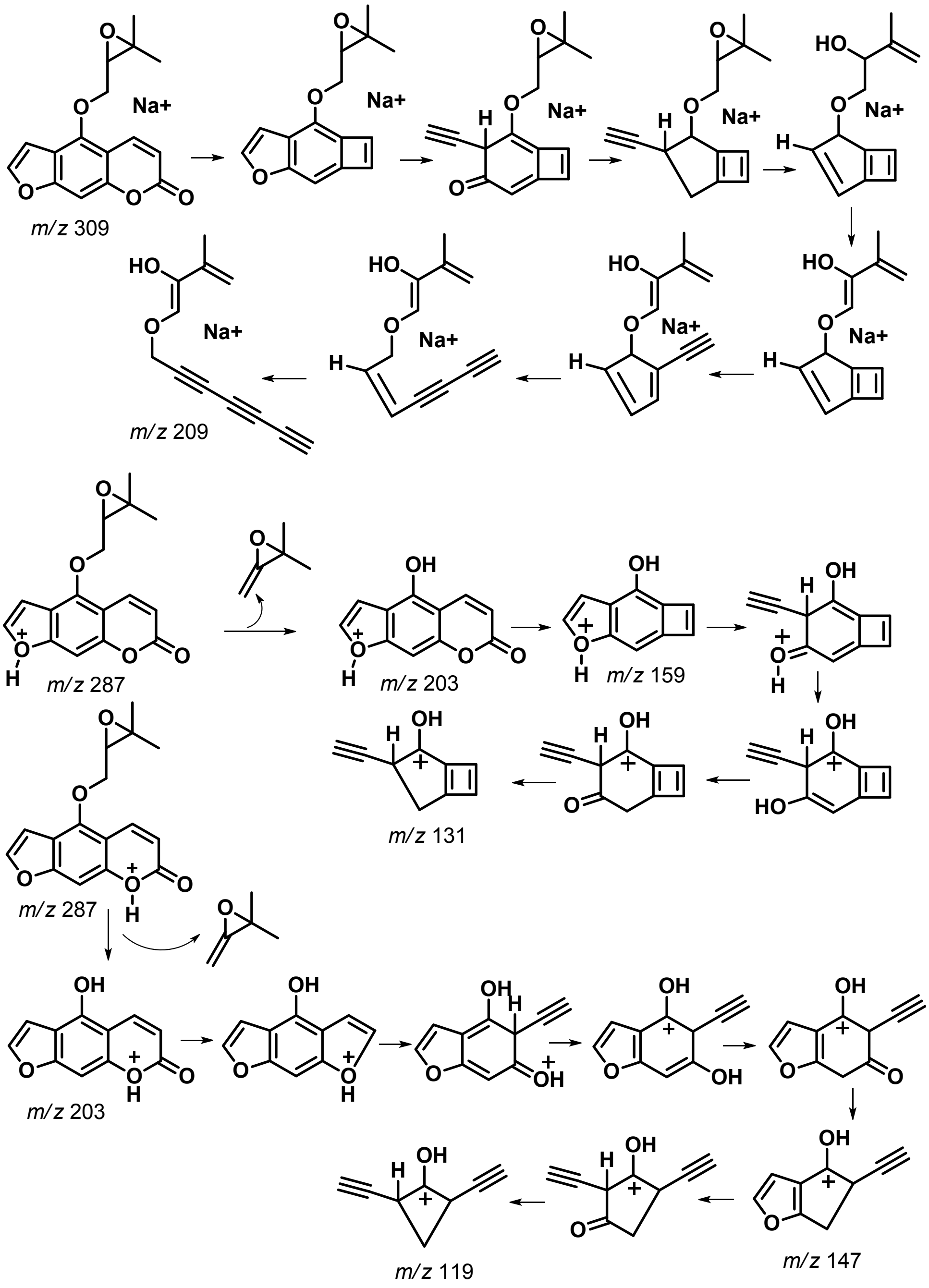

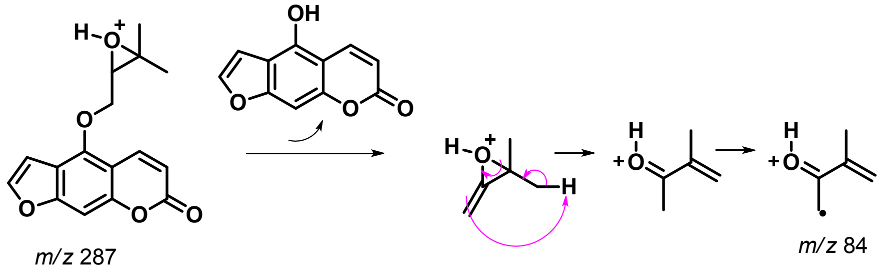

Scheme 63. MS fragmentation patterns for coumarin Oxypeucedanin (**63**).

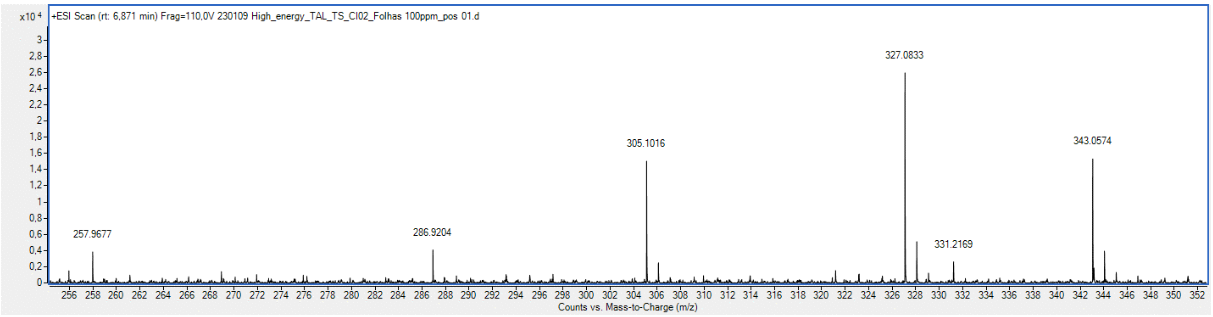

MS[1] spectrum from Oxypeucedanin hydrate (**64**), $[M+H]^+$ *m/z* 305.1016, $[M+Na^+]$ 327.0833 *m/z*, error: 1.3 ppm.

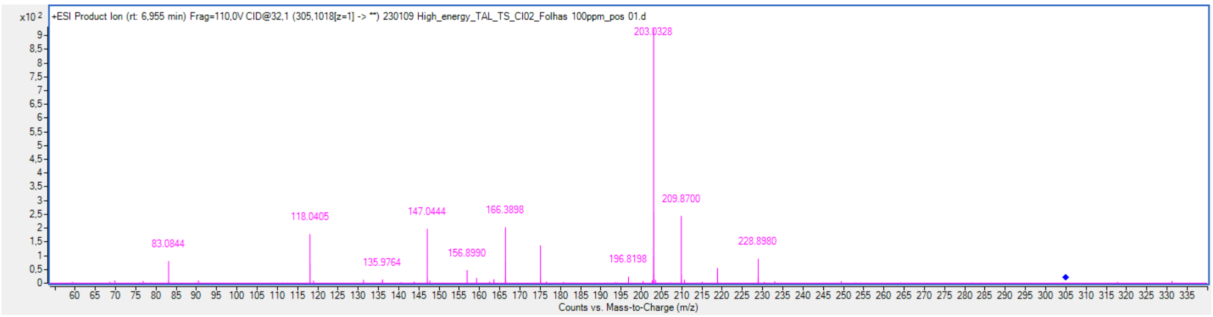

MS/MS spectrum from Oxypeucedanin hydrate (**64**).

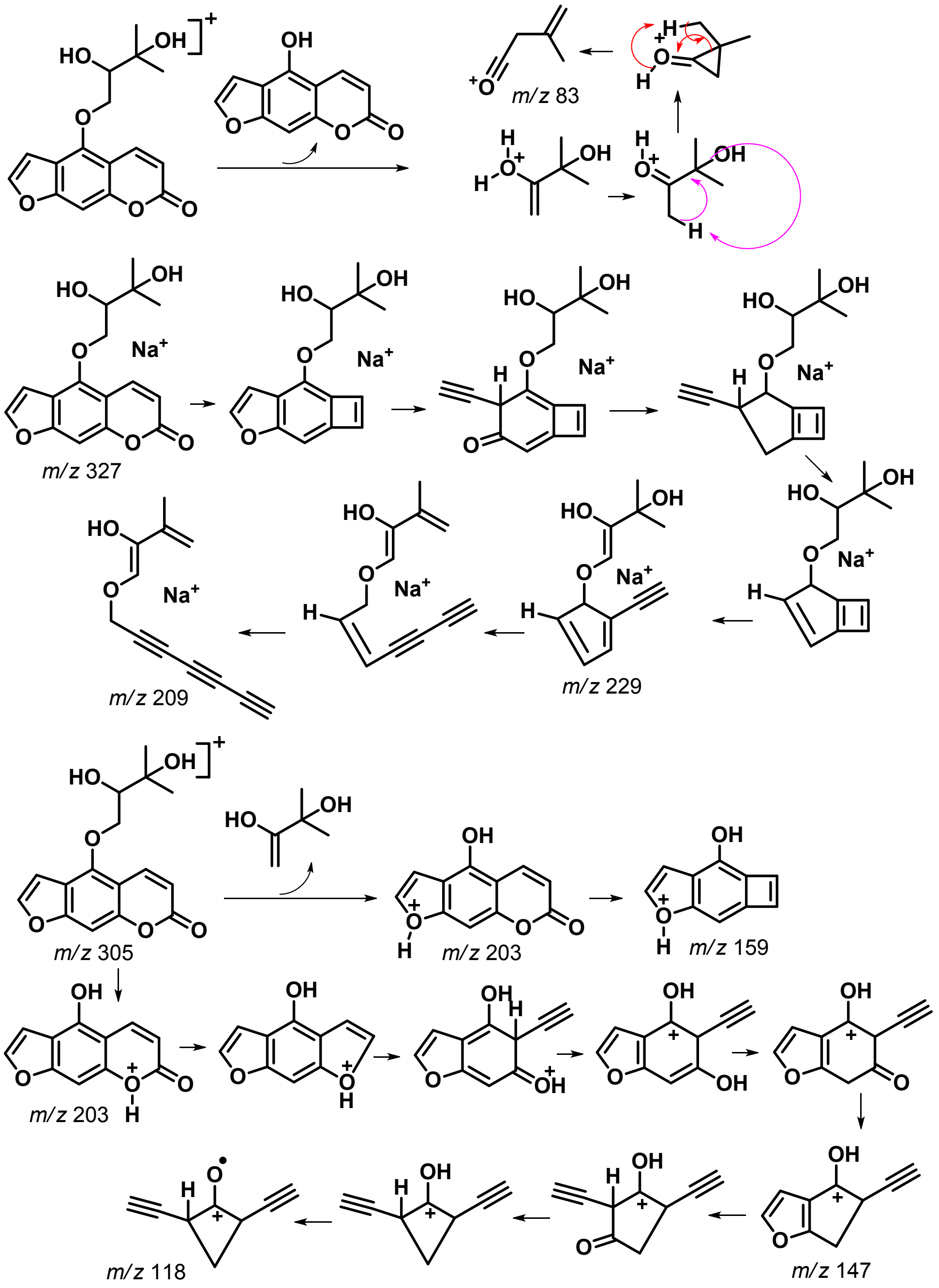

Scheme 64. MS fragmentation patterns for coumarin Oxypeucedanin hydrate (**64**).

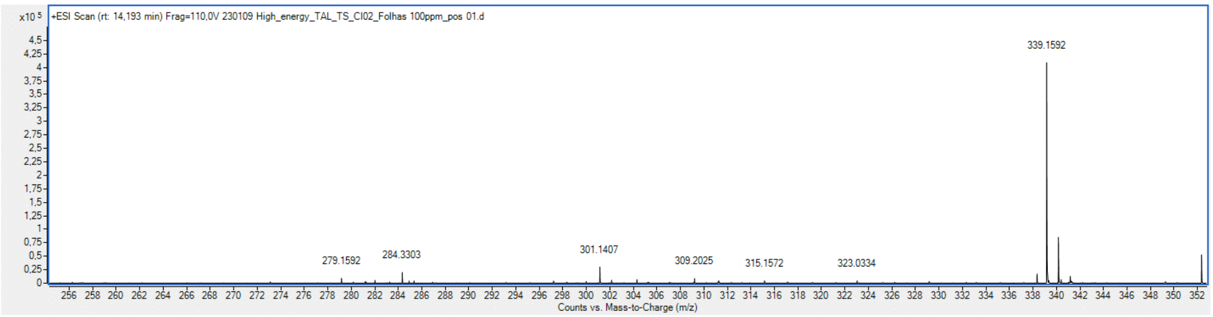

MS[1] spectrum from Bergamottin (**65**), $[M+H]^+$ *m/z* 339,1592, error: 0.3 ppm.

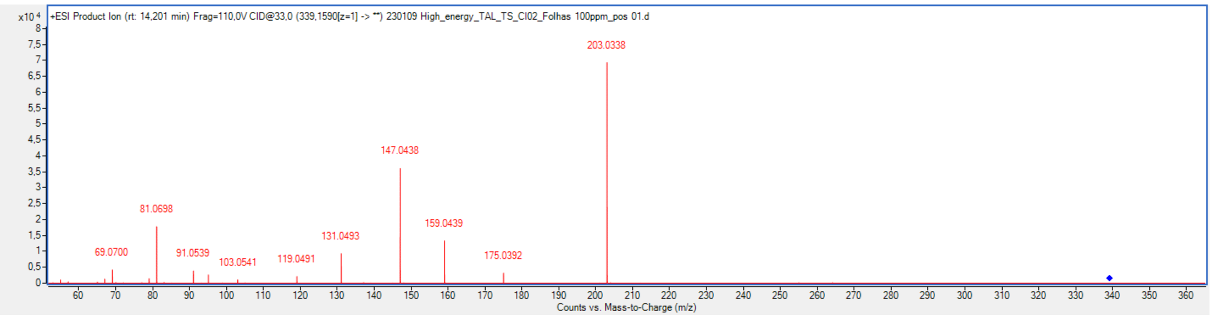

MS/MS spectrum from Bergamottin (**65**).

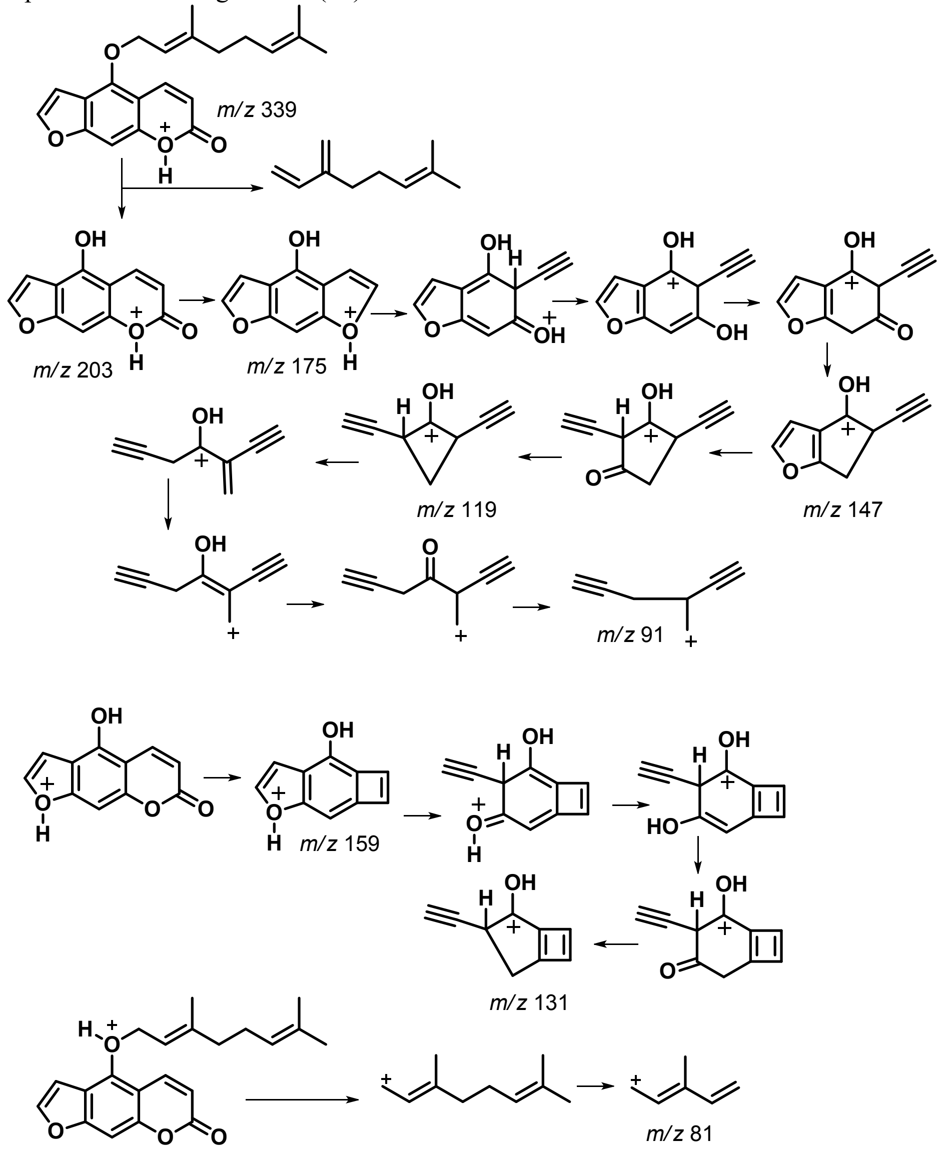

Scheme 65. MS fragmentation patterns for coumarin Bergamottin (**65**).

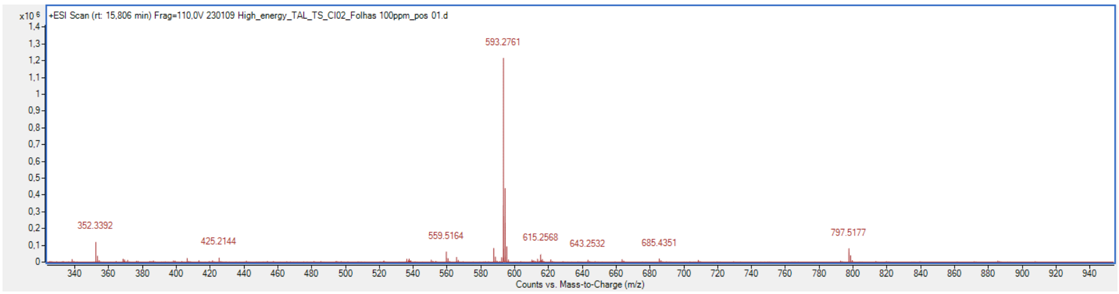

MS^1 spectrum from Pheophorbide A (**66**), $[M+H]^+$ *m/z* 593.2761, error: 0.5 ppm.

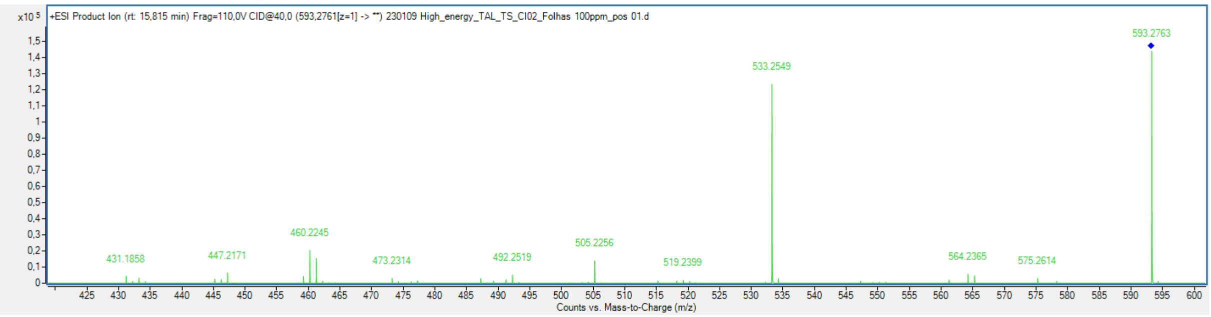

MS/MS spectrum from Pheophorbide A (**66**).

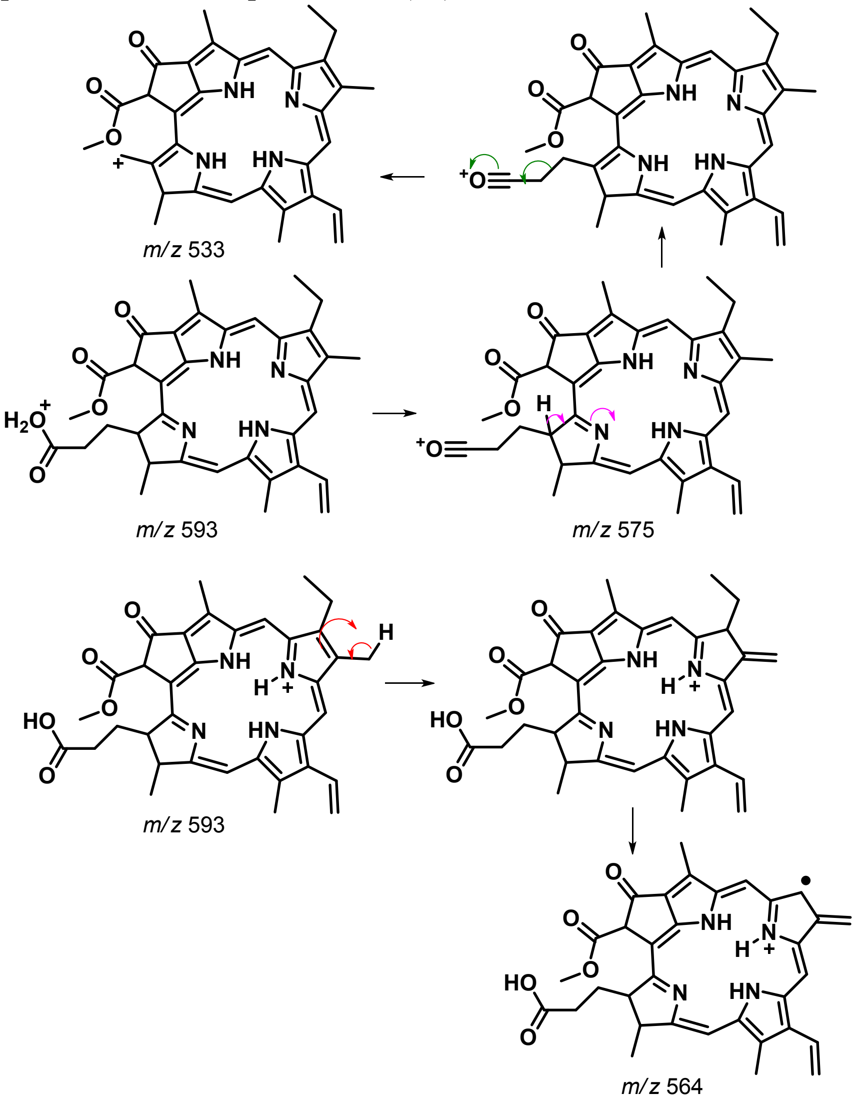

Scheme 66. MS fragmentation patterns for Pheophorbide A (**66**).

Table S2. Features exhibiting a coefficient of variation (CV%) greater than 20% in QC samples were removed from downstream data analysis to ensure data quality.

Metric	Summary
Median QC RSD percent	13 (IQR 7–22)
Features with QC RSD percent ≤ 20 percent	70%
Present in all QC replicates	>90%

Table S3. Univariate analysis of metabolites (IDs 1–66): mean ± SD, ANOVA p, FDR, fold change and log2FC versus control.

ID	Metabolite	Tissue	Control (mean ± SD)	Inoc_A (mean ± SD)	Inoc_B (mean ± SD)	Reinoc (mean ± SD)	p.value	FDR	F.stat	-log10(p)	FC_Control_vs_Inoc_A	log2FC_Control_vs_Inoc_A	FC_Control_vs_Inoc_B	log2FC_Control_vs_Inoc_B	FC_Control_vs_Reinoc	log2FC_Control_vs_Reinoc
1	osthenol (**1**)	Root	22,283 ± 39,926	7,803 ± 15,826	26,160 ± 47,325	7,177 ± 13,150	9.72e-33	2.34e-32	119.913	32.012	2.856	1.514	0.852	-0.231	3.105	1.635
2	osthol (**2**)	Root	98,069 ± 73,920	128,152 ± 128,459	123,528 ± 142,077	152,869 ± 143,621	4.79e-25	7.41e-25	55.200	24.320	0.765	-0.386	0.794	-0.333	0.642	-0.640
3	auraptenol (**3**)	Root	204,528 ± 373,701	280,074 ± 514,695	187,715 ± 385,528	296,486 ± 586,724	3.27e-16	3.67e-16	21.284	15.485	0.730	-0.454	1.090	0.124	0.690	-0.536
4	angenomalin (**4**)	Root	18,799 ± 32,001	5,781 ± 10,538	17,340 ± 29,406	5,312 ± 8,447	7.60e-32	1.70e-31	109.733	31.119	3.252	1.701	1.084	0.117	3.539	1.823
5	nordentatin (**5**)	Root	41,008 ± 75,080	118.583 ± 273.051	11,757 ± 25,353	5,804 ± 11,457	2.04e-23	2.76e-23	46.621	22.691	345.813	8.434	3.488	1.802	7.066	2.821
6	clausarin (**6**)	Root	222,730 ± 252,646	172,263 ± 313,366	111,734 ± 116,501	77,369 ± 85,647	3.35e-19	3.96e-19	29.740	18.475	1.293	0.371	1.993	0.995	2.879	1.525
7	xanthoxyletin (**7**)	Root	52,049 ± 93,535	57,598 ± 124,934	44,237 ± 79,600	34,082 ± 61,037	6.01e-15	6.62e-15	18.374	14.221	0.904	-0.146	1.177	0.235	1.527	0.611
8	moriramulosid B (**8**)	Root	84,454 ± 25,453	82,116 ± 29,700	82,945 ± 49,668	97,855 ± 55,602	1.15e-12	1.23e-12	13.924	11.938	1.028	0.040	1.018	0.026	0.863	-0.212
9	haploperoside A (**9**)	Root	102,680 ± 106,331	80,069 ± 85,411	83,551 ± 89,752	86,921 ± 93,193	6.62e-34	1.87e-33	134.595	33.179	1.282	0.359	1.229	0.297	1.181	0.240
10	moriramulosid B 6-glyceroyl (**10**)	root	1,353 ± 2,423	1,682 ± 3,927	1,035 ± 1,888	753 ± 1,416	4.93e-10	5.01e-10	9.853	9.307	0.804	-0.314	1.307	0.386	1.797	0.846
11	limonin (**11**)	root	9,331 ± 16,737	16,572 ± 30,542	10,051 ± 18,394	16,576 ± 31,194	1.78e-23	2.46e-23	46.907	22.750	0.563	-0.829	0.928	-0.107	0.563	-0.829
12	nomilin (**12**)	root	22,591 ± 40,706	32,587 ± 62,291	14,266 ± 26,248	21,797 ± 39,882	1.34e-21	1.68e-21	38.498	20.872	0.693	-0.529	1.584	0.663	1.036	0.052
13	umbelliferone (**13**)	root	2,065 ± 3,699	3,187 ± 5,758	2,210 ± 3,995	2,445 ± 4,504	8.91e-33	2.23e-32	120.362	32.050	0.648	-0.626	0.934	-0.098	0.845	-0.243
14	citrubuntin (**14**)	root	6,699 ± 12,206	15,832 ± 28,383	5,959 ± 10,689	8,639 ± 15,919	6.49e-37	2.81e-36	180.992	36.188	0.423	-1.241	1.124	0.169	0.775	-0.367
15	seselin (**15**)	root	316.250 ± 717.873	165.750 ± 301.679	1,143 ± 2,177	263.333 ± 502.655	1.49e-16	1.73e-16	22.135	15.828	1.908	0.932	0.277	-1.853	1.201	0.264
16	luvangetin (**16**)	root	18,799 ± 32,001	5,781 ± 10,538	17,340 ± 29,406	5,312 ± 8,447	7.60e-32	1.70e-31	109.733	31.119	3.252	1.701	1.084	0.117	3.539	1.823
17	5-hydroxynoracronicine (**17**)	root	5,929 ± 11,701	10,442 ± 18,840	3,196 ± 5,739	8,707 ± 15,701	1.64e-27	2.73e-27	71.016	26.785	0.568	-0.816	1.855	0.891	0.681	-0.554
18	citracridone III (**18**)	root	1,318 ± 2,372	9,686 ± 17,340	10,421 ± 18,666	20,631 ± 36,911	1.66e-68	1.08e-66	3829.567	67.780	0.136	-2.877	0.127	-2.983	0.064	-3.968

Table S3. Continuation.

19	clausine-L (**19**)	root	1,272 ± 2,291	1,768 ± 3,175	964 ± 1,736	1,654 ± 2,968	9.70e-44	1.58e-42	352.132	43.013	0.720	-0.474	1.320	0.400	0.769	-0.378
20	cytidine (**20**)	root	3,792 ± 7,010	4,351 ± 7,981	6,097 ± 10,955	3,457 ± 6,274	4.10e-31	8.32e-31	102.019	30.388	0.872	-0.198	0.622	-0.685	1.097	0.134
21	guanosine (**21**)	root	22,680 ± 42,788	34,953 ± 65,734	29,001 ± 51,912	27,738 ± 51,663	1.34e-22	1.78e-22	42.787	21.872	0.649	-0.624	0.782	-0.355	0.818	-0.290
22	adenosine (**22**)	root	8,333 ± 15,555	10,560 ± 19,697	11,760 ± 21,078	9,918 ± 18,255	6.26e-26	1.02e-25	60.442	25.203	0.789	-0.342	0.709	-0.497	0.840	-0.251
23	proline (**23**)	root	166,842 ± 298,703	161,738 ± 292,158	150,687 ± 278,712	234,991 ± 420,630	1.26e-37	6.31e-37	194.067	36.899	1.032	0.045	1.107	0.147	0.710	-0.494
24	arginine (**24**)	root	3,405 ± 6,217	1,748 ± 3,220	885 ± 1,593	1,490 ± 2,911	6.86e-25	1.01e-24	54.316	24.163	1.948	0.962	3.847	1.944	2.286	1.193
25	lochnocarpol A (**25**)	root	1,551 ± 3,065	11,590 ± 21,428	1,312 ± 2,586	1,591 ± 3,309	7.70e-24	1.09e-23	48.718	23.113	0.134	-2.902	1.182	0.242	0.975	-0.037
26	amygdalin amide (**26**)	root	695 ± 1,413	1,099 ± 2,449	842 ± 1,725	2,507 ± 4,845	6.16e-13	6.67e-13	14.406	12.210	0.633	-0.660	0.826	-0.276	0.277	-1.851
27	stachydrine (**27**)	root	136,305 ± 245,159	140,331 ± 259,538	227,701 ± 424,064	286,587 ± 515,297	3.48e-29	6.29e-29	84.089	28.458	0.971	-0.042	0.599	-0.740	0.476	-1.072
28	[*N*,*N*-dimethyltryptamine (**28**)	root	155.167 ± 299.672	238.250 ± 587.771	1,162 ± 2,100	457.583 ± 865.985	5.54e-25	8.38e-25	54.839	24.256	0.651	-0.619	0.134	-2.905	0.339	-1.560
29	N-hexanoyltryptamine (**29**)	root	280.500 ± 527.747	864 ± 1,734	1,411 ± 2,670	1,204 ± 2,747	1.20e-10	1.24e-10	10.715	9.920	0.325	-1.623	0.199	-2.330	0.233	-2.102
30	5-methoxytryptophan (**30**)	root	633 ± 1,164	918 ± 1,741	1,709 ± 3,069	1,732 ± 3,177	1.29e-28	2.26e-28	79.416	27.890	0.689	-0.537	0.370	-1.433	0.366	-1.451
31	6-demethoxy-tangeretin (**31**)	leave	30,170 ± 54,456	70,986 ± 127,850	48,607 ± 87,945	33,702 ± 62,852	1.17e-33	3.16e-33	131.370	32.934	0.425	-1.234	0.621	-0.688	0.895	-0.160
32	tetra-*O*-methylscutellarein (**32**)	leave	32,590 ± 53,183	9,601 ± 11,745	8,738 ± 10,212	5,665 ± 5,900	6.57e-38	3.88e-37	199.519	37.182	3.394	1.763	3.730	1.899	5.753	2.524
33	sinensetin (**33**)	leave	241,427 ± 324,344	281,606 ± 295,763	213,725 ± 231,490	209,429 ± 247,675	1.10e-35	3.97e-35	160.419	34.959	0.857	-0.222	1.130	0.176	1.153	0.205
34	6-demethoxynobiletin (isosinensetin) (**34**)	leave	5,079 ± 9,136	15,986 ± 28,826	11,733 ± 21,192	7,441 ± 13,329	6.54e-39	4.72e-38	220.065	38.184	0.318	-1.654	0.433	-1.208	0.683	-0.551
35	tangeritin (**35**)	leave	50,269 ± 65,304	66,536 ± 70,380	52,454 ± 56,289	46,678 ± 59,714	3.96e-31	8.30e-31	102.171	30.402	0.756	-0.404	0.958	-0.061	1.077	0.107

Table S3. Continuation.

36	3'-demethylnobiletin (**36**)	leave	3,905 ± 5,137	5,455 ± 5,773	6,068 ± 7,524	4,000 ± 4,562	2.23e-29	4.27e-29	85.736	28.651	0.716	-0.482	0.644	-0.636	0.976	-0.035
37	5-*O*-demethylnobiletin (**37**)	leave	17,806 ± 22,828	24,964 ± 26,180	17,216 ± 18,453	17,005 ± 21,091	3.26e-33	8.49e-33	125.680	32.486	0.713	-0.487	1.034	0.049	1.047	0.066
38	nobiletin (**38**)	leave	224,393 ± 296,274	261,965 ± 277,821	198,811 ± 212,511	190,518 ± 221,931	1.43e-35	4.89e-35	158.626	34.845	0.857	-0.223	1.129	0.175	1.178	0.236
39	5-Hydroxy-3,6,7,8,3',4'-hexamethoxyflavone (**39**)	leave	9,882 ± 12,850	12,246 ± 12,940	6,448 ± 7,006	7,938 ± 9,527	2.76e-34	8.53e-34	139.756	33.560	0.807	-0.310	1.533	0.616	1.245	0.316
40	eupatorin (**40**)	leave	3,062 ± 5,498	7,518 ± 13,578	5,227 ± 9,368	3,160 ± 5,678	2.51e-40	2.33e-39	252.663	39.600	0.407	-1.296	0.586	-0.771	0.969	-0.045
41	vitexin (**41**)	leave	3,246 ± 4,400	2,031 ± 2,344	4,259 ± 4,972	3,164 ± 4,725	2.00e-22	2.54e-22	42.019	21.700	1.599	0.677	0.762	-0.392	1.026	0.037
42	swertiajaponin (**42**)	leave	3,747 ± 5,169	5,452 ± 6,917	4,854 ± 5,368	4,260 ± 5,308	2.34e-21	2.87e-21	37.519	20.630	0.687	-0.541	0.772	-0.373	0.880	-0.185
43	isovitexin 2''-*O*-arabinoside (**43**)	leave	19,196 ± 21,158	14,824 ± 17,365	19,768 ± 20,676	7,048 ± 9,394	4.90e-21	5.90e-21	36.258	20.310	1.295	0.373	0.971	-0.042	2.724	1.446
44	rhoifolin (**44**)	leave	4,820 ± 8,771	2,604 ± 4,826	1,009 ± 1,833	1,361 ± 2,437	2.37e-29	4.40e-29	85.517	28.625	1.851	0.889	4.776	2.256	3.542	1.824
45	saponarin (**45**)	leave	3,021 ± 5,459	3,613 ± 6,474	2,372 ± 4,463	3,538 ± 6,342	6.62e-35	2.15e-34	148.564	34.179	0.836	-0.258	1.273	0.349	0.854	-0.228
46	hesperidin (**46**)	leave	2,957 ± 5,696	7,172 ± 12,837	4,704 ± 9,085	6,335 ± 11,380	6.45e-28	1.10e-27	73.996	27.191	0.412	-1.278	0.629	-0.670	0.467	-1.099
47	aspartic acid (**47**)	leave	815 ± 1,461	2,209 ± 3,982	443.000 ± 842.826	854 ± 1,538	6.00e-38	3.88e-37	200.296	37.222	0.369	-1.439	1.839	0.879	0.954	-0.069
48	choline (**48**)	leave	280,660 ± 504,417	303,919 ± 545,784	263,976 ± 480,039	290,438 ± 519,690	4.58e-41	5.95e-40	271.538	40.339	0.923	-0.115	1.063	0.088	0.966	-0.049
49	luteolin-7-*O*-rutinoside (**49**)	leave	3,375 ± 6,060	6,185 ± 11,083	4,343 ± 8,068	4,049 ± 7,438	1.48e-31	3.21e-31	106.610	30.829	0.546	-0.874	0.777	-0.364	0.834	-0.263
50	kaempferide 3-*O*-β-D-glucopyranoside 7-*O*-α-L-rhamnopyranoside (**50**)	leave	0.000 ± 0.000	0.000 ± 0.000	0.000 ± 0.000	0.000 ± 0.000	NA	NA	NA	NA	NA	NA	NA	NA	NA	NA
51	isovitexin (**51**)	leave	7,492 ± 13,528	2,232 ± 4,908	4,898 ± 9,167	4,412 ± 7,967	3.59e-25	5.69e-25	55.914	24.445	3.356	1.747	1.530	0.613	1.698	0.764
52	coumarin (**52**)	leave	10,192 ± 18,342	6,855 ± 12,544	9,154 ± 16,669	9,819 ± 17,680	3.71e-34	1.10e-33	137.988	33.431	1.487	0.572	1.113	0.155	1.038	0.054
53	herniarin (**53**)	leave	20,105 ± 35,989	42,437 ± 76,570	22,037 ± 40,062	6,493 ± 11,670	9.72e-38	5.26e-37	196.232	37.013	0.474	-1.078	0.912	-0.132	3.096	1.631

Table S3. Continuation.

54	limettin (**54**)	leave	38,048 ± 80,560	45,722 ± 96,213	60,676 ± 110,758	66,748 ± 122,420	2.65e-16	3.02e-16	21.511	15.577	0.832	-0.265	0.627	-0.673	0.570	-0.811
55	psoralen (**55**)	leave	664 ± 1,294	7,134 ± 14,637	294.250 ± 545.549	3,403 ± 6,311	1.48e-12	1.55e-12	13.736	11.829	0.093	-3.426	2.255	1.173	0.195	-2.358
56	bergaptol (**56**)	leave	1,383 ± 2,497	3,446 ± 6,178	1,450 ± 2,606	4,307 ± 10,896	4.82e-07	4.82e-07	6.277	6.317	0.401	-1.317	0.954	-0.069	0.321	-1.639
57	xanthotoxol (**57**)	leave	34,497 ± 61,714	58,693 ± 105,576	29,371 ± 53,589	60,292 ± 115,117	1.92e-24	2.77e-24	51.870	23.718	0.588	-0.767	1.175	0.232	0.572	-0.805
58	4-hydroxycinnamic acid (**58**)	leave	1,825 ± 3,279	2,760 ± 4,943	2,216 ± 4,067	1,086 ± 1,944	4.26e-37	1.98e-36	184.276	36.371	0.661	-0.597	0.824	-0.280	1.680	0.749
59	3-*O*-*p*-coumaroylquinic acid (**59**)	leave	149.312 ± 273.283	824 ± 1,476	116.750 ± 218.454	5,349 ± 9,763	1.27e-30	2.50e-30	97.129	29.896	0.181	-2.465	1.279	0.355	0.028	-5.163
60	coumaroylputrescine (**60**)	leave	22,502 ± 40,965	9,940 ± 18,072	23,074 ± 41,417	13,427 ± 24,096	4.59e-36	1.87e-35	166.503	35.338	2.264	1.179	0.975	-0.036	1.676	0.745
61	*p*-coumaroylglucose (**61**)	leave	158.062 ± 286.448	531.688 ± 965.899	97.188 ± 179.128	1,975 ± 3,393	2.96e-51	9.61e-50	797.129	50.529	0.297	-1.750	1.626	0.702	0.080	-3.643
62	kaempferol 3-*O*-rutinoside (**62**)	leave	4,080 ± 7,311	1,473 ± 2,675	1,305 ± 2,364	2,103 ± 3,764	2.26e-47	4.89e-46	500.669	46.647	2.769	1.470	3.125	1.644	1.940	0.956
63	oxypeucedanin (**63**)	leave	1,467 ± 2,661	1,742 ± 3,187	5,065 ± 9,076	1,576 ± 3,052	6.86e-36	2.62e-35	163.683	35.164	0.842	-0.248	0.290	-1.788	0.931	-0.104
64	oxypeucedanin hydrate (**64**)	leave	3,565 ± 3,722	1,711 ± 1,775	2,026 ± 2,119	2,027 ± 2,191	9.07e-41	9.82e-40	263.801	40.043	2.084	1.060	1.760	0.815	1.759	0.815
65	bergamottin (**65**)	leave	41,323 ± 74,311	7,437 ± 24,923	34,744 ± 64,295	14,721 ± 27,365	1.48e-22	1.93e-22	42.595	21.829	5.556	2.474	1.189	0.250	2.807	1.489
66	pheophorbide A (**66**)	leave	12,182 ± 21,948	5,989 ± 10,803	9,908 ± 17,783	13,577 ± 24,482	8.44e-40	6.86e-39	240.024	39.074	2.034	1.024	1.230	0.298	0.897	-0.156

Abbreviations: FC, fold change relative to Control; log2FC, log2 fold change relative to Control.
Statistics: One-way ANOVA; p-values adjusted by Benjamini–Hochberg to obtain FDR (q).
Common thresholds: $q < 0.05$ and $|\log2FC| \geq 0.585$ ($\approx$ 1.5-fold).
